# Design and Synthesis of Various 5′-Deoxy-5′-(4-Substituted-1,2,3-Triazol-1-yl)-Uridine Analogues as Inhibitors of *Mycobacterium tuberculosis* Mur Ligases

**DOI:** 10.3390/molecules25214953

**Published:** 2020-10-26

**Authors:** Vincent Hervin, Ritu Arora, Jyoti Rani, Srinivasan Ramchandran, Urmi Bajpai, Luigi A. Agrofoglio, Vincent Roy

**Affiliations:** 1 Institute of Organic and Analytical Chemistry, CNRS UMR 7311, Universite d’Orléans, Rue de Chartres, CEDEX 2, 45067 Orleans, France; vincent.hervin@univ-orleans.fr; 2 Department of Biomedical Science, Acharya Narendra Dev College, University of Delhi, New Delhi 110019, India; rituarora1391@gmail.com (R.A.); sharmajyoti829@gmail.com (J.R.); urmibajpai@andc.du.ac.in (U.B.); 3 G N Ramachandran Knowledge of Centre, Council of Scientific and Industrial Research-Institute of Genomics and Integrative Biology (CSIR-IGIB), Room No. 130, Mathura Road, New Delhi 110025, India; ramu@igib.res.in

**Keywords:** Mur ligase, nucleoside analogues, copper-catalyzed azide-alkyne cycloaddition, antibacterial agents, molecular modelling

## Abstract

The synthesis of hitherto unknown 5′-deoxy-5′-(4-substituted-1,2,3-triazol-1-yl)-uridine and its evaluation, through an one-pot screening assay, against MurA-F enzymes involved in *Mycobacterium tuberculosis* (Mtb), are described. Starting from UDP-*N*-acetylmuramic acid (UDP-MurNAc), the natural substrate involved in the peptidoglycan biosynthesis, our strategy was to substitute the diphosphate group of UDP-MurNAc by a 1,2,3-triazolo spacer under copper-catalyzed azide-alkyne cycloaddition conditions. The structure-activity relationship was discussed and among the 23 novel compounds developed, *N*-acetylglucosamine analogues **11c** and **11e** emerged as the best inhibitors against the Mtb MurA-F enzymes reconstruction pathway with an inhibitory effect of 56% and 50%, respectively, at 100 μM. Both compounds are selective inhibitors of Mtb MurE, the molecular docking and molecular dynamic simulation suggesting that **11c** and **11e** are occupying the active site of Mtb MurE ligase.

## 1. Introduction

**Figure 1:**
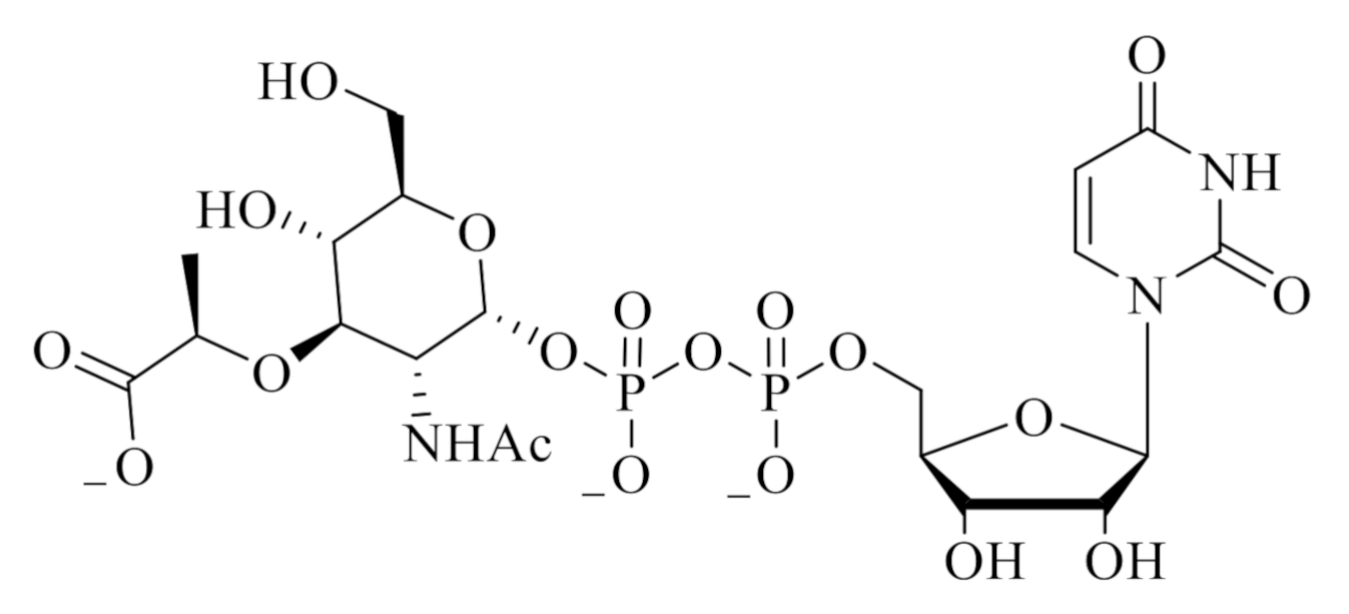
Structure of the natural substrate UDP-MurNAc.

*Mycobacterium tuberculosis* (Mtb) disease is one of the top 10 causes of death worldwide and has developed multi-drug resistance [1]. The viability of Mtb is directly linked to its remarkable cell wall structure and especially the peptidoglycan layer. Mur ligases enable the development of cell walls through cytoplasmic and periplasmic biosynthesis [2,3]. Thus, cell wall biosynthesis and especially Mur ligases appear to be a relevant target for new antibiotics [4,5] especially since bacteria have developed different alarming resistance against antibiotics drugs [6,7,8]. Of interest, the amide ligases MurC, MurD, MurE and MurF share the same catalytic function with similar amino acid regions and preserve comparable structural properties that must be exploited for the design of multi-inhibition molecules, which can reduce the incidence of bacterial resistance [3,5,9]. As part of our drug discovery program, we aim to develop new sugar-nucleotides with structural diversity targeting Mtb Mur ligases using quick synthetic approaches; the key step of the proposed chemistry follows the synthetic ecofriendly 1,3-dipolar cycloaddition [10] such as described by Huisgen [11] and Sharpless [12]. The [1,2,3]-Triazole ring can be considered as a phosphate mimic which imparts stability compared to the diphosphate unit, and a linker as well. Herein, we report the synthesis and enzymatic inhibition evaluation of 5′-deoxy-5′-(4-substituted-1,2,3-triazol-1-yl) uridines as analogs of UDP-MurNAc, the natural substrate of all MurA-F enzymes involved in peptidoglycan biosynthesis, (Figure 1).

## 2. Results and Discussion

### 2.1. Chemistry

**Scheme 1:**
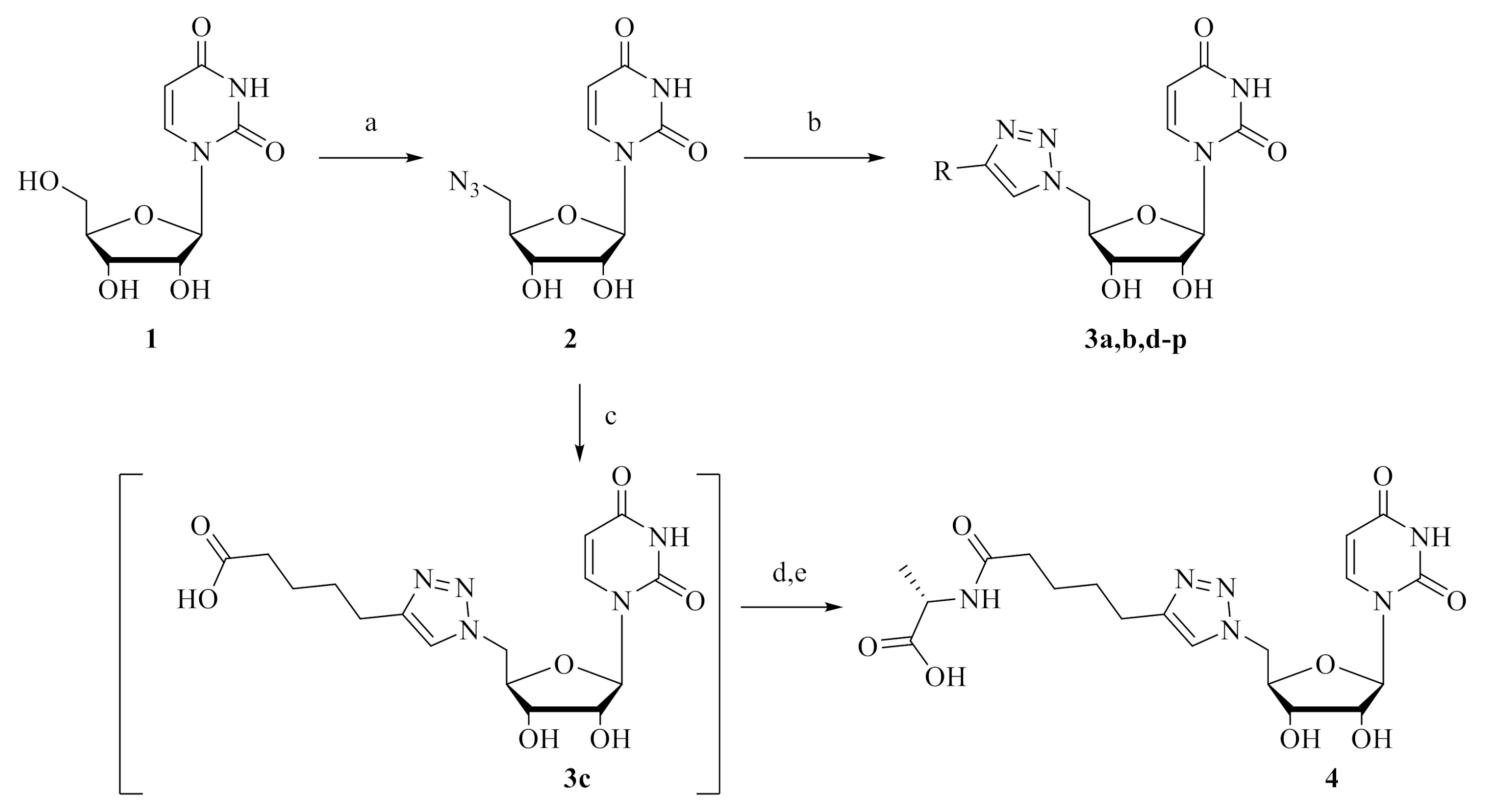
Reagents and conditions. (a) NaN_3_, Ph_3_P, CBr_4_, DMF, 25 °C, 24 h, 94%; (b) CuSO_4_.5H_2_O (10 mol%), sodium ascorbate (0.6 eq), substituted alkynes, *t*BuOH:H_2_O (2:1), 40 °C, 35 min-19 h, from 46% to 99%; (c) CuSO_4_.5H_2_O, sodium ascorbate, 6-heptynoic acid, *t*BuOH:H_2_O (2:1), 40 °C; (d) HOBt, BOP reagent, DIPEA, L-alanine methyl ester hydrochloride, dry DMF, 25 °C, 16 h; (**e**) LiOH.H_2_O, THF:H_2_O (3:1), 25 °C, 18 h, then H^+^, 29% from 2 (over 3 steps). where R is = (a) (CH_2_)_2_CO_2_H; (b) (CH_2_)_3_CO_2_H; (c) (CH_2_)_4_CO_2_H; (d) CH_2_OH; (e) (CH_2_)_2_OH; (f) (CH_2_)_3_OH; (g) CH_2_NH_2_; (h) CH_2_C(O)NH_2_; (i) CH_2_NH(C(O)(CH_2_)_4_CH_3_; (j) CH_2_NHC(O)(CH_2_)_6_CH_3_; (k) (m,m’)-(CO_2_CH_3_)_2_Ph; (l) (m,m’)-(OCH_3_)_2_Ph; (m) p-NO_2_-Ph; (n) 2-pyrimidinyl; (o) 2-thiofuranyl; (p) CH_2_-(1-β)-*O*-peracetylated glucose.

5’-Azido-5′-deoxy-uridine **2** was obtained according to the literature [13,14] on gram scale in 94% yield, through a one pot reaction, starting from commercially available uridine (**1**) in the presence of tetrabromomethane and triphenylphosphine through an in situ formation of 5′-bromo-5′-deoxy-uridine and subsequent substitution with sodium azide (Scheme 1). Azido analogue **2** was then reacted with a small library of alkyne derivatives bearing alcohols, amines, amides, carboxylic acids, aromatics and sugar moieties derivatives, chosen for their ability to form possible hydrogen bond interactions with the Mur ligase binding site and as a starting point in the development of Mur ligase inhibitors.

The 1,3-dipolar cycloaddition between compound **2** and alkynes was performed in water/*tert*-butanol (1:2) solvent mixture at 40 °C with sodium ascorbate/CuSO_4_. Compounds **3a**–**p** were isolated in yields ranging from 46% to 99%, and ^1^H- and ^13^C-NMR (HMBC) confirmed the regioselectivity of (1,4)-disubstituted [1,2,3]-triazole ring formation, without contamination by the (1,5)-regioisomer, (see Supplementary Materials).

Following the structure of the natural substrate UDP-MurNAc, we also synthesized the L-alanine peptidic derivative **4**, rationally designed to interact at the natural substrate and amino acid pocket on the active site of Mtb Mur ligases. Peptidic analogue **4** was obtained from acidic derivative **3c** in 29% yield (three steps, from **2**) using a peptide coupling reaction in the presence of hydroxybenzotriazole (HOBt) and benzotriazol-1-yloxytris (dimethylamino)phosphonium hexa- fluoro-phosphate (BOP) reagent followed by saponification and acidification.

**Scheme 2:**
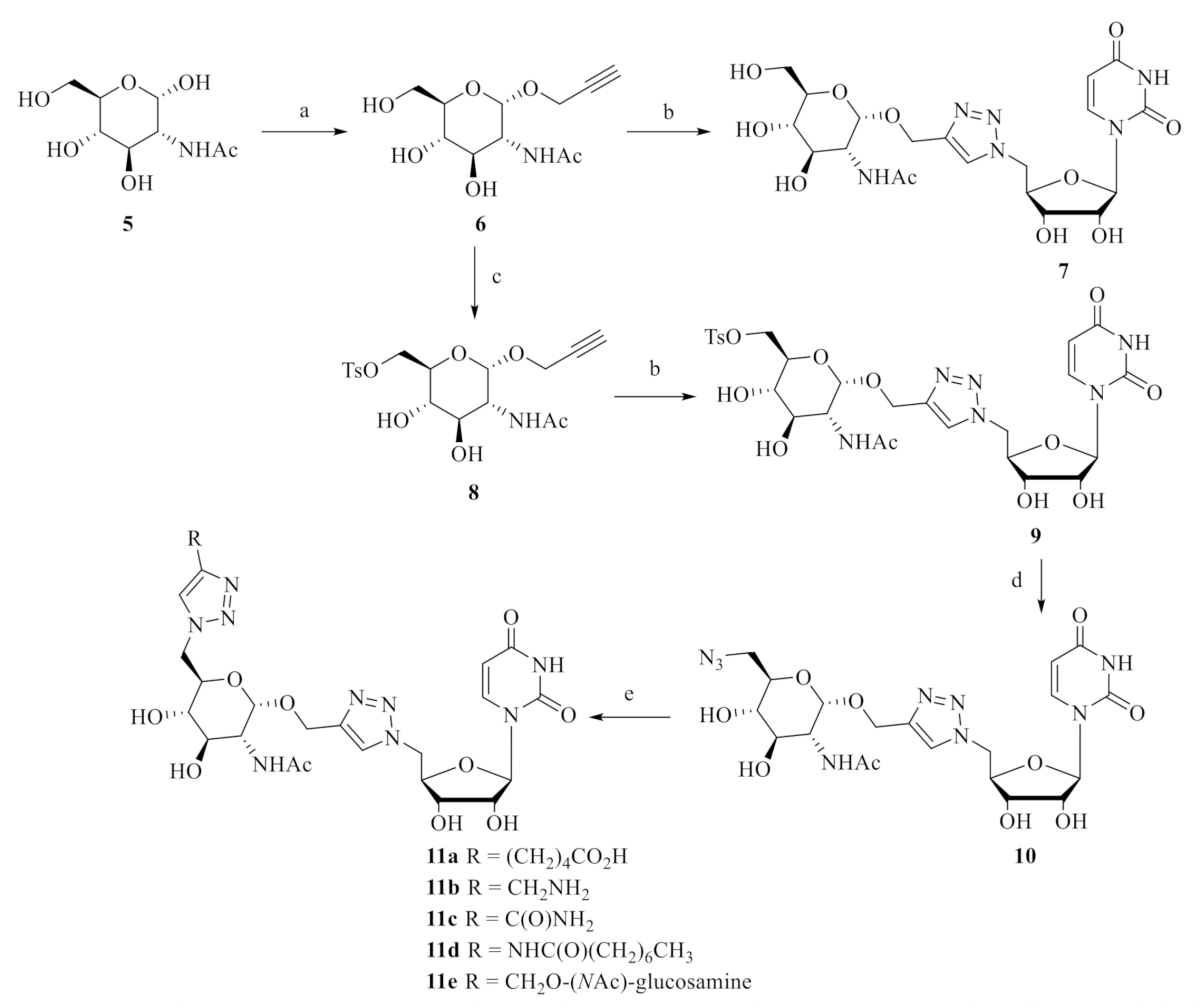
Reagents and conditions: (a) TMSOTf, propargyl alcohol, 4Å MS, 80 °C, 3 h, 52%; (b) CuSO_4_.5H_2_O, sodium ascorbate, compound **2**, *t*BuOH:H_2_O (2:1), 40 °C, 59% (**7**) and 82% (**9**) (c) TsCl, dry pyridine, 25 °C, 16 h, 50%; (d) NaN_3_, dry DMF, 25 °C, 72 h, 64%; (e) CuSO_4_·5H_2_O, sodium ascorbate, substituted alkynes, *t*BuOH:H_2_O (2:1), 40 °C, 19–90%.

We next investigated the biological influence of the *N*-acetylmuramic acid group on Mur ligase inhibition. The *N*-acetylglucosamine moiety was thus connected to the C5′-position of uridine through a CuAAc reaction, to replace the diphosphate group by the biologically stable [1,2,3]-triazolo spacer (Scheme 2). Starting from commercially available *N*-acetylglucosamine **5**, the anomeric position was substituted in the presence of propargylic alcohol and trimethylsilyl trifluoromethane-sulfonate (TMSOTf) to give the alkyne derivative **6** in 52% yield. CuAAC reaction between 5-azidouridine **2** and **6** give the desired *N*-acetylglucosamine uridine **7** in 59% yield. We also examined the influence of the C6-subtituted *N*-acetyl glucosamine derivatives **11a**–**e** on the inhibition activity. Tosylation of the primary alcohol at the C6-position of **6** in the presence of tosyl chloride and pyridine give the intermediate **8**, which was converted through CuAAC reaction in the presence of 5′-azido-5′-deoxyuridine **2** to the corresponding glucosamine-uridine intermediate **9**. Nucleophilic substitution of the tosyl group with sodium azide produced the intermediate **10** in moderate 64% yield. Intermediate **10** was then subjected to CuAAc reactions with various alkynes derivatives chosen for their abilities to generate hydrogen bonds and final derivatives **11a**–**e** were obtained in yields ranging from 19% to 90%.

All synthesized compounds were fully characterized and evaluated for their biological activity against *Mycobacterium tuberculosi*s MurA-F enzymes.

### 2.2. In Vitro Mur Ligase Inhibition Activity

Firstly, we evaluated the inhibitory activity of the synthesized 5′-deoxy-5′-(4-substituted-1,2,3-triazol-1-yl) uridines against *Mycobacterium tuberculosis* (Mtb) MurA-F enzymes in vitro*,* through our recently published [15] “one-pot assay” in order to screen and identify molecules with potential biological activity against a pool of Mtb MurA-F enzymes. The result from this in vitro screening are reported in Table 1 for compounds **3a**–**p**, **4** with 4-substituted-1,2,3-triazole moiety bearing an alkyl chain with terminal carboxylic acid **3a**–**c**, alcohol **3d**–**f**, amine/amide **3h**–**k** functional groups, peptidic **4**, which could afford hydrogen bond with the active site of Mtb Mur ligases. The results from Table 1 show that with an increased distance between the carboxylic acid and triazole ring (compounds **3a**–**c**), the inhibition increased from 11 to 29%, but conversely, the inhibitory activity of alcoholic compounds **3d**–**f** decreased from 19 to 14% with the alkyl chain length. Further substitution by amine **3g**, amide **3h** and amide-triazolyl bearing hydrophobic alkyl chains **3i**–**j** did not show improved inhibitory effects (39%). Both aromatic substitutions **3k**–**o**, with increased lipophilicity, electron withdrawing (ester-, nitro-) or donating (methoxy-) effect or a pyridine ring, did not display inhibitory activity exceeding 25%. Additionally, substitutions of the triazolo moiety by percylated glucose **3p** and L-alanine peptidic moiety **4** also did not exhibit better inhibition activity with 30% and 21% respectively. 

The second series of molecules bearing glucosamine derivatives **7**, **11a**–**e** were also tested, respectively, for their inhibitory effect against the one-pot assay (Table 2). Compared with the scaffold **7**, we found substitution by triazole derivatives at C6-position of glucosamine **11a**–**e** to increase the inhibitory activity. Substitution by carboxylic acid **11a** or amino group **11b** showed a moderate 25–30% of inhibition. The inhibitory effect could be improved up to 56% for the amide derivative group **11c** or glucosamine **11e** (50%). This effect could be due to suitable H-bonding between **11c,e** and the enzyme catalytic site.

Secondly, in order to define and elucidate the binding modes and specific inhibitory activities of the only compounds which exhibited ≥50% inhibition of one-pot assay containing Mtb MurA-F enzymes, we evaluated the abilities of **11c** and **11e**, respectively, to inhibit each of Mtb MurC-F ligases, taken separately, at 100 µM (Table 3).

Based on the individual assay analysis against Mur C, MurD, MurE and MurF enzymes compounds **11c** and **11e** were found to inhibit MurE enzyme selectively (Table 3). Also, since out of the four Mtb Mur ligases, the crystal structure of MurE has been solved [16], we did a computational analysis (molecular docking and molecular dynamic simulation) to investigate the stability of interaction between Mtb MurE and the compounds **11c** and **11e**.

### 2.3. Molecular Docking

Molecular docking of compounds **11c** and **11e**, respectively, with Mtb MurE showed a docking score of −9.9 kcal/mol, which is well below the chosen cut-off of −9.0 kcal/mol docking score with the natural ligand for Mtb MurE.

**Figure 2:**
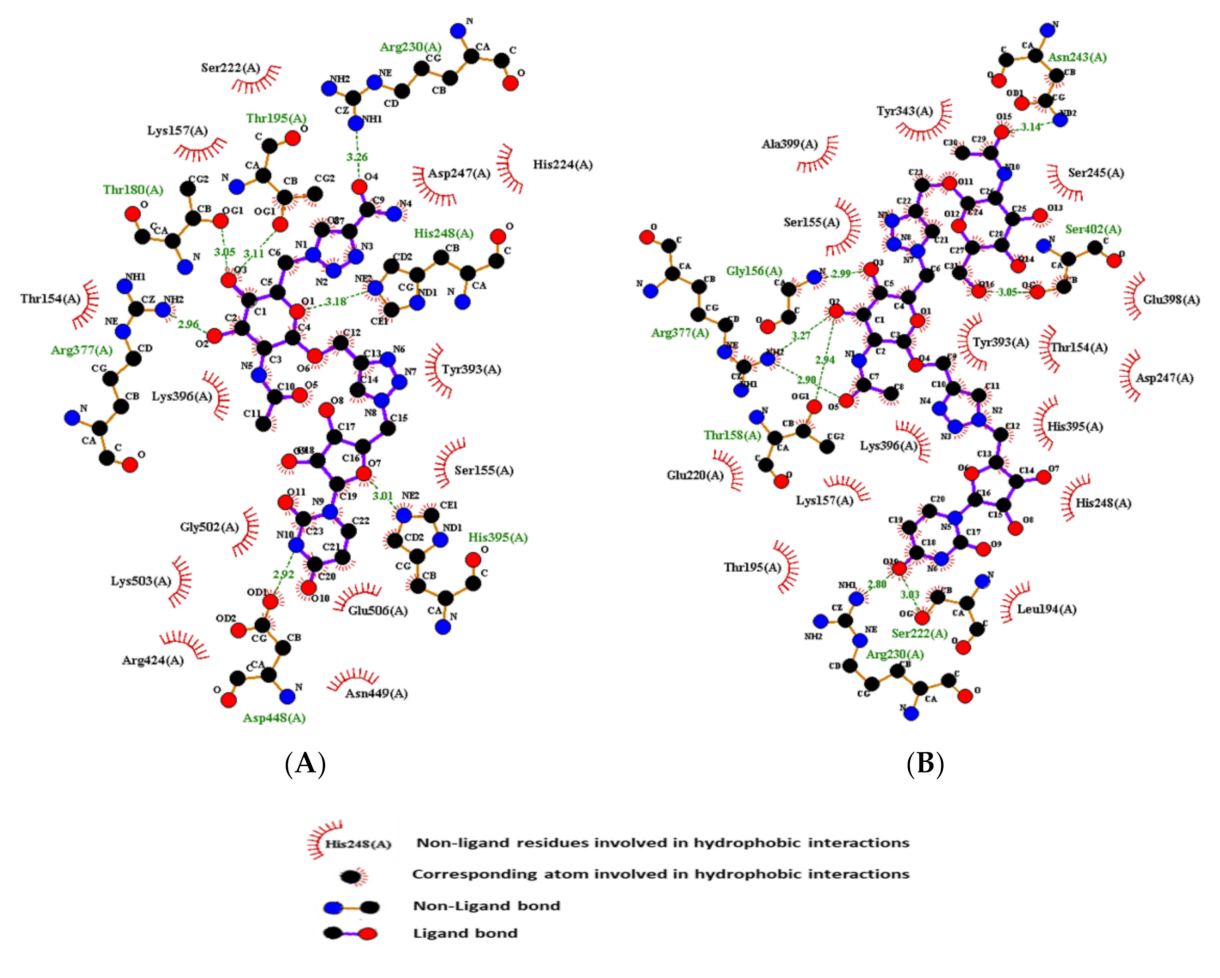
Hydrogen bonds and hydrophobic interactions between Mtb MurE and **11c** (**A**) and **11e** (**B**), respectively. The green dotted lines depict the hydrogen bonds whereas the other residues represent the hydrophobic interactions with the respective compounds.

Once the selected compounds were docked and filtered on the basis of binding affinity, they were visualized using Pymol [17], for the identification of binding residues. The binding pocket of MurE contains the following amino acid residues: LEU67, ARG68, ALA69, GLN70, LYS157, ARG230, HIS248, SER222, THR195, and LEU194. Compound **11c** with binding affinity −9.9 kcal/mol formed hydrogen bonds with THR154, SER155, ARG377, TYR397, GLY502, THR180, THR195, HIS395, ARG230, ASN449, ASP448, and HIS248 amino acid residues (Figure 2A). Compound **11e** with same binding affinity (–9.9 kcal/mol) formed hydrogen bonds to the ARG230, SER222, THR196, ASP247, ASN243, SER155, GLY156, THR180, GLY156, ARG377, SER402 residues of Mtb MurE (Figure 2B). This visual rescoring of the docked complex revealed the possession of compounds in the binding pocket of the protein. The stability of their binding tendency was further confirmed by using molecular dynamic simulation technique.

### 2.4. Molecular Dynamic Simulation

**Figure 3:**
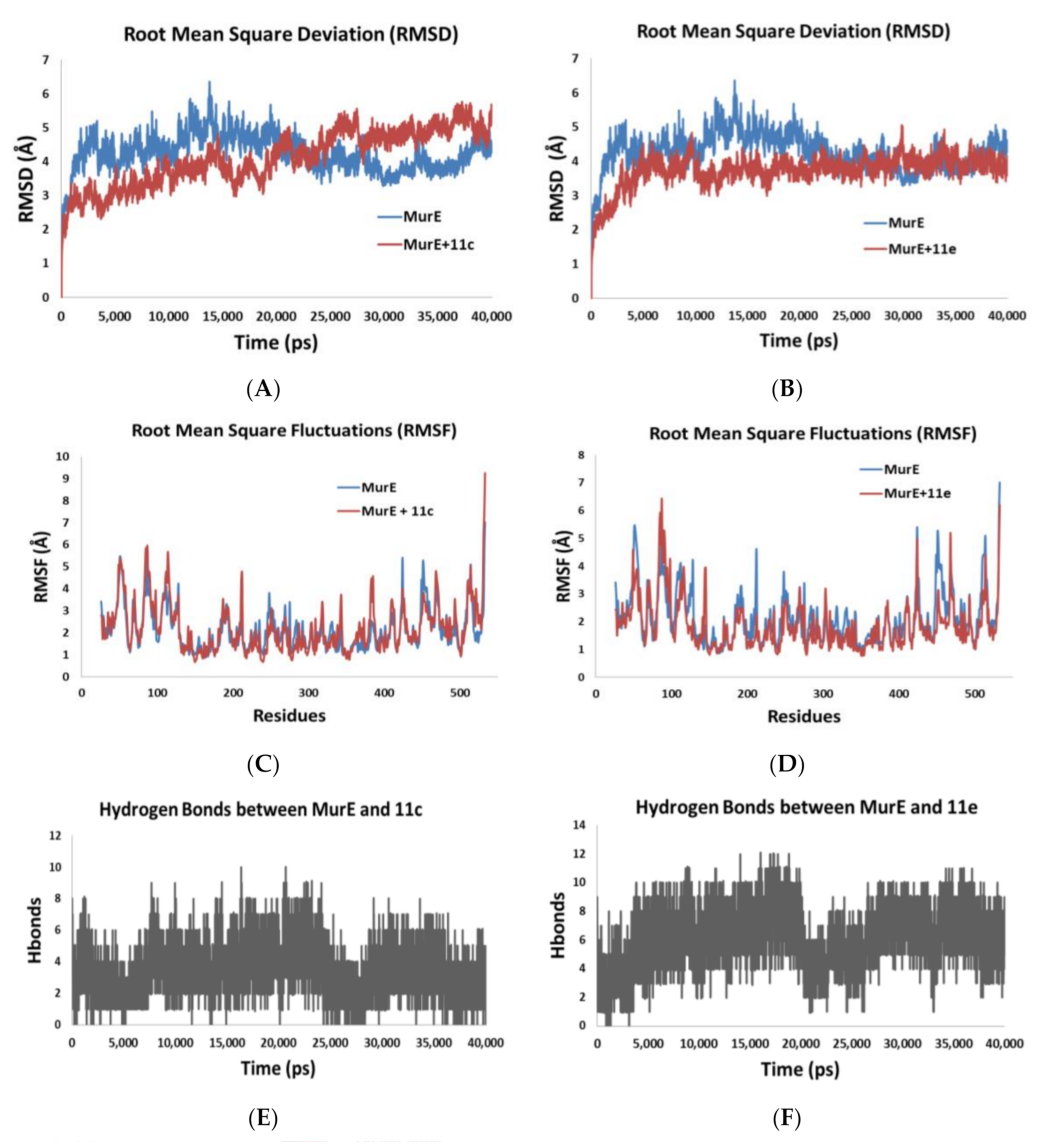
Molecular dynamic simulation plots of compounds **11c** and **11e**, respectively, with Mtb MurE: (**A**) and (**B**): RMSD plots; (**C**) and (**D**): RMSF plots; (**E**) and (**F**): hydrogen bonds.

Further, stability of the Mtb MurE-compound complexes was examined by MD simulation. MD simulation was carried out for 40 ns and the stability of the complexes was analyzed by calculating RMSD and the residual fluctuation in the protein was measured by evaluating the RMSF. The RMSD values for the complex were compared with the native protein in order to find whether the ligand remains bound or drifts away from the active site. We obtained lower RMSD values for both **11c** and **11e** (Figure 3A,B), thus suggesting the compounds to have occupied the active site of Mtb MurE throughout the simulation. While terminals of the protein showed higher fluctuations after docking with the compound **11c** with compound **11e**, protein residues exhibited less fluctuation (Figure 3C,D). For both the compounds, the binding site residues did not show much fluctuation throughout the simulation. On examining the hydrogen bond pattern (Figure 3E,F), stable hydrogen bonding was noted between the compounds **11c** and **11e** with the active site residues of Mtb MurE.

## 3. Materials and Methods

### 3.1. General Information

Commercially available chemicals were provided as reagent grade and used as received. Some reactions requiring anhydrous conditions were carried out using oven-dried glassware and under an atmosphere of dry argon. All anhydrous solvents were provided from commercially sources as very dry reagents. The reactions were monitored by thin layer chromatography (TLC) analysis using silica gel precoated plates (Kieselgel 60F254, E. Merck, Darmstadt, Germany). Compounds were visualized by UV irradiation (Sigma Aldrich Chimie, St-Quentin-Fallavier, France) and/or spraying with sulfuric acid (H_2_SO_4_ 5% in ethanol) stain followed by charring at average 150 °C. Flash column chromatography was performed on Silica Gel 60 M (0.040–0.063 mm, E. Merck, Darmstadt, Germany. The infrared spectra were measured with a Thermo Fischer Nicolet iS 10 FTIR spectrometer (Illkirch, France). The ^1^H and ^13^C-NMR spectra were recorded on an Bruker Avance DPX 250 or Avance 400 spectrometer (Champs sur Marne, France). Chemical shifts are given in ppm and are referenced to the deuterated solvent signal or to TMS as internal standard and multiplicities are reported as s (singlet), d (doublet), t (triplet), q (quartet) and m (multiplet). Carbon multiplicities were assigned by distortion less enhancement by polarization transfer (DEPT) experiments. ^1^H and ^13^C signals were attributed on the basis of H-H and H-C correlations. High Resolution Mass spectra were performed on a Bruker Q-TOF MaXis spectrometer by the “Fédération de Recherche” ICOA/CBM (FR2708) platform. LC-MS data was acquired on a Thermo-Fisher UHPLC-MSQ system (Illkirch, France) equipped with an electron spray ionization source (ESI). The temperature of the source was maintained at 350 °C. Initially, the cone voltage was set at 35 V and after 5 min was increased to 75 V. In full scan mode, data was acquired between 100 and 1000 *m*/*z* in the positive mode with a 1.00 s scan time. In addition, a UV detection was performed with a Diode array detector at three wavelengths 273, 254 and 290 nm, respectively. A water/methanol (70%/30%) solution mixture with 0.1% formic acid was used as mobile phase. The composition of the mobile phase was increased to 100% methanol with 0.1% formic acid with a 7% ramp. The flow rate was set at 0.300 mL min^−1^. Samples diluted in the mobile phase were injected (3 μL) on a C18 column (X-terra, Waters, Guyancourt, France), 2.1 mm internal diameter, and 100 mm length placed into an oven at 40 °C. Electronic extraction of ions was performed and the subsequent areas under the corresponding chromatographic peaks determined.

### 3.2. Synthesis

#### 3.2.1. Preparation of 1-((2R,3R,4S,5R)-5-(azidomethyl)-3,4-dihydroxytetrahydrofuran-2-yl)-pyrimidine-2,4-(1H,3H)-dione (2; CAS: 39483-48-2)

To a flame-dried round-bottom flask, tetrabromomethane (10.2 g, 30.8 mmol, 1.5 eq) was added to a solution of uridine **1** (5.0 g, 20.5 mmol, 1.0 eq), triphenylphosphine (7.68 g, 29.3 mol, 1.43 eq) and sodium azide (4.0 g, 61.5 mmol, 3.0 eq) with dry DMF (50 mL) at 25 °C under argon atmosphere. Then, the solution was stirred for 24 h. The reaction mixture became a slightly yellow pale solution. This was stopped and concentrated to dryness in vacuo. The resulting residue was purified by flash chromatography (DCM/MeOH 9/1) to give a white solid (5.20 g, 94%). Rf (DCM/MeOH 9:1) = 0.33. ^1^H-NMR (400 MHz, (CD_3_)_2_CO) δ (ppm): 10.0 (1H, s, *N*-H); 7.66 (1H, d, *J* = 8.09 Hz, =CH); 5.86 (1H, d, *J* = 4.43 Hz, CH); 5.62 (1H, d, *J* = 8.09 Hz, =CH); 4.73 (1H, m, CH); 4.45 (1H, m, CH); 4.32 (1H, m, CH); 4.18 (1H, m, CH); 4.04 (1H, m, CH); 3.60-3.72 (2H, m, CH_2_). ^13^C-NMR (100 MHz, (CD_3_)_2_CO) δ (ppm):162.5 (C=O); 150.5 (C=O); 140.7; 102.1; 90.2; 82.4; 73.4; 70.7; 52.0. ESI-HRMS calculated for [C_9_H_12_N_5_O_5_]^+^ *m*/*z* 270.0832 and found *m*/*z* 270.0832. IR (neat,cm^−1^): 3368.4*br*; 3100.2*m*; 3060.1*m*; 2101.6*s*; 1662.9*s*; 1463.2*m*; 1385.7*m*; 1264.7*m*; 1100.2*m*; 1034.2*m*; 811.5*w*; 764.4*w*. [α]_D_^20^ = + 27.4 (C = 0.02 M, MeOH). LC-MS purity 98.9%, t_R_ = 3.51 min water/methanol (70:30 *vol*/*vol*, 0.1% of formic acid).

#### 3.2.2. General Procedure: CuAAc Reaction

To a solution of 5′-azidouridine **2** (1.0 eq) in *t-*BuOH:H_2_O (2:1) (10 mL/0.5 mmol), the corresponding alkyne (1.0 eq), copper sulfate pentahydrate (10 mol%) and (+)-sodium ascorbate (0.6 eq) were added sequentially. Then the reaction mixture was stirred at 40 °C and monitored by TLC. When the starting material was completely converted, the reaction was stopped and co-evaporated twice with MeOH (20 mL/0.5 mmol). The residue was purified by flash chromatography (H_2_O/^I^PrOH/EtOAc 1/6/3 or DCM/MeOH 9/1) and then the residue was co-evaporated several times with MeOH (*x* × 15 mL) to remove residual water. Next, a second flash chromatography purification (acetone/MeOH 9/1 or EtOAc/MeOH 9/1) was used to give a pure product. All analysis are described according to each alkyne used.

*3-(1-(((2R,3S,4R,5R)-5-(2,4-Dioxo-3,4-dihydropyrimidin-1(2H)-yl)-3,4-dihydroxytetrahydrofuran-2-yl)-methyl)-1H-1,2,3-triazol-4-yl)propanoic acid* (**3a**). According to the general procedure, copper (II) sulfate pentahydrate (6 mg, 0.02 mmol, 10 mol%) and sodium ascorbate (28 mg, 0.14 mmol, 0.6 eq) were added to a solution of 5′-azido-5′-deoxyuridine **2** (65 mg, 0.24 mmol, 1.0 eq) and 4-pentynoic acid (24 mg, 0.24 mmol, 1.0 eq) with *t*BuOH:H_2_O (4 mL). The reaction mixture was stirred for 2.5 h at 40 °C. The residue was purified by a flash chromatography (H_2_O/^i^PrOH/EtOAc 1:6:3) followed by a second flash chromatography (acetone/MeOH 9/1) to give the desired product as white solid (50 mg, 56%). Rf (H_2_O/^i^PrOH/EtOAc 1:6:3) = 0.30. ^1^H-NMR (400 MHz, DMSO-d_6_) δ (ppm): 11.37 (1H, s, *N*-H); 7.81 (1H, s, =CH_triazol_); 7.49 (1H, d, *J* = 7.95 Hz, =CH); 5.74 (1H, d, *J* = 5.03 Hz, CH); 5.63 (1H, d, *J* = 7.95 Hz, CH); 4.56–4.68 (2H, m, CH_2_); 4.12 (1H, m, CH); 4.01 (1H, m, CH); 3.94 (1H, m, CH); 2.82 (2H, t, *J* = 7.79 Hz, CH_2_), 2.52 (2H, t, *J* = 7.72 Hz, CH_2_). ^13^C-NMR (100 MHz, DMSO-d_6_) δ (ppm): 174.5; 163.5; 151.1; 146.6; 141.5; 123.2; 102.5, 89.1; 82.2; 72.6; 71.0; 51.5; 34.3; 21.4. ESI-HRMS calculated for [C_14_H_18_N_5_O_7_]^+^ *m*/*z* 368.1200 and found *m*/*z* 368.1201. IR (neat, cm^−1^): 3201.0*br*; 2928.7*m*; 1682.4*s*; 1462.7*w*; 1384.5*m*; 1261.4*m*; 1100.6*m*; 1056.1*m*; 813.1*w*. [α]_D_^20^ = +32.7 (C = 0.017 M, MeOH). LC-MS purity 97.2%, t_R_ = 3.23 min water/methanol (70:30 *vol*/*vol*, 0.1% of formic acid).

*4-(1-(((2R,3S,4R,5R)-5-(2,4-Dioxo-3,4-dihydropyrimidin-1(2H)-yl)-3,4-dihydroxytetrahydrofuran-2-yl)-methyl)-1H-1,2,3-triazol-4-yl)butanoic acid *(**3b**). According to the general procedure, copper(II) sulfate pentahydrate (6 mg, 0.02 mmol, 10 mol%) and sodium ascorbate (28 mg, 0.14 mmol, 0.6 eq) were added to a solution of 5′-azido-5′-deoxyuridine **2** (65 mg, 0.24 mmol, 1.0 eq) and 5-hexynoic acid (27 mg, 0.24 mmol, 1.0 eq) with *t*BuOH:H_2_O (4 mL). The reaction mixture was stirred for 2.5 h at 40 °C. The residue was purified by a flash chromatography (H_2_O/^i^PrOH/EtOAc 1:6:3) followed by a second flash chromatography (acetone/MeOH 9/1) to give the desired product as white solid (55 mg, 60%). Rf (H_2_O/^i^PrOH/EtOAc 1:6:3) = 0.41. ^1^H-NMR (400 MHz, DMSO-d_6_) δ (ppm): 11.38 (1H, s, *N*-H); 7.85 (1H, s, =CH_triazol_); 7.50 (1H, d, *J* = 7.80 Hz, =CH); 5.74 (1H, d, *J* = 5.66 Hz, CH); 5.63 (1H, d, *J* = 7.80 Hz, =CH); 4.63 (2H, m, CH_2_); 4.13 (1H, m, CH); 4.03 (1H, t, *J* = 5.37 Hz, CH); 3.96 (1H, t, *J* = 4.78 Hz, CH); 2.62 (2H, t, *J* = 7.53 Hz, CH_2_), 2.25 (2H, t, *J* = 7.37 Hz, CH_2_) 1.80 (2H, q, *J* = 7.58 Hz, CH_2_). ^13^C-NMR (100 MHz DMSO-d_6_) δ (ppm): 174.8; 163.4; 151.1; 146.9; 141.5; 123.2; 102.5, 89.1; 82.2; 72.5; 71.0; 51.5; 33.7; 24.9; 24.8. ESI-HRMS calculated for [C_15_H_20_N_5_O_7_]^+^ *m*/*z* 382.1357 and found *m*/*z* 382.1356. IR (neat, cm^−1^): 3139.1*br*; 2925.2*m*; 1682.3*s*; 1462.2*w*; 1385.6*m*; 1261.9*m*; 1223.7*m*; 1100.5*m*; 1055.7*m*; 812.8*w*. [α]_D_^20^ = + 86.0 (C = 0.025 M, MeOH). LC-MS purity 95.5%, t_R_ = 3.87 min water/methanol (70:30 *vol*/*vol*, 0.1% of formic acid).

*5-(1-(((2R,3S,4R,5R)-5-(2,4-Dioxo-3,4-dihydropyrimidin-1(2H)-yl)-3,4-dihydroxytetrahydrofuran-2-yl)-methyl)-1H-1,2,3-triazol-4-yl)pentanoic acid *(**3c**). According to the general procedure, copper(II) sulfate pentahydrate (6 mg, 0.02 mmol, 10 mol%) and sodium ascorbate (28 mg, 0.14 mmol, 0.6 eq) were added to a solution of 5′-azido-5′-deoxyuridine **2** (65 mg, 0.24 mmol, 1.0 eq) and 6-heptynoic acid (34 mg, 0.24 mmol, 1.0 eq) with *t*BuOH:H_2_O (4 mL). The reaction mixture was stirred for 1 h at 40 °C. The residue was purified by a flash chromatography (H_2_O/^i^PrOH/EtOAc 1:6:3) followed by a second flash chromatography (acetone/MeOH 9/1) to give the desired product as white solid (60 mg, 63%). Rf (H_2_O/^i^PrOH/EtOAc 1:6:3) = 0.49. ^1^H-NMR (400 MHz, DMSO-d_6_) δ (ppm): 11.35 (1H, s, *N*-H); 7.81 (1H, s, =CH_triazol_); 7.50 (1H, d, *J* = 8.0 Hz, =CH); 5.73 (1H, d, *J* = 5.29 Hz, CH); 5.62 (1H, d, *J* = 8.0 Hz, CH); 4.62 (2H, m, CH_2_); 4.12 (1H, m, CH); 4.04 (1H, t, *J* = 5.28 Hz, CH); 3.95 (1H, t, *J* = 4.80 Hz, CH); 2.60 (2H, t, *J* = 6.80 Hz, CH_2_); 2.22 (2H, t, *J* = 7.26 Hz, CH_2_); 1.48-1.63 (4H, m, 2 CH_2_). ^13^C-NMR (100 MHz, DMSO-d_6_) δ (ppm): 174.9, 163.4, 151.1, 147.2, 141.5, 123.1, 102.5, 89.2, 82.2, 72.5, 71.0, 51.5, 33.9, 28.9, 25.1, 24.6. ESI-HRMS calculated for [C_16_H_22_N_5_O_7_]^+^ *m*/*z* 396.1513 and found *m*/*z* 396.1512. IR (neat, cm^−1^): 3142.7*br*; 2929.3*m*; 1682.5*s*; 1461.3*w*; 1384.1*m*; 1261.9*m*; 1222.6*m*; 1100.1*m*; 1052.0*m*; 811.7*w*. [α]_D_^20^ = + 44.5 (C = 0.015 M, MeOH). LC-MS purity 95.2%, t_R_ = 4.51 min water/methanol (70:30 *vol*/*vol*, 0.1% of formic acid).

*1-((2R,3R,4S,5R)-3,4-Dihydroxy-5-((4-(hydroxymethyl)-1H-1,2,3-triazol-1-yl)methyl)tetrahydrofuran-2-yl)pyrimidine-2,4(1H,3H)-dione *(**3d**). According to the general procedure, copper(II) sulfate pentahydrate (6 mg, 0.02 mmol, 10 mol%) and sodium ascorbate (28 mg, 0.14 mmol, 0.6 eq) were added to a solution of 5′-azido-5′-deoxyuridine **2** (65 mg, 0.24 mmol, 1.0 eq) and propargyl alcohol (14 mg, 0.24 mmol, 1.0 eq) with *t*BuOH:H_2_O (4 mL). The reaction mixture was stirred for 40 min at 40 °C. The residue was purified by a flash chromatography (H_2_O/^i^PrOH/EtOAc 0.1:6.9:3) followed by a second flash chromatography (acetone/MeOH 9/1) to give the desired product as white solid (54 mg, 68%). Rf (H_2_O/^i^PrOH/EtOAc 1:6:3) = 0.64. ^1^H-NMR (400 MHz, DMSO-d_6_) δ (ppm): 11.36 (1H, s, *N*-H); 7.92 (1H, s, =CH_triazol_); 7.50 (1H, d, *J* = 8.05 Hz, =CH); 5.74 (1H, d, *J* = 5.43 Hz, CH); 5.63 (1H, d, *J* = 7.97 Hz, =CH); 5.50 (1H, m, *O*-H); 5.39 (1H, m, *O*-H); 5.18 (1H, m, *O*-H); 4.66 (2H, m, CH_2_); 4.50 (2H, m, CH_2_); 4.14 (1H, m, CH); 4.03 (1H, m, CH); 3.97 (1H, m, CH). ^13^C-NMR (100 MHz, DMSO-d_6_) δ (ppm): 163.4, 151.3, 148.5, 141.5, 124.0, 102.6, 89.0, 82.3, 72.5, 71.0, 55.4, 51.6. ESI-HRMS calculated for [C_12_H_16_N_5_O_6_]^+^ *m*/*z* 326.1095 and found *m*/*z* 326.1096. IR (neat, cm^−1^): 3436.5*br*; 3044.1*w*; 2922.1*w*; 1689.7*s*; 1459.5*w*; 1419.4*m*; 1390.6*m*; 1282.4*m*; 1081.1*m*; 1015.3*m*; 993.0*m*; 792.2w. [α]_D_^20^ = + 47.6 (C = 0.015 M, MeOH). LC-MS purity 95%, t_R_ = 8.36 min water/methanol (70:30 *vol*/*vol*, 0.1% of formic acid).

*1-((2R,3R,4S,5R)-3,4-Dihydroxy-5-((4-(2-hydroxyethyl)-1H-1,2,3-triazol-1-yl)methyl)tetrahydrofuran-2-yl)-pyrimidine-2,4(1H,3H)-dione* (**3e**). According to the general procedure, copper(II) sulfate pentahydrate (6 mg, 0.02 mmol, 10 mol%) and sodium ascorbate (28 mg, 0.14 mmol, 0.6 eq) were added to a solution of 5′-azido-5′-deoxyuridine **2** (65 mg, 0.24 mmol, 1.0 eq) and 3-butyn-1-ol (17 mg, 0.24 mmol, 1.0 eq) with *t*BuOH:H_2_O (4 mL). The reaction mixture was stirred for 35 min at 40 °C. The residue was purified by a flash chromatography (acetone/MeOH 98/2) to give the desired product as white solid (47 mg, 57%). Rf (H_2_O/^i^PrOH/EtOAc 1:6:3) = 0.61. ^1^H-NMR (400 MHz, DMSO-d_6_) δ (ppm): 11.36 (1H, s, *N*-H); 7.82 (1H, s, =CH_triazol_); 7.47 (1H, d, *J* = 8.04 Hz, =CH); 5.74 (1H, d, *J* = 5.23 Hz, CH); 5.63 (1H, d, *J* = 8.04 Hz, =CH); 4.63 (2H, m, CH_2_); 4.13 (1H, m, CH); 4.00 (1H, m, CH); 3.96 (1H, m, CH); 3.62 (2H, t, *J* = 6.82 Hz, CH_2_); 2.76 (2H, t, *J* = 6.85 Hz, CH_2_). ^13^C-NMR (100 MHz, DMSO-d_6_) δ (ppm): 163.4, 151.1, 145.0, 141.5, 123.7, 102.6, 89.0, 82.2, 72.5, 70.9, 60.8, 51.5, 29.6. ESI-HRMS calculated for [C_13_H_18_N_5_O_6_]^+^ *m*/*z* 340.1251 and found *m*/*z* 340.1254. IR (neat, cm^−1^): 3424.1*br*; 2921.2*w*; 1691.5*s*; 1461.8*w*; 1422.2*m*; 1382.2*m*; 1279.1*m*; 1233.6*m*; 1044.1*m*; 1018.1*m*; 995.7*m*; 809.7w. [α]_D_^20^ = + 29.2 (C = 0.03 M, MeOH). LC-MS purity 94.2%, t_R_ = 2.58 min water/methanol (70:30 *vol*/*vol*, 0.1% of formic acid).

*1-((2R,3R,4S,5R)-3,4-Dihydroxy-5-((4-(3-hydroxypropyl)-1H-1,2,3-triazol-1-yl)methyl)tetrahydrofuran-2-yl)pyrimidine-2,4(1H,3H)-dione* (**3f**). According to the general procedure, copper(II) sulfate pentahydrate (6 mg, 0.02 mmol, 10 mol%) and sodium ascorbate (30 mg, 0.16 mmol, 0.6 eq) were added to a solution of 5′-azido-5′-deoxyuridine **2** (70 mg, 0.26 mmol, 1.0 eq) and 4-pentyn-1-ol (22 mg, 0.26 mmol, 1.0 eq) with *t*BuOH:H_2_O (4 mL). The reaction mixture was stirred for 45 min at 40 °C. The residue was purified by a flash chromatography (H_2_O/^i^PrOH/EtOAc 0.1:6.9:3) followed by a second flash chromatography (acetone/MeOH 95/5) to give the desired product as white solid (57 mg, 62%). Rf (H_2_O/^i^PrOH/EtOAc 1:6:3) = 0.63. ^1^H-NMR (400 MHz, DMSO-d_6_) δ (ppm): 11.36 (1H, s, *N*-H); 7.81 (1H, s, =CH_triazol_); 7.48 (1H, d, *J* = 8.02 Hz, =CH); 5.74 (1H, d, *J* = 5.55 Hz, CH); 5.62 (1H, d, *J* = 8.02 Hz, =CH); 5.49 (1H, m, *O*-H); 5.36 (1H, m, *O*-H); 4.62 (2H, m, CH_2_); 4.47 (1H, m, *O*-H); 4.13 (1H, m, CH); 4.03 (1H, m, CH); 3.96 (1H, m, CH); 3.42 (2H, m, CH_2_); 2.63 (2H, t, *J* = 7.57 Hz, CH_2_); 1.72 (2H, q, *J* = 6.77 Hz, CH_2_). ^13^C-NMR (100 MHz, DMSO-d_6_) δ (ppm): 163.5, 151.1, 147.3, 141.5, 123.1, 102.5, 89.1, 82.2, 72.5, 71.0, 60.5, 51.5, 32.7, 22.1. ESI-HRMS calculated for [C_14_H_20_N_5_O_6_]^+^ *m*/*z* 354.1408 and found *m*/*z* 354.1408. IR (neat, cm^−1^): 3367.9*br*; 2945.1*m*; 1673.9*s*; 1462.5*m*; 1422.9*m*; 1384.7*m*; 1263.2*m*; 1224.0*m*; 1100.2*m*; 1051.8*m*; 811.9w. [α]_D_^20^ = + 58.0 (C = 0.026 M, MeOH). LC-MS purity 94.6%, t_R_ = 3.24 min water/methanol (70:30 *vol*/*vol*, 0.1% of formic acid).

*1-((2R,3R,4S,5R)-5-((4-(Aminomethyl)-1H-1,2,3-triazol-1-yl)methyl)-3,4-dihydroxytetrahydrofuran-2-yl)-pyrimidine-2,4(1H,3H)-dione* (**3g**). According to the general procedure, copper(II) sulfate pentahydrate (18.5 mg, 0.07 mmol, 10 mol%) and sodium ascorbate (88.3 mg, 0.45 mmol, 0.6 eq) were added to a solution of 5′-azido-5′-deoxyuridine **2** (200 mg, 0.74 mmol, 1.0 eq) and propargyl amine (61 mg, 1.10 mmol, 1.5 eq) with *t*BuOH:H_2_O (10 mL). The reaction mixture was stirred for 1 h at 40 °C. The residue was purified by a flash chromatography (CHCl_3_:MeOH:AcOH 1:1:1) followed by a second flash chromatography (H_2_O:^i^PrOH:EtOAc:AcOH 1:6:2:1) to give the desired product as white solid (110 mg, 46%). Rf (CHCl_3_:MeOH:AcOH 1:1:1) = 0.28. ^1^H-NMR (400 MHz, DMSO-d_6_) δ (ppm): 7.88 (1H, s, =CH_triazol_); 7.52 (1H, d, *J* = 7.91 Hz, =CH); 5.74 (1H, d, *J* = 4.90 Hz, CH); 5.63 (1H, d, *J* = 7.91 Hz, =CH); 4.64 (2H, m, CH); 4.12 (1H, m, CH); 4.01 (1H, m, CH); 3.94 (1H, m, CH); 3.77 (2H, m, CH_2_). ^13^C-NMR (100 MHz, DMSO-d_6_) δ (ppm): 163.5, 151.1, 149.1, 141.6, 123.3, 102.5, 89.3, 82.3, 72.7, 71.1, 51.7, 37.2. ESI-HRMS calculated for [C_12_H_17_N_6_O_5_]^+^ *m*/*z* 325.1254 and found *m*/*z* 325.1254. IR (neat, cm^−1^): 3150.4*br*; 1673.9*s*; 1462.5*m*; 1385.5*m*; 1263.1*m*; 1099.7*m*; 1050.8*m*; 812.3w. [α]_D_^20^ = + 96.0 (C = 0.04 M, MeOH). LC-MS purity 95.1%, t_R_ = 0.72 min water/methanol (70:30 *vol*/*vol*, 0.1% of formic acid).

*1-(((2R,3S,4R,5R)-5-(2,4-Dioxo-3,4-dihydropyrimidin-1(2H)-yl)-3,4-dihydroxytetrahydrofuran-2-yl)methyl)-1H-1,2,3-triazole-4-carboxamide* (**3h**). According to the general procedure, copper(II) sulfate pentahydrate (18.5 mg, 0.07 mmol, 10 mol%) and sodium ascorbate (88.3 mg, 0.45 mmol, 0.6 eq) were added to a solution of 5′-azido-5′-deoxyuridine **2** (200 mg, 0.74 mmol, 1.0 eq) and propiolamide (51 mg, 0.74 mmol, 1.0 eq) with *t*BuOH:H_2_O (10 mL). The reaction mixture was stirred for 19 h at 40 °C. The residue was purified by a flash chromatography (acetone/MeOH 9/1) followed by a second flash chromatography (EtOAc/MeOH 9/1) to give the desired product as white solid (0.17 g, 68%). Rf (EtOAc:MeOH 9:1) = 0.21. ^1^H-NMR (400 MHz, DMSO-d_6_) δ (ppm): 11.37 (1H, s, *N*-H_3_); 8.47 (1H, s, =CH_triazol_); 7.85 (1H, s, *N*-H); 7.56 (1H, d, *J* = 8.16 Hz, =CH); 7.46 (1H, s, *N*-H); 5.74 (1H, d, *J* = 5.55 Hz, CH); 5.63 (1H, d, *J* = 8.16 Hz, CH); 5.52 (1H, d, *J* = 5.62 Hz, *O*-H); 5.40 (1H, d, *J* = 5.34 Hz, *O*-H); 4.74 (2H, m, CH_2_); 4.18 (1H, m, CH); 4.11 (1H, m, CH); 3.98 (1H, m, CH); 3.77 (2H, m, CH_2_). ^13^C-NMR (100 MHz, DMSO-d_6_) δ (ppm): 163.0, 161.5, 151.6, 142.9, 141.2, 127.2, 102.1, 88.9, 81.6, 72.1, 70.6, 51.5. ESI-HRMS calculated for [C_12_H_15_N_6_O_6_]^+^ *m*/*z* 339.1047 and found *m*/*z* 339.1048. IR (neat, cm^−1^): 3192.8*br*; 1690.5*s*; 1652.1*s*; 1466.2*w*; 1393.9*w*; 1266.7*w*; 1100.0*w*; 1045.7*w*; 819.8*w*; 779.5w. [α]_D_^20^ = + 37.9 (C = 0.04 M, MeOH). LC-MS purity 95.2%, t_R_ = 1.94 min water/methanol (70:30 *vol*/*vol*, 0.1% of formic acid).

*N-((1-(((2R,3S,4R,5R)-5-(2,4-Dioxo-3,4-dihydropyrimidin-1(2H)-yl)-3,4-dihydroxytetrahydrofuran-2-yl)methyl)-1H-1,2,3-triazol-4-yl)methyl)hexanamide* (**3i**). According to the general procedure, copper(II) sulfate pentahydrate (6 mg, 0.02 mmol, 10 mol%) and sodium ascorbate (28 mg, 0.14 mmol, 0.6 eq) were added to a solution of 5′-azido-5′-deoxyuridine **2** (65 mg, 0.24 mmol, 1.0 eq) and N-(prop-2-yn-1-yl)hexanamide (37 mg, 0.24 mmol, 1.0 eq) with *t*BuOH:H_2_O (3 mL). The reaction mixture was stirred for 2 h at 40 °C. The residue was purified by a flash chromatography (H_2_O/^i^PrOH/EtOAc 0.1:6.9:3) to give the desired product as white solid (99 mg, 99%). Rf (H_2_O/^i^PrOH/EtOAc 1:6:3) = 0.78. ^1^H-NMR (400 MHz, DMSO-d_6_) δ (ppm): 11.38 (1H, brs, *N*-H); 8.27 (1H, t, *J* = 5.68 Hz, *N*-H); 7.85 (1H, s, =CH_triazol_); 7.51 (1H, d, *J* = 8.09 Hz, =CH); 5.75 (1H, d, *J* = 5.24 Hz, CH); 5.65 (1H, d, = 8.09 Hz, =CH); 5.51 (1H, d, *J* = 5.70 Hz, *O*-H); 5.39 (1H, d, *J* = 5.22 Hz, *O*-H); 4.66 (2H, m, CH_2_); 4.27 (2H, d, *J* = 5.70 Hz, CH_2_); 4.13 (1H, m, CH); 4.05 (1H, m, CH); 3.96 (1H, m, CH); 2.07 (2H, t, *J* = 7.41 Hz, CH_2_); 1.49 (2H, m, CH_2_), 1.16-1.30 (4H, m, 2 CH_2_); 0.84 (3H, t, *J* = 6.78 Hz, CH_3_). ^13^C-NMR (100 MHz, DMSO-d_6_) δ (ppm): 172.6, 163.4, 151.1, 145.6, 141.5, 124.0, 102.6, 89.0, 82.2, 72.5, 71.0, 51.6, 35.6, 34.5, 29.1, 26.0, 22.3, 14.3. ESI-HRMS calculated for [C_18_H_27_N_6_O_6_]^+^ *m*/*z* 423.1986 and found *m*/*z* 423.1985. IR (neat, cm^−1^): 3295.5*br*; 2930.2*m*; 2871.3*m*; 1682.4*s*; 1538.6*m*; 1462.0*m*; 1424.0*m*; 1381.3*m*; 1258.8*m*; 1101.1*m*; 1051.7*m*; 813.0w. [α]_D_^20^ = + 42.1 (C = 0.02 M, MeOH).LC-MS purity 95%, t_R_ = 5.71 min water/methanol (70:30 *vol*/*vol*, 0.1% of formic acid).

*N-((1-(((2R,3S,4R,5R)-5-(2,4-Dioxo-3,4-dihydropyrimidin-1(2H)-yl)-3,4-dihydroxytetrahydrofuran-2-yl)methyl)-1H-1,2,3-triazol-4-yl)methyl)octanamide *(**3j**). According to the general procedure, copper(II) sulfate pentahydrate (6 mg, 0.02 mmol, 10 mol%) and sodium ascorbate (28 mg, 0.14 mmol, 0.6 eq) were added to a solution of 5′-azido-5′-deoxyuridine **2** (65 mg, 0.24 mmol, 1.0 eq) and N-(prop-2-yn-1-yl)octanamide (44 mg, 0.24 mmol, 1.0 eq) with *t*BuOH:H_2_O (3 mL). The reaction mixture was stirred for 2 h at 40 °C. The residue was purified by a flash chromatography (H_2_O/^i^PrOH/EtOAc 0.1:6.9:3) to give the desired product as white solid (85 mg, 77%). Rf (H_2_O/^i^PrOH/EtOAc 1:6:3) = 0.81. ^1^H-NMR (400 MHz, DMSO-d_6_) δ (ppm): 11.38 (1H, brs, *N*-H); 8.27 (1H, t, *J* = 5.56 Hz, *N*-H); 7.85 (1H, s, =CH_triazol_); 7.51 (1H, d, *J* = 8.06 Hz, =CH); 5.75 (1H, d, *J* = 5.24 Hz, CH); 5.65 (1H, d, *J* = 8.06 Hz, =CH); 5.51 (1H, d, *J* = 5.67 Hz, *O*-H); 5.38 (1H, d, *J* = 5.24 Hz, *O*-H); 4.66 (2H, m, CH_2_); 4.27 (2H, d, *J* = 5.46 Hz, CH_2_); 4.13 (1H, m, CH); 4.05 (1H, m, CH); 3.96 (1H, m, CH); 2.07 (2H, t, *J* = 7.34 Hz, CH_2_); 1.48 (2H, m, CH_2_), 1.23 (8H, m, 4 CH_2_); 0.85 (3H, t, *J* = 6.31 Hz, CH_3_). ^13^C-NMR (100 MHz, DMSO-d_6_) δ (ppm): 172.6, 163.4, 151.1, 145.6, 141.4, 124.0, 102.6, 89.1, 82.2, 72.6, 71.0, 51.6, 35.7, 34.5, 31.6, 29.1, 28.9, 25.7, 22.5, 14.4. ESI-HRMS calculated for [C_20_H_31_N_6_O_6_]^+^ *m*/*z* 451.2299 and found *m*/*z* 451.2299. IR (neat, cm^−1^): 3293.4*br*; 2927.2*m*; 2855.8*m*; 1682.3*s*; 1538.7*m*; 1462.1*m*; 1381.7*m*; 1263.1*m*; 1101.0*m*; 1052.4*m*; 812.0w. [α]_D_^20^ = + 61.4 (C = 0.02 M, MeOH). LC-MS purity 98.3%, t_R_ = 7.63 min water/methanol (70:30 *vol*/*vol*, 0.1% of formic acid).

*Dimethyl 5-(1-(((2R,3S,4R,5R)-5-(2,4-dioxo-3,4-dihydropyrimidin-1(2H)-yl)-3,4-dihydroxytetrahydrofuran-2-yl)methyl)-1H-1,2,3-triazol-4-yl)isophthalate* (**3k**). According to the general procedure, copper(II) sulfate pentahydrate (29 mg, 0.11 mmol, 10 mol%) and sodium ascorbate (136 mg, 0.69 mmol, 0.6 eq) were added to a solution of 5′-azido-5′-deoxy-uridine **2** (310 mg, 1.14 mmol, 1.0 eq) and dimethyl-5-ethynylisophathalate (250 mg, 1.14 mmol, 1.0 eq) with *t*BuOH:H_2_O (10 mL). The reaction mixture was stirred for 5 h 30 min at 40 °C. The residue was purified by a flash chromatography (DCM/MeOH 93/7 to 90/10) followed by a second flash chromatography (acetone/MeOH 99/1) to give the desired product as white solid (0.40 g, 71%). Rf (DCM/MeOH 9:1) = 0.45. ^1^H-NMR (400 MHz, DMSO-d_6_) δ (ppm): 11.36 (1H, s, *N*-H); 8.91 (1H, s, =CH_triazol_); 8.63 (2H, s, Ar-H); 8.39 (1H, m, Ar-H); 7.56 (1H, d, *J* = 8.09 Hz, =CH); 5.77 (1H, d, *J* = 5.06 Hz, CH); 5.59 (1H, d, *J* = 8.09 Hz, =CH); 5.52 (1H, d, *J* = 5.58 Hz, *O*-H); 5.41 (1H, d, *J* = 5.46 Hz, *O*-H); 4.76 (2H, m, CH_2_); 4.25 (1H, m, CH); 4.11 (1H, m, CH); 4.03 (1H, m, CH); 3.92 (6H, s, 2 CH_3_). ^13^C-NMR (100 MHz, DMSO-d_6_) δ (ppm): 165.2, 163.0, 150.6, 144.6, 141.2, 132.0, 131.0, 129.7, 128.6, 123.4, 102.1, 89.0, 82.5, 72.1, 70.5, 52.6, 51.6. ESI-HRMS calculated for [C_21_H_22_N_5_O_9_]^+^ *m*/*z* 488.1412 and found *m*/*z* 488.1411. IR (neat, cm^−1^): 3136.9*br*; 2955.2*m*; 1704.9*s*; 1467.1*m*; 1432.7*m*; 1302.5*m*; 1242.4*s*; 1204.8*m*; 1093.5*m*; 1057.2*m*; 996.4*w*; 819.5*w*; 753.7w. [α]_D_^20^ = + 23.5 (C = 0.03 M, MeOH). LC-MS purity 98.4%, t_R_ = 7.21 min water/methanol (70:30 *vol*/*vol*, 0.1% of formic acid).

*1-((2R,3R,4S,5R)-5-((4-(3,5-Dimethoxyphenyl)-1H-1,2,3-triazol-1-yl)methyl)-3,4-dihydroxytetrahydro-furan-2-yl)pyrimidine-2,4(1H,3H)-dione* (**3l**). According to the general procedure, copper(II) sulfate pentahydrate (18.5 mg, 0.07 mmol, 10 mol%) and sodium ascorbate (88 mg, 0.46 mmol, 0.6 eq) were added to a solution of 5′-azido-5′-deoxyuridine **2** (200 mg, 0.74 mmol, 1.0 eq) and 3,5-dimethoxyphenyl-1-ethynyl (120 mg, 0.74 mmol, 1.0 eq) with *t*BuOH:H_2_O (10 mL). The reaction mixture was stirred for 17 h at 40 °C. The residue was purified by a flash chromatography (DCM/MeOH 9/1) followed by a second flash chromatography (EtOAc/MeOH 95/5) to give the desired product as white solid (0.25 g, 78%). Rf (DCM/MeOH 9:1) = 0.33. ^1^H-NMR (400 MHz, DMSO-d_6_) δ (ppm): 11.36 (1H, d, *J* = 2.07 Hz, *N*-H); 8.60 (1H, s, =CH_triazol_); 7.53 (1H, d, *J* = 8.13 Hz, =CH); 7.02 (2H, d, *J* = 2.24 Hz, 2 Ar-H); 6.47 (1H, t, *J* = 2.24 Hz, Ar-H); 5.76 (1H, d, *J* = 5.32 Hz, =CH); 5.58 (1H, dd, *J* = 2.07 Hz, *J* = 8.13 Hz, =CH); 5.51 (1H, d, *J* = 5.56 Hz, *O*-H); 5.40 (1H, d, *J* = 5.45 Hz, *O*-H); 4.73 (2H, m, CH_2_); 4.21 (1H, m, CH); 4.09 (1H, m, CH); 4.02 (1H, m, CH); 3.79 (6H, s, 2 CH_3_). ^13^C-NMR (100 MHz, DMSO-d_6_) δ (ppm): 163.0, 160.9, 150.6, 146.3, 141.2, 132.5, 122.5, 103.1, 102.0, 100.0, 88.9, 81.6, 72.1, 70.5, 55.3, 51.4. ESI-HRMS calculated for [C_19_H_22_N_5_O_7_]^+^ *m*/*z* 432.1513 and found *m*/*z* 432.1512. IR (neat, cm^−1^): 3233.9*br*; 2943.7*w*; 2840.0*w*; 1682.2*s*; 1596.2*s*; 1556.9*w*; 1456.1*m*; 1423.2*m*; 1370.5*m*; 1265.4*m*; 1203.5*m*; 1153.1*m*; 1100.2*m*; 1043.8*m*; 925.4*w*; 809.6*w*; 685.8w. [α]_D_^20^ = + 74.7 (C = 0.04 M, MeOH). LC-MS purity 99.5%, t_R_ = 6.94 min water/methanol (70:30 *vol*/*vol*, 0.1% of formic acid).

*1-((2R,3R,4S,5R)-3,4-Dihydroxy-5-((4-(4-nitrophenyl)-1H-1,2,3-triazol-1-yl)methyl)tetrahydrofuran-2-yl)-pyrimidine-2,4(1H,3H)-dione* (**3m**). According to the general procedure, copper(II) sulfate pentahydrate (18.5 mg, 0.07 mmol, 10 mol%) and sodium ascorbate (88 mg, 0.46 mmol, 0.6 eq) were added to a solution of 5′-azido-5′-deoxyuridine **2** (200 mg, 0.74 mmol, 1.0 eq) and 1-ethynyl-4-nitrophenyl (110 mg, 0.74 mmol, 1.0 eq) with *t*BuOH:H_2_O (10 mL). The reaction mixture was stirred for 17 h at 40 °C. The residue was purified by a flash chromatography (DCM/MeOH 92/8 to 90/10) followed by a second flash chromatography (EtOAc/MeOH 9/1) to give the desired product as white solid (0.15 g, 48%). Rf (DCM/MeOH 9:1) = 0.26. ^1^H-NMR (400 MHz, DMSO-d_6_) δ (ppm): 10.46 (1H, s, *N*-H); 9.11 (1H, s, =CH_triazol_); 8.77 (2H, d, *J* = 8.46 Hz, 2 Ar-H); 8.63 (2H, d, *J* = 8.46 Hz, 2 Ar-H); 7.88 (1H, d, *J* = 8.08 Hz, =CH); 6.27 (1H, m, CH); 5.97 (1H, d, *J* = 8.08 Hz, =CH); 5.35 (2H, m, CH_2_); 5.23 (1H, d, *J* = 4.28 Hz, *O*-H); 5.08 (1H, d, *J* = 5.73 Hz, *O*-H); 4.71-4.85 (3H, m, 3 CH). ^13^C-NMR (100 MHz, DMSO-d_6_) δ (ppm): 162.9, 150.8, 147.3, 145.6, 141.6, 138.1, 126.6, 124.6, 124.2, 102.6, 92.1, 82.1, 73.4, 71.4, 51.9. ESI-HRMS calculated for [C_17_H_17_N_6_O_7_]^+^ *m*/*z* 417.1153 and found *m*/*z* 417.1153. IR (neat, cm^−1^): 3104.2*br*; 2938.7*w*; 1674.4*s*; 1606.1*m*; 1514.7*m*; 1461.2*m*; 1378.7*m*; 1335.0*s*; 1238.2*m*; 1107.0*m*; 1043.4*m*; 853.4*w*; 813.0*w*; 756.0w. [α]_D_^20^ = + 57.7 (C = 0.02 M, MeOH). LC-MS purity 99.5%, t_R_ = 6.49 min water/methanol (70:30 *vol*/*vol*, 0.1% of formic acid).

*1-((2R,3R,4S,5R)-3,4-Dihydroxy-5-((4-(pyridin-2-yl)-1H-1,2,3-triazol-1-yl)methyl)tetrahydrofuran-2-yl)-pyrimidine-2,4(1H,3H)-dione* (**3n**). According to the general procedure, copper(II) sulfate pentahydrate (18.5 mg, 0.07 mmol, 10 mol%) and sodium ascorbate (88 mg, 0.46 mmol, 0.6 eq) were added to a solution of 5′-azido-5′-deoxyuridine **2** (200 mg, 0.74 mmol, 1.0 eq) and 2-ethynylpyridine (0.11 mL, 1.11 mmol, 1.5 eq) with *t*BuOH:H_2_O (10 mL). The reaction mixture was stirred for 19 h at 40 °C. The residue was purified by a flash chromatography (DCM/MeOH 9/1) followed by a second flash chromatography (EtOAc/MeOH 95/5 to 90/10) to give the desired product as white solid (0.14 g, 50%). Rf (DCM/MeOH 9:1) = 0.15. ^1^H-NMR (400 MHz, DMSO-d_6_) δ (ppm): 11.36 (1H, d, *J* = 2.05 Hz, *N*-H); 8.58 (1H, d, *J* = 4.71 Hz, Ar-H); 8.56 (1H, s, =CH_triazol_); 8.03 (1H, d, *J* = 7.77 Hz, Ar-H); 7.89 (1H, td, *J* = 7.67 Hz, *J* = 1.75 Hz, Ar-H); 7.60 (1H, d, *J* = 8.06 Hz, =CH); 7.34 (1H, dd, *J* = 1.15 Hz, *J* = 4.86 Hz, Ar-H); 5.76 (1H, d, *J* = 5.57 Hz, CH); 5.60 (1H, dd, *J* = 2.05 Hz, *J* = 8.06 Hz, =CH); 5.51 (1H, d, *J* = 5.67 Hz, *O*-H); 5.40 (1H, d, *J* = 5.30 Hz, *O*-H); 4.77 (2H, m, CH_2_); 4.23 (1H, q, *J* = 4.39 Hz, CH); 4.11 (1H, q, *J* = 5.52 Hz, CH); 4.02 (1H, q, *J* = 5.06 Hz, CH). ^13^C-NMR (100 MHz, DMSO-d_6_) δ (ppm): 162.9, 150.6, 149.8, 149.6, 147.3, 141.1, 137.2, 124.0, 123.0, 119.4, 102.0, 88.7, 81.7, 72.0, 70.6, 51.4. ESI-HRMS calculated for [C_16_H_17_N_6_O_5_]^+^ *m*/*z* 373.1254 and found *m*/*z* 373.1254. IR (neat, cm^−1^): 3527.6*w*; 3433.8*m*; 3139.4*w*; 3067.3*w*; 2929.8*w*; 1764.0*w*; 1710.2*s*; 1686.9*s*; 1613.8*s*; 1555.9*w*; 1488.4*w*; 1464.3*m*; 1423.5*w*; 1379.6*w*; 1350.0*w*; 1325.4*w*; 1279.6*w*; 1250.8*m*; 1211.3*w*; 1191.3*w*; 1104.3*w*; 1062.1*m*; 1045.7*w*; 1027.9*w*; 943.4*w*; 898.5*w*; 859.7*w*; 814.5*w*; 788.3*w*; 717.7*w*; 628.5w. [α]_D_^20^ = + 46.4 (C = 0.04 M, MeOH). LC-MS purity 95.8%, t_R_ = 4.33 min water/methanol (70:30 *vol*/*vol*, 0.1% of formic acid).

*1-((2R,3R,4S,5R)-3,4-Dihydroxy-5-((4-(thiophen-2-yl)-1H-1,2,3-triazol-1-yl)methyl)tetrahydrofuran-2-yl)-pyrimidine-2,4(1H,3H)-dione* (**3o**). According to the general procedure, copper(II) sulfate pentahydrate (18.5 mg, 0.07 mmol, 10 mol%) and sodium ascorbate (88 mg, 0.46 mmol, 0.6 eq) were added to a solution of 5′-azido-5′-deoxy-uridine **2** (200 mg, 0.74 mmol, 1.0 eq) and 2-ethynylthiophene (0.18 mL, 1.86 mmol, 2.5 eq) with *t*BuOH:H_2_O (10 mL). The reaction mixture was stirred for 19 h at 40 °C. The residue was purified by a flash chromatography (DCM/MeOH 9/1) followed by a second flash chromatography (EtOAc/MeOH 95/5) to give the desired product as white solid (0.20 g, 71%). Rf (DCM/MeOH 9:1) = 0.26. ^1^H-NMR (400 MHz, DMSO-d_6_) δ (ppm): 11.36 (1H, d, *J* = 2.20 Hz, *N*-H); 8.46 (1H, s, =CH_triazol_); 7.54 (1H, d, *J* = 8.15 Hz, =CH); 7.53 (1H, dd, *J* = 1.17 Hz, *J* = 5.15 Hz, Ar-H); 7.43 (1H, dd, *J* = 3.55 Hz, *J* = 1.18 Hz, Ar-H); 7.12 (1H, dd, *J* = 3.56 Hz, *J* = 5.05 Hz, Ar-H); 5.76 (1H, d, *J* = 5.33 Hz, CH); 5.59 (1H, dd, *J* = 2.20 Hz, *J* = 8.15 Hz, =CH); 5.51 (1H, d, *J* = 5.65 Hz, *O*-H); 5.39 (1H, d, *J* = 5.46 Hz, *O*-H); 4.72 (2H, m, CH_2_); 4.20 (1H, q, *J* = 4.42 Hz, CH); 4.10 (1H, q, *J* = 5.45 Hz, CH); 4.00 (1H, q, *J* = 5.10 Hz, CH). ^13^C-NMR (100 MHz, DMSO-d_6_) δ (ppm): 162.9, 150.6, 141.8, 141.1, 132.8, 127.9, 125.4, 124.2, 121.4, 102.0, 88.9, 81.6, 72.1, 70.5, 51.4. ESI-HRMS calculated for [C_15_H_16_N_5_O_5_S]^+^ *m*/*z* 378.0866 and found *m*/*z* 378.0866. IR (neat, cm^−1^): 3280.9*br*; 3108.2*m*; 2951.7*w*; 2900.9*w*; 1675.3*s*; 1634.1*m*; 1461.2*m*; 1418.2*w*; 1391.3*w*; 1356.5*w*; 1275.5*m*; 1226.6*w*; 1210.1*w*; 1138.1*w*; 1102.6*m*; 1064.0*m*; 1040.9*w*; 996.7*w*; 935.7*w*; 823.2*w*; 685.7w. [α]_D_^20^ = −24.3 (C = 0.04 M, MeOH). LC-MS purity 99.5%, t_R_ = 5.84 min water/methanol (70:30 *vol*/*vol*, 0.1% of formic acid).

*(2R,3R,4S,5R,6R)-2-(Acetoxymethyl)-6-((1-(((2R,3S,4R,5R)-5-(2,4-dioxo-3,4-dihydropyrimidin-1(2H)-yl)-3,4-dihydroxytetrahydrofuran-2-yl)methyl)-1H-1,2,3-triazol-4-yl)methoxy)tetrahydro-2H-pyran-3,4,5-triyl triacetate* (**3p**). According to the general procedure, copper(II) sulfate pentahydrate (28 mg, 0.11 mmol, 10 mol%) and sodium ascorbate (132 mg, 0.67 mmol, 0.6 eq) were added to a solution of 5′-azido-5′-deoxyuridine **2** (300 mg, 1.11 mmol, 1.0 eq) and 2-propynyl-tetra-O-acetyl-β-D-glucopyronoside (430 mg, 1.11 mmol, 1.0 eq) with *t*BuOH:H_2_O (15 mL). The reaction mixture was stirred for 6 h at 25 °C. The residue was purified by a flash chromatography (DCM/MeOH 93/7 to 90/10) followed by a second flash chromatography (acetone/MeOH 99/1) to give the desired product as white solid (0.44 g, 60%). Rf (DCM/MeOH 9:1) = 0.41. ^1^H-NMR (400 MHz, DMSO-d_6_) δ (ppm): 11.35 (1H, s, *N*-H); 8.03 (1H, s, =CH_triazol_); 7.54 (1H, d, *J* = 8.13 Hz, =CH); 5.74 (1H, d, *J* = 5.32 Hz, CH); 5.64 (1H, d, *J* = 8.08 Hz, =CH); 5.50 (1H, d, *J* = 5.47 Hz, *O*-H); 5.38 (1H, d, *J* = 5.28 Hz, *O*-H); 5.25 (1H, t, *J* = 9.49 Hz, CH); 4.87-4.95 (2H, m, 2 CH); 4.60-4.83 (5H, m, 2 CH_2_, 1 CH); 3.95-4.23 (6H, m, 1 CH_2_, 4 CH); 2.03 (3H, s, CH_3_); 1.98 (3H, s, CH_3_); 1.92 (3H, s, CH_3_); 1.90 (3H, s, CH_3_). ^13^C-NMR (100 MHz, DMSO-d_6_) δ (ppm): 170.1, 169.5, 169.3, 169.0, 163.0, 150.6, 143.1, 141.2, 124.9, 102.1, 98.7, 88.9, 81.7, 72.0, 70.9, 70.7, 70.6, 68.1, 61.9, 61.7, 51.3, 20.5, 20.4, 20.3. ESI-HRMS calculated for [C_26_H_34_N_5_O_15_]^+^ *m*/*z* 656.2045 and found *m*/*z* 656.2044. IR (neat, cm^−1^): 3271.1*br*; 2954.3*w*; 1744.8*s*; 1688.5*s*; 1458.2*w*; 1429.5*w*; 1368.8*m*; 1217.4*s*; 1168.6*w*; 1034.9*s*; 905.3*w*; 813.4w. [α]_D_^20^ = + 4.5 (C = 0.02 M, MeOH). LC-MS purity 95.3%, t_R_ = 5.96 min water/methanol (70:30 *vol*/*vol*, 0.1% of formic acid).

*(5-(1-(((2R,3S,4R,5R)-5-(2,4-Dioxo-3,4-dihydropyrimidin-1(2H)-yl)-3,4-dihydroxytetrahydrofuran-2-yl)-methyl)-1H-1,2,3-triazol-4-yl)pentanoyl)-L-alanine* (**4**). According to the general procedure, copper(II) sulfate pentahydrate (23 mg, 0.09 mmol, 10 mol%) and sodium ascorbate (112 mg, 0.56 mmol, 0.6 eq) were added to a solution of 5′-azido-5′-deoxyuridine **2** (254 mg, 0.94 mmol, 1.0 eq) and 6-heptynoic acid (132 mg, 0.94 mmol, 1.0 eq) with *t*BuOH:H_2_O (8 mL). The reaction mixture was stirred for 1 h at 40 °C. The residue was purified by a flash chromatography (H_2_O/^i^PrOH/EtOAc 1:6:3) to give the desired product as white solid. The pure compound was dried under reduced pressure to remove residual water. Then, in a flame-dried round-bottom flask, the corresponding carboxylic acid derivative **3c** (373 mg, 0.94 mmol, 1.0 eq) was stirred with anhydrous DMF (10 mL) under argon atmosphere at 0 °C. Then, BOP reagent (417 mg, 0.94 mmol, 1.0 eq), HOBt (127 mg, 0.94 mmol, 1.0 eq), DIPEA (0.43 mL, 2.46 mmol, 3.0 eq) and molecular sieves 4 Å (one spatula) were successively added and stirred for 10 min at 0 °C. Thus, L-alanine methyl ester hydrochloride (107 mg, 1.04 eq, 1.1 eq) was added to the reaction mixture and stirred at 25 °C for 16 h. The reaction was stopped and concentrated to dryness in vacuo with several co-evaporation with n-heptane (3 × 15 mL) to remove residual DMF. The residue was purified by flash chromatography (H_2_O/^I^PrOH/EA 0.1/6.9/3) followed by a second flash chromatography (acetone/MeOH 96/4 to 90/10) to give a white solid. Then, the desired compound was directly deprotected without further characterization. This peptidic compound (40 mg, 0.08 mmol, 1.0 eq) was stirred with THF:H_2_O 3:1 and lithium hydroxide monohydrate (7 mg, 0.17 mmol, 2.0 eq) was added to the reaction which was stirred for 18 h at 25 °C. Then, the reaction was quenched with 1M HCl aqueous solution to pH 2. The reaction mixture was concentrated to dryness in vacuo. Then, the residue was purified by a flash chromatography SiO_2_-C_18_ (H_2_O 100%) to give the desired product as white solid (27 mg, 29% in three steps). Rf (SiO_2_-C_18_-H_2_O 100%) = 0.18. ^1^H-NMR (400 MHz, DMSO-d_6_) δ (ppm): 11.34 (1H, d, *J* = 1.70 Hz, *N*-H); 8.14 (1H, d, *J* = 7.25 Hz, *N*-H); 7.83 (1H, s, =CH_triazol_); 7.55 (1H, d, *J* = 8.08 Hz, =CH); 5.73 (1H, d, *J* = 5.36 Hz, CH); 5.60 (1H, dd, *J* = 1.70 Hz, *J* = 8.08 Hz, =CH); 4.64 (2H, m, CH_2_); 4.10-4.22 (2H, m, 2 CH); 4.06 (1H, t, *J* = 5.38 Hz, CH); 3.96 (1H, t, *J* = 4.92 Hz, CH); 2.59 (2H, t, *J* = 6.84 Hz, CH_2_); 2.12 (2H, t, *J* = 7.06 Hz, CH_2_); 1.45-1.61 (4H, m, 2 CH_2_); 1.23 (3H, d, *J* = 7.31 Hz, CH_3_). ^13^C-NMR (100 MHz, DMSO-d_6_) δ (ppm): 174.2, 171.9, 163.0, 150.6, 146.8, 141.2, 122.6, 102.0, 88.7, 81.8, 72.1, 70.5, 51.1, 47.4, 34.7, 28.5, 24.8, 24.7, 17.2. ESI-HRMS calculated for [C_19_H_27_N_6_O_8_]^+^ *m*/*z* 467.1884 and found *m*/*z* 467.1882. IR (neat, cm^−1^): 3366.2*br*; 3079.2*w*; 2942.8*w*; 2867.3*w*; 1686.6*m*; 1551.7*w*; 1460.3*w*; 1415.8*w*; 1384.4*w*; 1261.4*w*; 1217.1*w*; 1129.9*w*; 1102.5*w*; 1050.0*s*; 1022.8*s*; 998.9*s*; 825.5*m*; 764.6*m*; 630.7w. [α]_D_^20^ = + 20.6 (C = 0.01 M, MeOH). LC-MS purity 94.9%, t_R_ = 4.37 min water/methanol (70:30 *vol*/*vol*, 0.1% of formic acid).

*N-((2S,3R,4R,5S,6R)-4,5-Dihydroxy-6-(hydroxymethyl)-2-(prop-2-yn-1-yloxy)tetrahydro-2H-pyran-3-yl)acetamide* (**6**, CAS: 1000593-95-2). To a suspension of commercially *N*-acetyl-D-glucosamine **5** (1.0 g, 4.52 mmol, 1.0 eq) in propargylic alcohol (10 mL), 4 Å molecular sieves (one spatula) was added under argon atmosphere. At 0 °C, trimetylsilyl triflate (0.82 mL, 4.52 mmol, 1.0 eq) was added drop by drop and the mixture was stirred at 0 °C for an additional 5 min (until the fully solubilization of starting material). Then, the solution was stirred at 80 °C for 3 h. Thus, the reaction was cooled at 0 °C and quenched with addition of solid NaHCO_3_ (3 g). The suspension was filtered through a Celite pad and this was washed with methanol several times. The filtrate was concentrated to dryness in vacuo and the resulting residue was directly purified by a flash chromatography (DCM/MeOH 9/1) to give a brown solid. Then, the purified residue was dispersed with acetone (10 mL) and this was stirred at 50 °C for 1 h. After, the suspension was cooled at 0 °C for 1 h and this was filtrated and washed with cold acetone (twice) to give a clean white solid (0.61 g, 52%). Rf (DCM/MeOH 9:1) = 0.17. ^1^H-NMR (400 MHz, DMSO-d_6_) δ (ppm): 7.75 (1H, d, *J* = 8.25 Hz, *N*-H); 5.01 (1H, d, *J* = 5.62 Hz, *O*-H); 4.81 (1H, d, *J* = 3.54 Hz, CH); 4.73 (1H, d, *J* = 5.74 Hz, *O*-H); 4.51 (1H, t, *J* = 6.02 Hz, *O*-H); 4.18 (2H, dd, *J* = 2.44 Hz, *J* = 15.95 Hz, CH_2_); 3.60-3.73 (2H, m, 2 CH); 3.43-3.50 (2H, m, 2 CH); 3.42 (1H, t, *J* = 2.39 Hz, CH_alkyne_); 3.32-3.37 (1H, m, CH); 3.11-3.18 (1H, m, CH); 1.83 (3H, s, CH_3_). ^13^C-NMR (100 MHz, DMSO-d_6_) δ (ppm): 169.4, 95.2, 79.7, 77.3, 73.2, 70.7, 70.5, 60.6, 53.4, 22.6. ESI-HRMS calculated for [C_11_H_18_NO_6_]^+^ *m*/*z* 260.1128 and found *m*/*z* 260.1126. IR (neat, cm^−1^): 3585.7*w*; 3284.0*s*; 3079.5*w*; 2921.1*w*; 1632.5*s*; 1546.7*s*; 1412.9*w*; 1378.4*w*; 1320.1*w*; 1248.6*w*; 1113.0*m*; 1094.9*m*; 1029.0*s*; 1000.8*s*; 950.0*w*; 886.8*w*; 845.7*w*; 663.6*w*. [α]_D_^20^ = + 186.6 (C = 0.06 M, MeOH).LC-MS purity 96.6%, t_R_ = 2.99 min water/methanol (70:30 *vol*/*vol*, 0.1% of formic acid).

*N-((2S,3R,4R,5S,6R)-2-((1-(((2R,3S,4R,5R)-5-(2,4-Dioxo-3,4-dihydropyrimidin-1(2H)-yl)-3,4-dihydroxy-tetrahydrofuran-2-yl)methyl)-1H-1,2,3-triazol-4-yl)methoxy)-4,5-dihydroxy-6-(hydroxymethyl)tetrahydro-2H-pyran-3-yl)acetamide *(**7,** CAS: 1152423-06-7). According to the general procedure, copper(II) sulfate pentahydrate (19.3 mg, 0.08 mmol, 10 mol%) and sodium ascorbate (91.7 mg, 0.46 mmol, 0.6 eq) were added to a solution of 5′-azido-5′-deoxyuridine **2** (208 mg, 0.77 mmol, 1.0 eq) and 2-propynyl-*N*-acetyl-*α*-D-glucosamine **6** (200 mg, 0.77 mmol, 1.0 eq) with *t*BuOH:H_2_O (15 mL). The reaction mixture was stirred for 4 h at 40 °C. The residue was purified by a flash chromatography (acetone/H_2_O 95/5) followed by a second flash chromatography (H_2_O/^i^PrOH/EtOAc 1/6/3) to give the desired product as white solid (0.24 g, 59%). Rf (H_2_O/^i^PrOH/EtOAc 1/6/3) = 0.28. ^1^H-NMR (400 MHz, DMSO-d_6_) δ (ppm): 11.36 (1H, s, *N*-H); 8.08 (1H, s, =CH_tosyl_); 7.68 (1H, d, *J* = 8.17 Hz, *N*-H); 7.58 (1H, d, *J* = 8.09 Hz, =CH); 5.74 (1H, d, *J* = 5.38 Hz, CH); 5.65 (1H, d, *J* = 8.09 Hz, =CH); 5.51 (1H, d, *J* = 5.64 Hz, *O*-H); 5.40 (1H, d, *J* = 5.38 Hz, *O*-H); 4.99 (1H, d, J_H19′_ = 5.34 Hz, *O*-H); 4.75 (1H, d, *J* = 3.57 Hz, CH); 4.60–4.74 (4H, m, *O*-H/CH_2_/CH); 4.46-4.54 (2H, m, *O*-H/CH); 4.06-4.17 (2H, m, 2 CH); 3.97 (1H, m, CH); 3.61-3.69 (2H, m, 2 CH); 3.41-3.53 (3H, m, 3 CH); 3.14 (1H, m, CH); 1.78 (3H, s, CH_3_). ^13^C-NMR (100 MHz, DMSO-d_6_) δ (ppm): 169.4, 163.0, 150.6, 143.7, 141.2, 124.6, 102.1, 96.2, 88.9, 81.7, 73.0, 72.0, 70.8, 70.5, 60.8, 59.8, 53.7, 51.3, 22.5. ESI-HRMS calculated for [C_20_H_29_N_6_O_11_]^+^ *m*/*z* 529.1888 and found *m*/*z* 529.1883. IR (neat, cm^−1^): 3307.1*br*; 3099.6*m*; 2929.3*m*; 2833.5*w*; 1674.1*s*; 1548.6*w*; 1462.6*w*; 1423.5*w*; 1380.9*w*; 1320.9*w*; 1263.0*w*; 1101.1*m*; 1019.8*s*; 951.2*w*; 897.4*w*; 814.1*w*; 765.3*w*; 708.8w. [α]_D_^20^ = + 152.0 (C = 0.03 M, H_2_O).LC-MS purity 95.5%, t_R_ = 3.39 min water/methanol (70:30 *vol*/*vol*, 0.1% of formic acid).

*((2R,3S,4R,5R,6S)-5-Acetamido-3,4-dihydroxy-6-(prop-2-yn-1-yloxy)tetrahydro-2H-pyran-2-yl)methyl4-methylbenzenesulfonate* (**8**, CAS: 1895096-35-1). To a solution of 2-propynyl-*N*-acetyl-α-D-glucosamine **6** (1.0 g, 3.86 mmol, 1.0 eq) in adry pyridine (15 mL) at 0 °C under argon atmosphere, Tosyl chloride (0.81 g, 4.24 mmol, 1.1 eq) was added and the reaction was stirred at 25 °C for 16 h. After total conversion of starting material, the reaction mixture was stopped and concentrated to dryness in vacuo with co-evaporation once with toluene (50 mL) and once with cyclohexane (50 mL). The resulting residue was directly purified by a flash chromatography (DCM/MeOH 95/5) followed by a second chromatography (EtOAc 100%) to give a white solid (0.79 g, 50%). Rf (DCM/MeOH 9:1) = 0.38. ^1^H-NMR (400 MHz, DMSO-d_6_) δ (ppm): 7.78 (2H, d, *J* = 8.32 Hz, 2 Ar-H); 7.77 (1H, m, *N*-H); 7.48 (2H, d, *J* = 8.32 Hz, 2 Ar-H); 5.31 (1H, d, *J* = 5.93 Hz, *O*-H); 4.86 (1H, d, *J* = 6.06 Hz, *O*-H); 4.74 (1H, d, *J* = 3.55 Hz, CH); 4.18 (2H, dd, *J* = 1.92 Hz, *J* = 10.75 Hz, CH_2_); 4.11 (2H, Dd, *J* = 2.44 Hz, *J* = 16.11 Hz, CH_2_); 3.65 (1H, m, CH); 3.55 (1H, m, CH); 3.44 (1H, t, *J* = 2.43 Hz, C-H_alkyne_); 3.41 (1H, m, CH); 3.11 (1H, m, CH); 2.43 (3H, s, CH_3_); 1.82 (3H, s, CH_3_). ^13^C-NMR (100 MHz, DMSO-d_6_) δ (ppm):169.4, 144.9, 132.4, 130.1, 127.6, 95.4, 79.4, 77.5, 70.1, 70.0, 69.9, 53.9, 53.2, 22.5, 21.1. ESI-HRMS calculated for [C_18_H_24_NO_8_S]^+^ *m*/*z* 414.1217 and found *m*/*z* 414.1219. IR (neat, cm^−1^): 3282.0*br*; 2921.7*w*; 1651.6*m*; 1597.1*w*; 1538.1*m*; 1441.4*w*; 1353.7*m*; 1293.0*w*; 1189.4*w*; 1173.7*s*; 1122.2*w*; 1095.9*w*; 1032.0*m*; 996.2*w*; 969.2*w*; 929.4*w*; 891.5*w*; 813.4*w*; 664.8*w*. [α]_D_^20^ = + 122.2 (C = 0.07 M, MeOH).LC-MS purity 94.3%, t_R_ = 6.88 min water/methanol (70:30 *vol*/*vol*, 0.1% of formic acid).

*((2R,3S,4R,5R,6S)-5-Acetamido-6-((1-(((2R,3S,4R,5R)-5-(2,4-dioxo-3,4-dihydropyrimidin-1(2H)-yl)-3,4-dihydroxytetrahydrofuran-2-yl)methyl)-1H-1,2,3-triazol-4-yl)methoxy)-3,4-dihydroxytetrahydro-2H-pyran-2-yl)methyl 4-methylbenzenesulfonate* (**9**). According to the general procedure, copper(II) sulfate pentahydrate (47 mg, 0.19 mmol, 10 mol%) and sodium ascorbate (220 mg, 1.12 mmol, 0.6 eq) were added to a solution of 5′-azido-5′-deoxyuridine **2** (500 mg, 1.86 mmol, 1.0 eq) and 6-tosylate-2-propynyl-*N*-acetyl-*α*-D-glucosamine **8** (770 mg, 1.86 mmol, 1.0 eq) with *t*BuOH:H_2_O (25 mL). The reaction mixture was stirred for 2 h at 40 °C. The residue was purified by a flash chromatography (acetone/H_2_O 98/2) followed by a second flash chromatography (H_2_O/^i^PrOH/EtOAc 0.5/6.5/3) to give the desired product as colorless oil (1.04 g, 82%). Rf (H_2_O/^i^PrOH/EtOAc 1/6/3) = 0.68. ^1^H-NMR (400 MHz, DMSO-d_6_) δ (ppm): 11.36 (1H, s, *N*-H); 8.06 (1H, s, =CH_triazol_); 7.79 (2H, d, *J* = 8.35 Hz, 2 Ar-H); 7.70 (1H, d, *J* = 8.12 Hz, *N*-H); 7.58 (1H, d, *J* = 8.12 Hz, =CH); 7.47 (2H, d, *J* = 8.35 Hz, 2 Ar-H); 5.74 (1H, d, *J* = 5.31 Hz, CH); 5.64 (1H, dd, *J* = 1.38 Hz, *J* = 8.12 Hz, =CH); 5.51 (1H, d, *J* = 5.63 Hz, *O*-H); 5.40 (1H, d, *J* = 5.44 Hz, *O*-H); 5.30 (1H, d, *J* = 5.84 Hz, *O*-H); 4.83 (1H, d, *J* = 6.01 Hz, *O*-H); 4.70 (1H, d, *J* = 3.39 Hz, CH); 4.60–4.74 (2H, m, CH_2_); 4.40–4.60 (2H, m, CH_2_); 4.24 (1H, m, CH); 4.10–4.17 (3H, m, 3 CH); 3.98 (1H, m, CH); 3.57–3.67 (2H, m, 2 CH); 3.36–3.44 (1H, m, CH); 3.06–3.14 (1H, m, CH); 2.41 (3H, s, CH_3_); 1.76 (3H, s, CH_3_). ^13^C-NMR (100 MHz, DMSO-d_6_) δ (ppm): 169.5, 163.0, 150.6, 144.9, 143.5, 141.2, 132.4, 130.1, 127.6, 124.6, 102.1, 96.2, 88.9, 81.8, 72.0, 70.6, 70.2, 70.0, 69.8, 60.1, 53.4, 51.4, 22.5, 21.1. ESI-HRMS calculated for [C_27_H_35_N_6_O_13_S]^+^ *m*/*z* 683.1977 and found *m*/*z* 683.1963. IR (neat, cm^−1^): 3303.8*br*; 2929.2*m*; 2832.7*w*; 1682.4*s*; 1598.0*w*; 1548.6*w*; 1461.8*w*; 1427.3*w*; 1379.5*w*; 1351.8*m*; 1265.7*w*; 1189.5*w*; 1174.0*s*; 1123.3*m*; 1096.8*m*; 1020.2*m*; 998.7*m*; 969.6*w*; 931.0*w*; 813.2*w*; 768.8*w*; 666.1w. [α]_D_^20^ = + 91.7 (C = 0.02 M, MeOH).LC-MS purity 98.3%, t_R_ = 5.55 min water/methanol (70:30 *vol*/*vol*, 0.1% of formic acid).

*N-((2S,3R,4R,5S,6R)-6-(Azidomethyl)-2-((1-(((2R,3S,4R,5R)-5-(2,4-dioxo-3,4-dihydropyrimidin-1(2H)-yl)-3,4-dihydroxytetrahydrofuran-2-yl)methyl)-1H-1,2,3-triazol-4-yl)methoxy)-4,5-dihydroxytetrahydro-2H-pyran-3-yl)acetamide *(**10**). To flame dried round-bottom flask, compound **9** (0.91 g, 1.33 mmol, 1.0 eq) was stirred with dry DMF (10 mL) at 25 °C under argon atmosphere. Then, sodium azide (0.26 g, 3.99 mmol, 3.0 eq) was added to the solution and this was stirred for 72 h. Thus, the reaction mixture was concentrated to dryness in vacuo with co-evaporation with n-heptane to remove residual DMF. The residue was purified by a flash chromatography (acetonitrile/H_2_O 9/1) to give the desired product as white solid (0.47 g, 64%). Rf (acetonitrile/H_2_O 9/1) = 0.36. ^1^H-NMR (400 MHz, DMSO-d_6_) δ (ppm): 11.36 (1H, s, *N*-H); 8.08 (1H, s, =CH_triazol_); 7.74 (1H, d, *J* = 8.12 Hz, *N*-H); 7.59 (1H, d, *J* = 8.09 Hz, =CH); 5.74 (1H, d, *J* = 5.37 Hz, CH); 5.65 (1H, d, *J* = 8.09 Hz, =CH); 5.52 (1H, d, *J* = 5.63 Hz, *O*-H); 5.40 (1H, d, *J* = 5.33 Hz, *O*-H); 5.28 (1H, m, *O*-H); 4.82 (1H, d, *J* = 5.97 Hz, *O*-H); 4.80 (1H, d, *J* = 3.54 Hz, CH); 4.61–4.75 (2H, m, CH_2_); 4.51–4.70 (2H, m, CH); 4.09–4.18 (2H, m, 2 CH); 3.98 (1H, m, CH); 3.61–3.72 (2H, m, 2 CH); 3.37–3.50 (3H, m, CH_2_/CH); 3.15 (1H, m, CH); 1.78 (3H, s, CH_3_). ^13^C-NMR (100 MHz, DMSO-d_6_) δ (ppm): 169.5, 163.0, 150.6, 143.5, 141.2, 124.6, 102.1, 96.5, 88.9, 81.7, 72.0, 71.5, 71.4, 70.6, 70.2, 60.2, 53.5, 51.3, 51.1, 22.5. ESI-HRMS calculated for [C_20_H_28_N_9_O_10_]^+^ *m*/*z* 554.1953 and found *m*/*z* 554.1949. IR (neat, cm^−1^): 3289.0*br*; 3258.4*m*; 3193.2*m*; 3151.7*m*; 3077.0*m*; 2929.4*br*; 2722.4*w*; 2595.2*w*; 2101.1*s*; 1682.3*s*; 1577.6*w*; 1529.9*w*; 1546.7*w*; 1463.2*w*; 1421.5*w*; 1381.5*w*; 1318.7*w*; 1267.5*w*; 1234.2*w*; 1121.0*m*; 1098.4*m*; 1053.8*s*; 897.3*w*; 839.9*w*; 815.0*w*; 767.8w. [α]_D_^20^ = + 72.8 (C = 0.01 M, MeOH).LC-MS purity 94.0%, t_R_ = 5.78 min water/methanol (70:30 *vol*/*vol*, 0.1% of formic acid).

*5-(1-(((2R,3S,4R,5R,6S)-5-Acetamido-6-((1-(((2R,3S,4R,5R)-5-(2,4-dioxo-3,4-dihydropyrimidin-1(2H)-yl)-3,4-dihydroxytetrahydrofuran-2-yl)methyl)-1H-1,2,3-triazol-4-yl)methoxy)-3,4-dihydroxytetrahydro-2H-pyran-2-yl)methyl)-1H-1,2,3-triazol-4-yl)pentanoic acid* (**11a**). According to the general procedure, copper(II) sulfate pentahydrate (3.9 mg, 0.02 mmol, 10 mol%) and sodium ascorbate (18.5 mg, 0.09 mmol, 0.6 eq) were added to a solution of compound **10** (86 mg, 0.16 mmol, 1.0 eq) and 6-heptynoic acid (26.1 mg, 0.19 mmol, 1.2 eq) with *t*BuOH:H_2_O (10 mL). The reaction mixture was stirred for 5 h at 40 °C. The reaction was concentrated to dryness in vacuo with co-evaporation of methanol several times. The residue was purified by a flash chromatography (H_2_O/^i^PrOH/EtOAc 1/6/3) to give the desired product as white solid (87 mg, 82%). Rf (H_2_O/^i^PrOH/EtOAc 1/6/3) = 0.23. ^1^H-NMR (400 MHz, DMSO-d_6_) δ (ppm): 11.35 (1H, s, *N*-H); 7.93 (1H, s, =CH_triazol_); 7.84 (1H, s, =CH_triazol_); 7.77 (1H, d, *J* = 8.17 Hz, *N*-H); 7.59 (1H, d, *J* = 8.09 Hz, =CH); 5.73 (1H, d, *J* = 5.43 Hz, CH); 5.64 (1H, d, *J* = 8.09 Hz, =CH); 4.71 (1H, d, *J* = 3.42 Hz, CH); 4.58–4.73 (3H, m, CH_2_/CH); 4.37–4.45 (1H, m, CH); 4.18–4.28 (2H, m, CH_2_); 4.15 (1H, m, CH); 4.06 (1H, t, *J* = 5.38 Hz, CH); 3.93 (1H, m, CH); 3.82 (1H, m, CH); 3.66 (1H, m, CH); 3.47 (1H, m, CH); 3.07 (1H, m, CH); 2.58 (2H, t, = 7.01 Hz, CH_2_); 2.05 (2H, t, *J* = 7.05 Hz, CH_2_); 1.76 (3H, s, CH_3_); 1.41–1.61 (4H, m, 2 CH_2_). ^13^C-NMR (100 MHz, DMSO-d_6_) δ (ppm): 175.7, 169.5, 163.5, 150.6, 146.8, 143.0, 141.2, 124.6, 122.6, 102.1, 95.9, 89.0, 81.8, 72.1, 70.8, 70.6, 70.3, 59.4, 53.5, 51.3, 50.6, 35.0, 28.8, 24.9, 24.7, 22.5,. ESI-HRMS calculated for [C_27_H_38_N_9_O_12_]^+^ *m*/*z* 680.2634 and found *m*/*z* 680.2627. IR (neat, cm^−1^): 3294.2*br*; 3149.3*m*; 2927.8*br*; 2858.9*m*; 1689.3*s*; 1549.6*m*; 1461.5*m*; 1423.2*m*; 1380.5*m*; 1262.9*m*; 1224.5*m*; 1101.7*m*; 1053.1*m*; 1037.5*m*; 896.2*w*; 813.3*w*; 774.4*w*; 720.7w. [α]_D_^20^ = + 44.2 (C = 0.01 M, MeOH).LC-MS purity 96.7%, t_R_ = 2.58 min water/methanol (70:30 *vol*/*vol*, 0.1% of formic acid).

*N-((2S,3R,4R,5S,6R)-6-((4-(Aminomethyl)-1H-1,2,3-triazol-1-yl)methyl)-2-((1-(((2R,3S,4R,5R)-5-(2,4-dioxo-3,4-dihydropyrimidin-1(2H)-yl)-3,4-dihydroxytetrahydrofuran-2-yl)methyl)-1H-1,2,3-triazol-4-yl)methoxy)-4,5-dihydroxytetrahydro-2H-pyran-3-yl)acetamide* (**11b**). According to the general procedure, copper(II) sulfate pentahydrate (3.9 mg, 0.02 mmol, 10 mol%) and sodium ascorbate (18.5 mg, 0.09 mmol, 0.6 eq) were added to a solution of compound **10** (86 mg, 0.16 mmol, 1.0 eq) and propargylamine (12.8 mg, 0.23 mmol, 1.5 eq) with *t*BuOH:H_2_O (10 mL). The reaction mixture was stirred for 5 h at 40 °C. The reaction was concentrated to dryness in vacuo with co-evaporation of methanol several times. The residue was purified by a flash chromatography (Acetone/H_2_O 95/5) to give the desired product as pale yellow solid (18 mg, 19%). Rf (acetone/H_2_O 95/5) = 0.25. ^1^H-NMR (400 MHz, DMSO-d_6_) δ (ppm): 11.34 (1H, s, *N*-H); 8.79 (1H, s, =CH_triazol_); 8.01 (1H, s, =CH_triazol_); 7.74 (1H, d, *J* = 8.13 Hz, *N*-H); 7.58 (1H, d, *J* = 8.10 Hz, =CH); 5.73 (1H, d, *J* = 5.24 Hz, CH); 5.63 (1H, d, *J* = 8.07 Hz, CH); 5.57 (1H, d, *J* = 5.13 Hz, *O*-H); 5.52 (1H, d, *J* = 5.33 Hz, *O*-H); 5.42 (1H, d, *J* = 4.92 Hz, *O*-H); 4.92 (1H, d, *J* = 5.74 Hz, *O*-H); 4.73 (1H, d, *J* = 3.43 Hz, CH); 4.57–4.82 (3H, m, CH_2_/CH); 4.37–4.51 (1H, m, CH); 4.22–4.36 (2H, m, CH_2_); 4.05–4.18 (4H, m, *N*-H_2_/CH/CH); 3.97 (1H, m, CH); 3.88 (1H, m, CH); 3.62 (1H, m, CH); 3.48 (1H, m, CH); 3.17 (1H, d, *J* = 5.03 Hz, CH); 3.05 (1H, m, CH); 1.76 (3H, s, CH_3_). ^13^C-NMR (100 MHz, DMSO-d_6_) δ (ppm): 169.5, 163.0, 150.6, 146.7, 144.1 141.2, 129.3, 124.5, 102.1, 96.4, 89.0, 81.7, 72.0, 71.9, 70.6, 70.1, 59.9, 55.8, 51.3, 48.6, 22.4. ESI-HRMS calculated for [C_23_H_30_N_9_O_11_]^+^ *m*/*z* 608.2059 and found *m*/*z* 608.2053. IR (neat, cm^−1^): 3275.5*br*; 2953.8*m*; 2922.9*s*; 2852.8*m*; 1688.6*s*; 1537.8*w*; 1463.0*w*; 1428.3*w*; 1379.6*w*; 1260.0*w*; 1231.2*w*; 1187.8*w*; 1126.1*w*; 1102.0*m*; 1083.0*m*; 1050.1*s*; 816.0*w*; 776.8*w*; 719.3w. [α]_D_^20^ = + 43.3 (C = 0.03 M, H_2_O).LC-MS purity 95.0%, t_R_ = 2.22 min water/methanol (70:30 *vol*/*vol*, 0.1% of formic acid).

*1-(((2R,3S,4R,5R,6S)-5-Acetamido-6-((1-(((2R,3S,4R,5R)-5-(2,4-dioxo-3,4-dihydropyrimidin-1(2H)-yl)-3,4-dihydroxytetrahydrofuran-2-yl)methyl)-1H-1,2,3-triazol-4-yl)methoxy)-3,4-dihydroxytetrahydro-2H-pyran-2-yl)methyl)-1H-1,2,3-triazole-4-carboxamide* (**11c**). According to the general procedure, copper(II) sulfate pentahydrate (3.9 mg, 0.02 mmol, 10 mol%) and sodium ascorbate (18.5 mg, 0.09 mmol, 0.6 eq) were added to a solution of compound **10** (86 mg, 0.16 mmol, 1.0 eq) and propiolamide (12 mg, 0.17 mmol, 1.1 eq) with *t*BuOH:H_2_O (10 mL). The reaction mixture was stirred for 5 h at 40 °C. The reaction was concentrated to dryness in vacuo with co-evaporation of methanol several times. The residue was purified by a flash chromatography (H_2_O/^i^PrOH/EtOAc 1/6/3) to give the desired product as white solid (87 mg, 90%). Rf (Acetone/H_2_O 95/5) = 0.31. ^1^H-NMR (400 MHz, DMSO-d_6_) δ (ppm): 11.36 (1H, s, *N*-H); 8.45 (1H, s, =CH_triazol_); 8.02 (1H, s, =CH_triazol_); 7.84 (1H, s, *N*-H); 7.75 (1H, d, *J* = 8.06 Hz, *N*-H); 7.59 (1H, d, *J* = 8.06 Hz, =CH); 7.43 (1H, s, *N*-H); 5.74 (1H, d, *J* = 5.32 Hz, CH); 5.65 (1H, d, *J* = 8.04 Hz, =CH); 4.74 (1H, d, *J* = 3.38 Hz, CH); 4.53–4.78 (4H, m, CH/CH_2_); 4.24–4.37 (2H, m, CH_2_); 4.10–4.18 (2H, m, 2 CH); 3.98 (1H, m, CH); 3.89 (1H, m, CH); 3.63 (1H, m, CH); 3.49 (1H, m, CH); 3.04 (1H, t, *J* = 9.22 Hz, CH); 1.76 (3H, s, CH_3_). ^13^C-NMR (100 MHz, DMSO-d_6_) δ (ppm): 169.5, 163.0, 161.6, 150.6, 143.5, 142.7, 141.3, 127.6, 124.5, 102.1, 96.3, 89.0, 81.8, 72.0, 71.9, 70.6, 70.2, 59.9, 53.5, 51.4, 50.9, 22.5. ESI-HRMS calculated for [C_23_H_31_N_10_O_11_]^+^ *m*/*z* 623.2168 and found *m*/*z* 623.2164. IR (neat, cm^−1^): 3304.7*br*; 2922.8*m*; 2851.5*w*; 1667.0*s*; 1556.8*m*; 1463.0*m*; 1423.1*m*; 1382.1*m*; 1322.8*w*; 1263.2*m*; 1232.2*m*; 1101.2*m*; 1049.8*s*; 1019.6*s*; 896.1*w*; 814.8*w*; 773.3w. [α]_D_^20^ = + 62.8 (C = 0.02 M, MeOH).LC-MS purity 96.1%, t_R_ = 2.88 min water/methanol (70:30 *vol*/*vol*, 0.1% of formic acid).

***N**-((1-(((2R,3S,4R,5R,6S)-5-Acetamido-6-((1-(((2R,3S,4R,5R)-5-(2,4-dioxo-3,4-dihydropyrimidin-1(2H)-yl)-3,4-dihydroxytetrahydrofuran-2-yl)methyl)-1H-1,2,3-triazol-4-yl)methoxy)-3,4-dihydroxytetrahydro-2H-pyran-2-yl)methyl)-1H-1,2,3-triazol-4-yl)methyl)octanamide *(**11d**). According to the general procedure, copper(II) sulfate pentahydrate (3.9 mg, 0.02 mmol, 10 mol%) and sodium ascorbate (18.5 mg, 0.09 mmol, 0.6 eq) were added to a solution of compound **10** (86 mg, 0.16 mmol, 1.0 eq) and N-(prop-2-yn-1-yl)octanamide (31 mg, 0.17 mmol, 1.1 eq) with *t*BuOH:H_2_O (10 mL). The reaction mixture was stirred for 5 h at 40 °C. The reaction was concentrated to dryness in vacuo with co-evaporation of methanol several times. The residue was purified by a flash chromatography (acetone/H_2_O 95/5) to give the desired product as white solid (88 mg, 78%). Rf (acetone/H_2_O 95/5) = 0.38. ^1^H-NMR (400 MHz, DMSO-d_6_) δ (ppm): 11.35 (1H, d, *J* = 1.30 Hz, *N*-H); 8.23 (1H, t, *J* = 5.64 Hz, *N*-H); 7.99 (1H, s, =CH_triazol_); 7.84 (1H, s, =CH_triazol_); 7.72 (1H, d, *J* = 8.16 Hz, *N*-H); 7.59 (1H, d, *J* = 8.07 Hz, =CH); 5.74 (1H, d, *J* = 5.35 Hz, CH); 5.64 (1H, dd, *J* = 1.30 Hz, *J* = 8.07 Hz, =CH); 5.51 (1H, d, *J* = 5.63 Hz, *O*-H); 5.47 (1H, d, *J* = 5.79 Hz, *O*-H); 5.40 (1H, d, *J* = 5.43 Hz, *O*-H); 4.88 (1H, d, *J* = 5.79 Hz, *O*-H); 4.71 (1H, d, *J* = 3.57 Hz, CH); 4.60–4.74 (3H, m, CH/CH_2_); 4.44–4.51 (1H, m, CH); 4.22–4.37 (4H, m, 2 CH_2_); 4.07–4.18 (2H, m, 2 CH); 3.98 (1H, m, CH); 3.83 (1H, m, CH); 3.63 (1H, m, CH); 3.47 (1H, m, CH); 3.05 (1H, m, CH); 2.03 (3H, t, *J* = 7.35 Hz, CH_3_); 1.76 (3H, s, H_29′_); 1.44 (2H, m, CH_2_); 1.15–1.28 (8H, m, 4 CH_2_); 0.84 (3H, t, *J* = 6.70 Hz, CH_3_). ^13^C-NMR (100 MHz, DMSO-d_6_) δ (ppm): 172.1, 169.5, 163.0 150.6, 144.9, 143.2, 141.2, 124,6, 123.7, 102.1, 96.1, 88.9, 81.7, 72.0, 71.9, 70.6, 70.5, 70.2, 59.8, 53.4, 51.4, 50.5, 35.2, 34.1, 31.1, 28.6, 28.4, 25.2, 22.5, 22.0, 13.9. ESI-HRMS calculated for [C_31_H_47_N_10_O_11_]^+^ *m*/*z* 735.3420 and found *m*/*z* 735.3421. IR (neat, cm^−1^): 3291.1*br*; 2928.4*s*; 2855.8*m*; 1682.8*s*; 1540.6*m*; 1461.6*m*; 1427.5*m*; 1379.1*m*; 1264.0*m*; 1227.8*m*; 1102.8*m*; 1052.8*s*; 814.2*w*; 776.8*w*; 720.7*w*. [α]_D_^20^ = + 79.0 (C = 0.02 M, MeOH).LC-MS purity 95.2%, t_R_ = 3.42 min water/methanol (70:30 *vol*/*vol*, 0.1% of formic acid).

*N-((2S,3R,4R,5S,6R)-6-((4-((((2S,3R,4R,5S,6R)-3-Acetamido-4,5-dihydroxy-6-(hydroxymethyl)tetrahydro-2H-pyran-2-yl)oxy)methyl)-1H-1,2,3-triazol-1-yl)methyl)-2-((1-(((2R,3S,4R,5R)-5-(2,4-dioxo-3,4-dihydro-pyrimidin-1(2H)-yl)-3,4-dihydroxytetrahydrofuran-2-yl)methyl)-1H-1,2,3-triazol-4-yl)methoxy)-4,5-dihydroxytetrahydro-2H-pyran-3-yl)acetamide* (**11e**). According to the general procedure, copper(II) sulfate pentahydrate (3.9 mg, 0.02 mmol, 10 mol%) and sodium ascorbate (18.5 mg, 0.09 mmol, 0.6 eq) were added to a solution of compound **10** (86 mg, 0.16 mmol, 1.0 eq) and 2-propynyl-*N*-acetyl-*α*-D-glucosamine **6** (44.3 mg, 0.17 mmol, 1.1 eq) with *t*BuOH:H_2_O (10 mL). The reaction mixture was stirred for 5 h at 40 °C. The reaction was concentrated to dryness in vacuo with co-evaporation of methanol several times. The residue was purified by a flash chromatography (acetone/H_2_O 9/1) to give the desired product as white solid (97 mg, 77%). Rf (acetone/H_2_O 9/1) = 0.26. ^1^H-NMR (400 MHz, DMSO-d_6_) δ (ppm): 11.35 (1H, s, *N*-H); 8.04 (1H, s, =CH_triazol_); 8.00 (1H, s, =CH_triazol_); 7.74 (1H, d, *J* = 8.21 Hz, *N*-H); 7.70 (1H, d, *J* = 8.24 Hz, *N*-H); 7.59 (1H, d, *J* = 8.11 Hz, =CH); 5.74 (1H, d, *J* = 5.37 Hz, CH); 5.65 (1H, d, *J* = 8.11 Hz, =CH); 5.53 (1H, m, *O*-H); 5.49 (1H, m, *O*-H); 5.42 (1H, m, *O*-H); 4.99 (1H, m, *O*-H); 4.90 (1H, m, *O*-H); 4.74 (1H, d, *J* = 3.46 Hz, CH); 4.72 (1H, d, *J* = 3.39 Hz, CH); 4.59–4.74 (4H, m, 3 CH); 4.46–4.57 (3H, m, 3 CH); 4.25–4.35 (2H, m, CH_2_); 4.10–4.18 (2H, m, 2 CH); 4.09 (1H, m, *O*-H); 3.98 (1H, m, CH); 3.86 (1H, m, CH); 3.60–3.70 (3H, m, 3 CH); 3.40–3.53 (4H, m, 4 CH); 3.15 (1H, t, *J* = 10.28 Hz, CH); 3.05 (1H, t, *J* = 9.08 Hz, CH); 1.80 (3H, s, CH_3_); 1.76 (3H, s, CH_3_). ^13^C-NMR (100 MHz, DMSO-d_6_) δ (ppm): 169.5, 163.0, 150.6, 143.5, 143.4, 141.2, 125.1, 124.4, 102.1, 96.4, 96.1, 88.9, 81.8, 73.0, 72.0, 71.9, 70.8, 70.6, 70.5, 70.4, 70.3, 60.8, 60.0, 59.8, 53.6, 53.5, 51.4, 50.6, 22.6, 22.5. ESI-HRMS calculated for [C_31_H_45_N_10_O_16_]^+^ *m*/*z* 813.3009 and found *m*/*z* 813.3006. IR (neat, cm^−1^): 3306.8*br*; 2923.4*br*; 1682.6*s*; 1548.2*m*; 1461.6*w*; 1428.0*w*; 1378.4*w*; 1318.5*w*; 1263.6*w*; 1229.9*w*; 1103.2*m*; 1022.6*s*; 949.6*w*; 841.3*w*; 811.3*w*; 759.8*w*. [α]_D_^20^ = + 113.5 (C = 0.01 M, MeOH).LC-MS purity 97.1%, t_R_ = 2.48 min water/methanol (70:30 *vol*/*vol*, 0.1% of formic acid).

### 3.3. Screening of Ligase Inhibitors Using the One-Pot Assay Developed for MurA-F Enzymes of Mycobacterium tuberculosis

The compounds were prepared in DMSO and screened for inhibitory effect on Mur enzyme activity at a concentration of 100 μM using the one pot assay [15]. Briefly, the enzyme mix (MurA-F) was pre-incubated with each inhibitor for 15 min, followed by incubation with UDP-*N*-acetylglucosamine (sugar substrate for MurA). The other reaction components were added as described previously [15] and the reaction mixture was incubated at 37 °C for 30 min. Release of inorganic phosphate (Pi) was measured at 630 nm using a PiColorlock™ Assay kit (Novus Biologicals, New Delhi, India). In the positive control reaction, inhibitor was not added and in the negative control, enzymes were not included and in place of inhibitor, DMSO (5%) was added. Assays were carried out in 96-wells microtiter plates (Tarsons, New Delhi, India) in triplicates.

After identifying the compounds that showed 50% or higher inhibition of the one-pot assay, aim was to identify the target Mtb Mur ligases (MurC, MurD, MurE or MurF) for each compound. For this, coupled assay for Mur enzymes was carried out in a sequential manner as described earlier [15]. Briefly, each of the four Mtb Mur ligases was pre-incubated separately with compound for 15 min and then was added to the total reaction mixture (one at a time) and assayed at 37 °C for 30 min. The Pi released was calculated as described before. Negative control contained all the components except the Mur ligase enzyme. 

### 3.4. In Silico Screening

The compounds showing low IC_50_ were studied for the stability of binding. Molecular docking of the compounds to the Mtb MurE ligase of *Mycobacterium tuberculosis* was done using AutoDock Vina [18]. Autodock Vina has significant speed and accuracy and it also allows ligands to be flexible. The 3D structures of identified compounds were prepared using MarvinSketch (ChemAxon Ltd., Budapest, Hungary), whereas, Mtb MurE structure was retrieved from Protein Data Bank (http://www.ecsb/pdb). 

### 3.5. Protein Structure Preparation

AutoDock Vina required.pdbqt format of input files. MGLTools software was used to prepare the protein/ligand structures for the docking studies. Structure preparation included (i) removal of water, (ii) addition of hydrogen atoms, (iii) Gasteiger charge addition, (iv) merging of non-polar hydrogen atoms, and energy minimization of the structure. The energy minimization was performed using UCSF Chimera program [19]. Prior to energy minimization, Chimera improves the structural inconsistencies namely, addition of hydrogen and charge to the structure and assigning force field parameters to atoms. The Amber force field parameters were used. Steepest descent minimization was performed followed by conjugate gradient minimization, as it is more effective to reach minimum energy [19].

### 3.6. Molecular Dynamic Simulation

The best fitted confirmations of Mtb MurE and ligand complex were subjected to GROMACS (version 4.5.3) [20] to further examine the stability of interaction between Mtb MurE and ligands. The PRODRG sever [21] was used to generate the ligand topology files. Molecular dynamics (MD) simulation was carried out for 40 ns using GROMOS53a6.ff force field. Initially the system was neutralized with sodium ions followed by energy minimization by using steepest descent method in order to calm down the initial solvent along with abolition of the residual strain. The LINCS algorithm was used to constrain the bond lengths within the protein. The temperature of the system was maintained at constant value 300 K by using Berendsen thermostat and the constant pressure at 1 atm was sustained by Parrinello-Rahman method. 

### 3.7. Molecular Docking

Compounds **11c** and **11e** were screened against Mtb MurE (PDB ID 2WTZ, [16]) by using an in-house script for the screening of multiple compounds. A rigid docking was performed by constructing the grid with the 40, 40, 40 dimension and spacing of 0.375 Å. The grid was centered to the active site residues of the Mtb MurE. Binding energy (kcal/mol) and hydrogen bonds between the compounds and the active site residues in Mtb MurE were considered to study the protein-ligand interactions. Cutoff (−9.0 kcal/mol) for binding score was referred from a previous report Eniyan et al. [22]. Vina, by default, predicts nine poses for each ligand. The docked complexes were visualized using the Pymol tool [17].

### 3.8. Trajectory Analysis

The g_rms and g_rmsf modules of GROMACS were used to evaluate the root mean square deviation (RMSD) and root mean square fluctuations (RMSF). The RMSD of backbone atoms of the protein was calculated by considering starting coordinates as reference structure. The hydrogen bond interactions were examined by using g_hbond package in GROMACS.

## 4. Conclusions

In summary, we have synthetized various hitherto unknown 5′-deoxy-5′-(4-substituted-1,2,3-triazol-1-yl) uridine analogues through CuAAC cross-coupling reactions as the key step with the aim of exploring the structure activity relationships for the inhibition of Mur ligases in *Mycobacterium tuberculosis*. Out of all the synthesized compounds, the glucosamine uridine derivatives **11c** and **11e** exhibited an IC_50_ ≥ 50% at 100 µM on the one-pot assay containing *Mycobacterium tuberculosis* MurA-F enzymes. Interestingly, we could identify MurE ligase as the potential target for these compounds (**11c** and **11e** showed selective 41.8% and 48.9% inhibition, respectively). Altogether, these data indicate that both **11c** and **11e** are promising selective inhibitors of Mtb MurE ligase and represent starting material for further modifications. Future work will be focused on the chemical optimization of those compounds to more active inhibitors and to allow their penetration [23] in bacterial cells which, even if it remains elusive [24], represents one of the biggest challenges.

## Figures and Tables

**Table 1:** Results of the inhibition of one-pot assay containing Mtb MurA-F enzymes by compounds **3a**–**p** and **4** at 100 μM.

Cpd	Structure 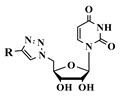 where R is:	Mean Inhibition (%)	% S.D.	Cpd	Structure 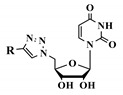 where R is:	Mean inhibition (%)	% S.D.
**3a**	(CH_2_)_2_CO_2_H	11.4	5.8	**3j**	CH_3_(CH_2_)_6_C(O)NHCH_2_	19.6	2.8
**3b**	(CH_2_)_3_CO_2_H	16.9	4.2	**3k**	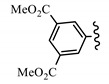	21.0	6.6
**3c**	(CH_2_)_4_CO_2_H	29.4	0.2	**3l**	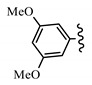	3.1	1.8
**3d**	CH_2_-OH	19.3	0.7	**3m**	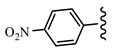	5.0	2.8
**3e**	(CH_2_)_2_-OH	13.6	0.5	**3n**	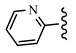	25.0	16.9
**3f**	(CH_2_)_3_-OH	14.8	2.5	**3o**	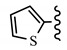	3.1	0
**3g**	(CH_2_)NH_2_	38.7	0.9	**3p**	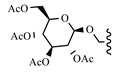	30.0	14.7
**3h**	CH_2_C(O)NH_2_	5.0	2.7	**4**	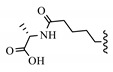	21.0	2.4
**3i**	CH_3_(CH_2_)_4_C(O)NHCH_2_	35.7	0.6				

**Table 2:** Results of the inhibition of one-pot assay containing Mtb MurA-F enzymes by glucosamine derivatives **7** and **11a**–**e** at 100 μM.

Cpd	StruStructure 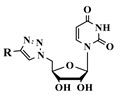 where R is:	Mean inhibition (%)	% S.D.	Cpd	Structure 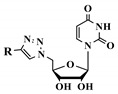 where R is:	Mean inhibition (%)	% S.D.
**7**	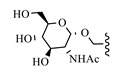	6.0	2.9	**11c**	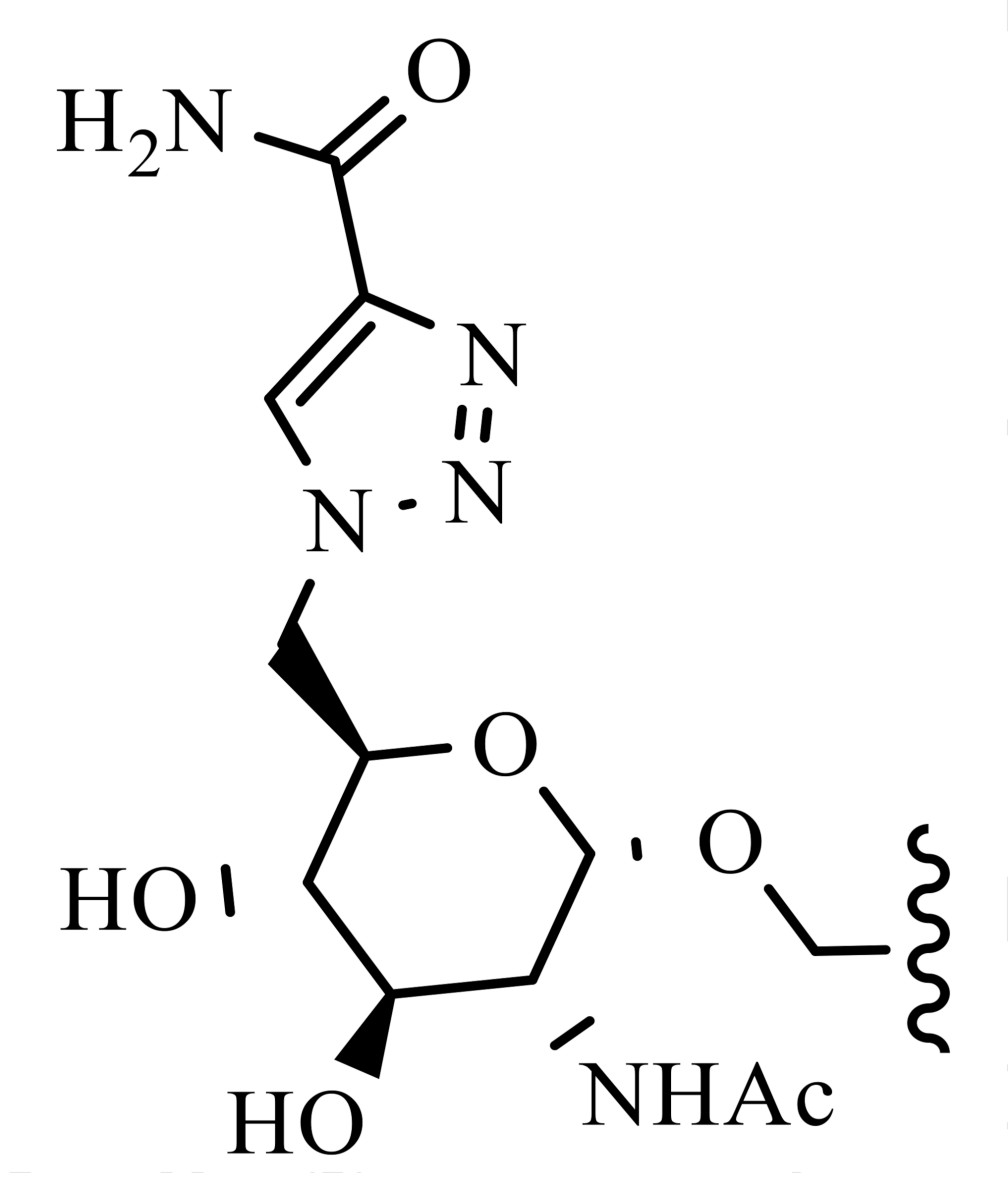	56	1.9
**11a**	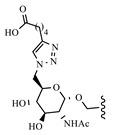	25.1	1.0	**11d**	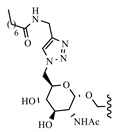	39	1.0
**11b**	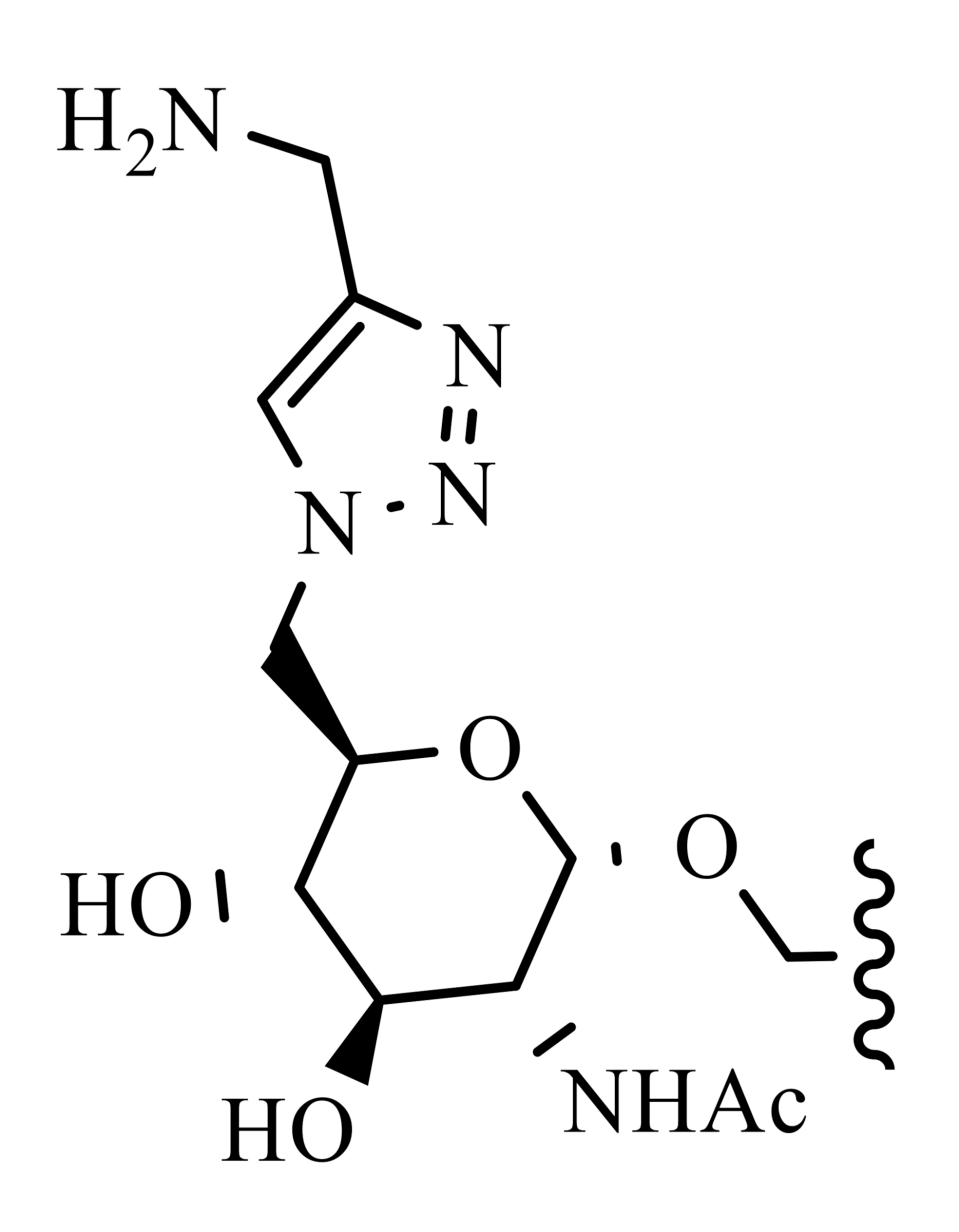	30.1	0.9	**11e**	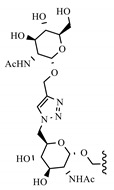	50	2.0

**Table 3:** Inhibitory effect of compound **11c** and **11e** (100 µM) on Mtb MurC-F ligases.

Targets	Cpd	Mean Inhibition (%)	% S.D.
MurC	**11c**	12.1	1.52
	**11e**	1.0	0.89
MurD	**11c**	23.0	1.11
	**11e**	5.2	1.25
MurE	**11c**	41.8	1.42
	**11e**	48.9	3.41
MurF	**11c**	6.1	1.94
	**11e**	12.5	4.59

## Supplementary Materials

^1^H-, ^13^C- and ^1^H-^13^C HMBC NMR spectra of representative molecules (**2**, **3a–p**, **4**, **6–10**, **11a–e**) are available online.
