# Supplementary material for: Design and Synthesis of Various 5′-Deoxy-5′-(4-Substituted-1,2,3-Triazol-1-yl)-Uridine Analogues as Inhibitors of Mycobacterium tuberculosis Mur Ligases
Source: Molecules. 2020 Oct 26;25(21):4953. doi: 10.3390/molecules25214953 (PMC7663697; doi:10.3390/molecules25214953)
Supplement: Supplementary file 1 [file molecules-25-04953-s001.pdf]

Design and synthesis of various 5′-deoxy-5′-(4-substituted-1,2,3-triazol-1-yl)uridine analogues as inhibitors of *Mycobacterium tuberculosis* Mur-ligases

Vincent Hervin [1], Ritu Arora [2], Jyoti Rani [2,3], Srinivasan Ramchandran [3], Urmi Bajpai [2], Luigi A. Agrofoglio [1,*] and Vincent Roy [1,*]

[1] Institute of Organic and Analytical Chemistry, CNRS UMR 7311, Universite d'Orléans, Rue de Chartres, CEDEX 2, 45067 Orleans, France; vincent.hervin@univ-orleans.fr

[2] Department of Biomedical Science, Acharya Narendra Dev College, University of Delhi, New Delhi 110019, India; rituarora1391@gmail.com (R.A.); sharmajyoti829@gmail.com (J.R.); urmibajpai@andc.du.ac.in (U.B.)

[3] G N Ramachandran Knowledge of Centre, Council of Scientific and Industrial Research – Institute of Genomics and Integrative Biology (CSIR-IGIB), Room No. 130, Mathura Road, New Delhi, 110025 India; ramu@igib.res.in

Corresponding authors : vincent.roy@univ-orleans.fr; luigi.agrofoglio@univ-orleans.fr

Supplementary Informations

Copies of ^{1}H-, ^{13}C- and ^{1}H-^{13}C HMBC NMR spectra of representative molecules

^{1}H NMR (DMSO-d6) of compound **2**

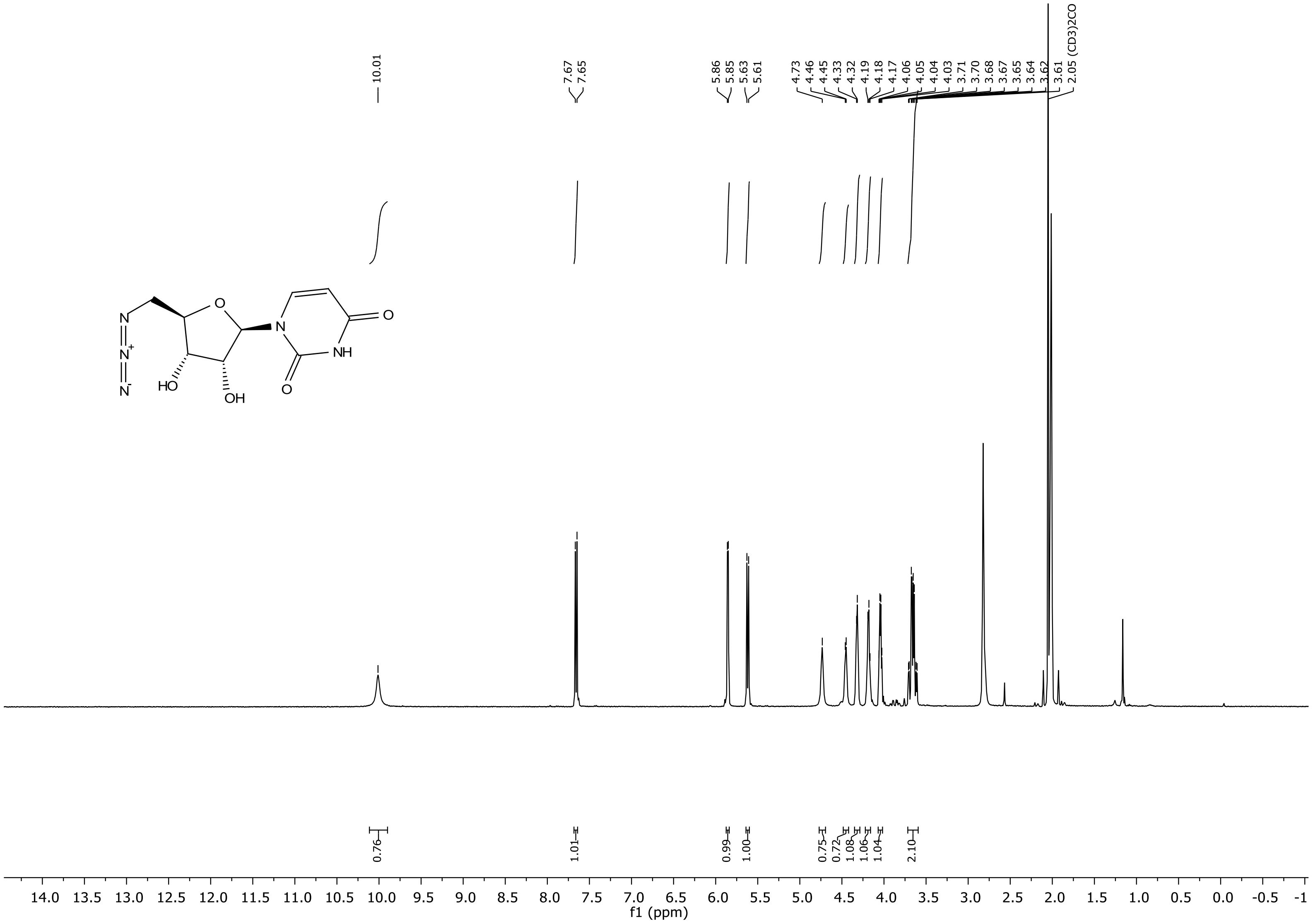

^{13}C NMR (DMSO-d6) of compound **2**

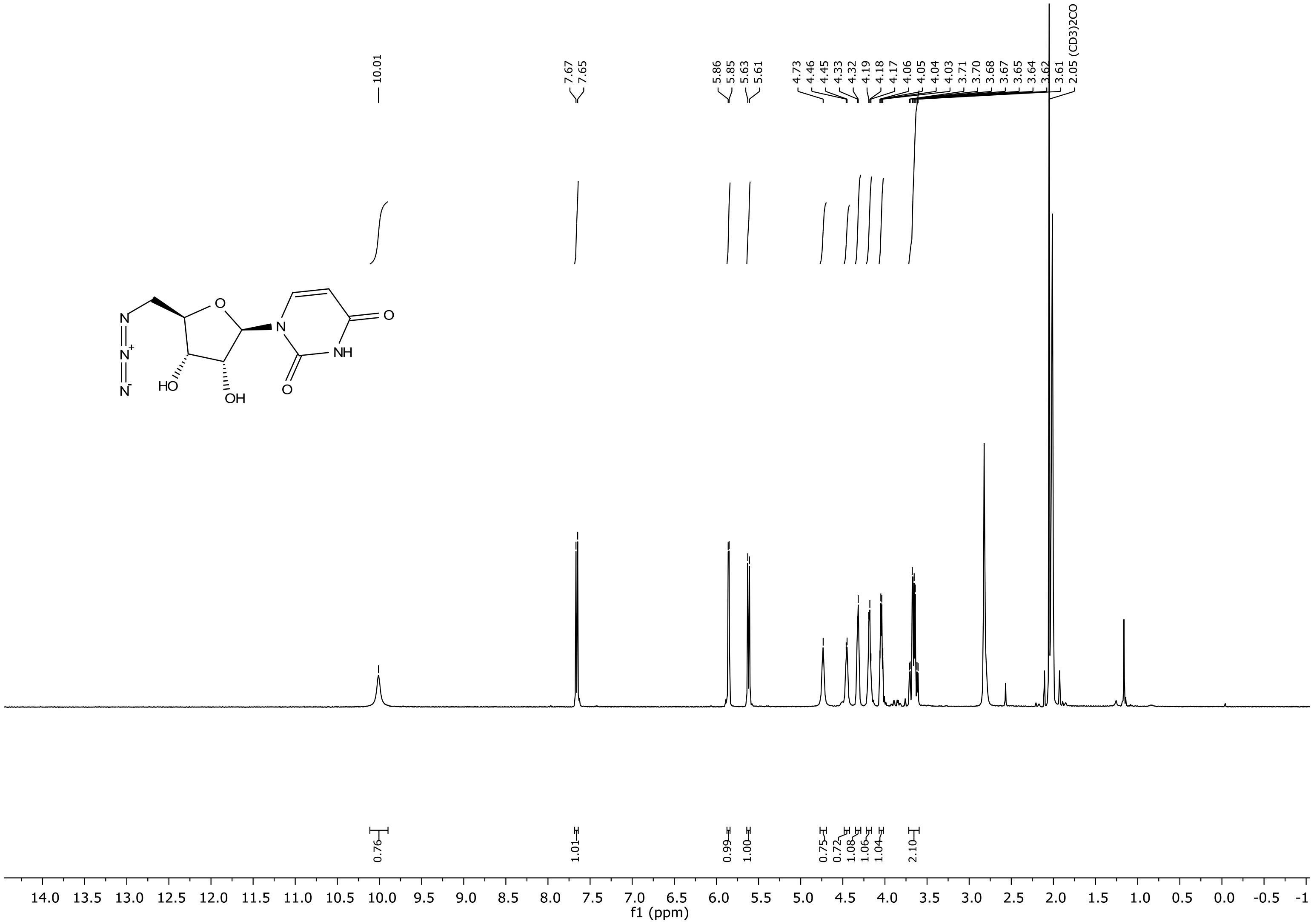

^{1}H NMR (DMSO-d6) of compound **3a**

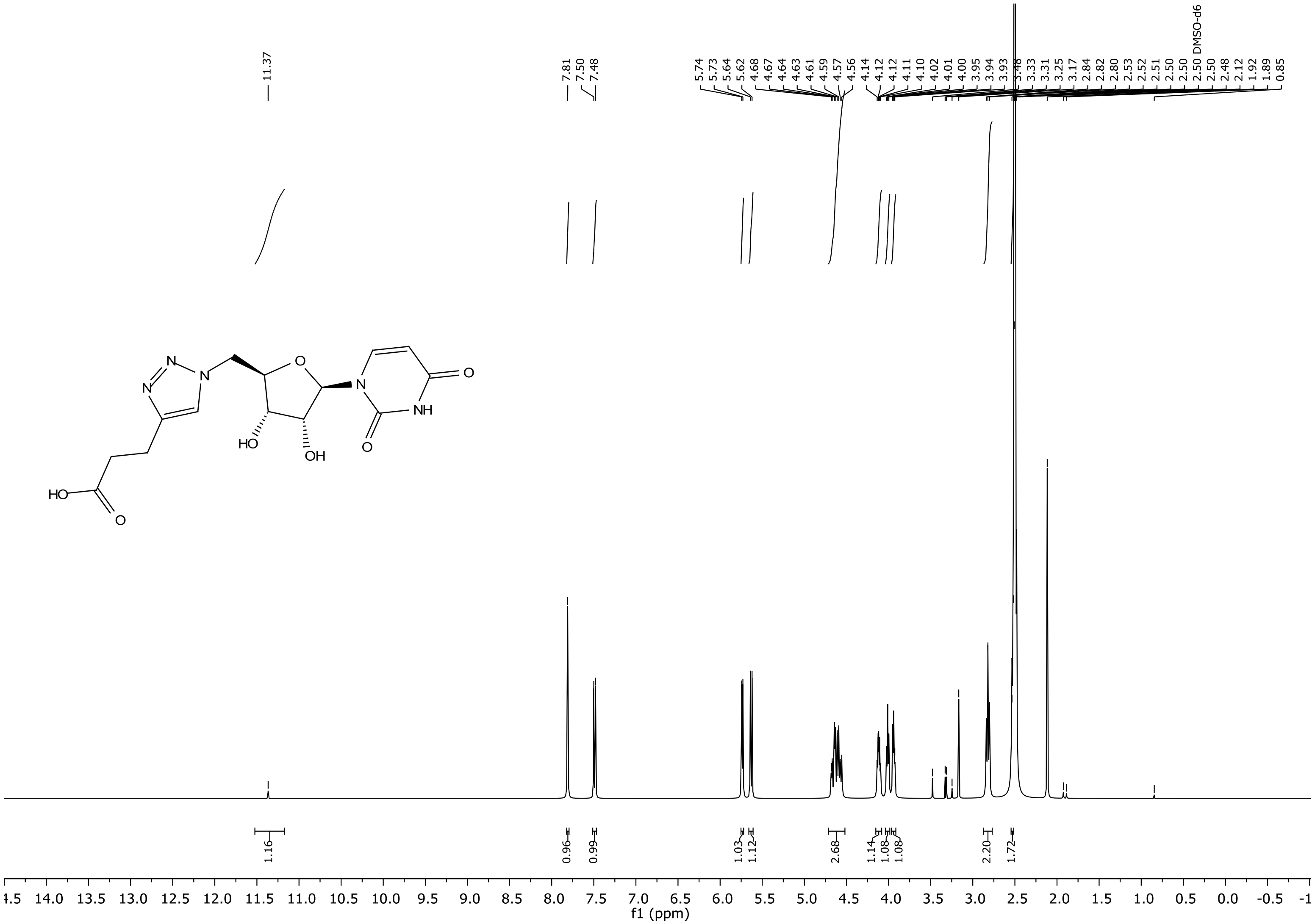

^{13}C NMR (DMSO-d6) of compound **3a**

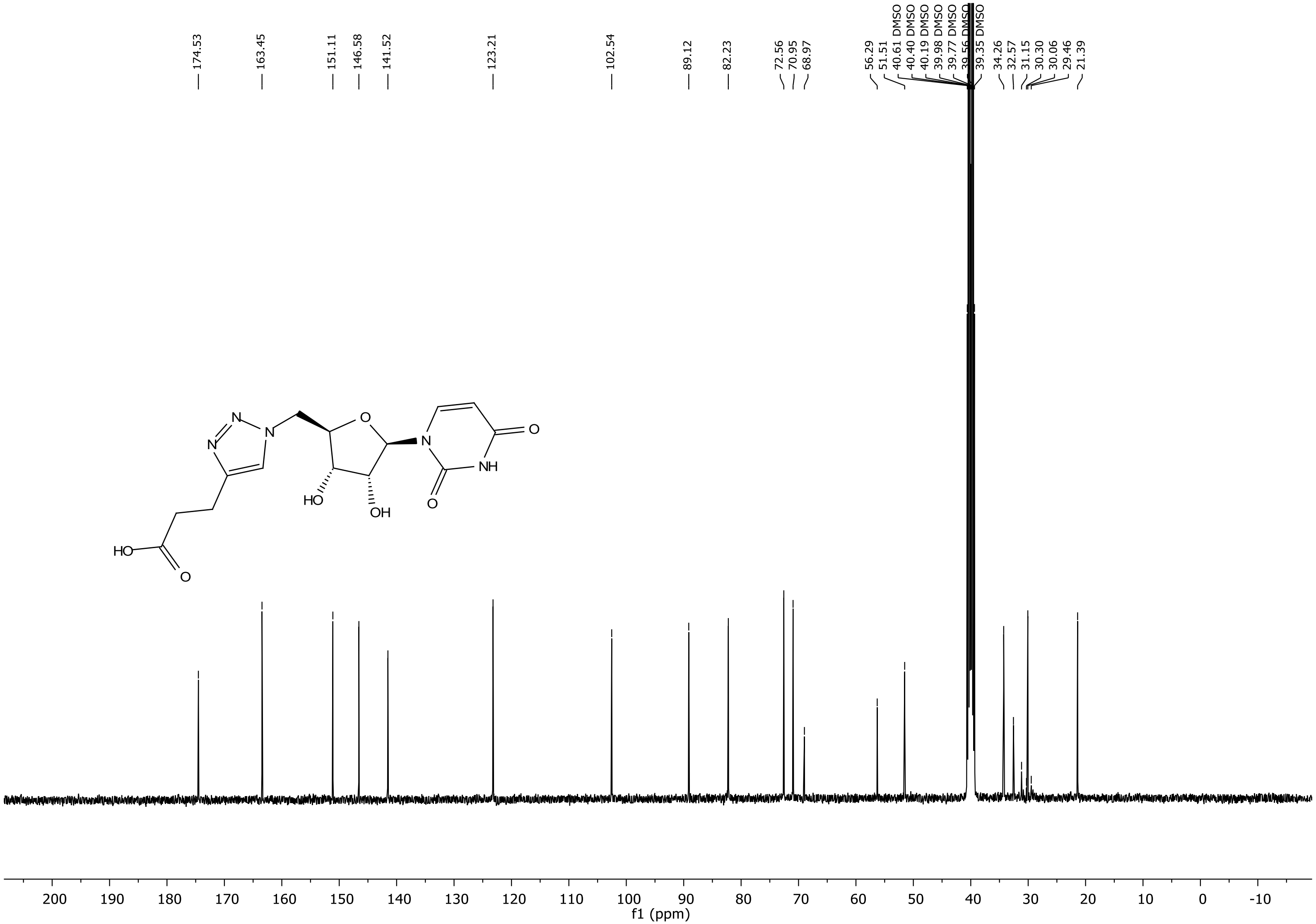

^{1}H-^{13}C HMBC NMR (DMSO-d6) of compound **3a**

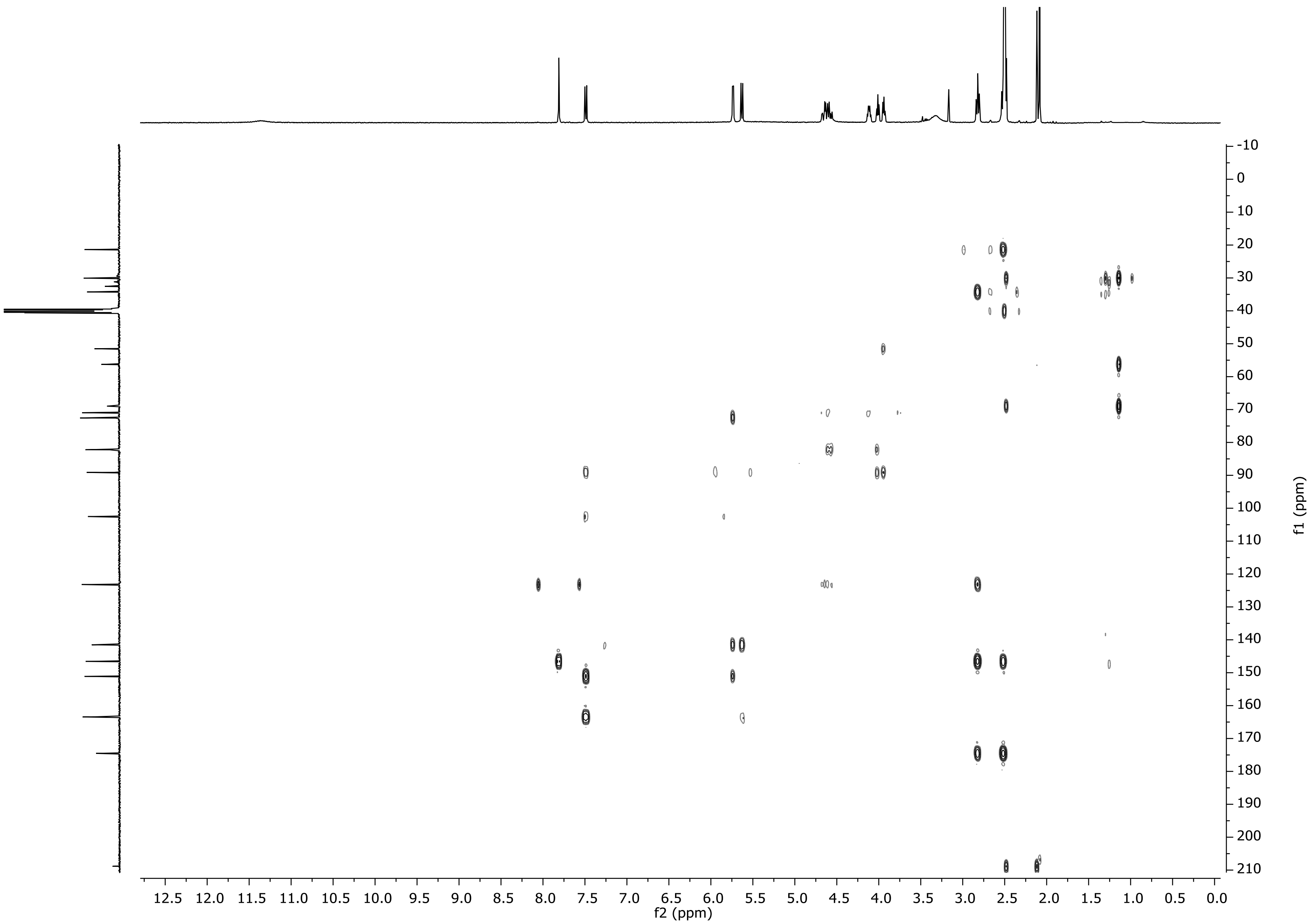

^{1}H NMR (DMSO-d6) of compound **3b**

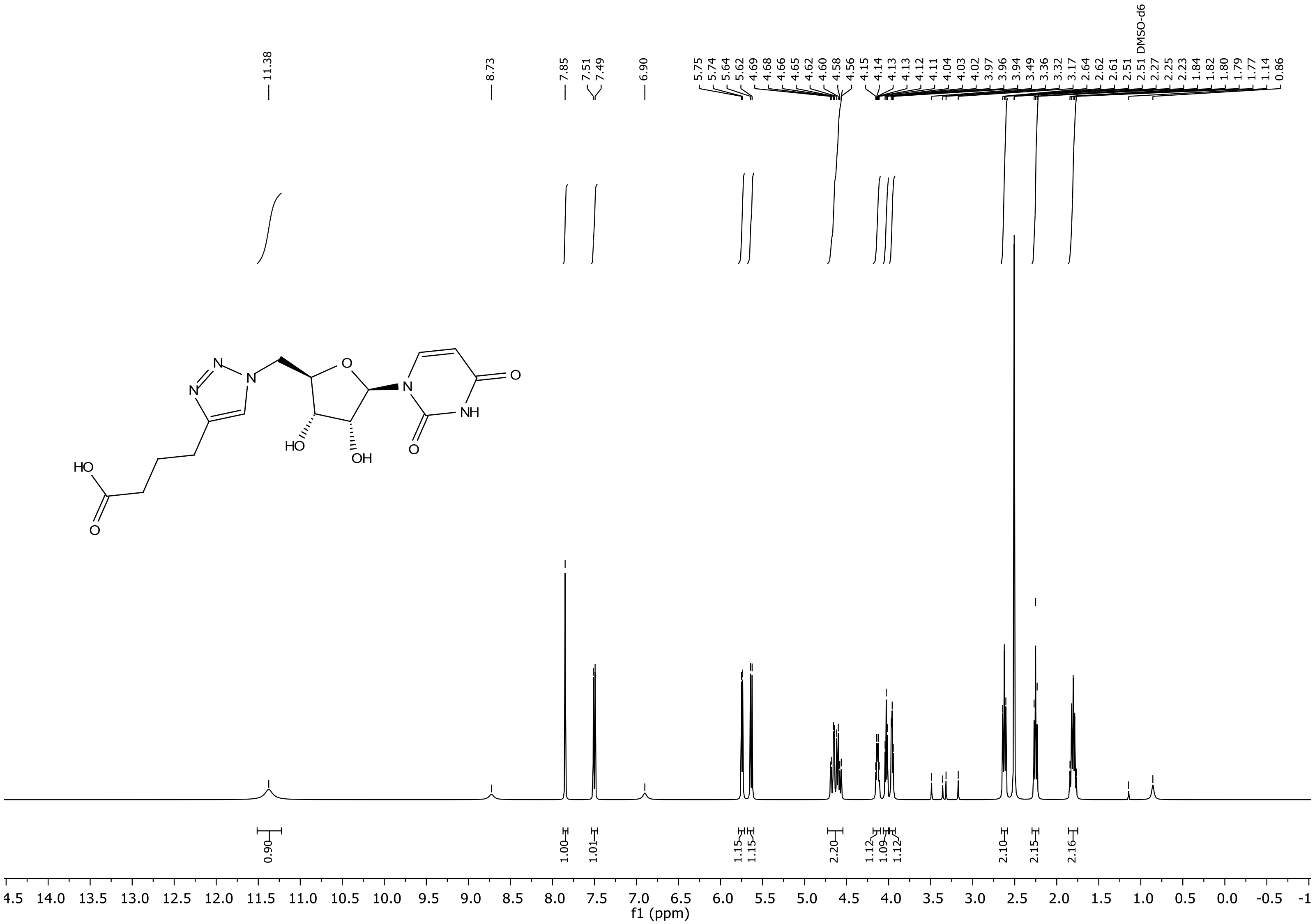

^{13}C NMR (DMSO-d6) of compound **3b**

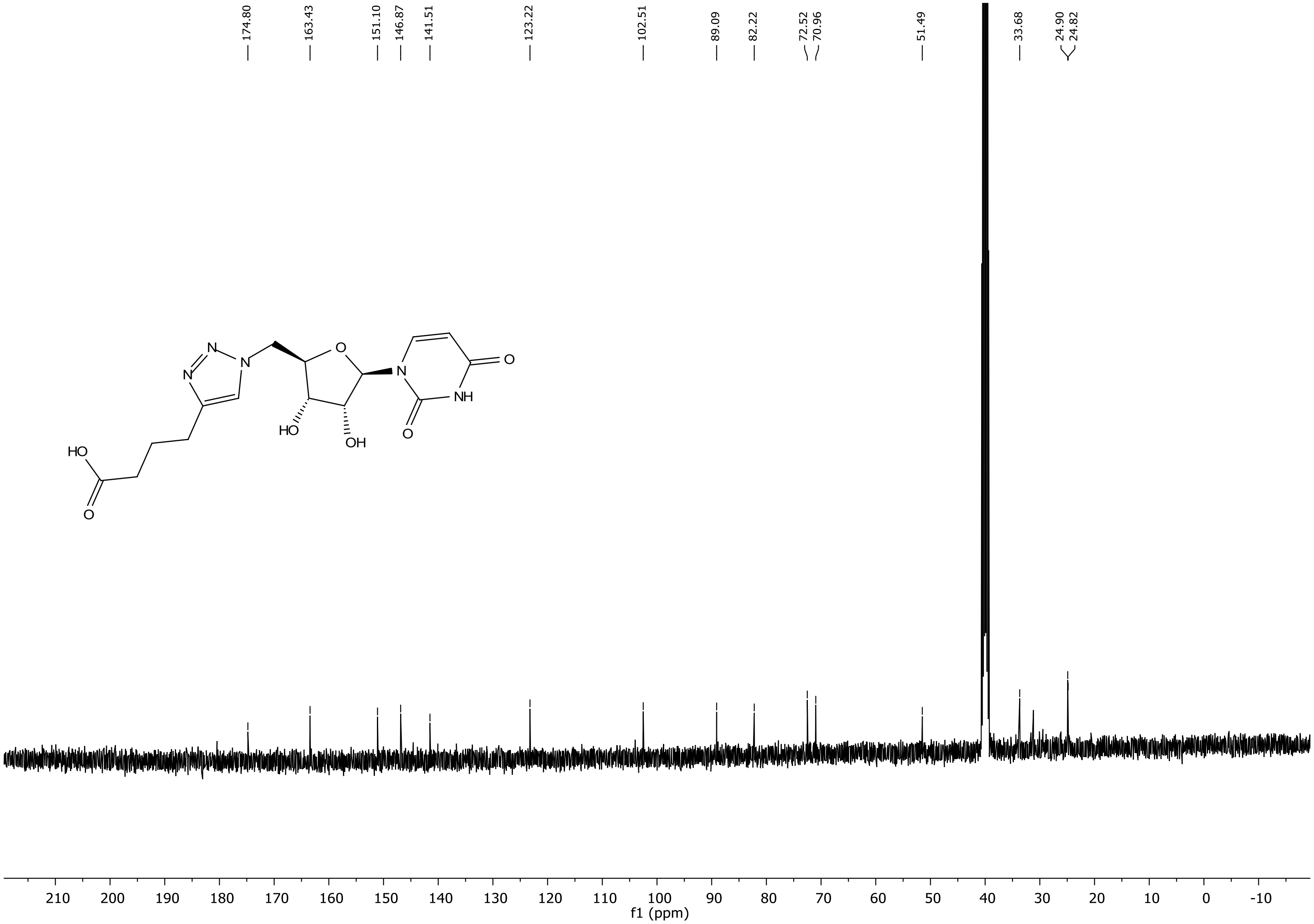
174.80
163.43
151.10
146.87
141.51
123.22
102.51
89.09
82.22
72.52
70.96
51.49
33.68
24.90
24.82
HO
O
N
N
N
O
N
NH
O
O
HO
OH
210 200 190 180 170 160 150 140 130 120 110 100 90 80 70 60 50 40 30 20 10 0 -10
f1 (ppm)

^{1}H NMR (DMSO-d6) of compound **3c**

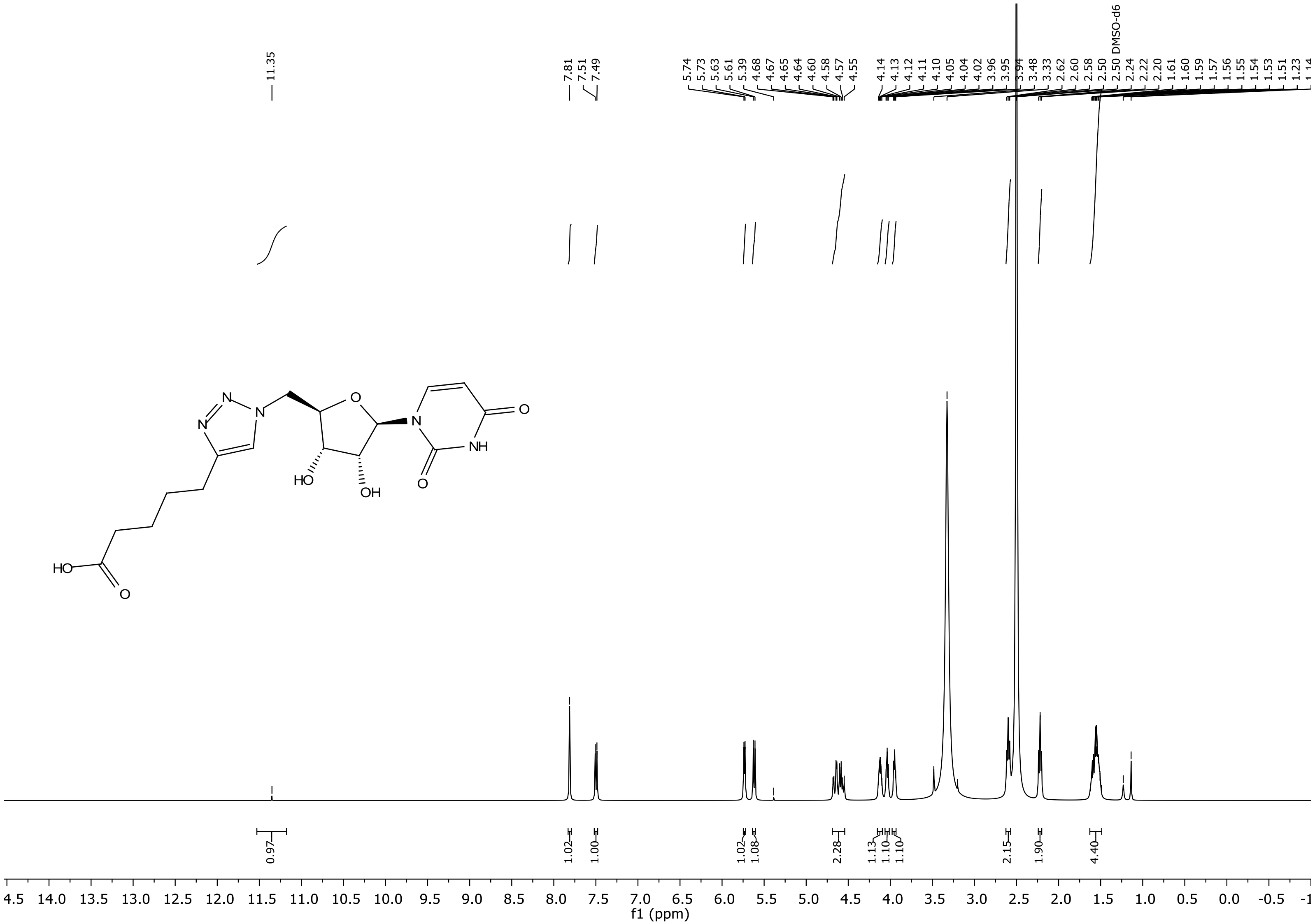

^{13}C NMR (DMSO-d6) of compound **3c**

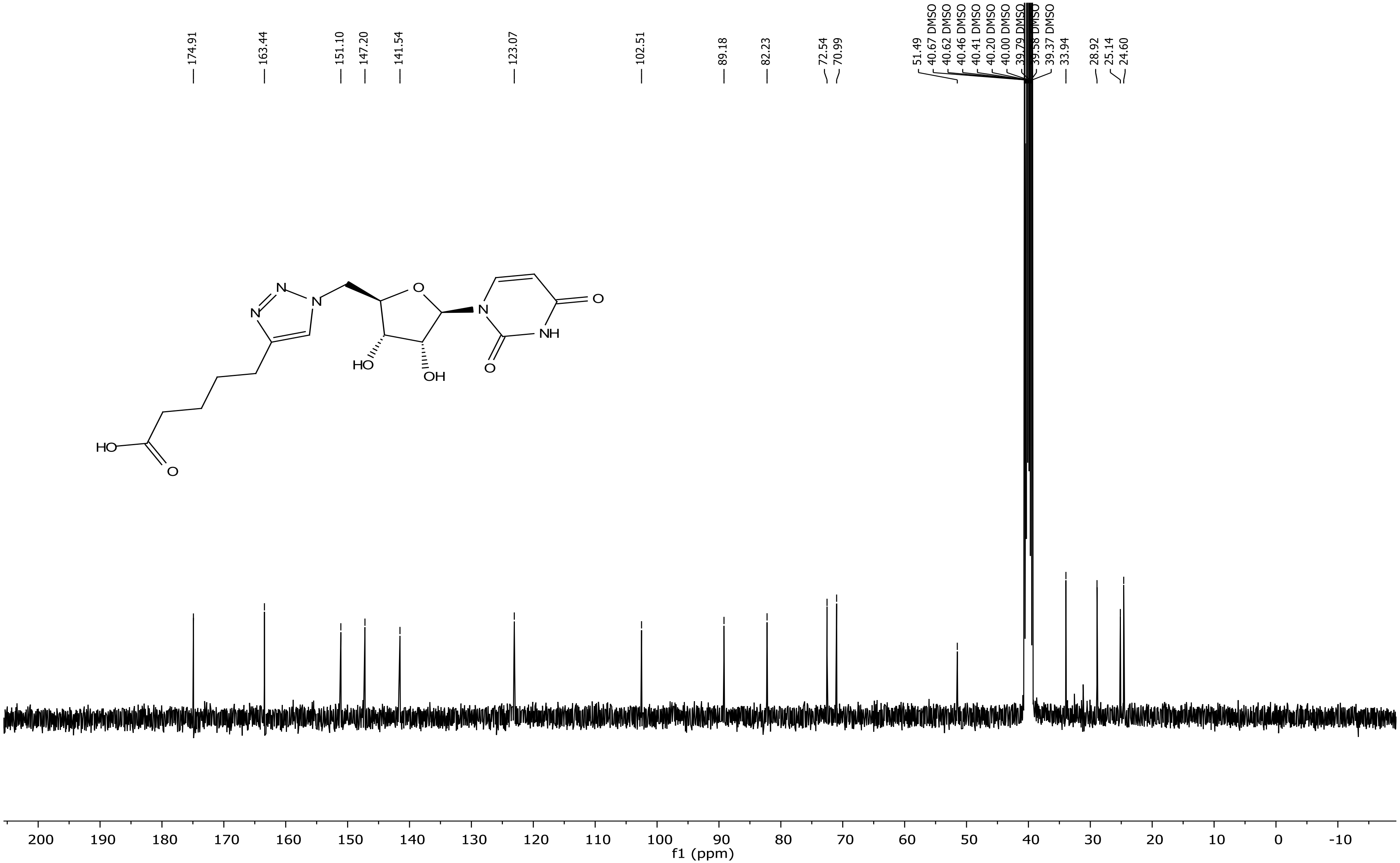
174.91
163.44
151.10
147.20
141.54
123.07
102.51
89.18
82.23
72.54
70.99
51.49
40.67 DMSO
40.62 DMSO
40.46 DMSO
40.41 DMSO
40.20 DMSO
40.00 DMSO
39.79 DMSO
39.58 DMSO
39.37 DMSO
33.94
28.92
25.14
24.60
200 190 180 170 160 150 140 130 120 110 100 90 80 70 60 50 40 30 20 10 0 -10
f1 (ppm)
HO
O
N
N
N
O
HO
OH
N
NH
O
O

^{1}H-^{13}C HMBC NMR (DMSO-d6) of compound **3c**

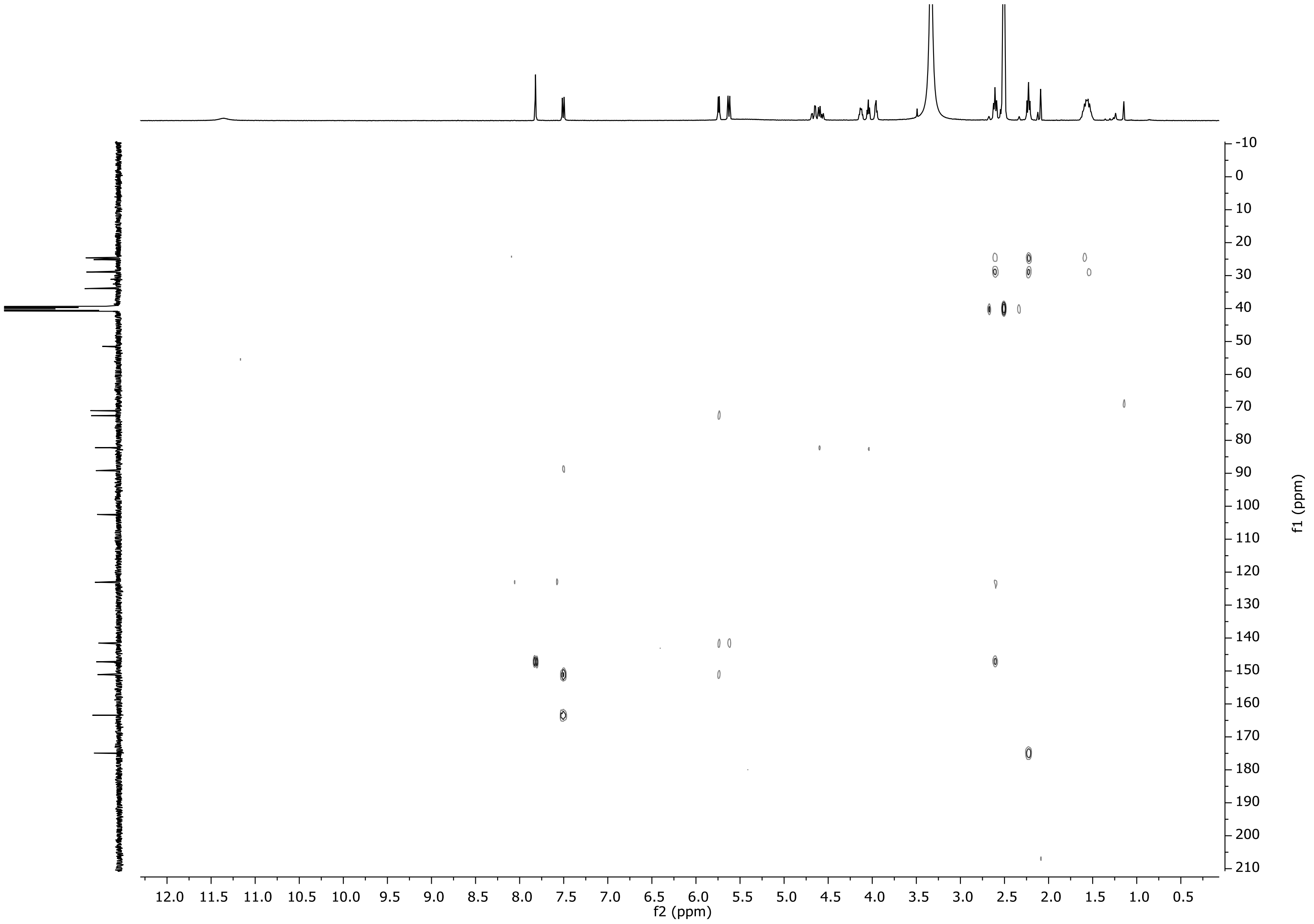

^{1}H NMR (DMSO-d6) of compound **3d**

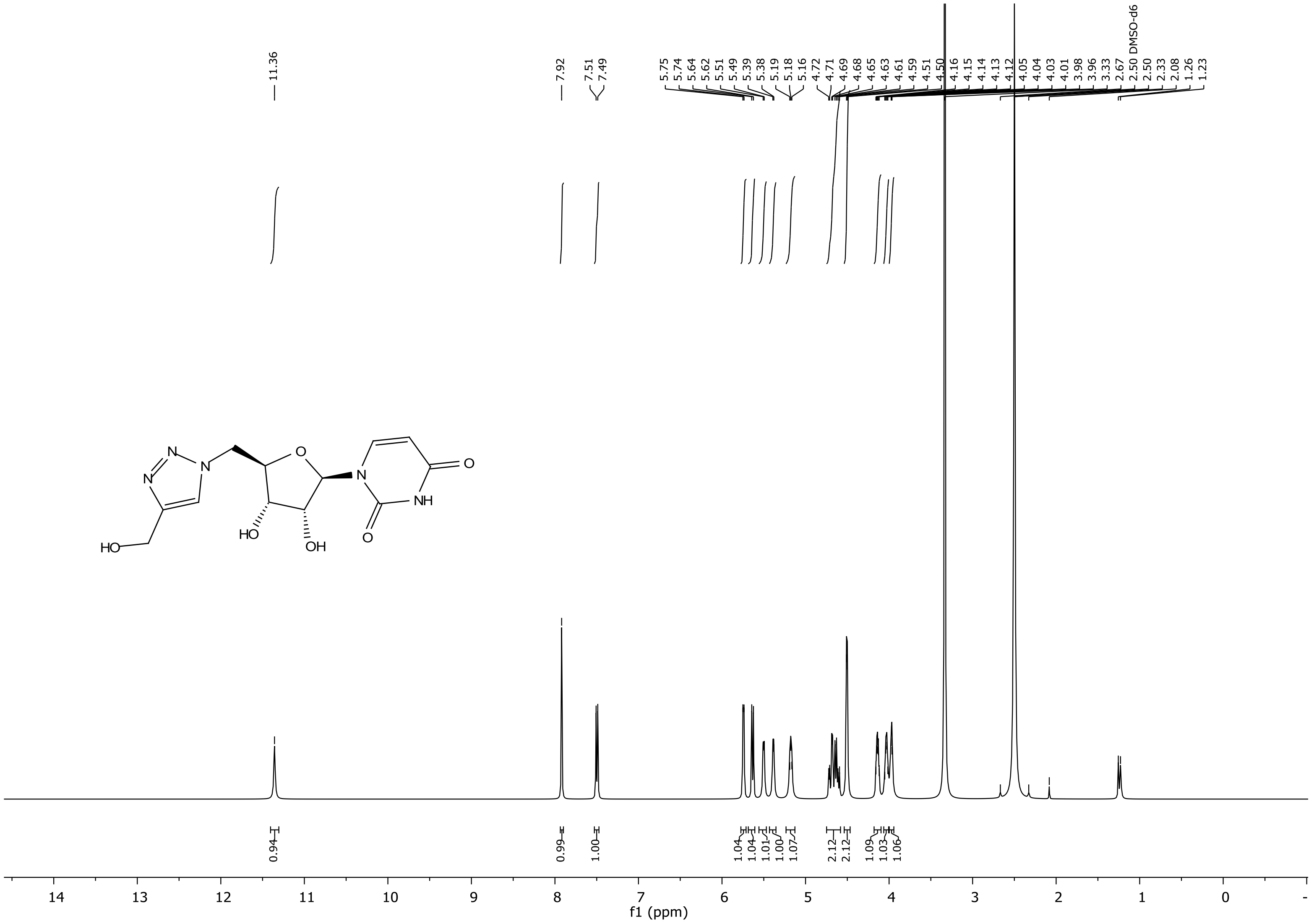

^{13}C NMR (DMSO-d6) of compound **3d**

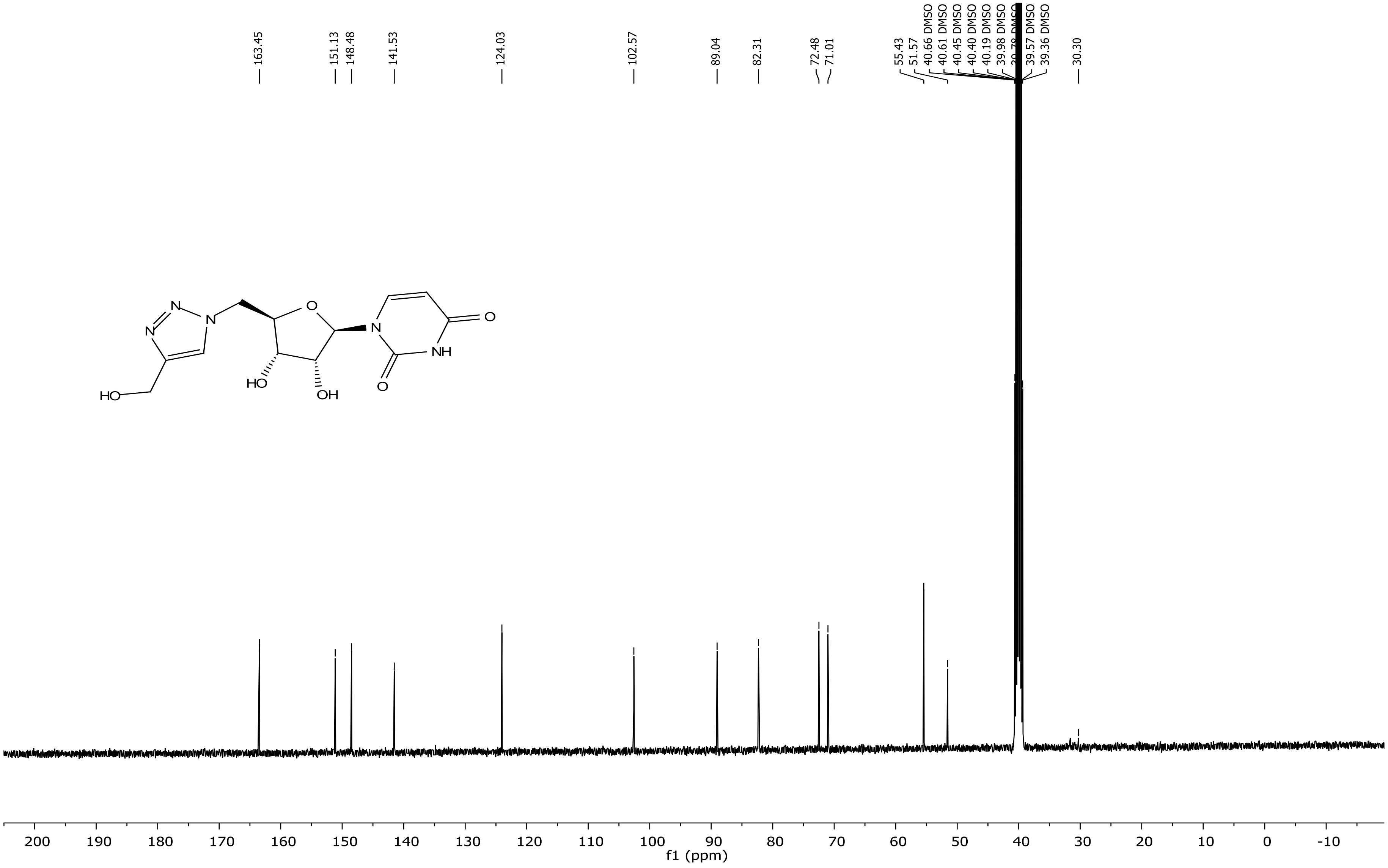

^{1}H-^{13}C HMBC NMR (DMSO-d6) of compound **3d**

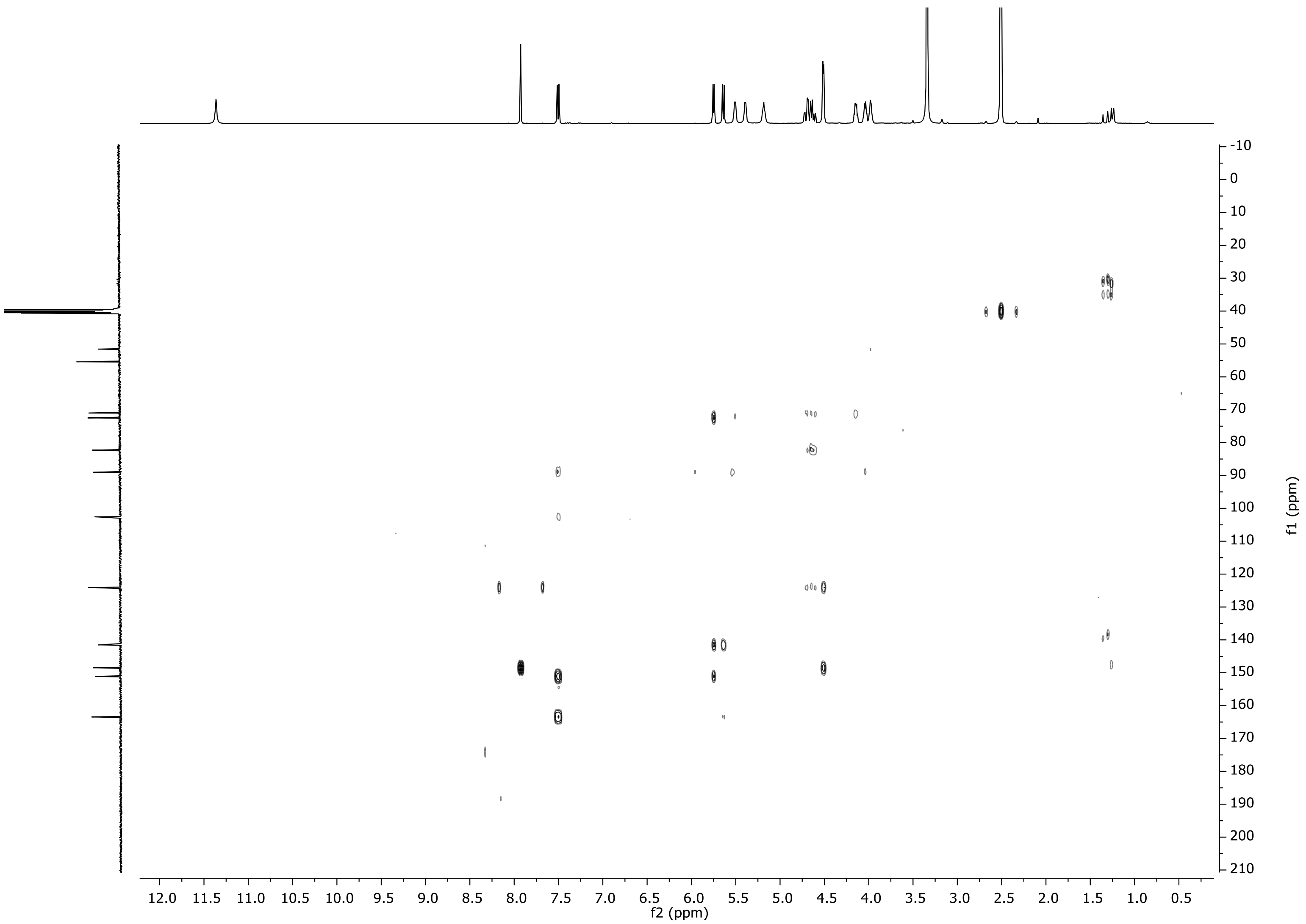

^{1}H NMR (DMSO-d6) of compound **3e**

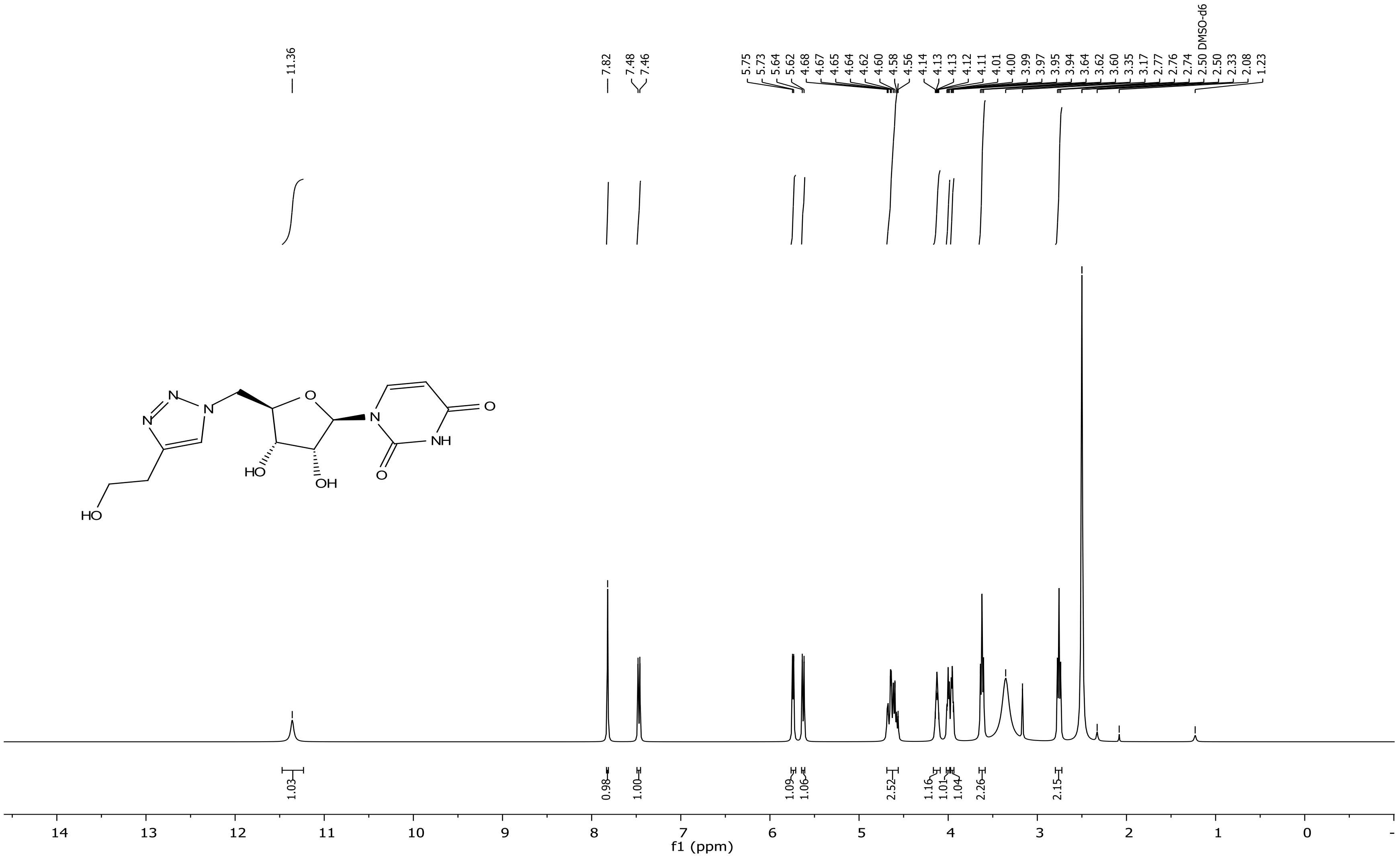

^{13}C NMR (DMSO-d6) of compound 3**e**

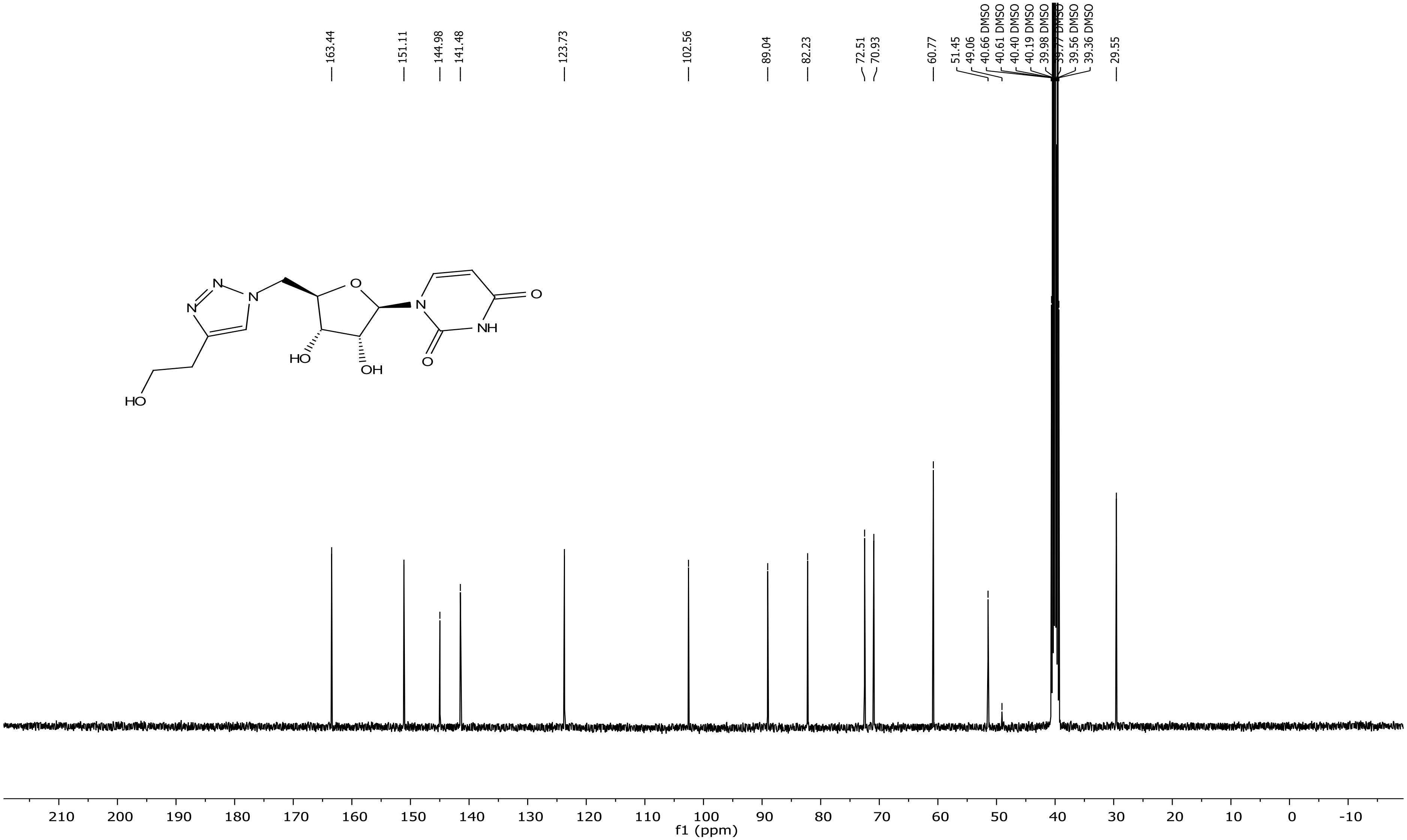

^{1}H-^{13}C HMBC NMR (DMSO-d6) of compound **3e**

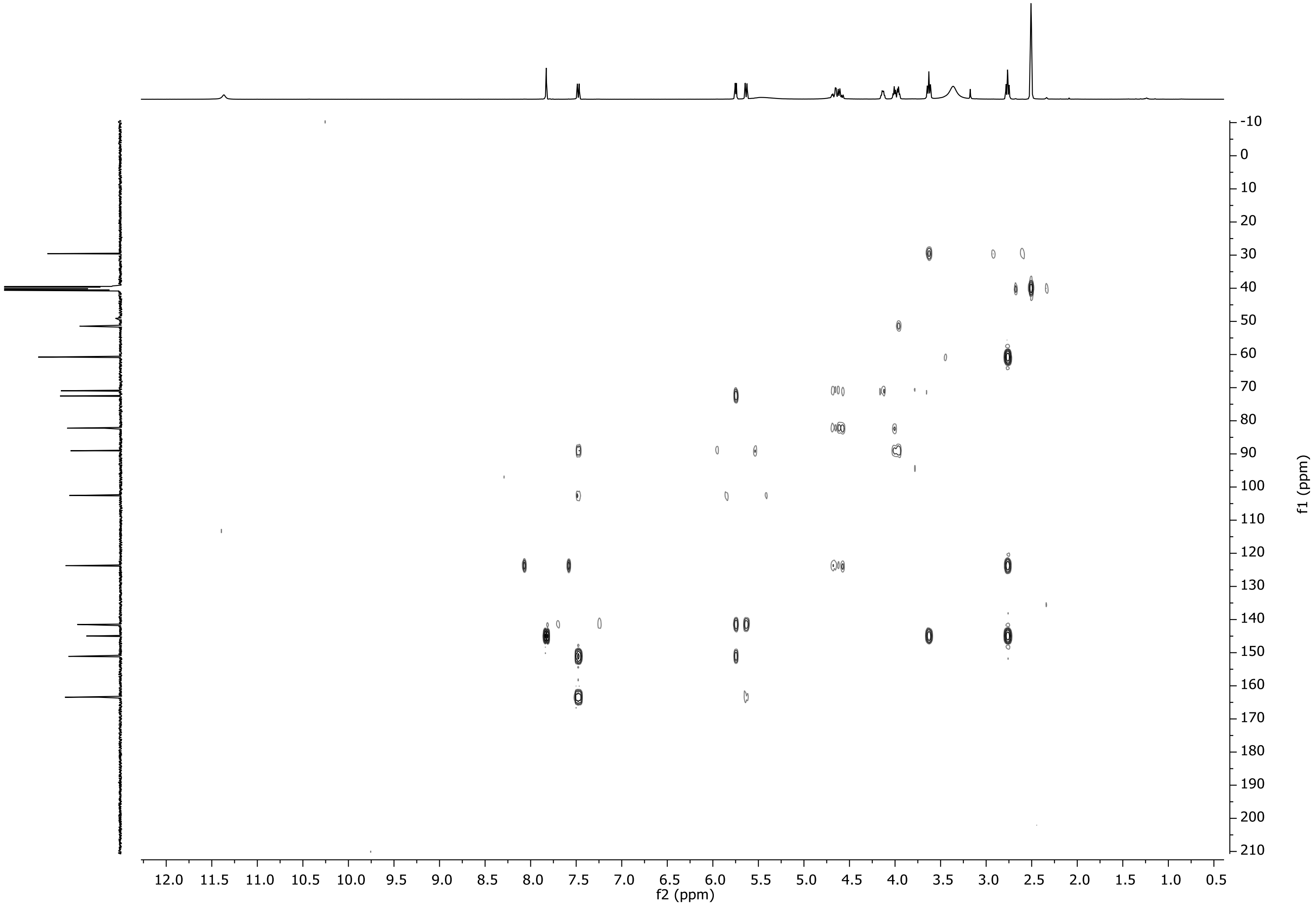

^{1}H NMR (DMSO-d6) of compound **3f**

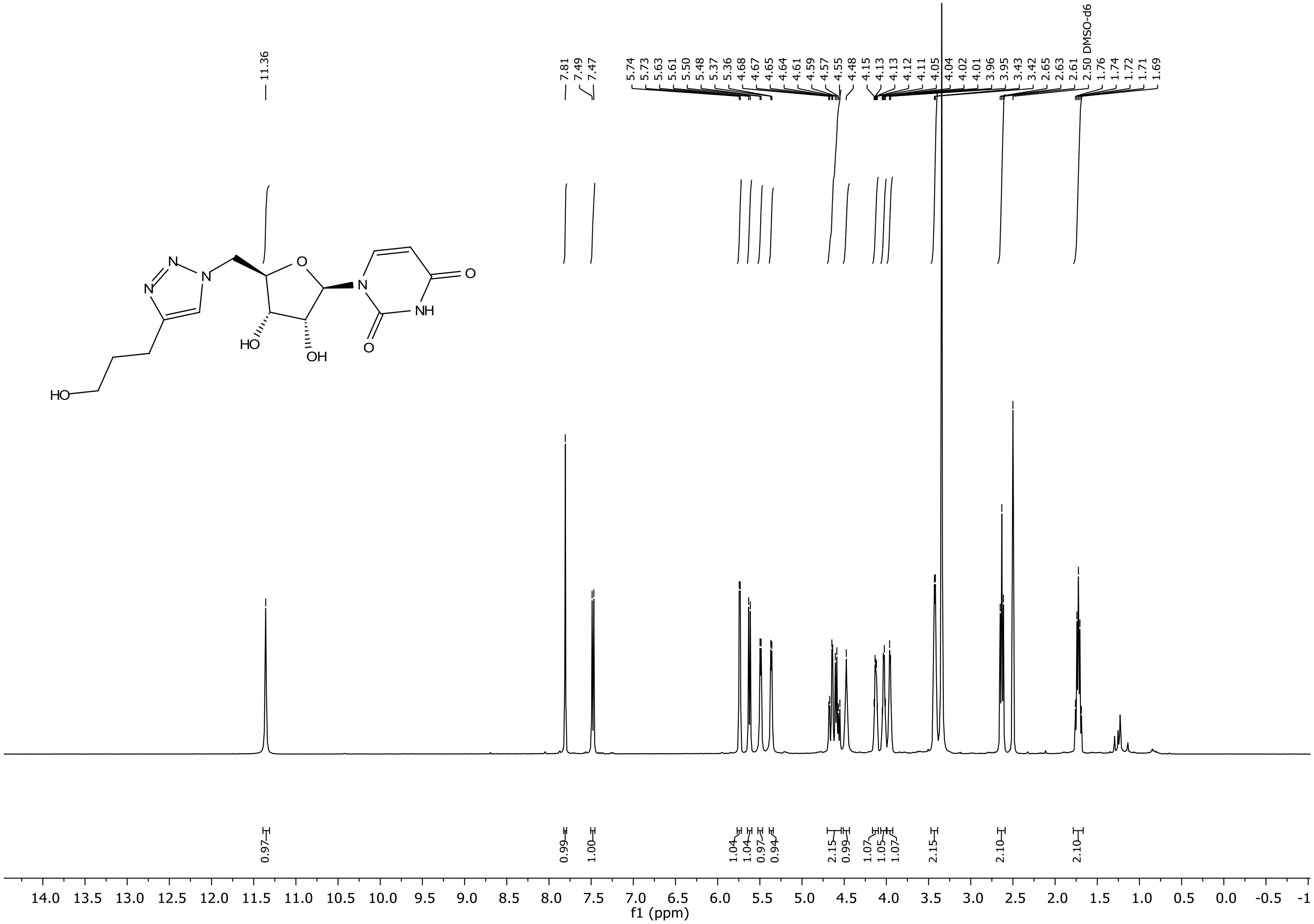

^{13}C NMR (DMSO-d6) of compound **3f**

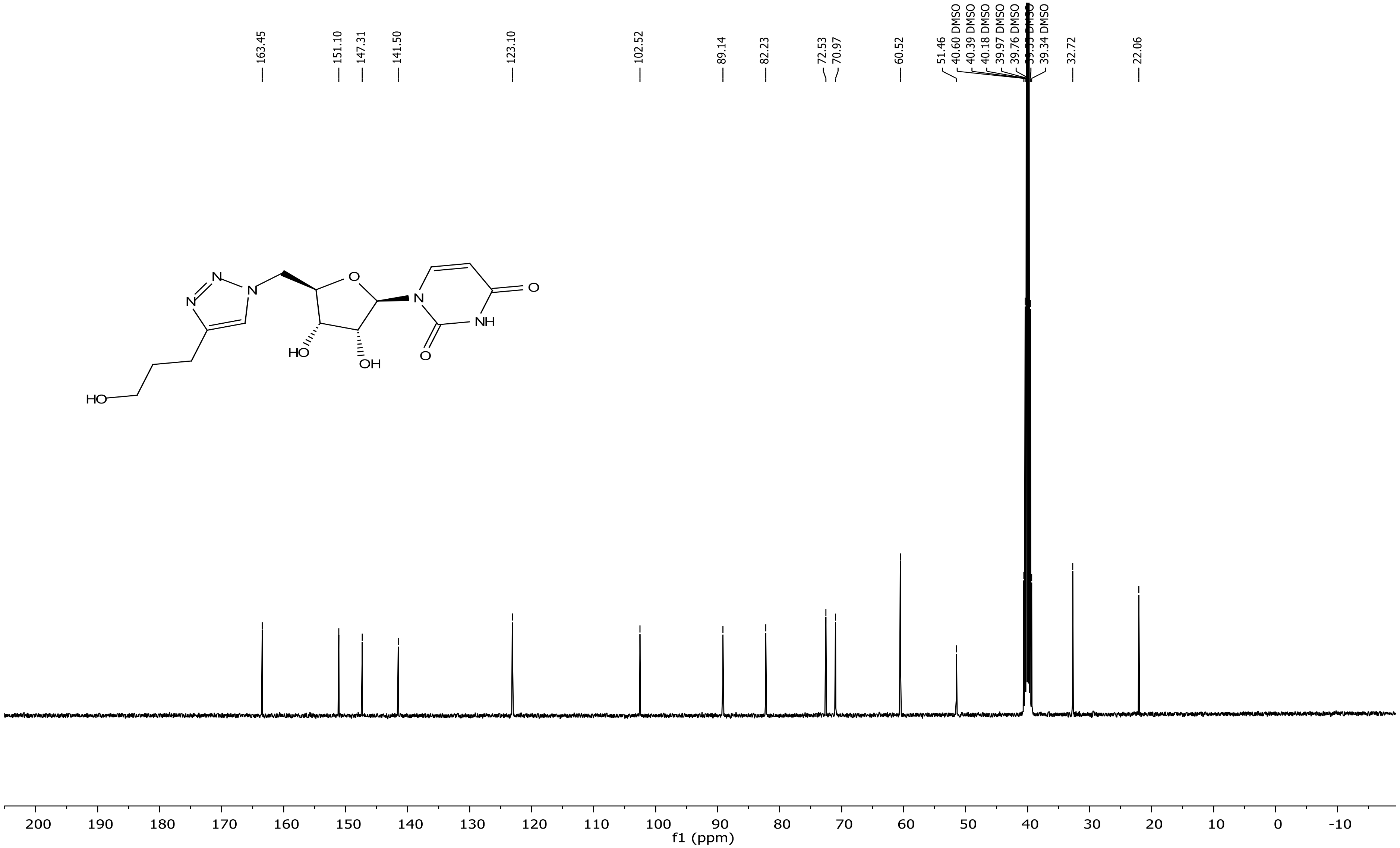

^{1}H-^{13}C HMBC NMR (DMSO-d6) of compound **3f**

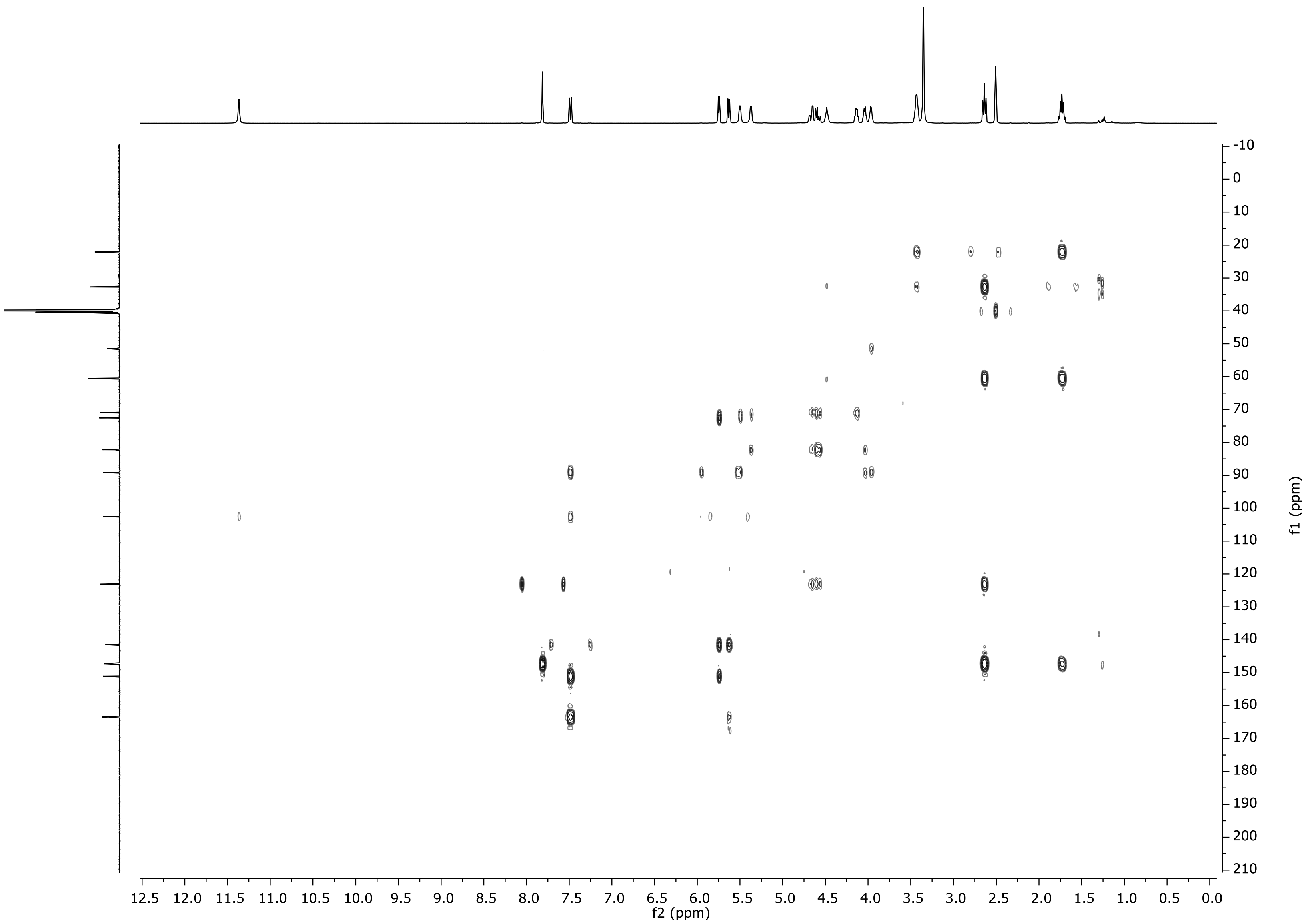

f2 (ppm)
f1 (ppm)

^{1}H NMR (DMSO-d6) of compound **3g**

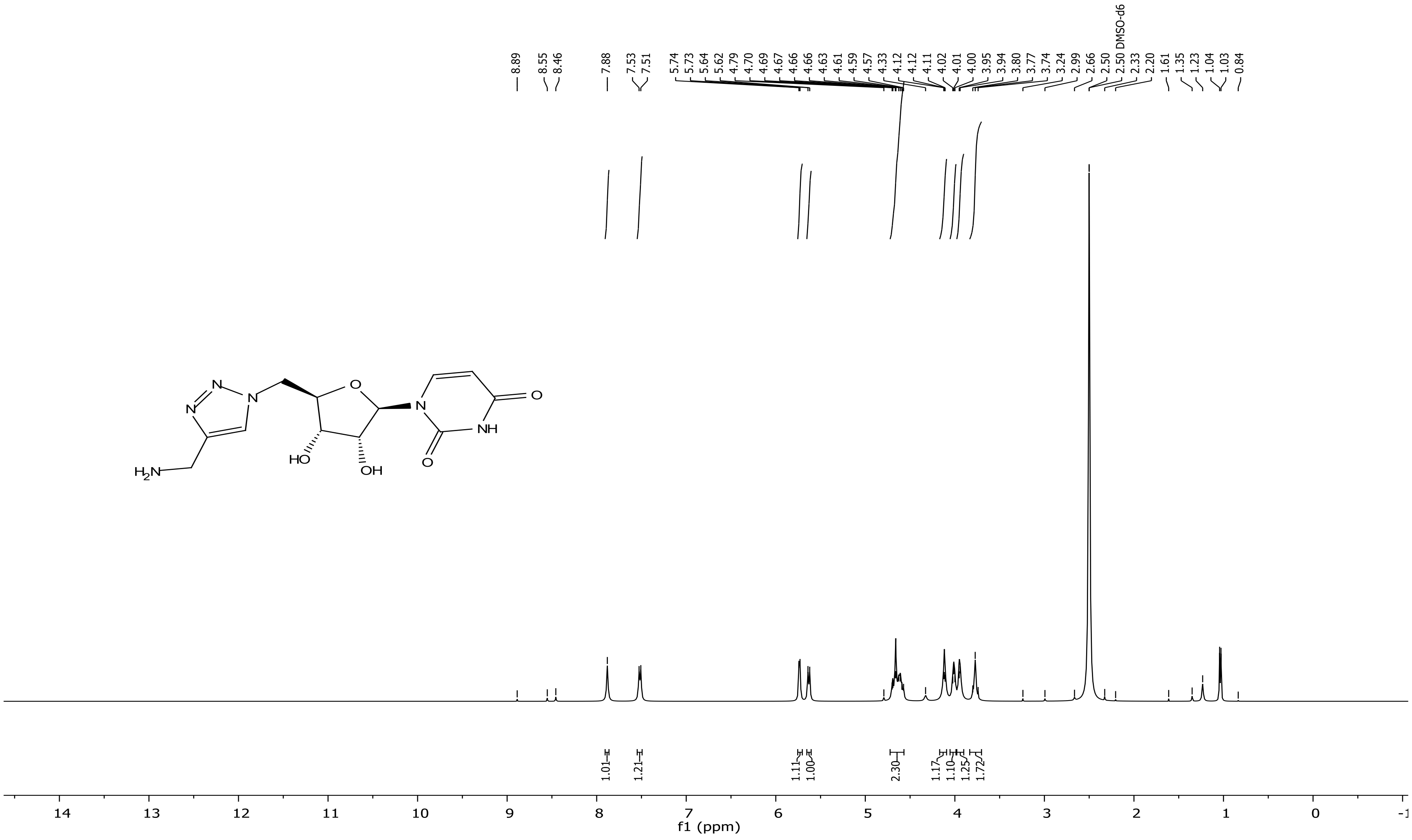

^{13}C NMR (DMSO-d6) of compound **3g**

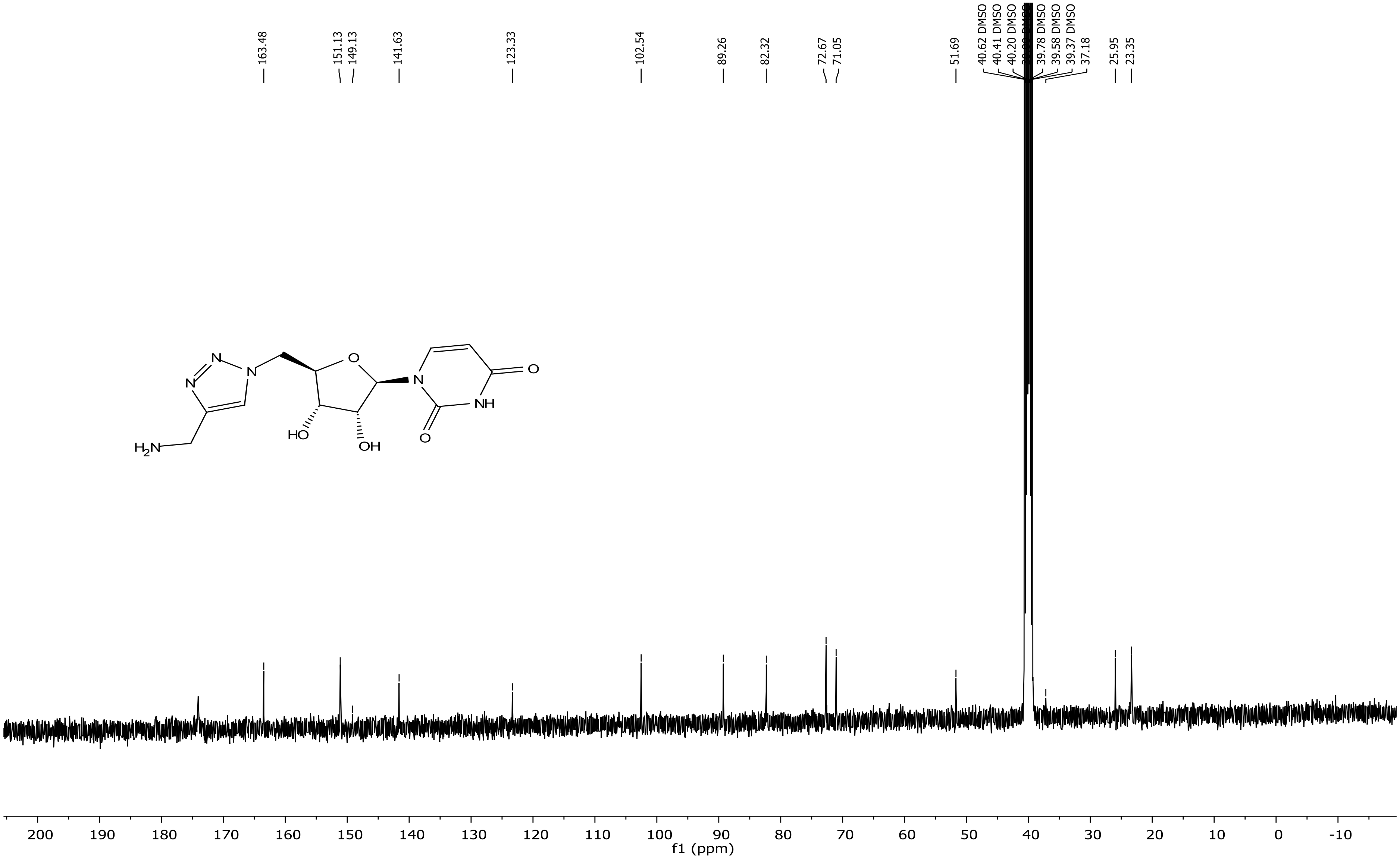

163.48
151.13
149.13
141.63
123.33
102.54
89.26
82.32
72.67
71.05
51.69
40.62 DMSO
40.41 DMSO
40.20 DMSO
39.78 DMSO
39.58 DMSO
39.37 DMSO
37.18
25.95
23.35
200 190 180 170 160 150 140 130 120 110 100 90 80 70 60 50 40 30 20 10 0 -10
f1 (ppm)
N
N
N
O
N
NH
O
O
HO
OH
H2N

^{1}H-^{13}C HMBC NMR (DMSO-d6) of compound **3g**

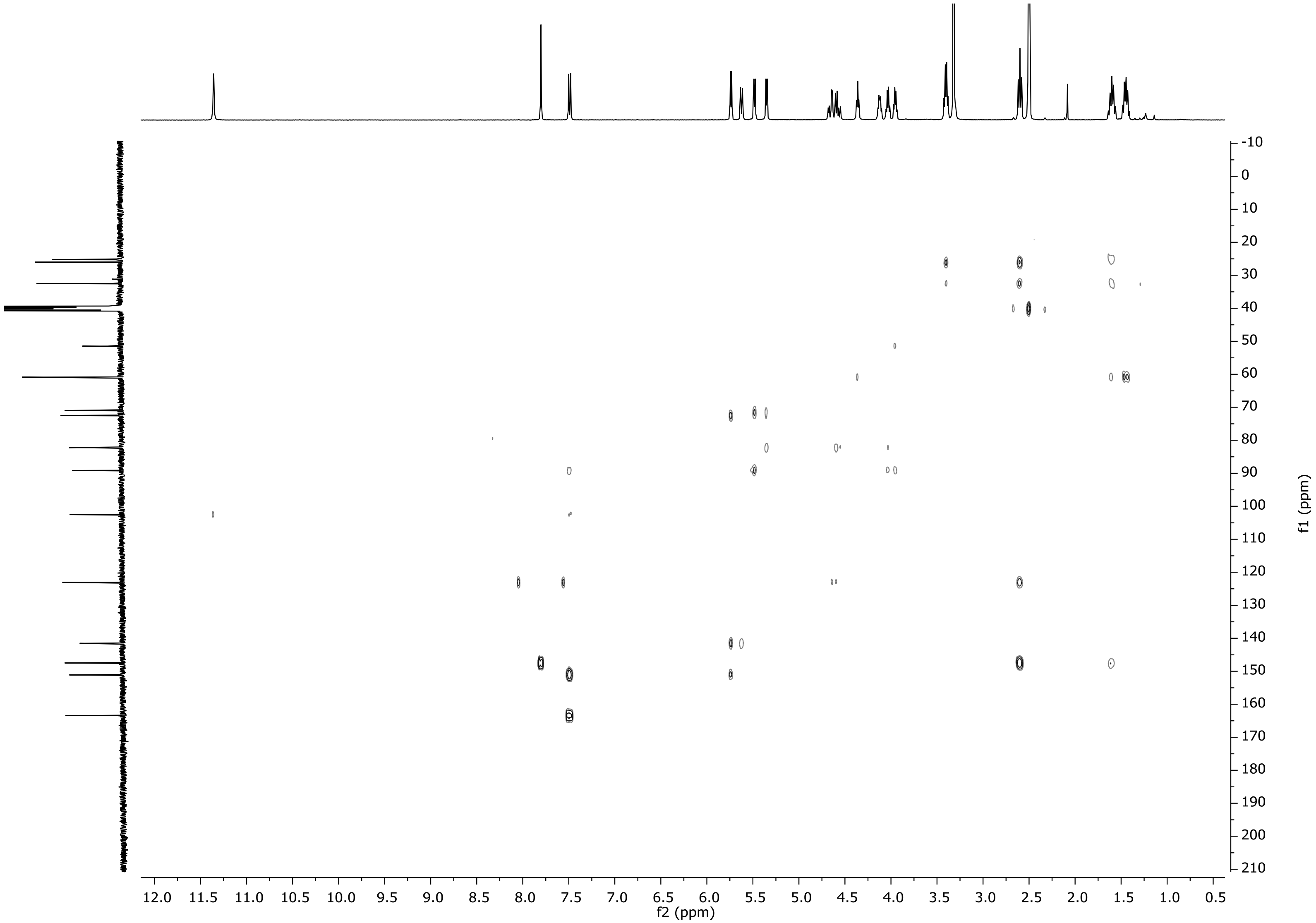

^{1}H NMR (DMSO-d6) of compound **3h**

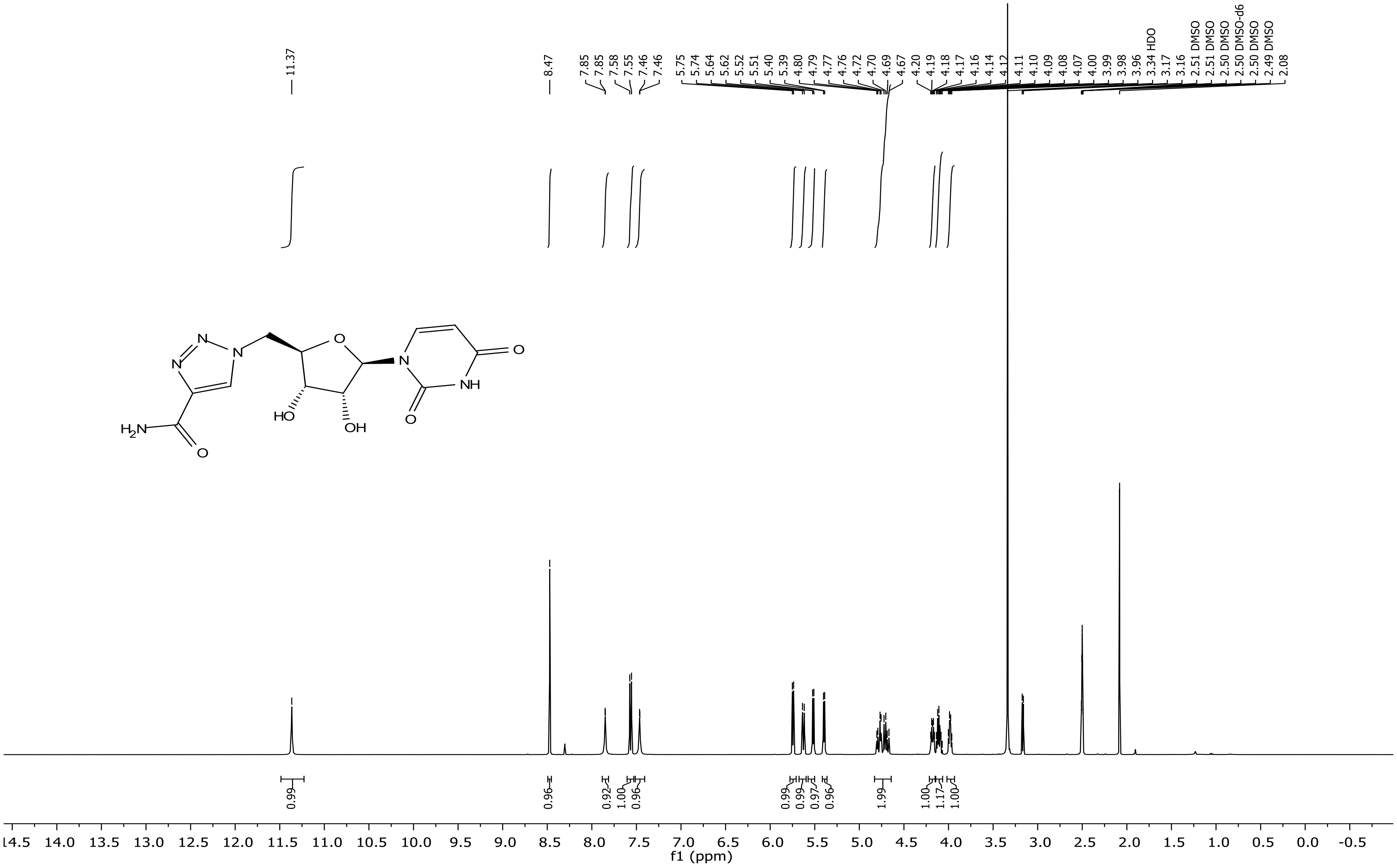

^{13}C NMR (DMSO-d6) of compound **3h**

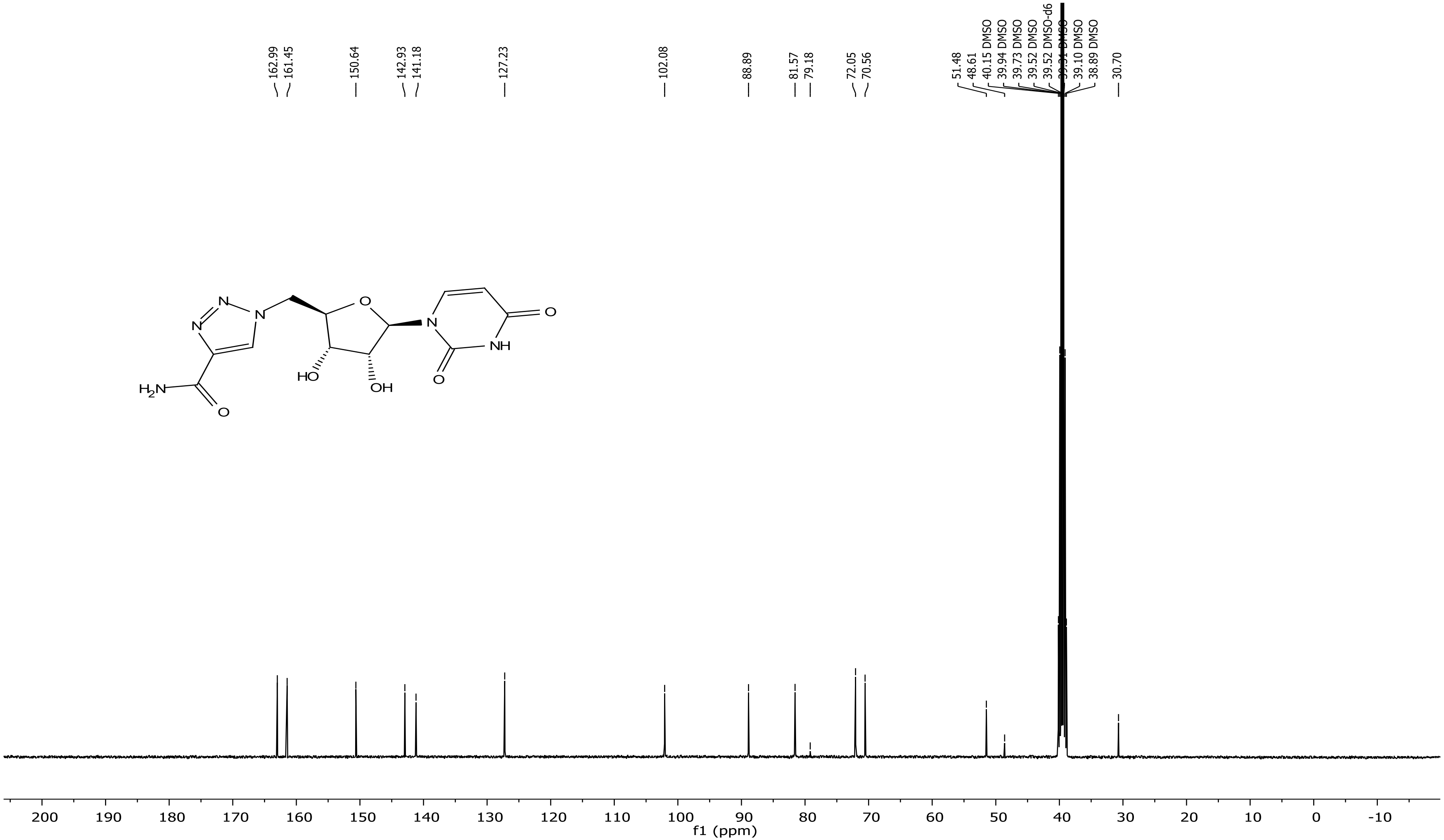

^{1}H-^{13}C HMBC NMR (DMSO-d6) of compound **3h**

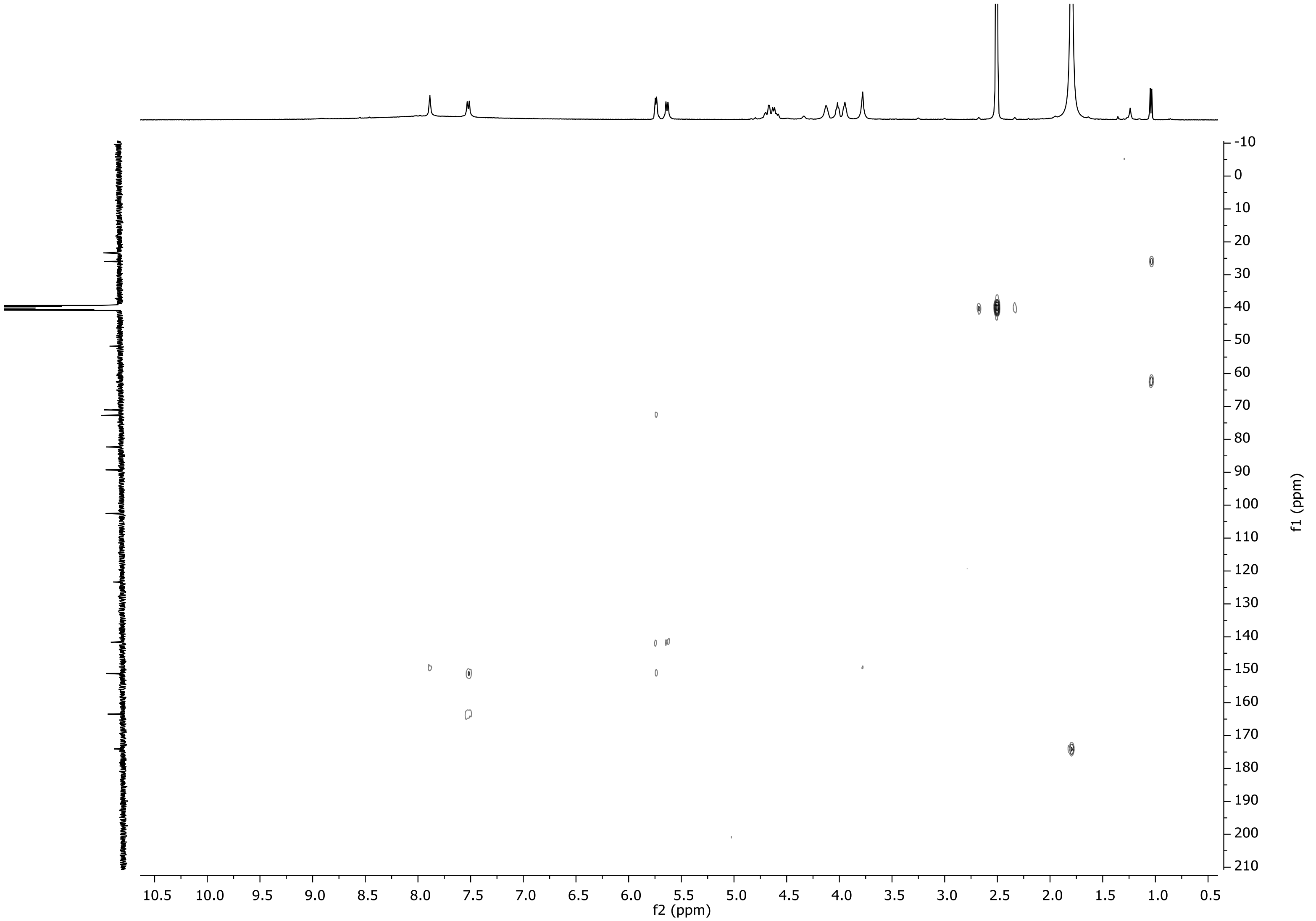

f2 (ppm)
f1 (ppm)
10.5 10.0 9.5 9.0 8.5 8.0 7.5 7.0 6.5 6.0 5.5 5.0 4.5 4.0 3.5 3.0 2.5 2.0 1.5 1.0 0.5
-10 0 10 20 30 40 50 60 70 80 90 100 110 120 130 140 150 160 170 180 190 200 210

^{1}H NMR (DMSO-d6) of compound **3i**

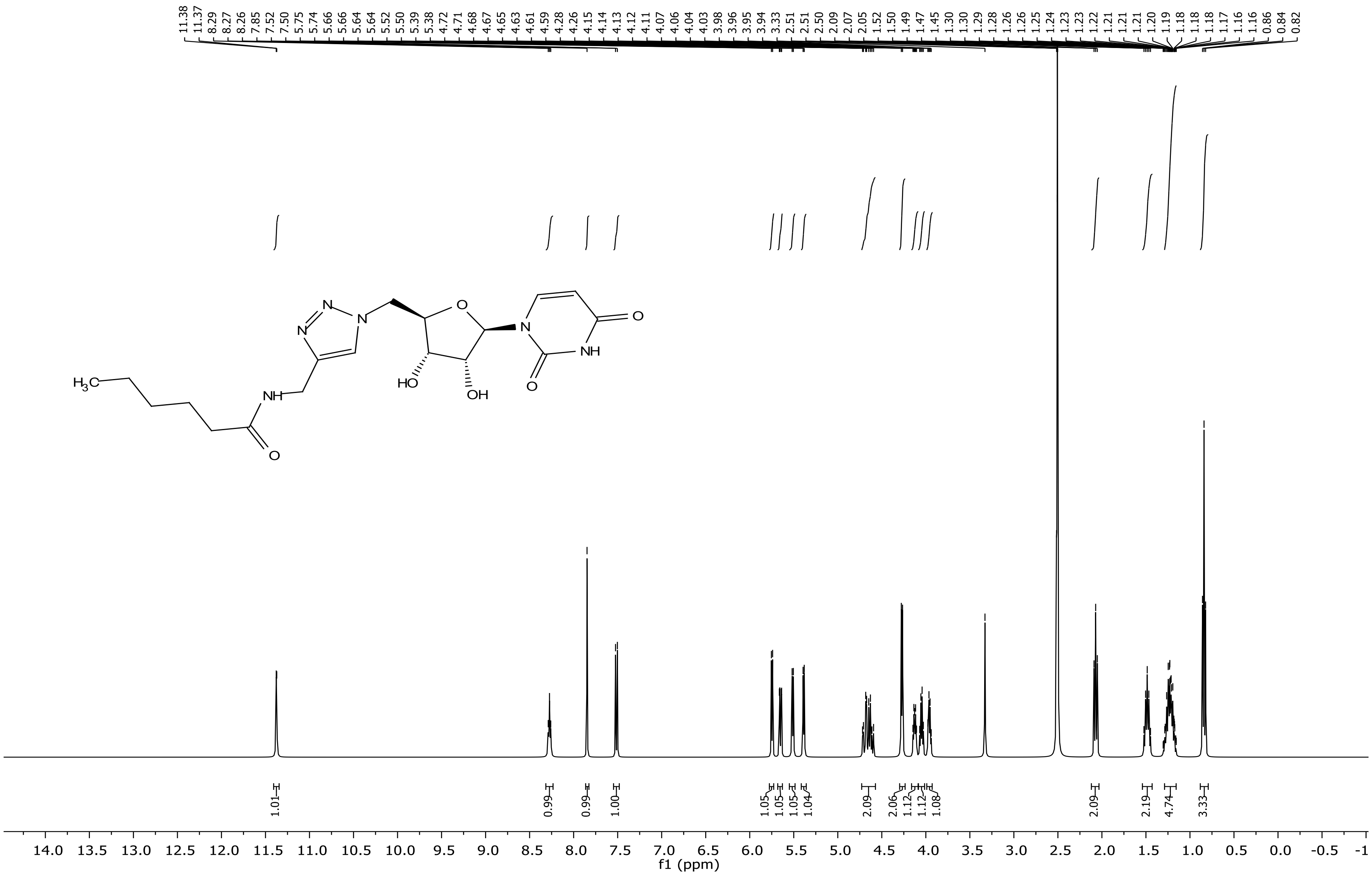

^{13}C NMR (DMSO-d6) of compound **3i**

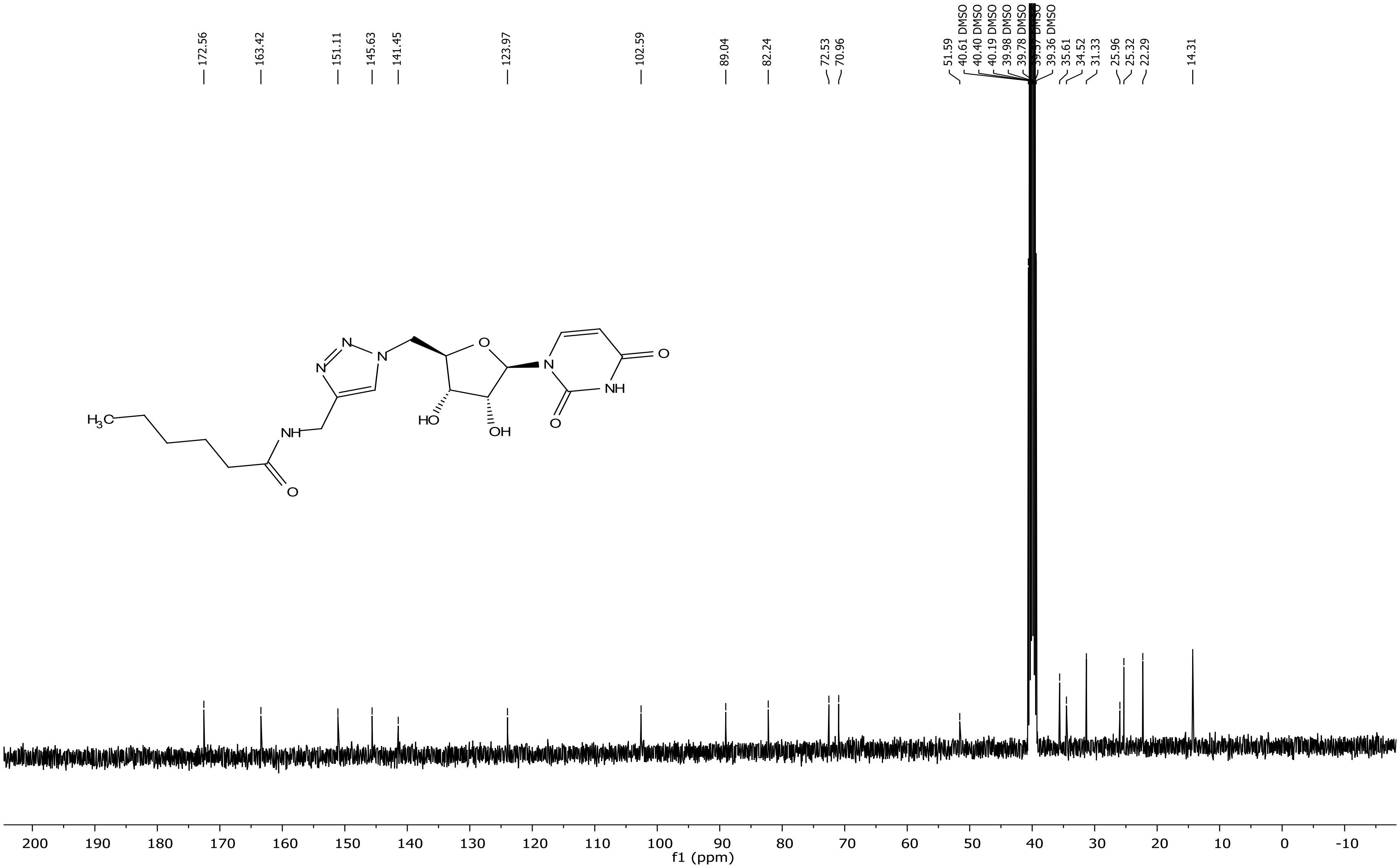

^{1}H-^{13}C HMBC NMR (DMSO-d6) of compound **3i**

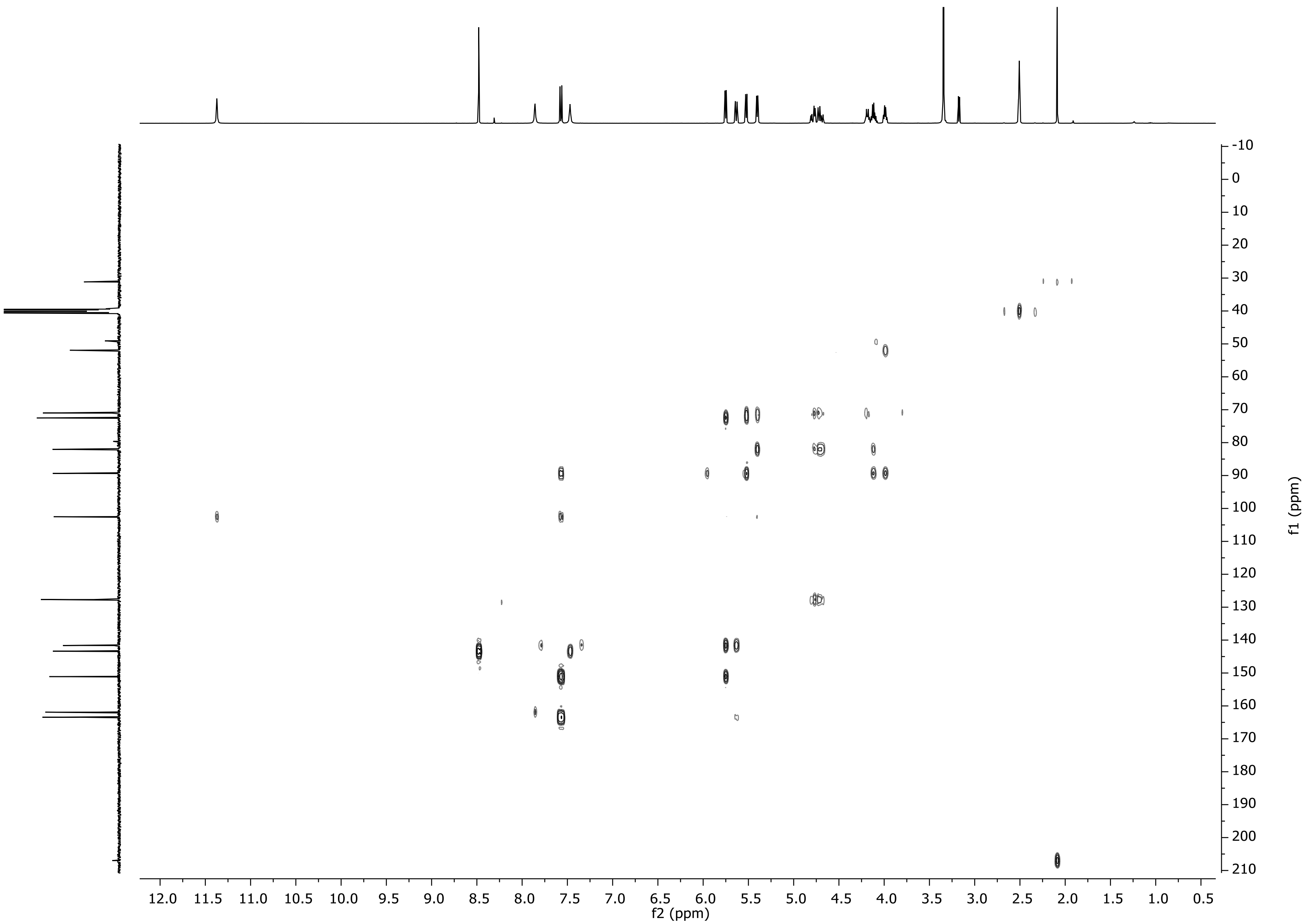

^{1}H NMR (DMSO-d6) of compound **3j**

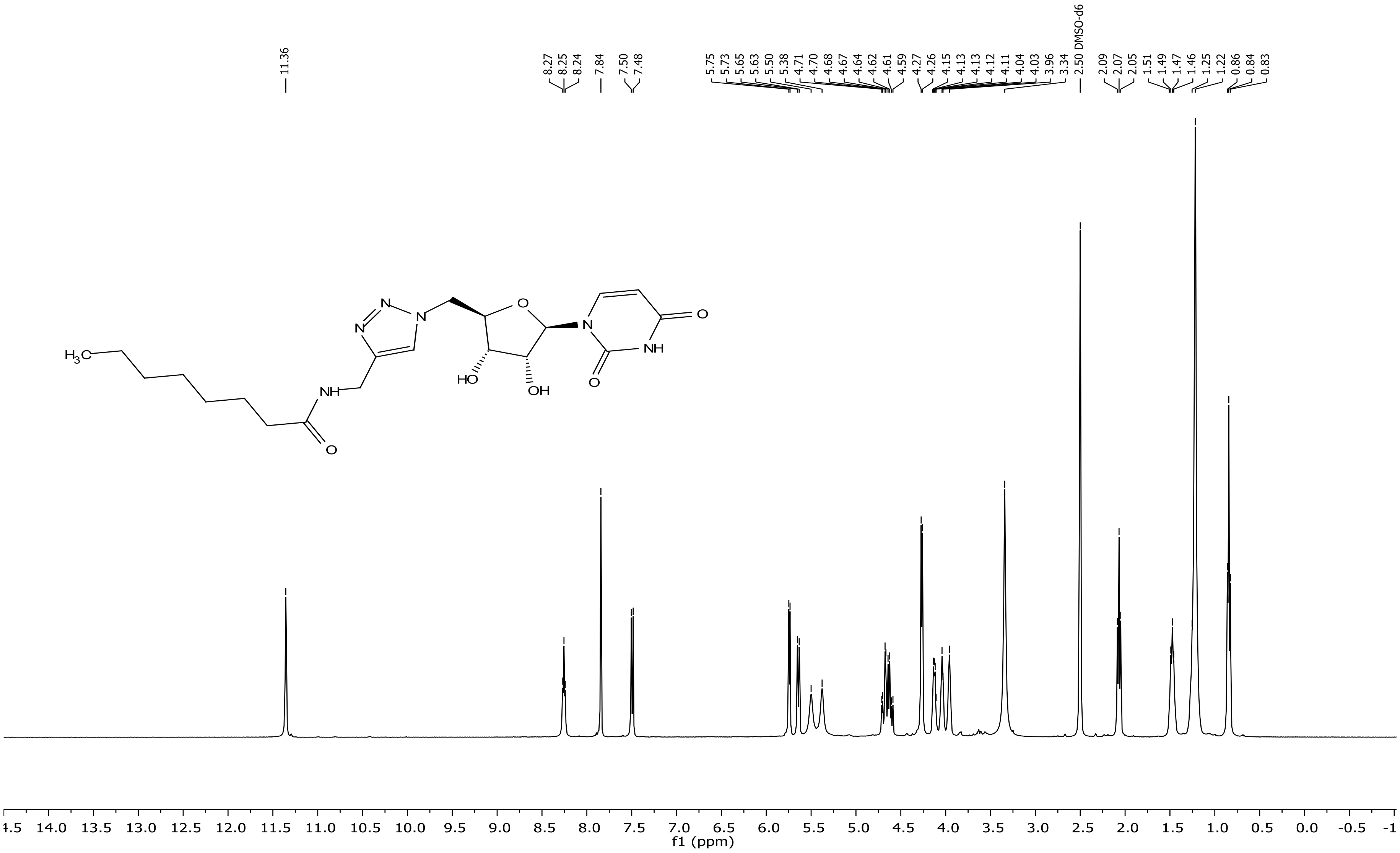

^{13}C NMR (DMSO-d6) of compound **3j**

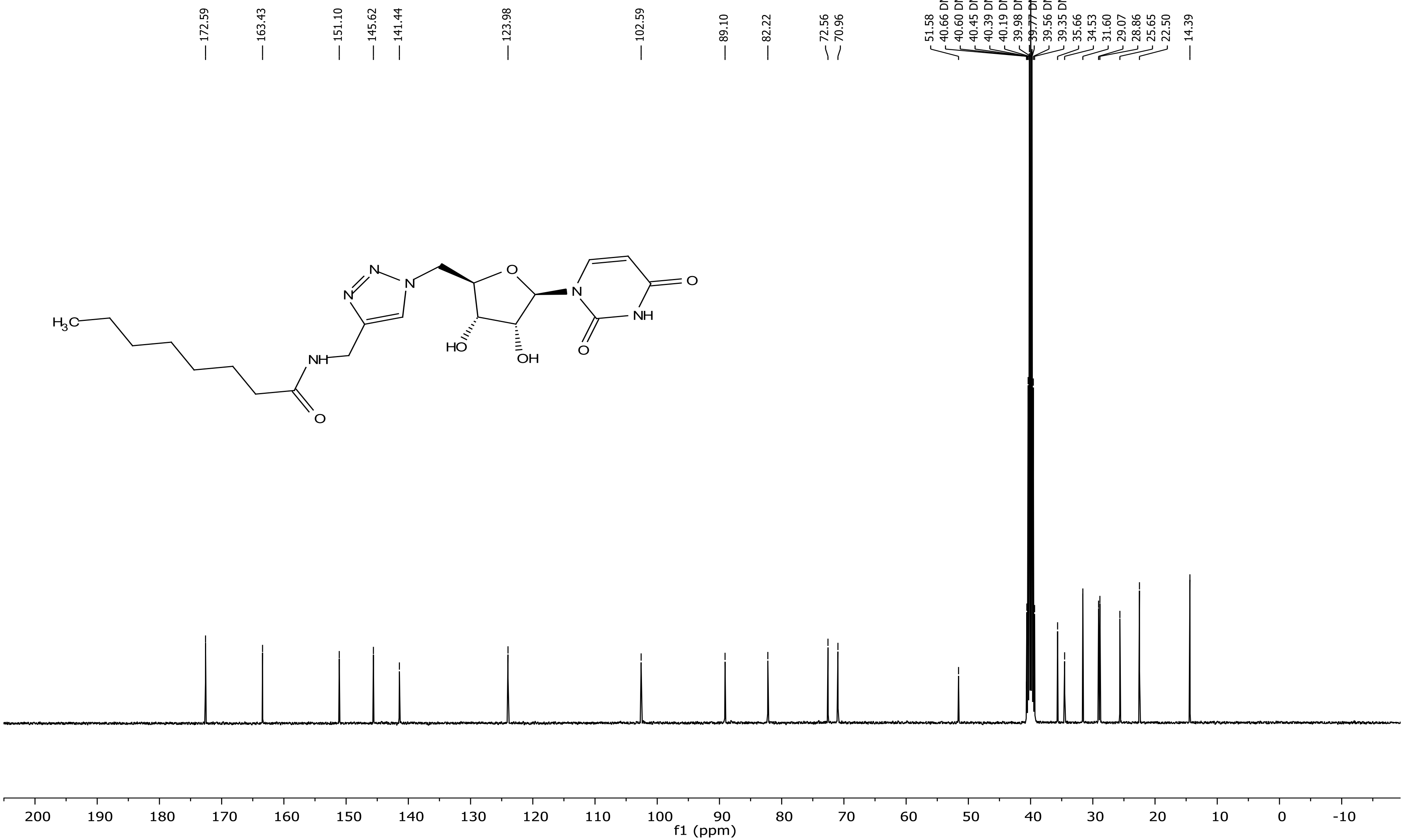

172.59
163.43
151.10
145.62
141.44
123.98
102.59
89.10
82.22
72.56
70.96
51.58
40.66 DMSO
40.60 DMSO
40.45 DMSO
40.39 DMSO
40.19 DMSO
39.98 DMSO
39.77 DMSO
39.56 DMSO
39.35 DMSO
35.66
34.53
31.60
29.07
28.86
25.65
22.50
14.39
H3C
NH
O
N
N
N
O
HO
OH
N
NH
O
O
200 190 180 170 160 150 140 130 120 110 100 90 80 70 60 50 40 30 20 10 0 -10
f1 (ppm)

^{1}H NMR (DMSO-d6) of compound **3k**

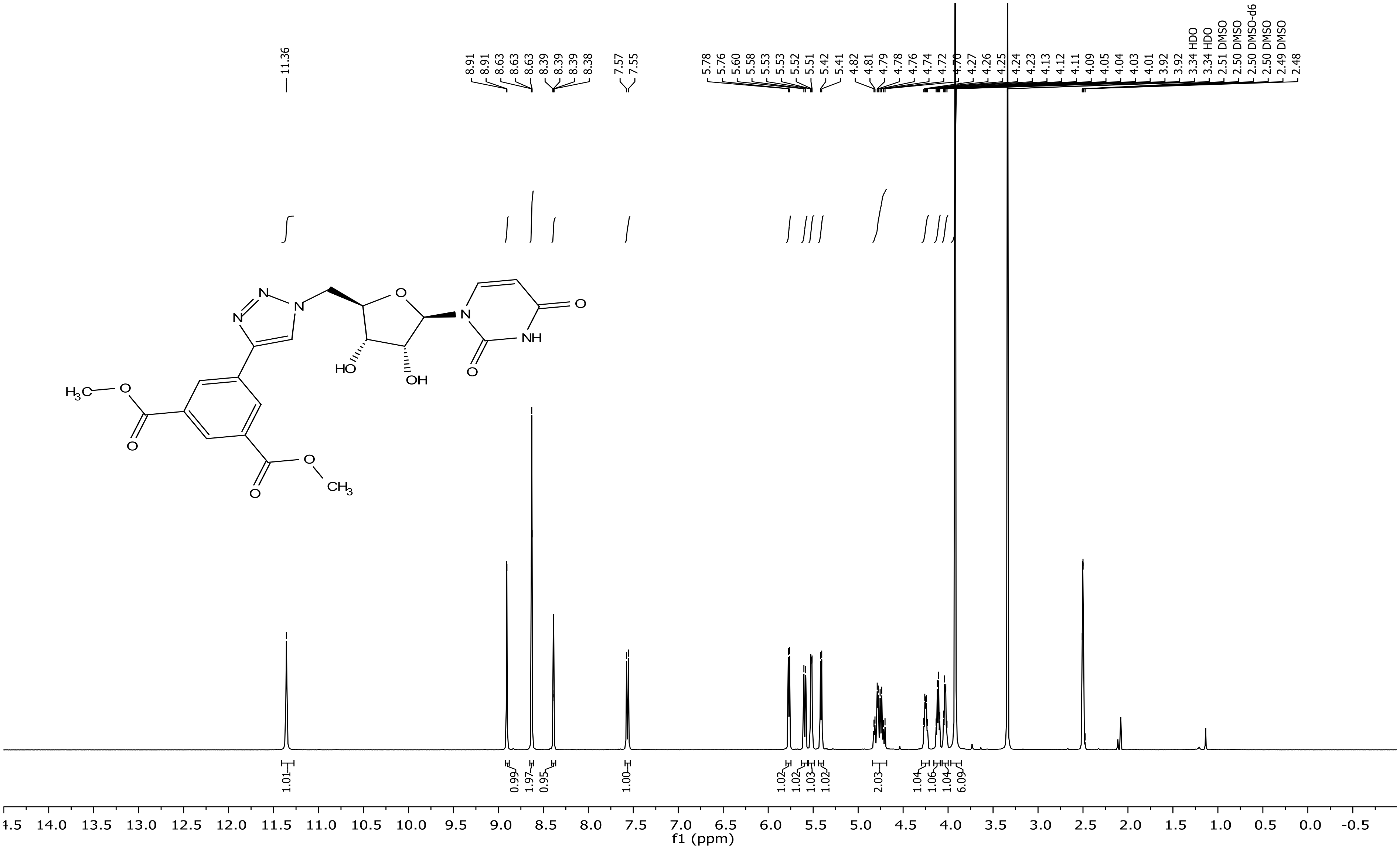

^{13}C NMR (DMSO-d6) of compound **3k**

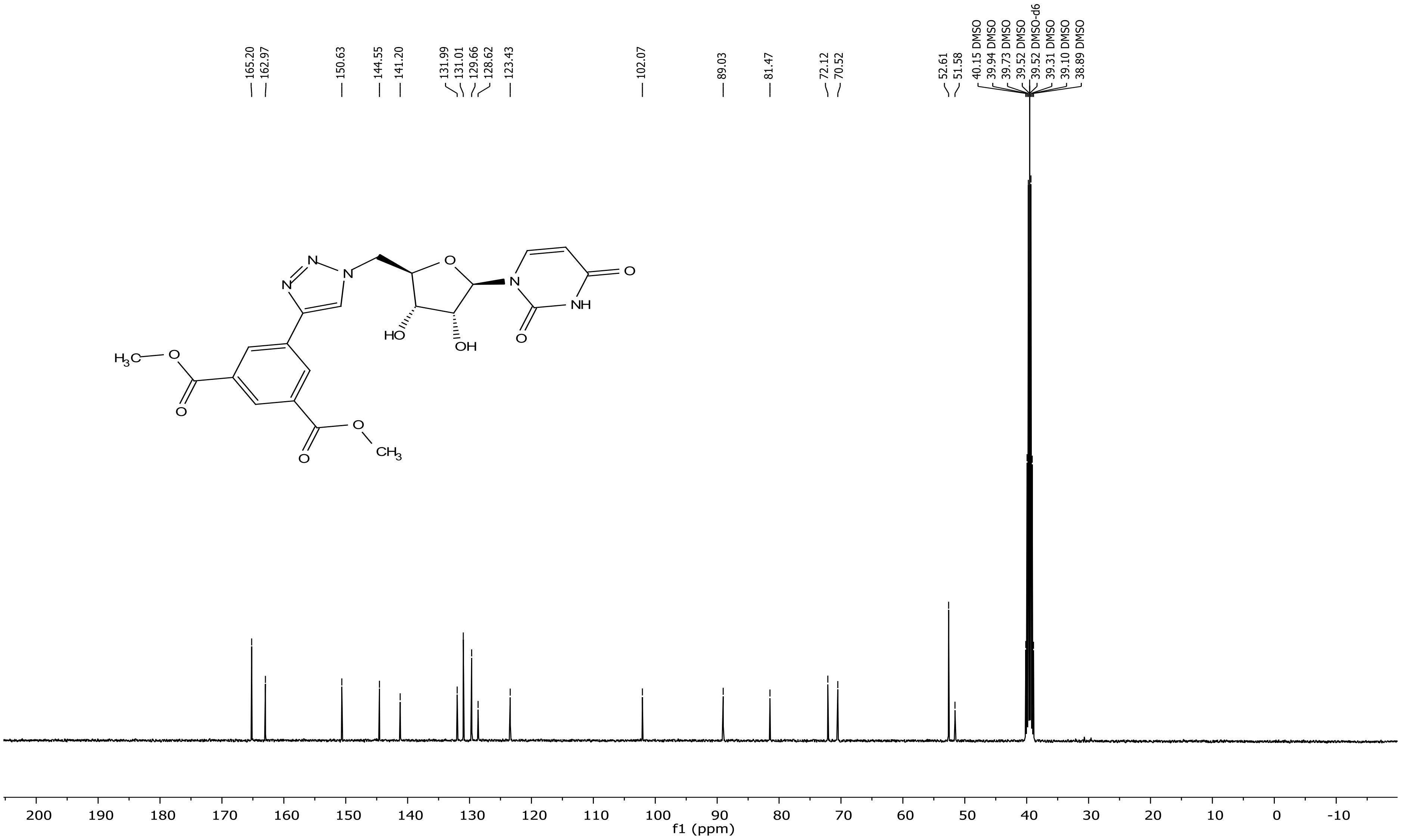

^{1}H-^{13}C HMBC NMR (DMSO-d6) of compound **3k**

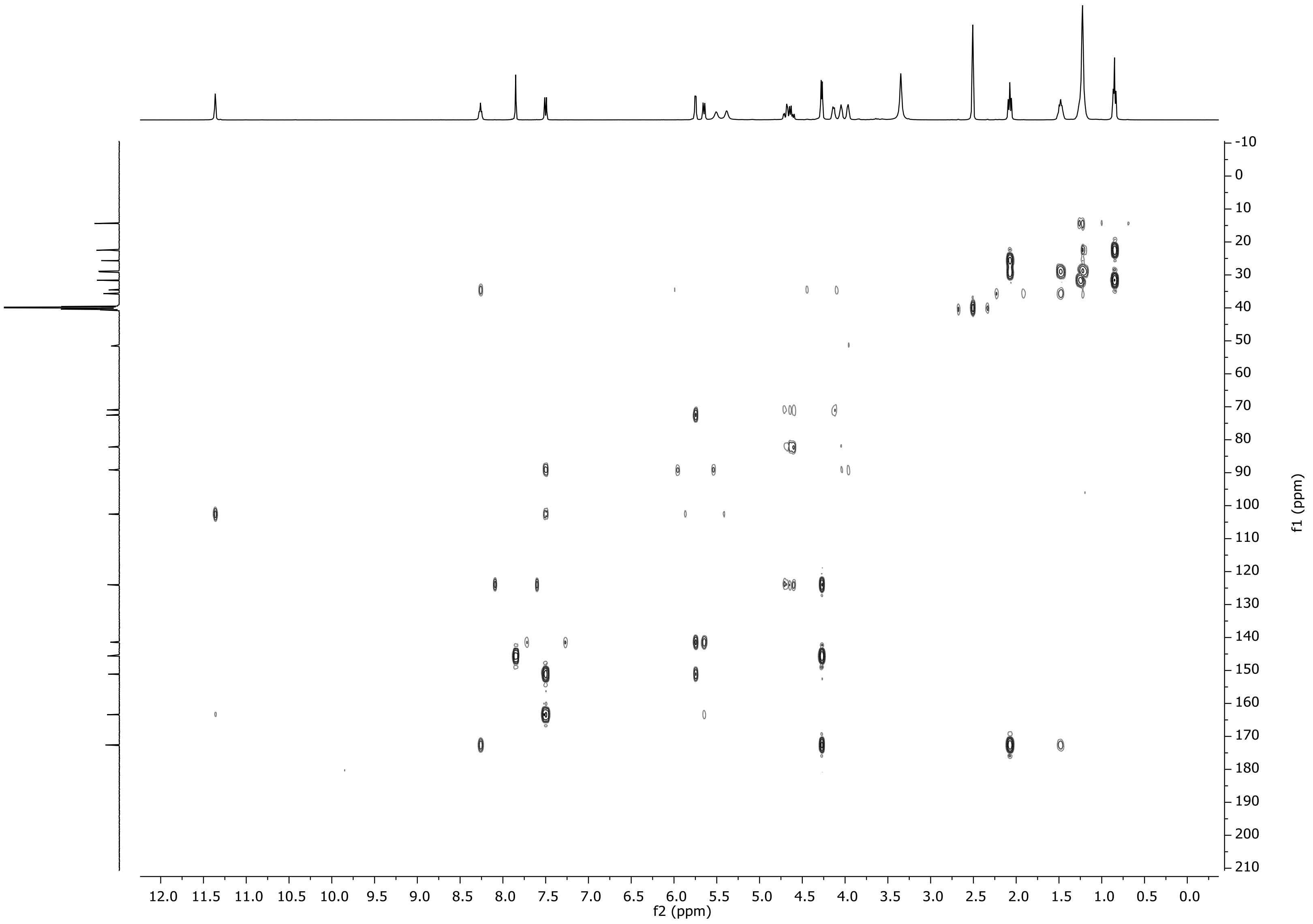

^{1}H NMR (DMSO-d6) of compound **3l**

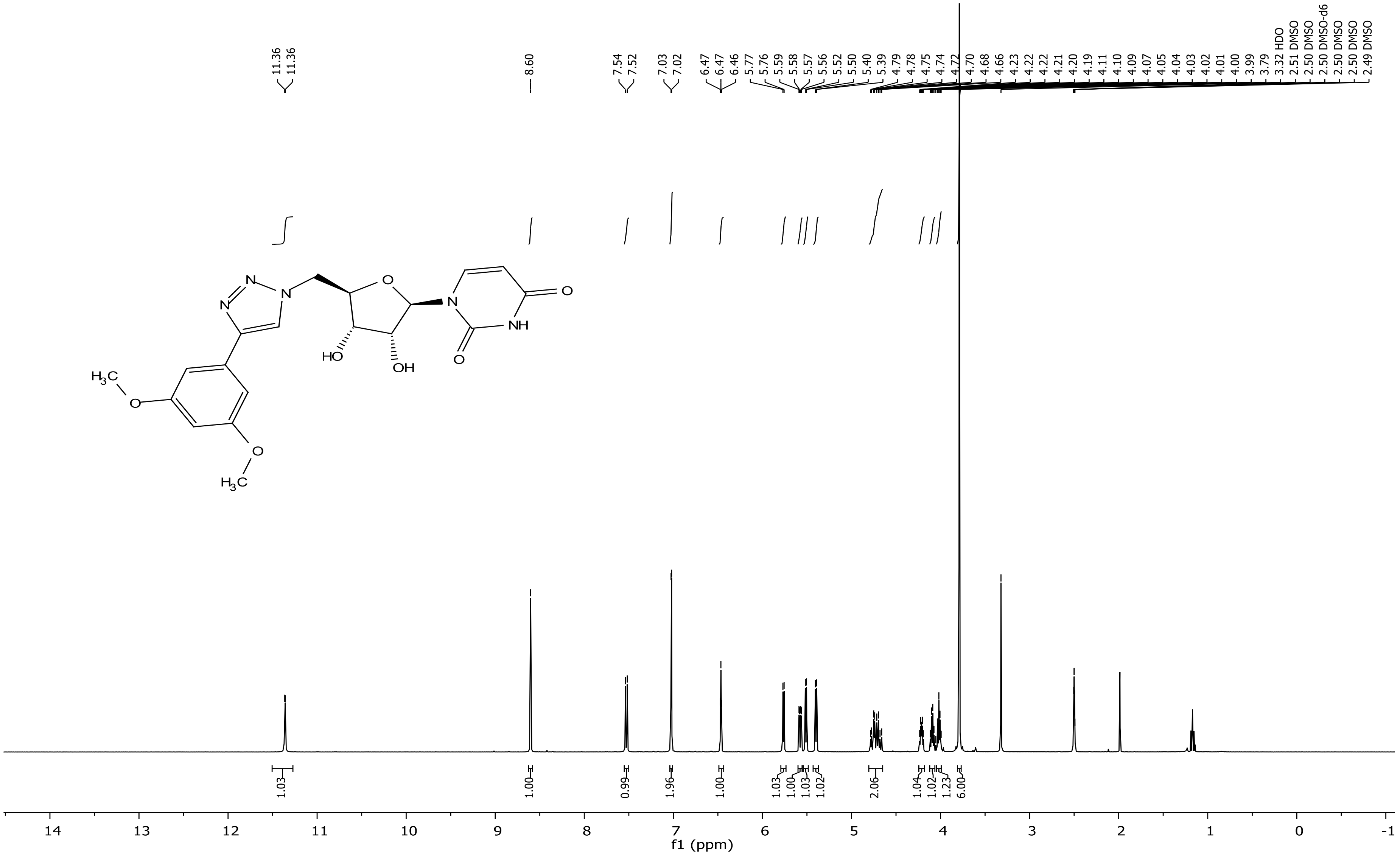

^{13}C NMR (DMSO-d6) of compound **3l**

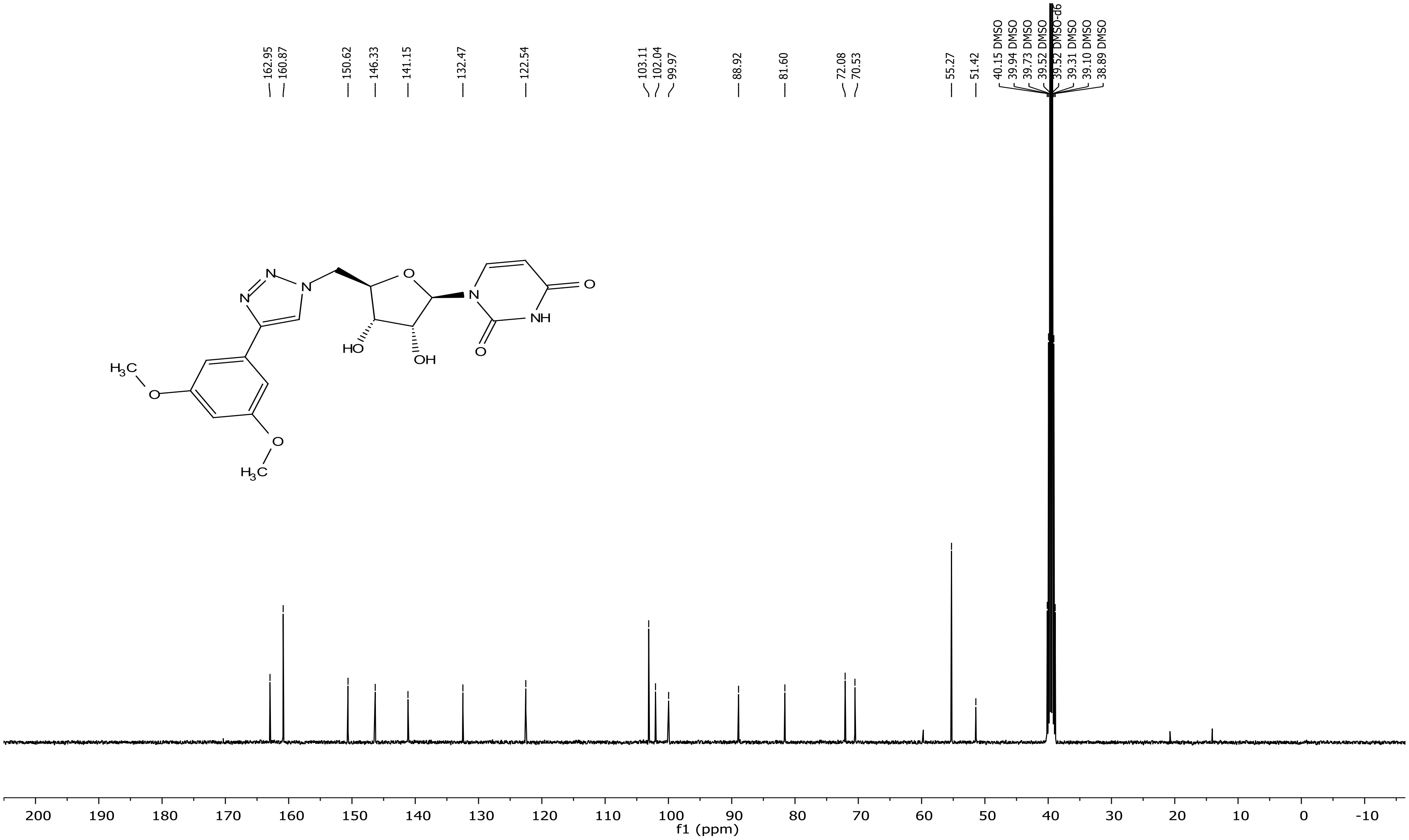

^{1}H-^{13}C HMBC NMR (DMSO-d6) of compound **3l**

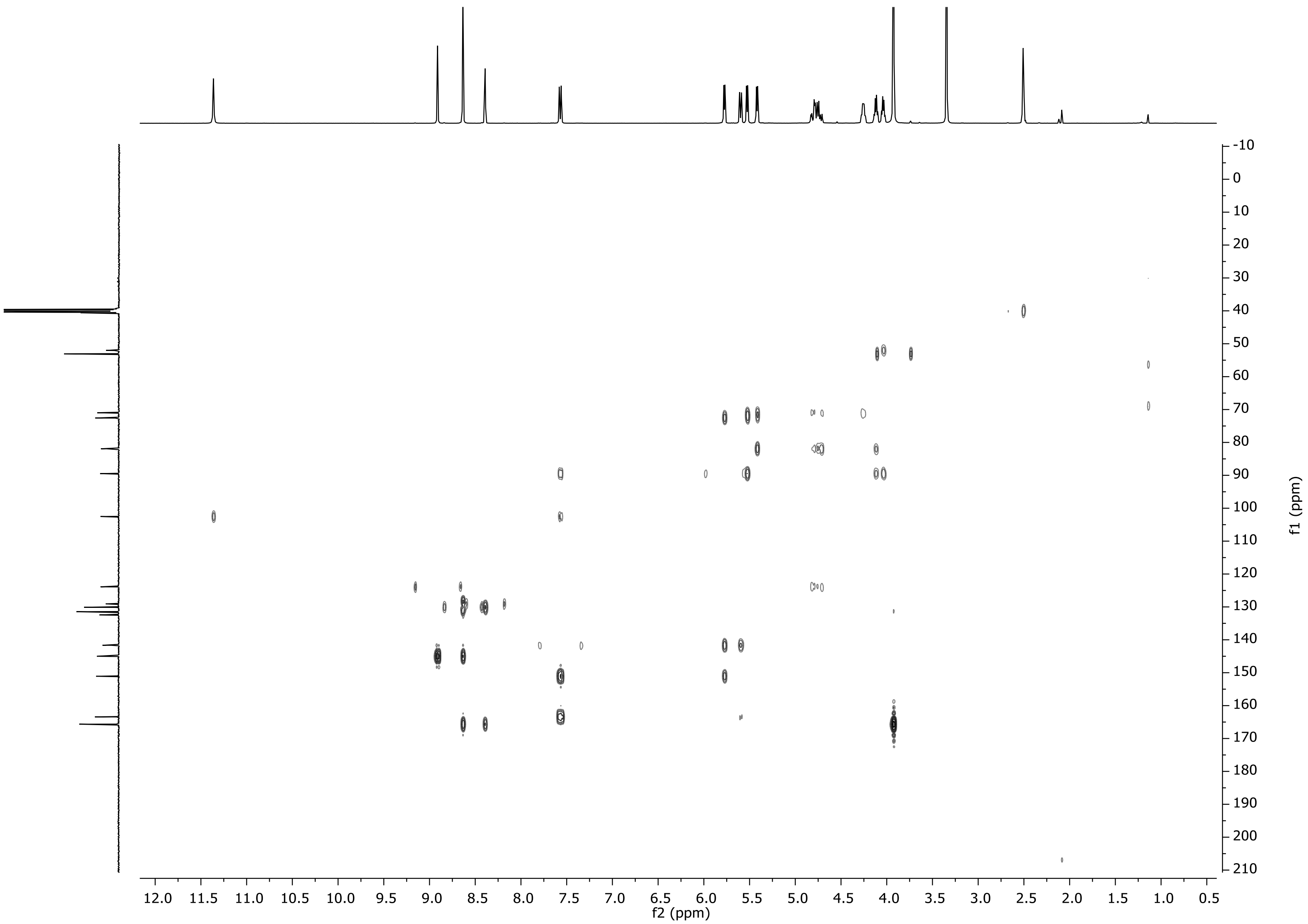

^{1}H NMR (DMSO-d6) of compound **3m**

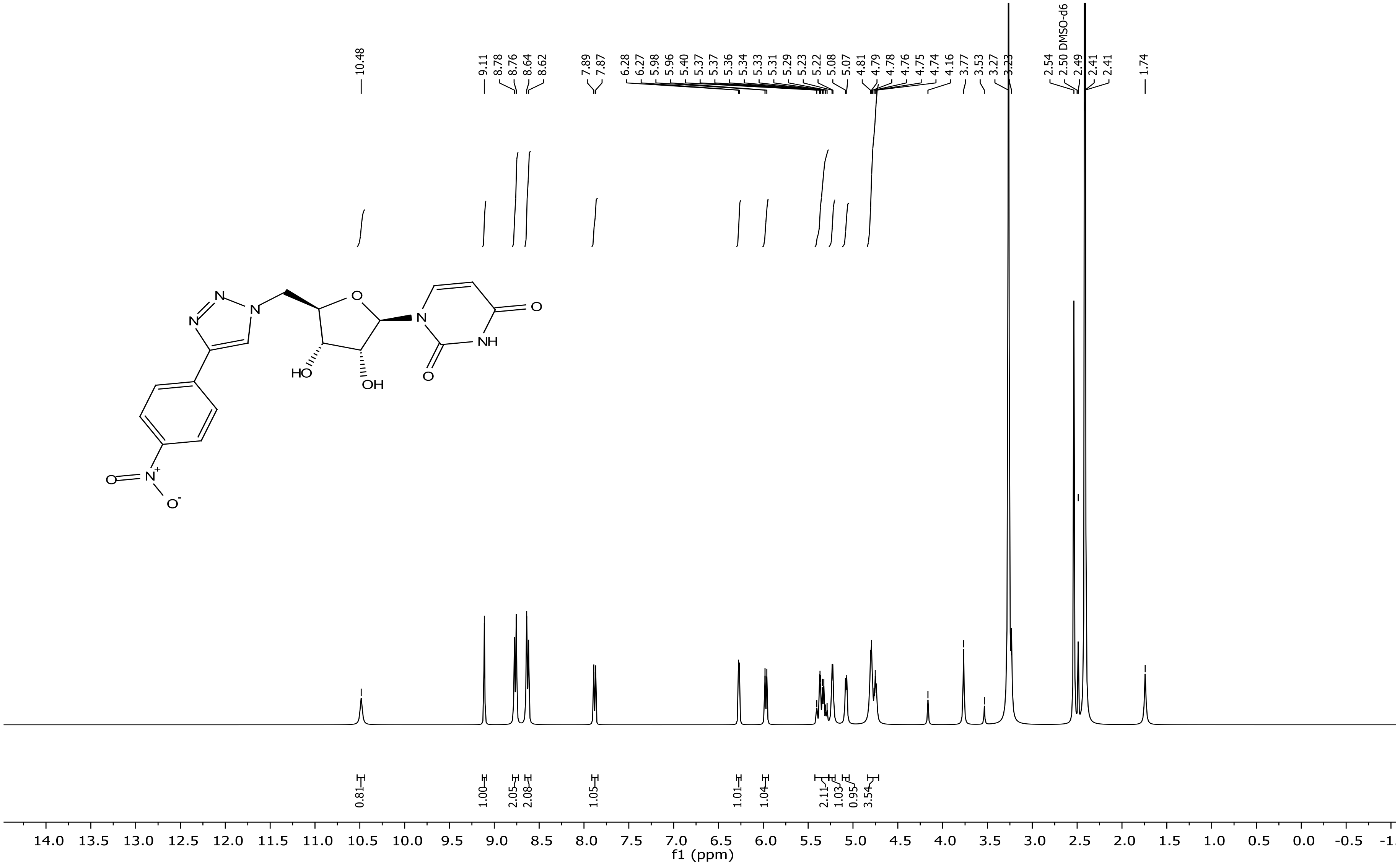

^{13}C NMR (DMSO-d6) of compound **3m**

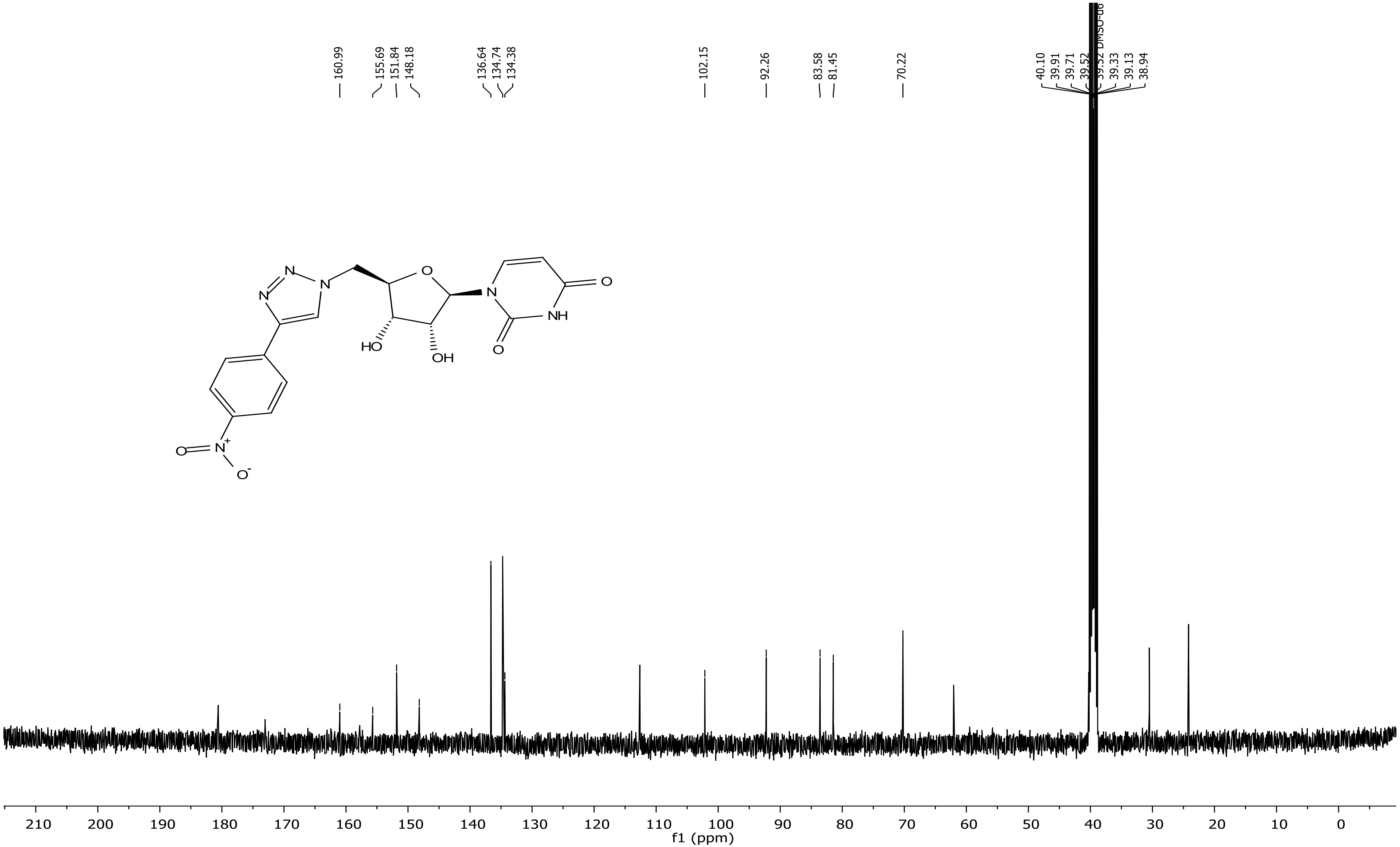

^{1}H-^{13}C HMBC NMR (DMSO-d6) of compound **3m**

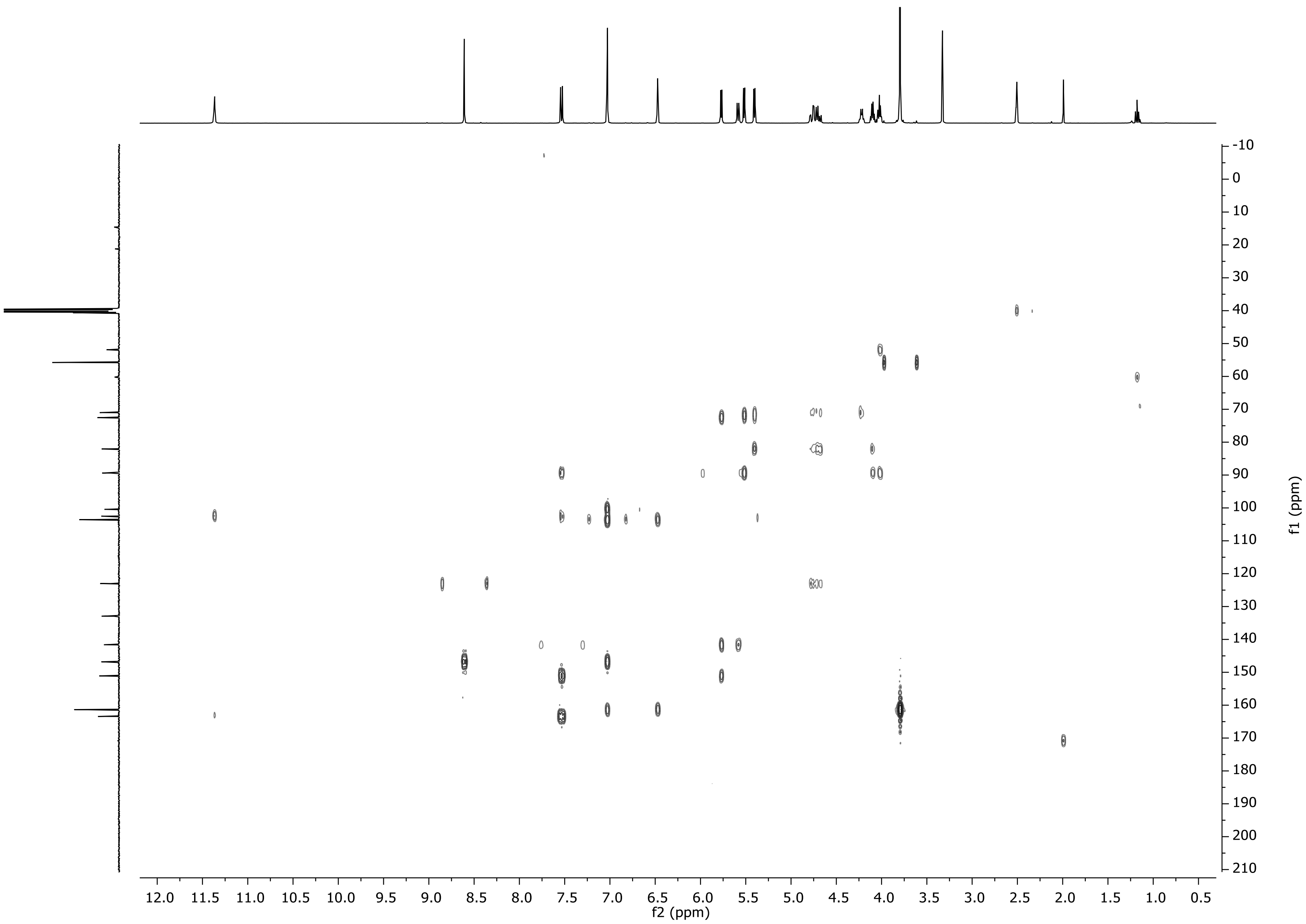

^{1}H NMR (DMSO-d6) of compound **3n**

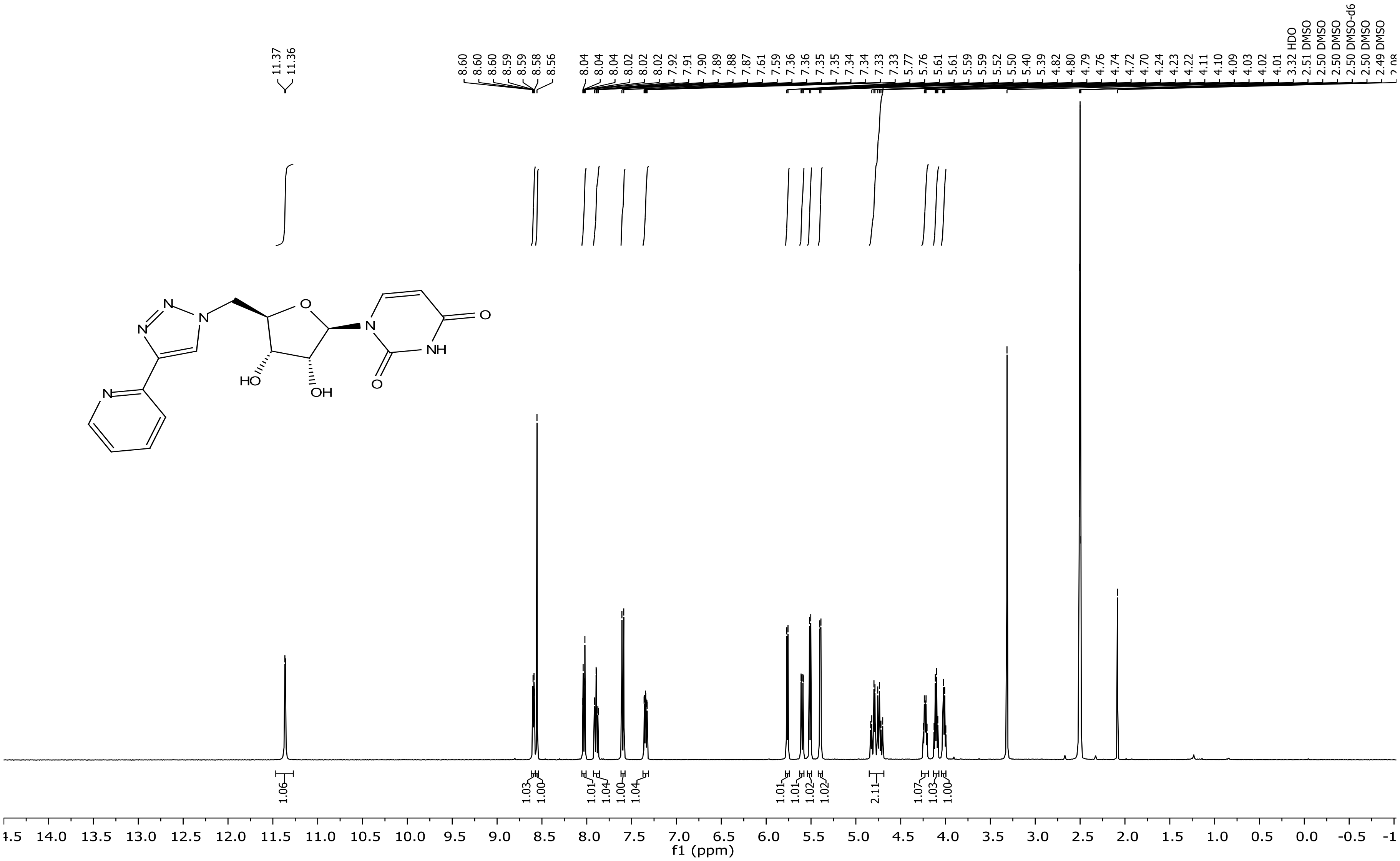

^{13}C NMR (DMSO-d6) of compound **3n**

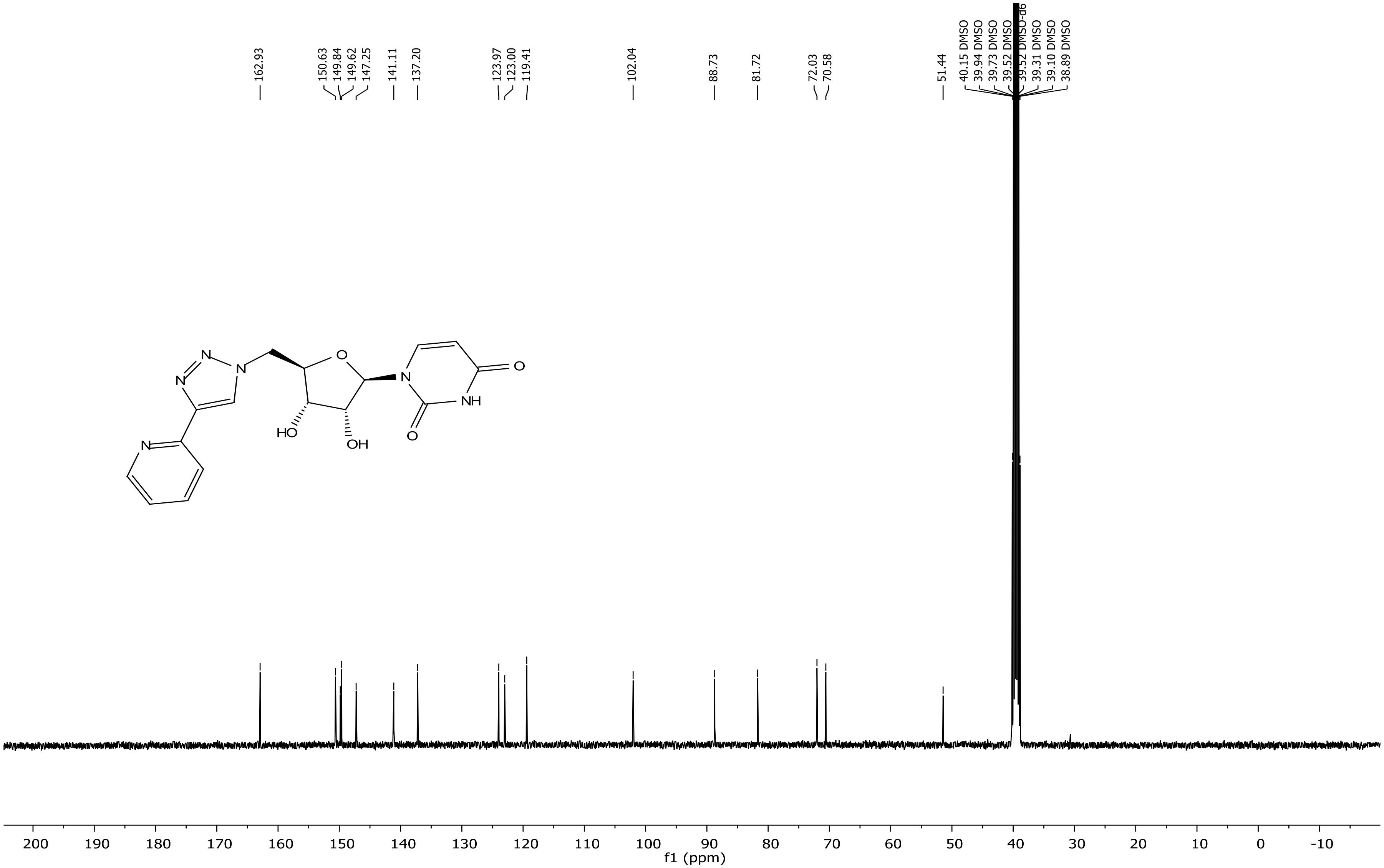

^{1}H-^{13}C HMBC NMR (DMSO-d6) of compound **3n**

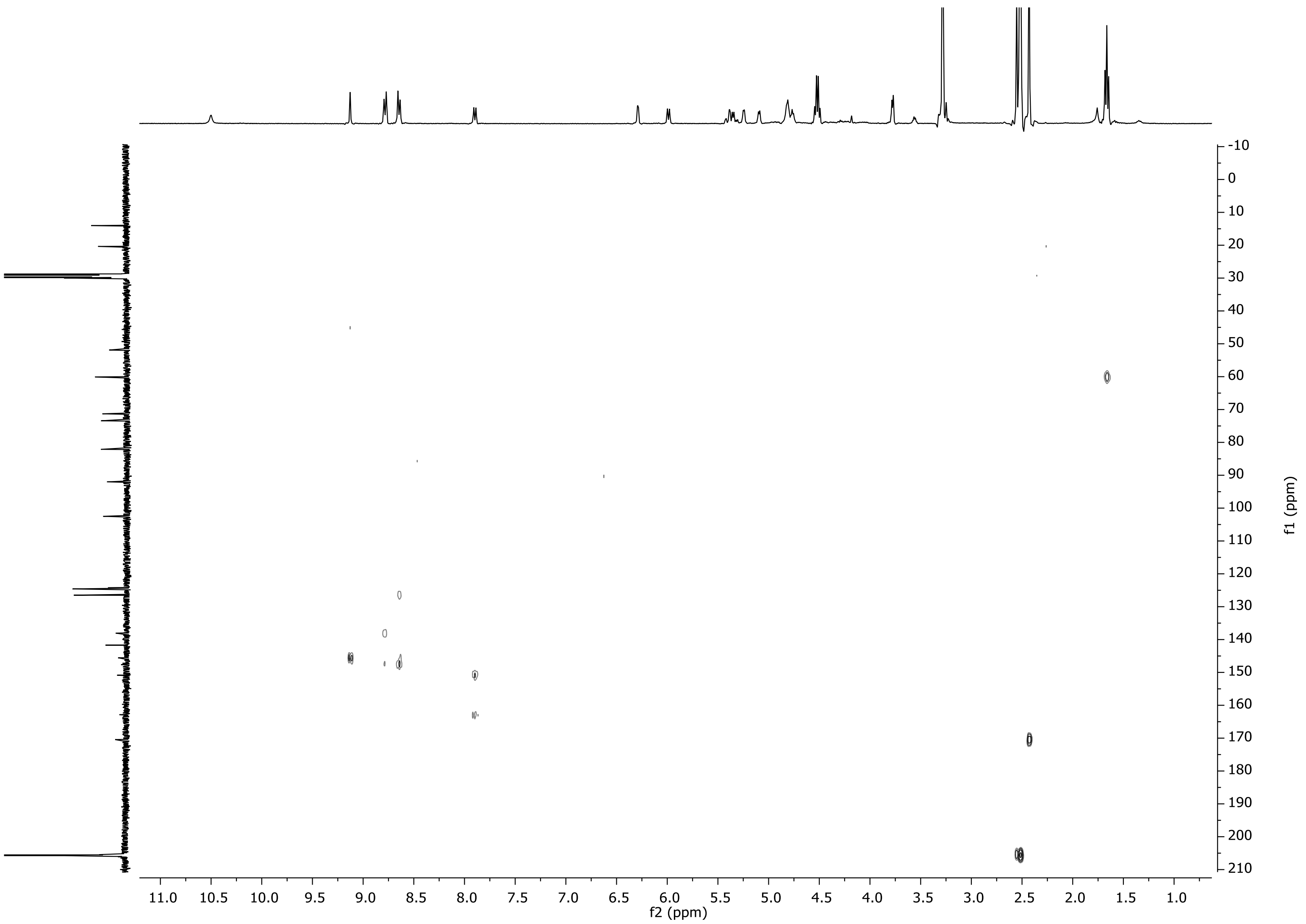

^{1}H NMR (DMSO-d6) of compound **3o**

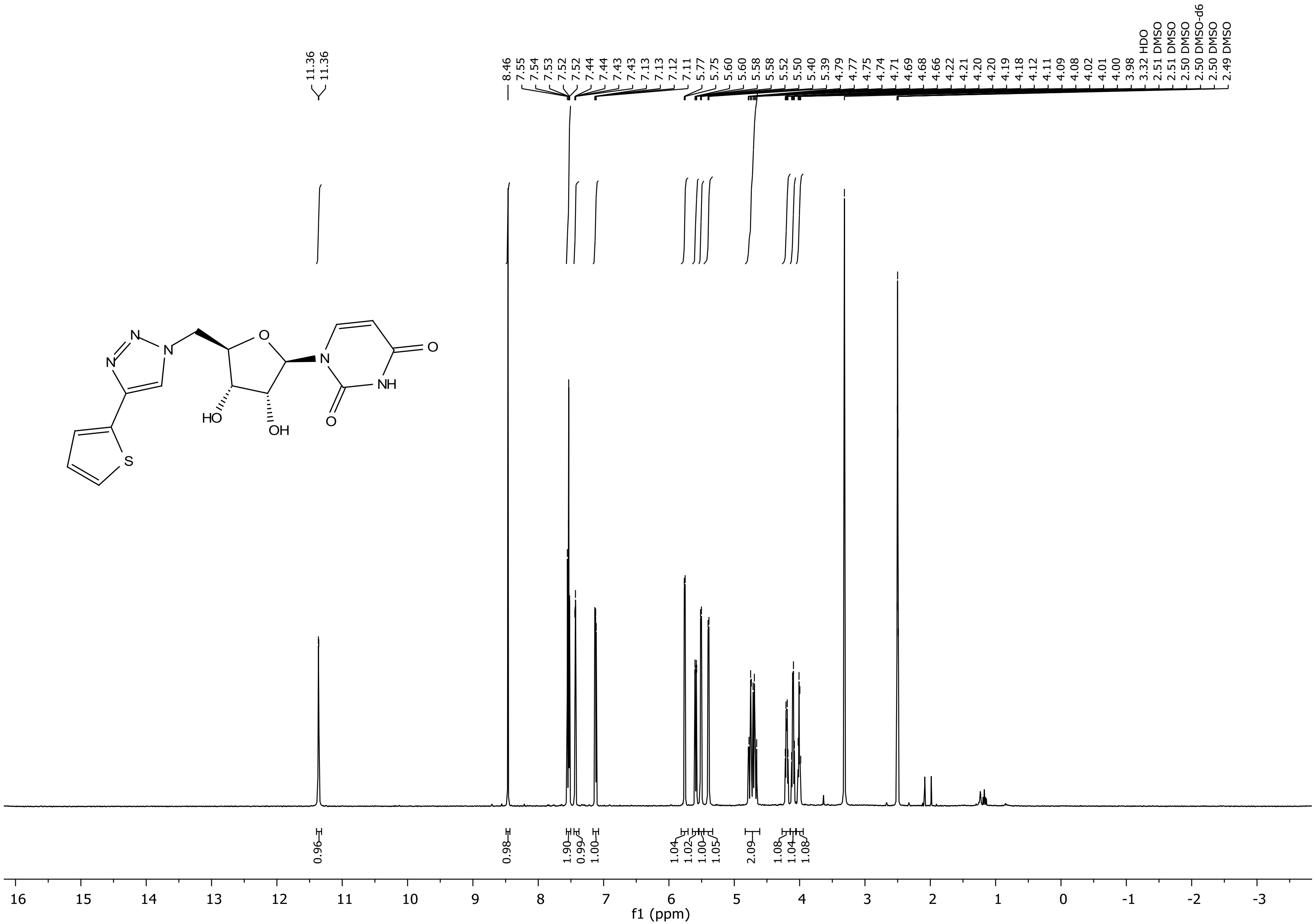

^{13}C NMR (DMSO-d6) of compound **3o**

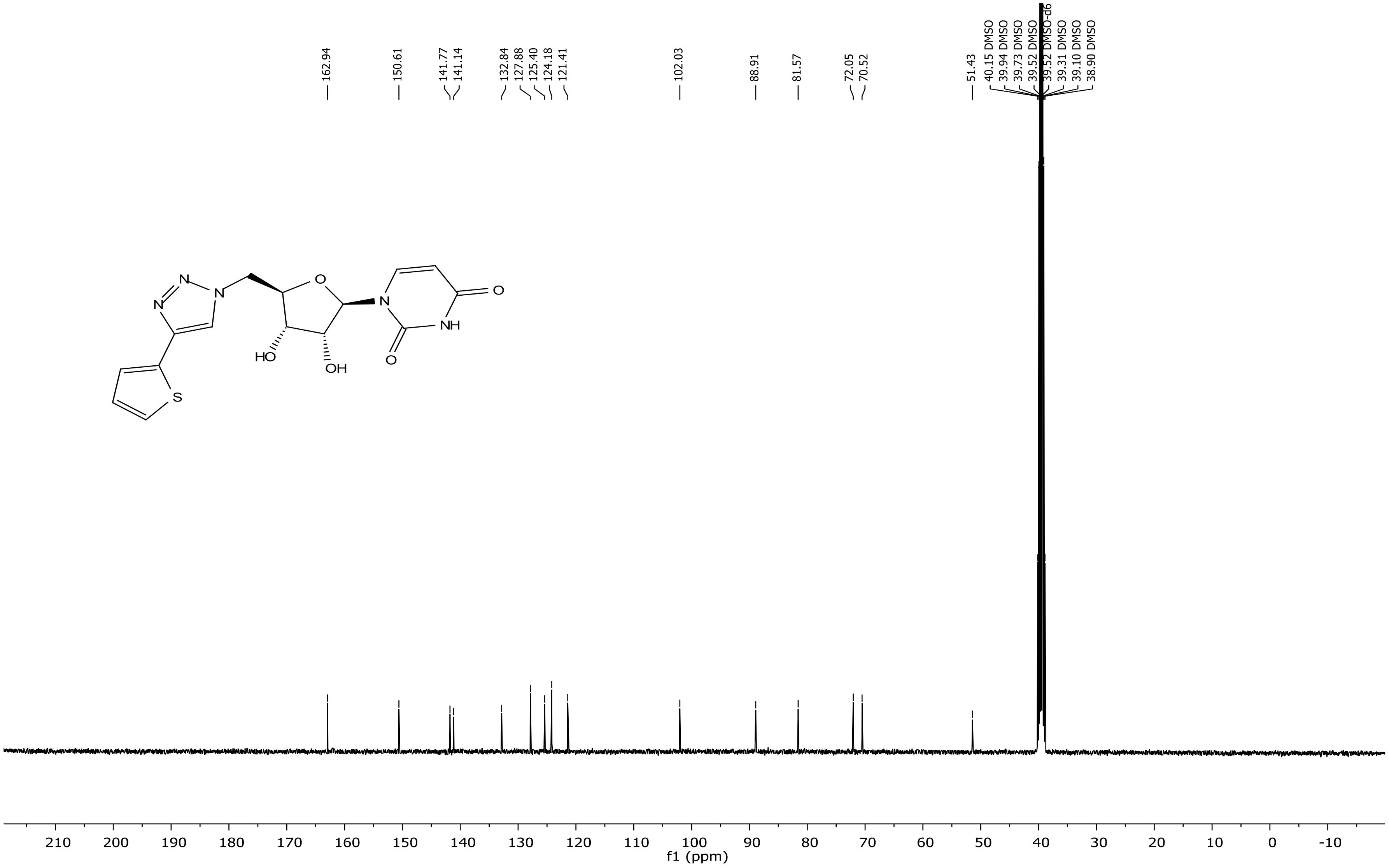

^{1}H-^{13}C HMBC NMR (DMSO-d6) of compound **3o**

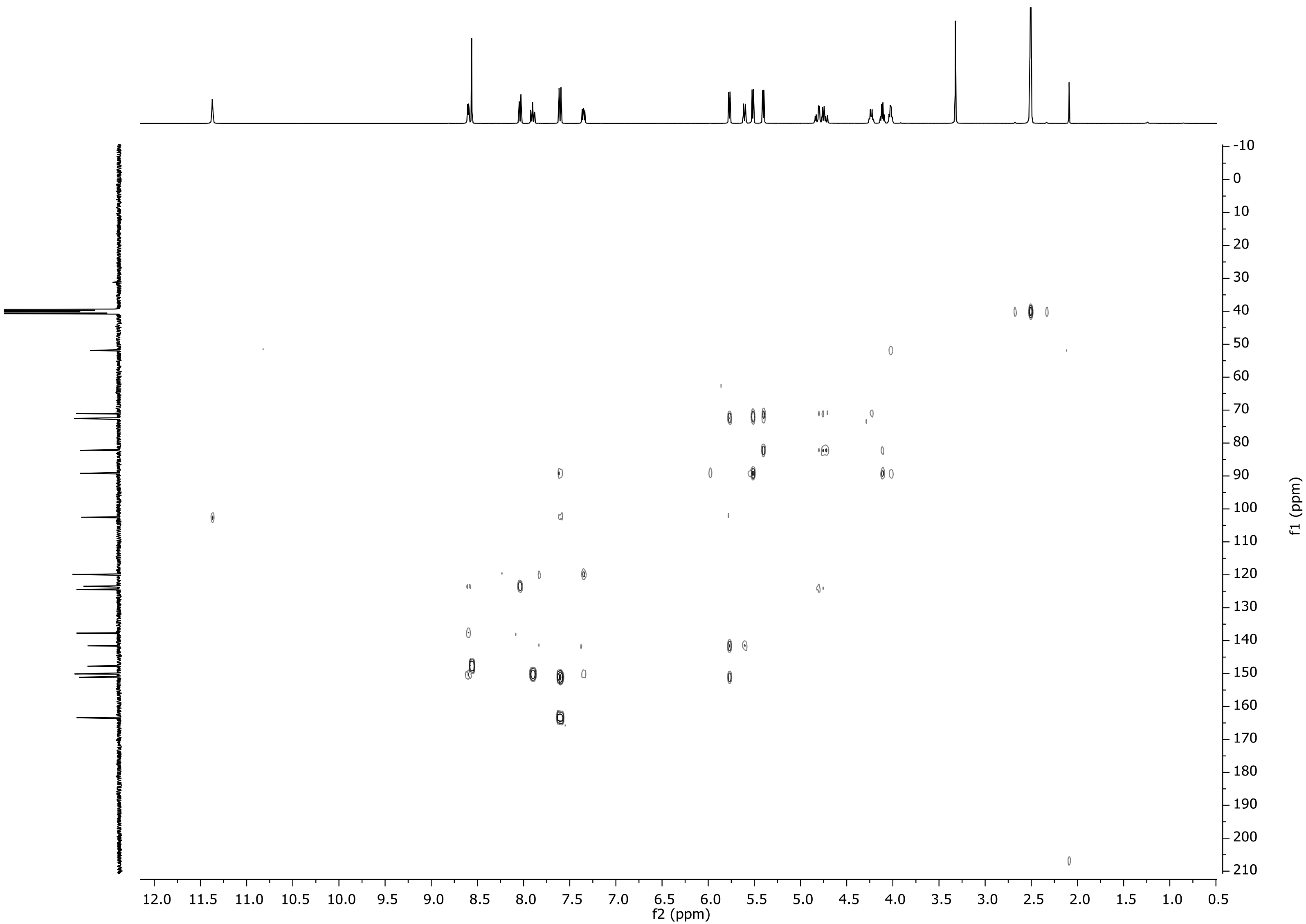

^{1}H NMR (DMSO-d6) of compound **3p**

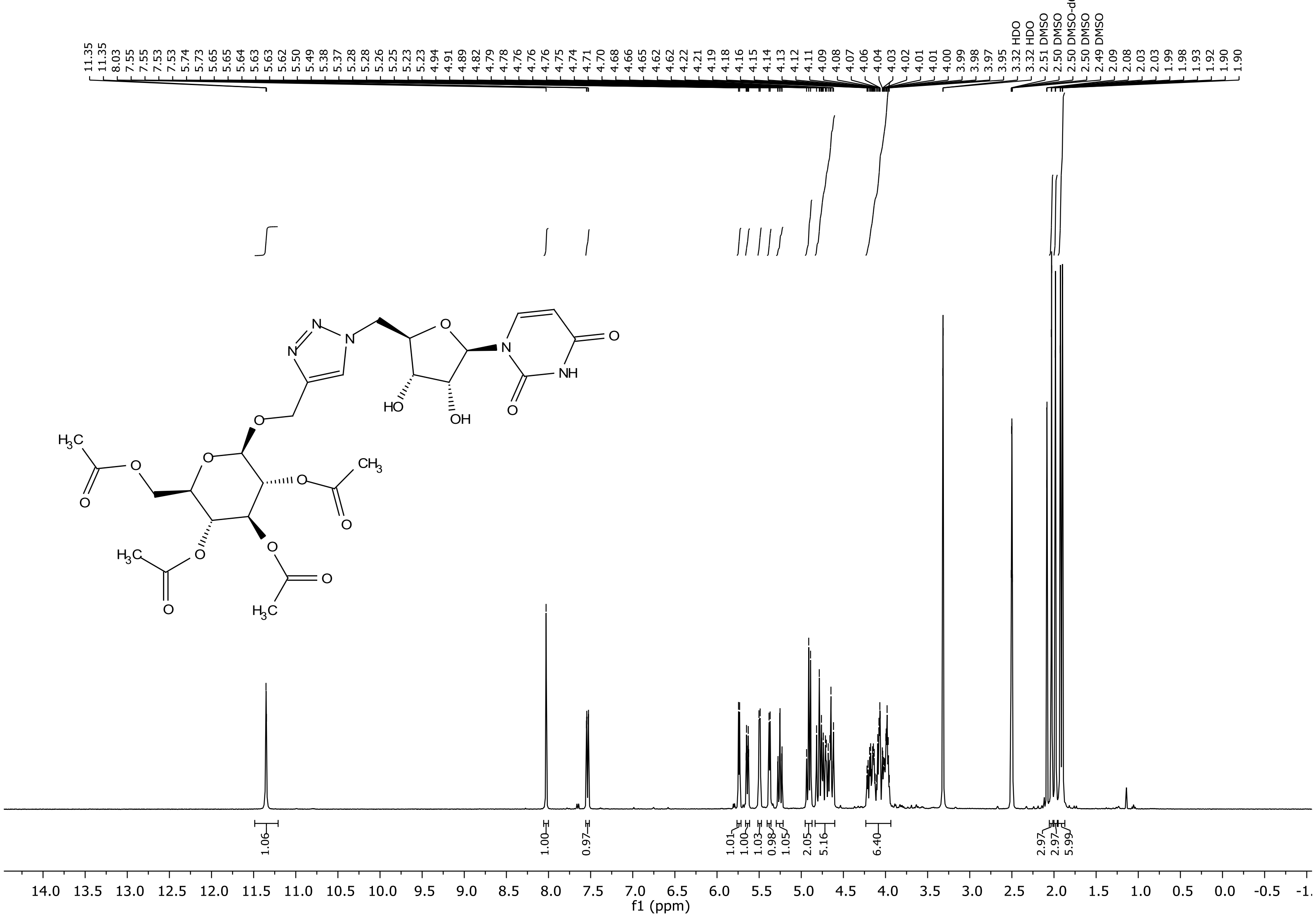

^{13}C NMR (DMSO-d6) of compound **3p**

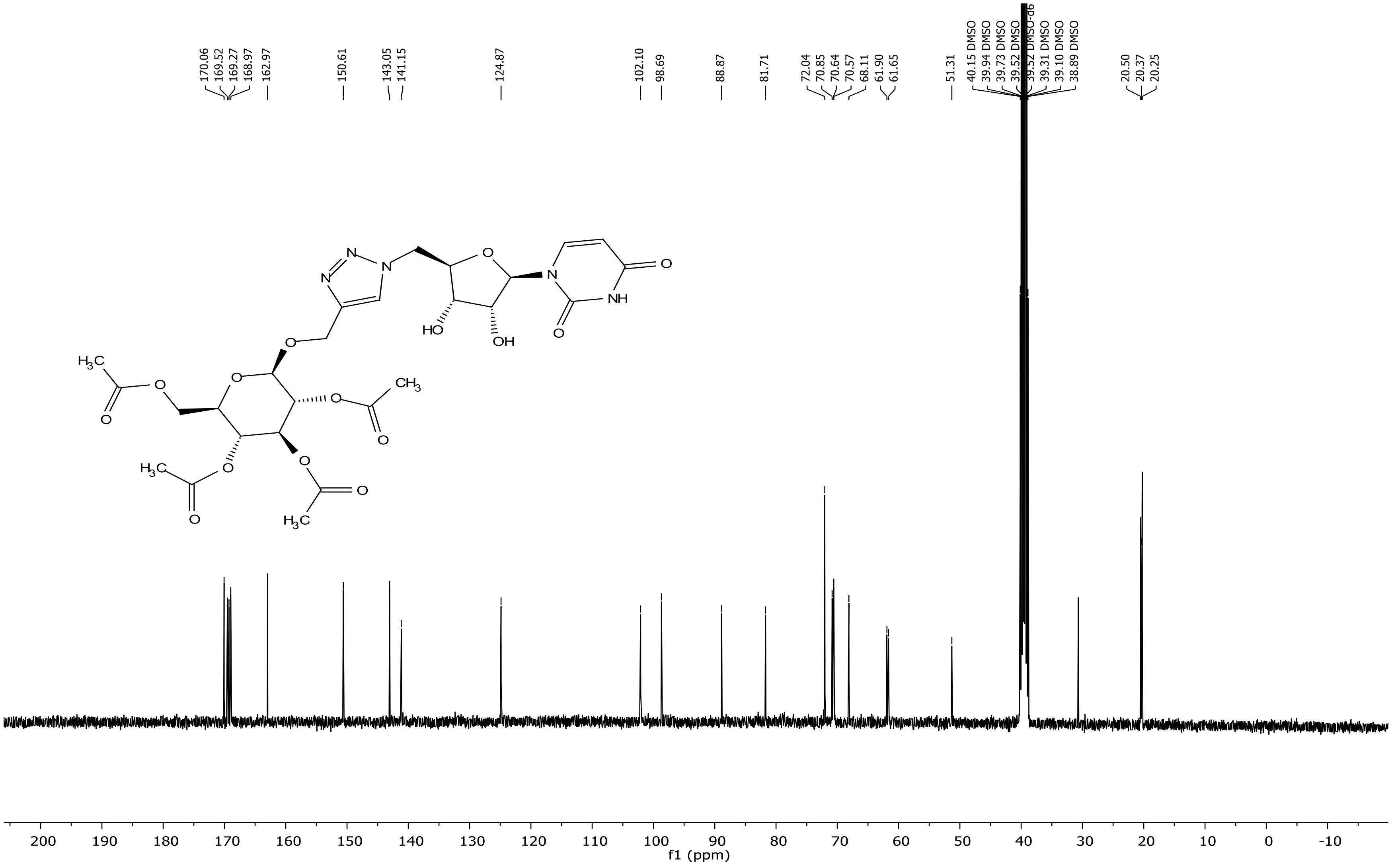

^{1}H-^{13}C HMBC NMR (DMSO-d6) of compound **3p**

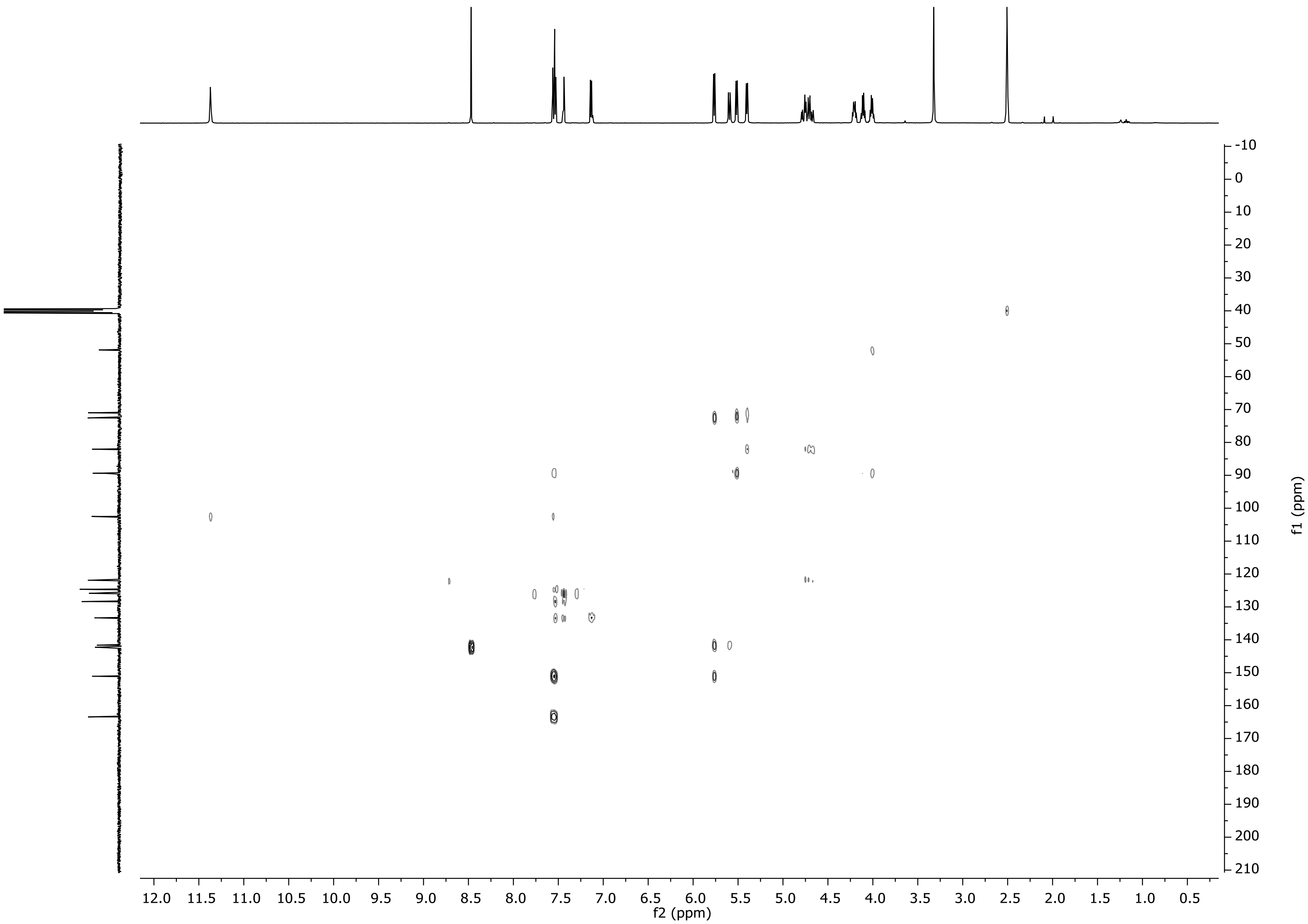

^{1}H NMR (DMSO-d6) of compound **4**

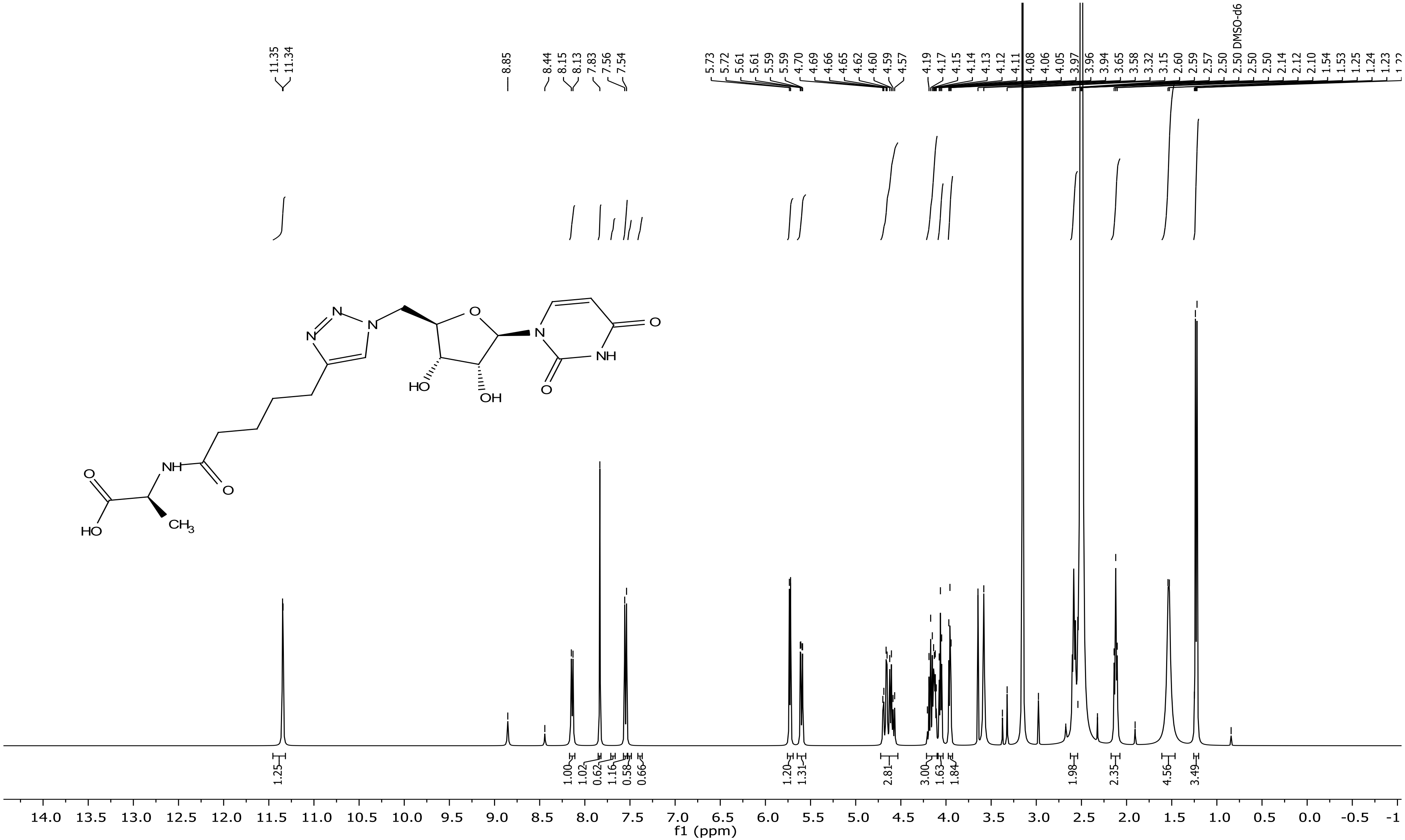

^{13}C NMR (DMSO-d6) of compound **4**

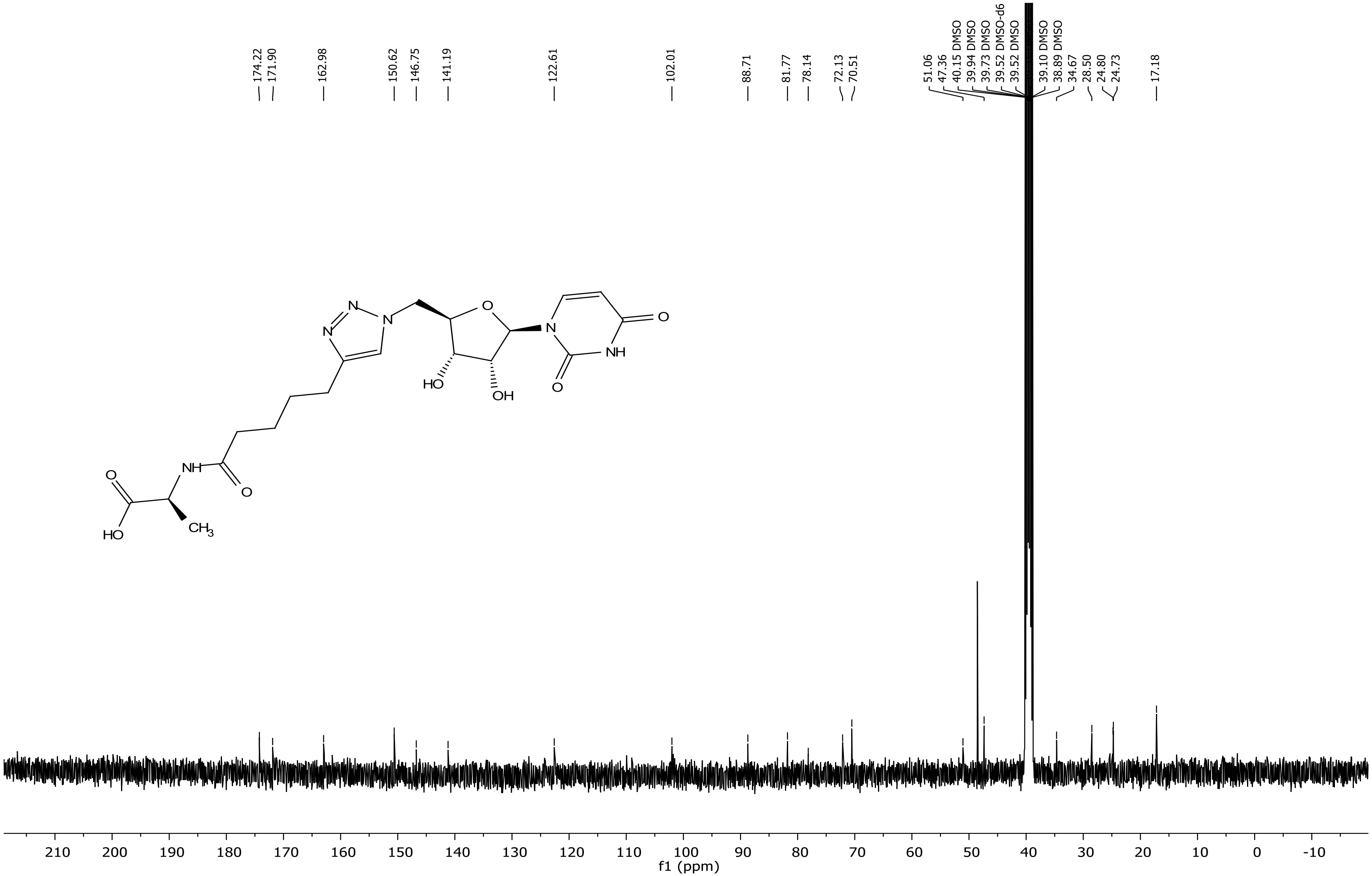

^{1}H-^{13}C HMBC NMR (DMSO-d6) of compound **4**

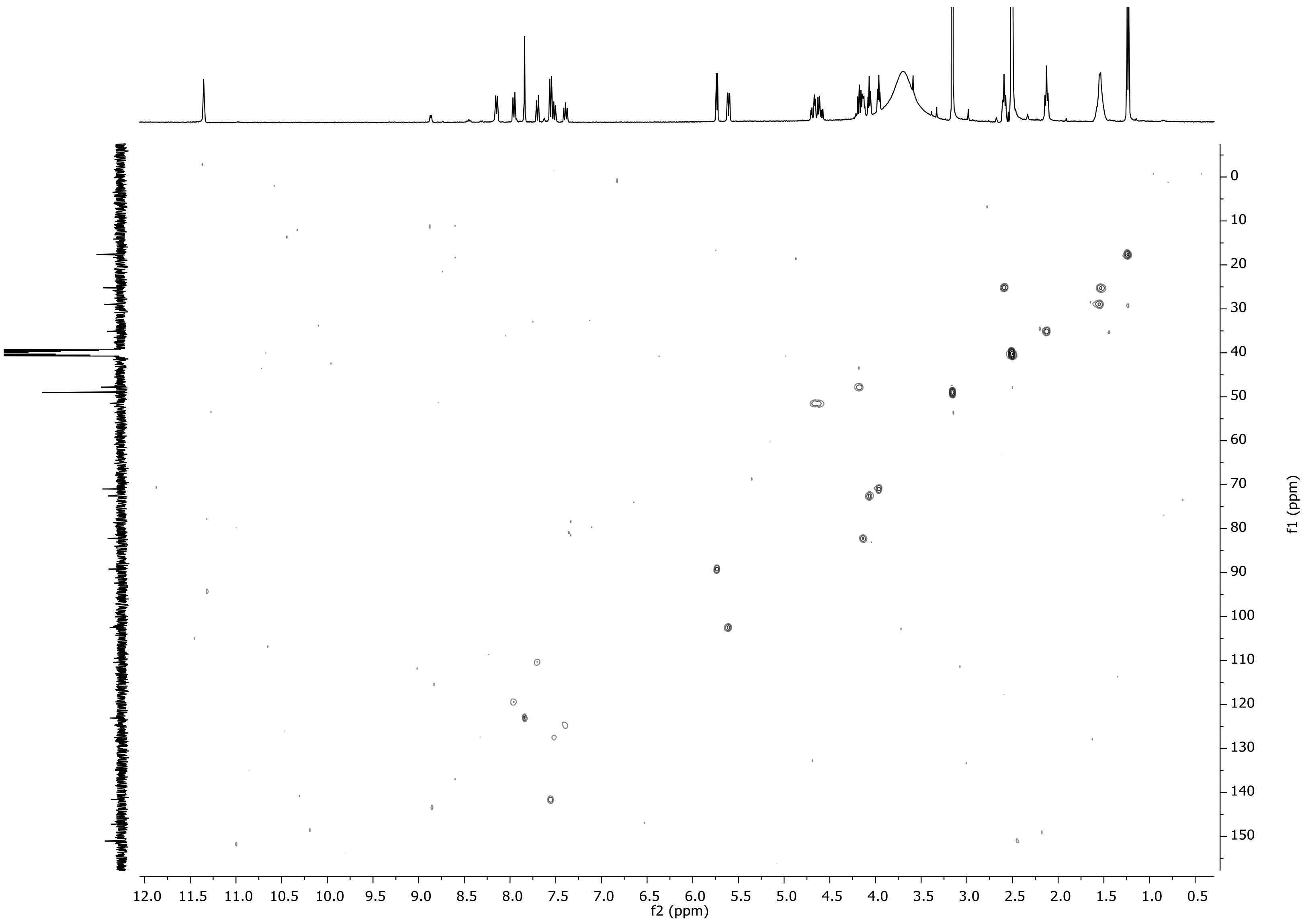

^{1}H NMR (DMSO-d6) of compound **6**

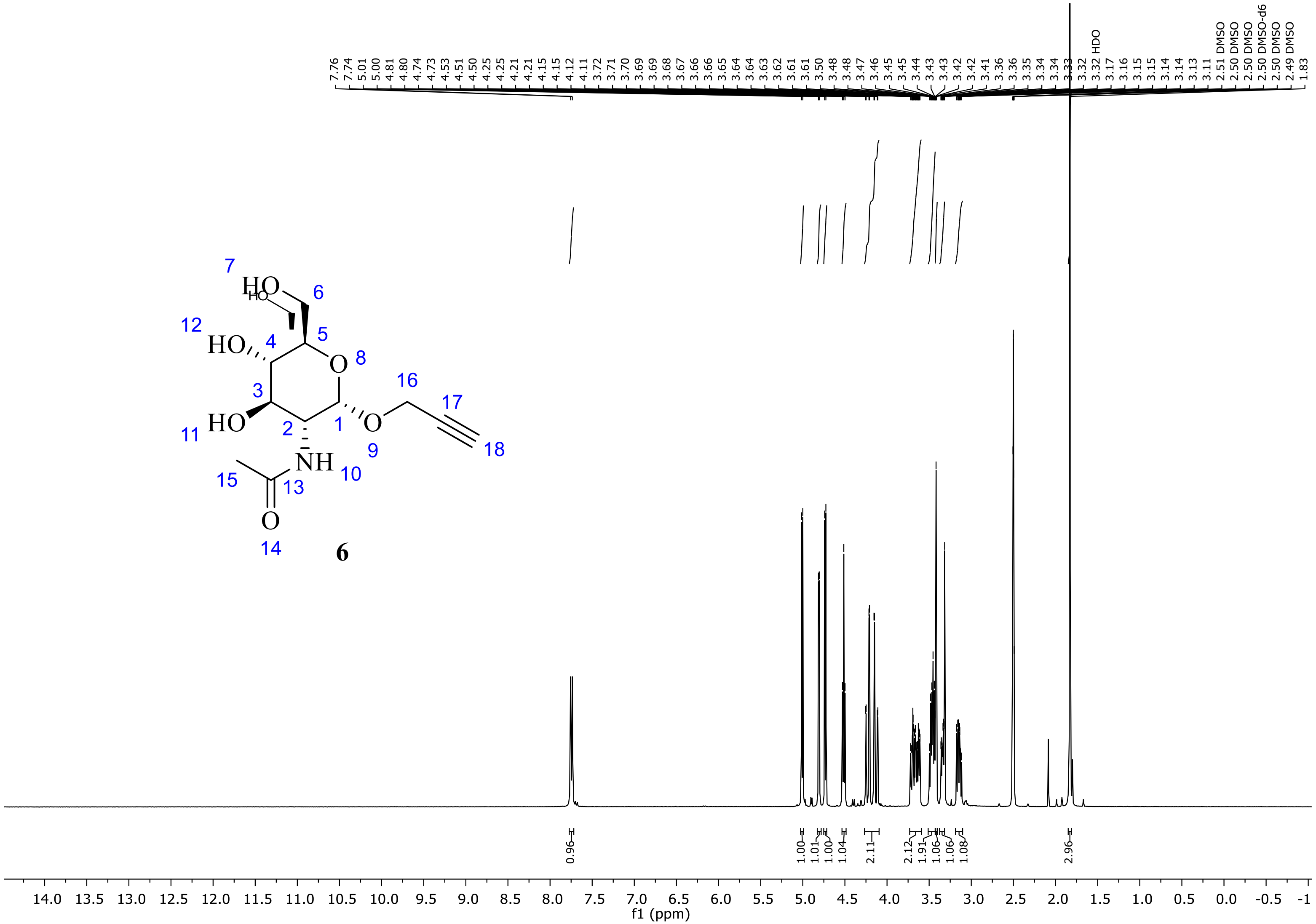

7.76 7.74 5.01 5.00 4.81 4.80 4.74 4.73 4.53 4.51 4.50 4.25 4.25 4.21 4.21 4.15 4.15 4.12 4.11 3.72 3.71 3.70 3.69 3.69 3.68 3.67 3.66 3.66 3.65 3.64 3.64 3.63 3.62 3.61 3.61 3.50 3.48 3.48 3.47 3.46 3.45 3.44 3.43 3.43 3.42 3.42 3.41 3.36 3.36 3.35 3.34 3.34 3.33 3.32
3.32 HDO
3.17 3.16 3.15 3.15 3.14 3.14 3.13 3.11
2.51 DMSO
2.50 DMSO
2.50 DMSO
2.50 DMSO-d6
2.50 DMSO
2.49 DMSO
1.83
HO
HO
HO
HO
O
O
NH
O
7 6 12 4 5 8 16 17 3 2 1 11 9 18 15 10 13 14
6
0.96 1.00 1.01 1.00 1.04 2.11 2.12 1.91 1.06 1.08 2.96
14.0 13.5 13.0 12.5 12.0 11.5 11.0 10.5 10.0 9.5 9.0 8.5 8.0 7.5 7.0 6.5 6.0 5.5 5.0 4.5 4.0 3.5 3.0 2.5 2.0 1.5 1.0 0.5 0.0 -0.5 -1
f1 (ppm)

^{13}C NMR (DMSO-d6) of compound **6**

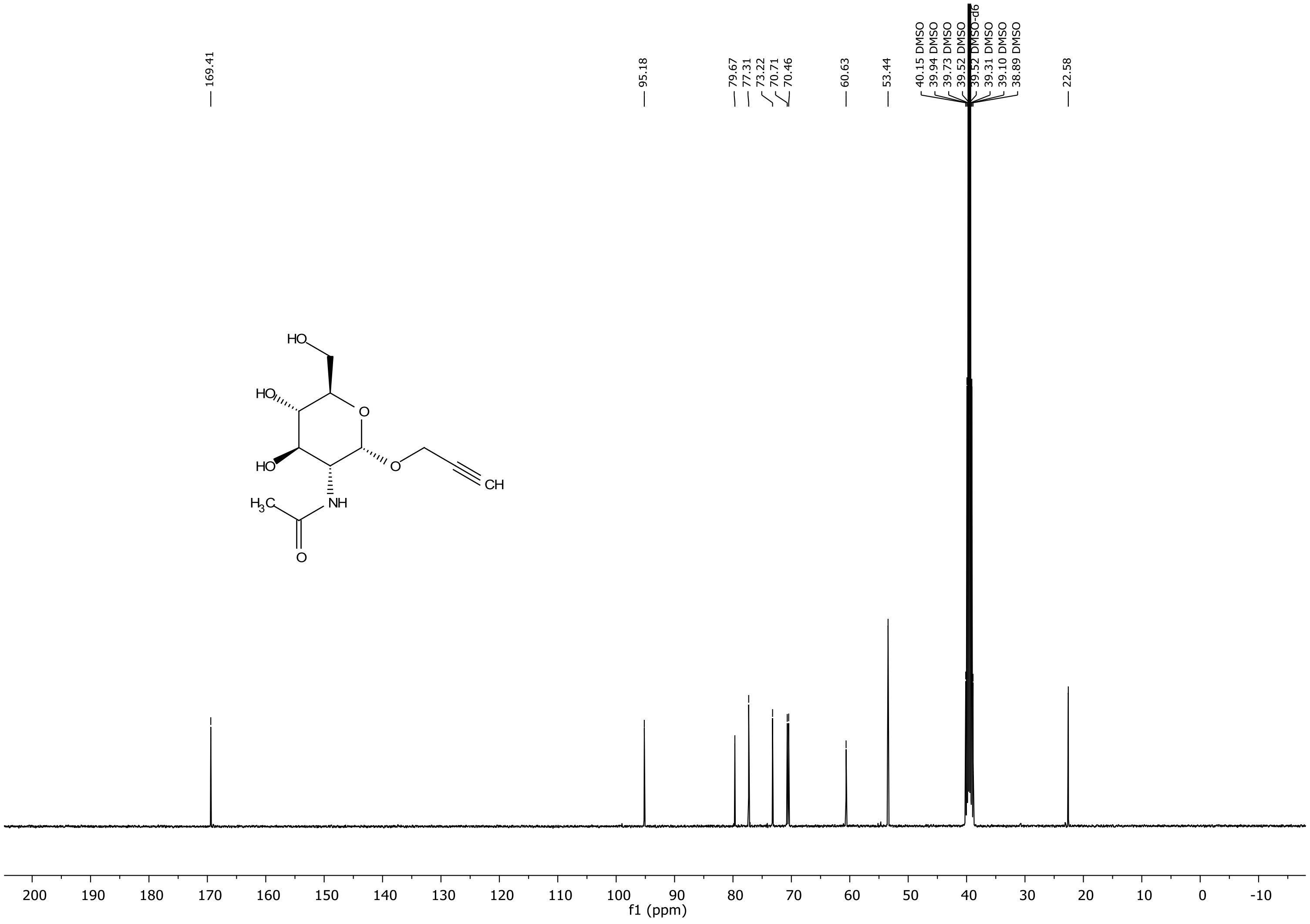

^{1}H NMR (DMSO-d6) of compound **7**

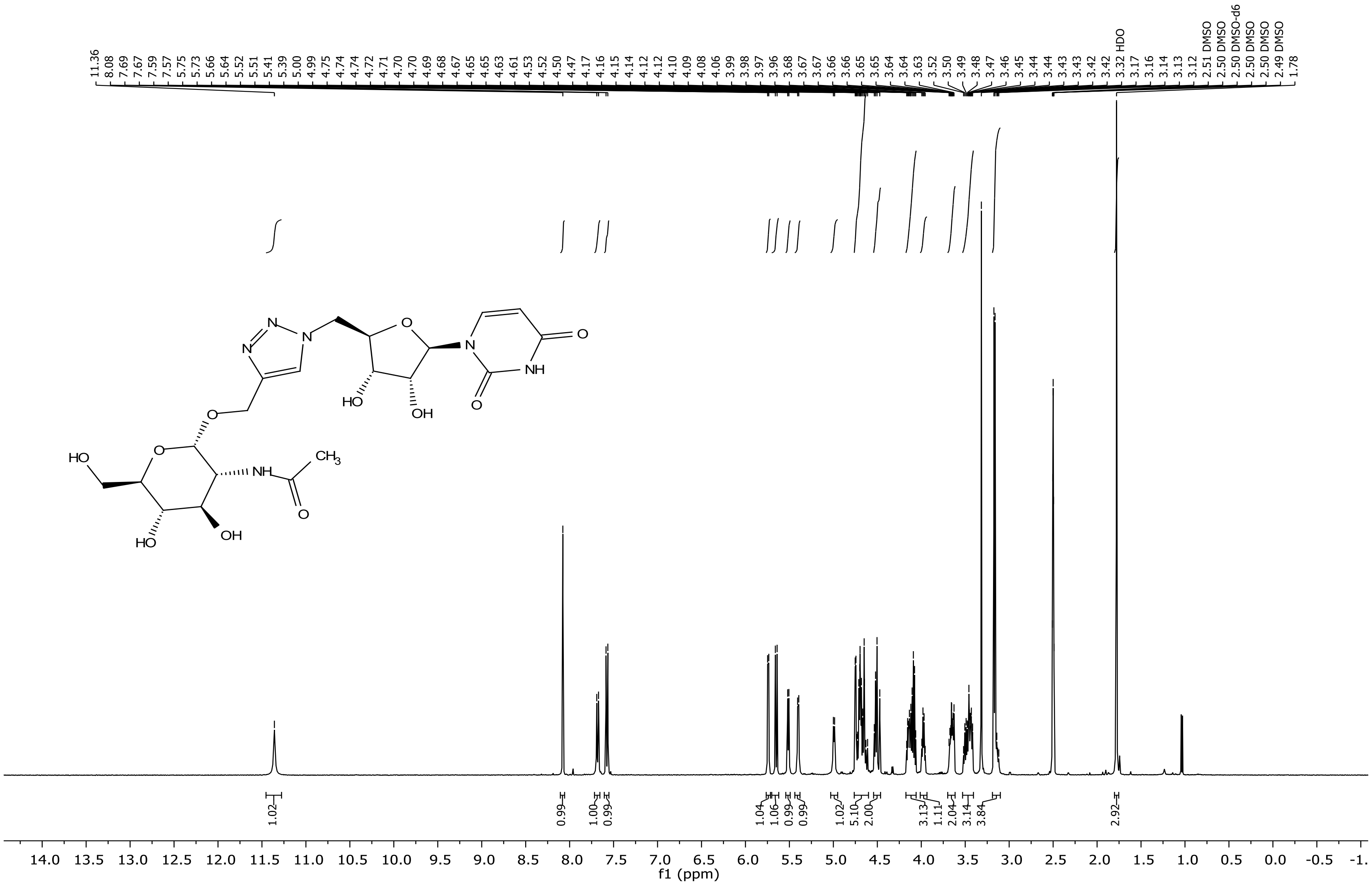

^{13}C NMR (DMSO-d6) of compound **7**

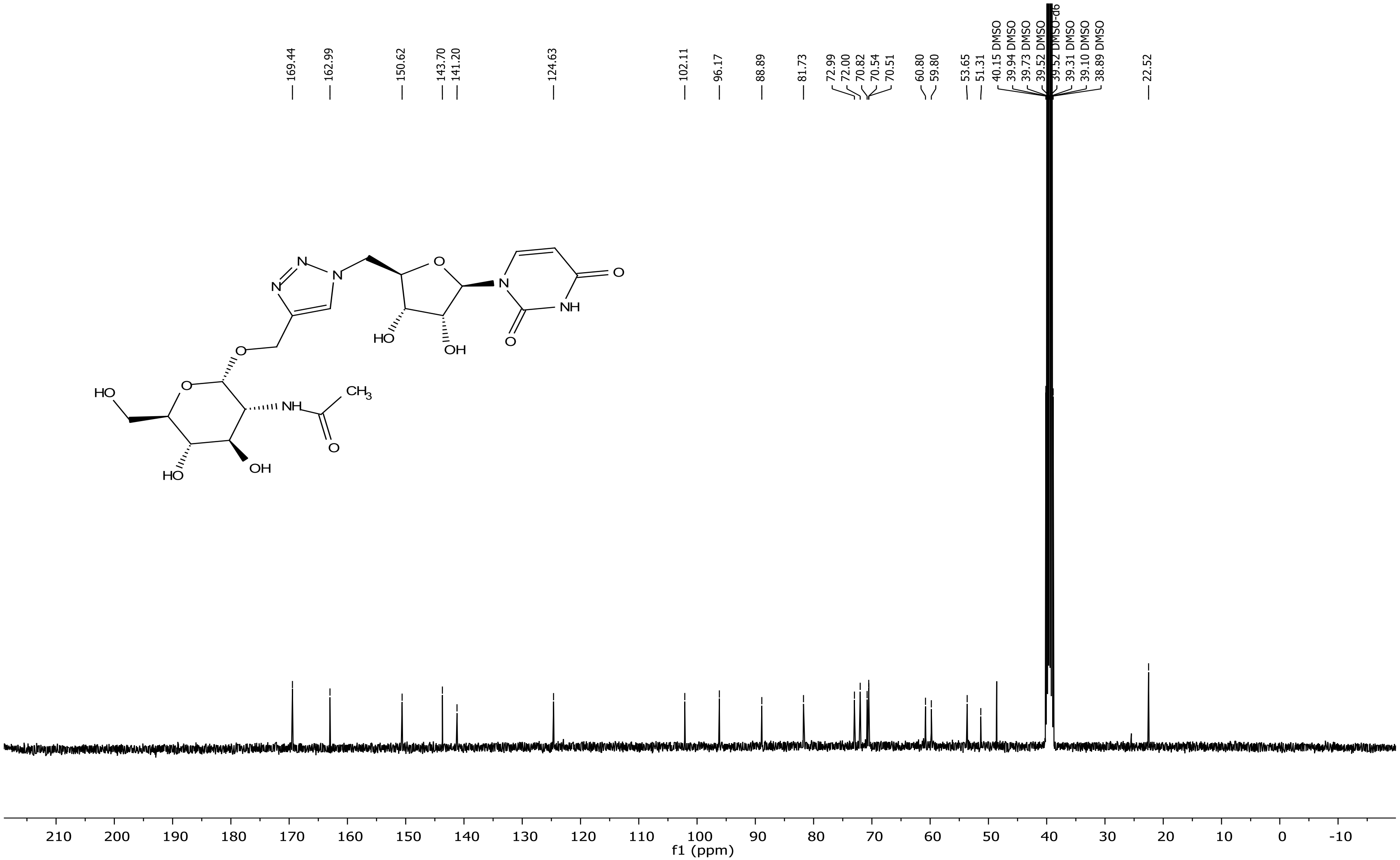

^{1}H-^{13}C HMBC NMR (DMSO-d6) of compound **7**

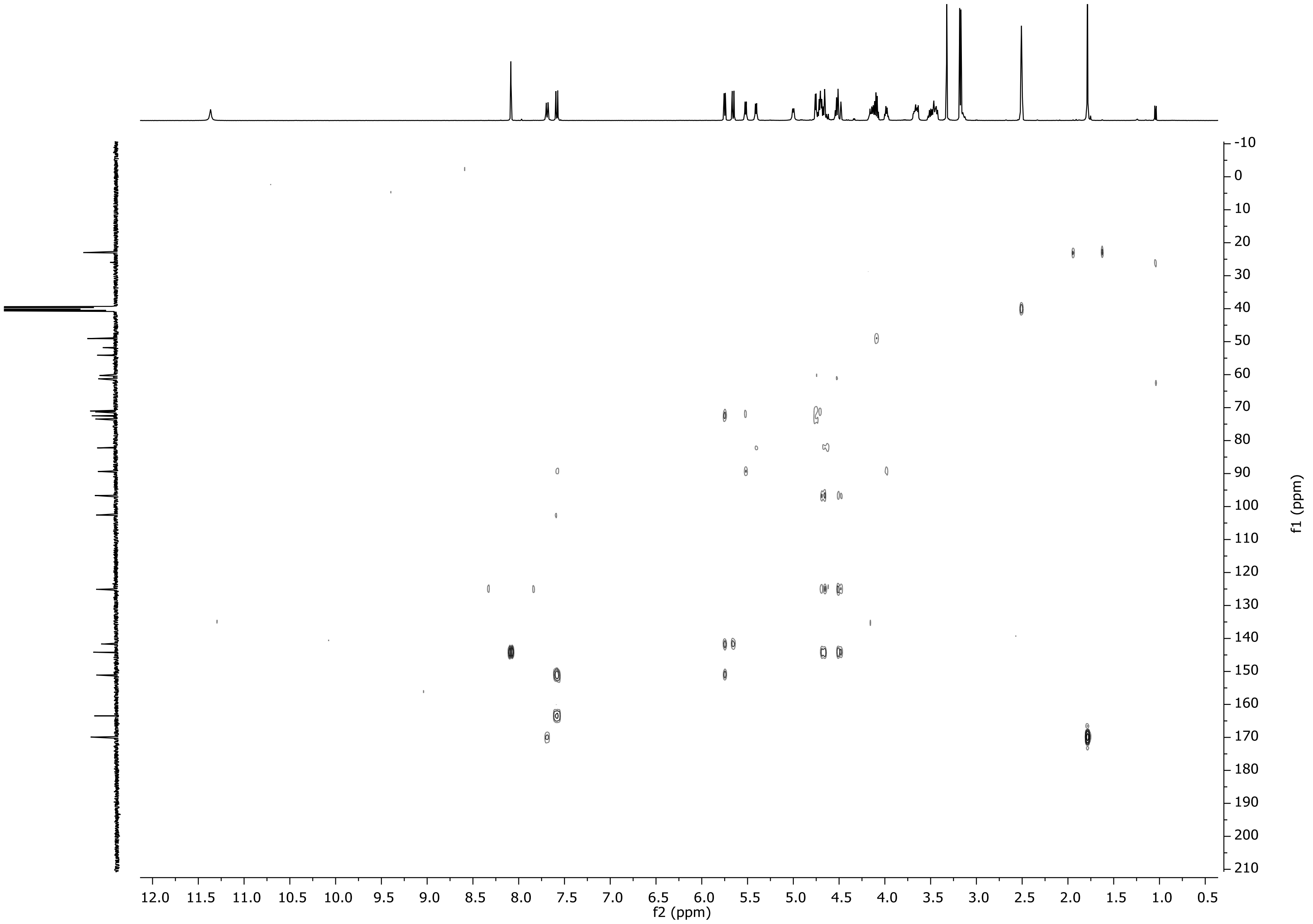

^{1}H NMR (DMSO-d6) of compound **8**

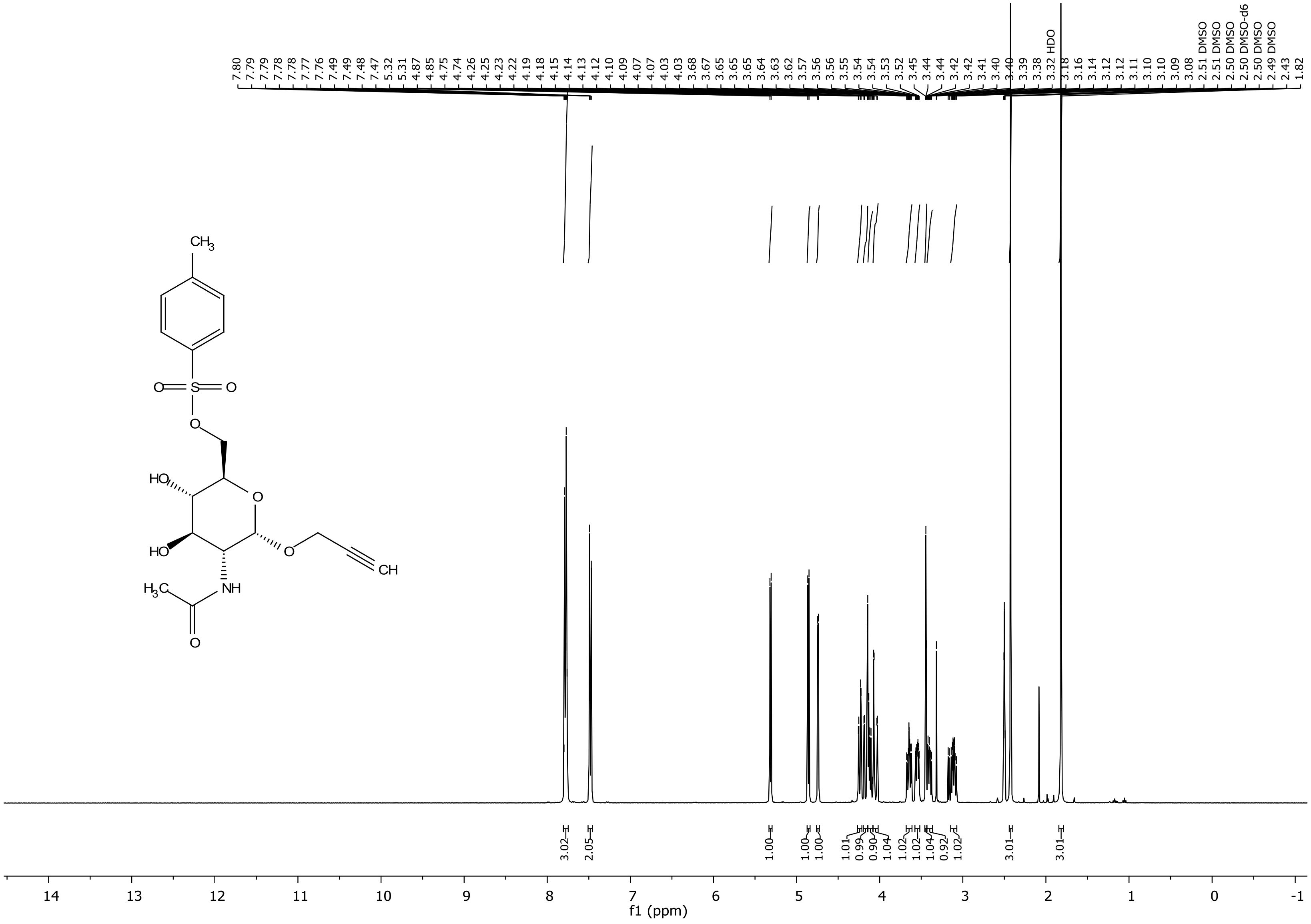

7.80 7.79 7.79 7.78 7.78 7.77 7.76 7.49 7.49 7.48 7.47 5.32 5.31 4.87 4.85 4.75 4.74 4.26 4.25 4.23 4.22 4.19 4.18 4.15 4.14 4.13 4.12 4.10 4.09 4.07 4.07 4.03 4.03 3.68 3.67 3.65 3.65 3.64 3.63 3.62 3.57 3.56 3.55 3.54 3.54 3.53 3.52 3.45 3.44 3.44 3.42 3.42 3.41 3.40 3.40 3.39 3.38 3.32 HDO 3.18 3.16 3.14 3.12 3.12 3.11 3.10 3.10 3.09 3.08 2.51 DMSO 2.51 DMSO 2.50 DMSO-d6 2.50 DMSO 2.49 DMSO 2.43 1.82
3.02 2.05 1.00 1.00 1.00 1.01 0.99 0.90 1.04 1.02 1.02 1.04 0.92 1.02 3.01 3.01
14 13 12 11 10 9 8 7 6 5 4 3 2 1 0 -1
f1 (ppm)
CH3
O S O
O
HO
O
HO
O
CH
H3C
NH
O

^{13}C NMR (DMSO-d6) of compound **8**

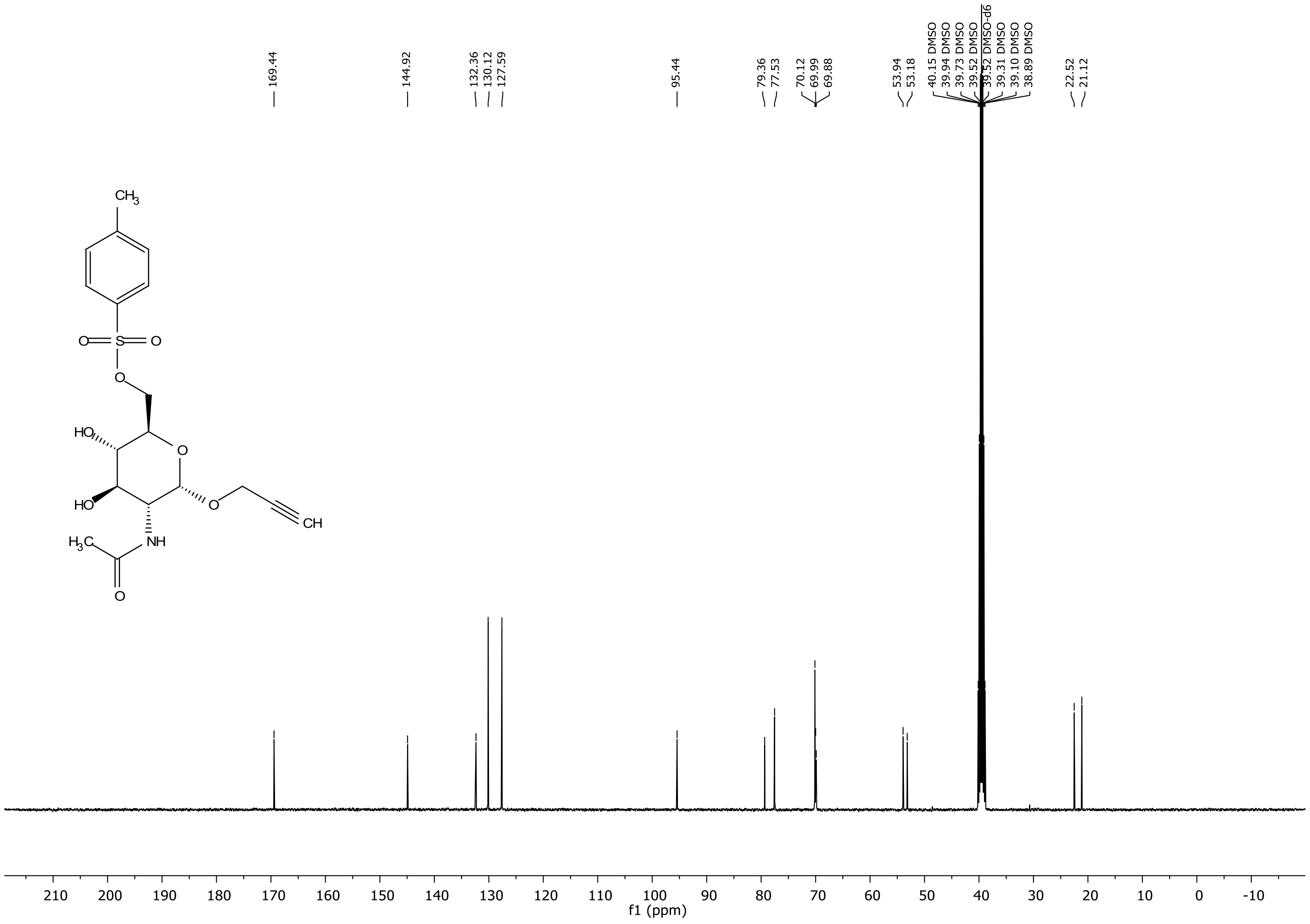

^{1}H NMR (DMSO-d6) of compound **9**

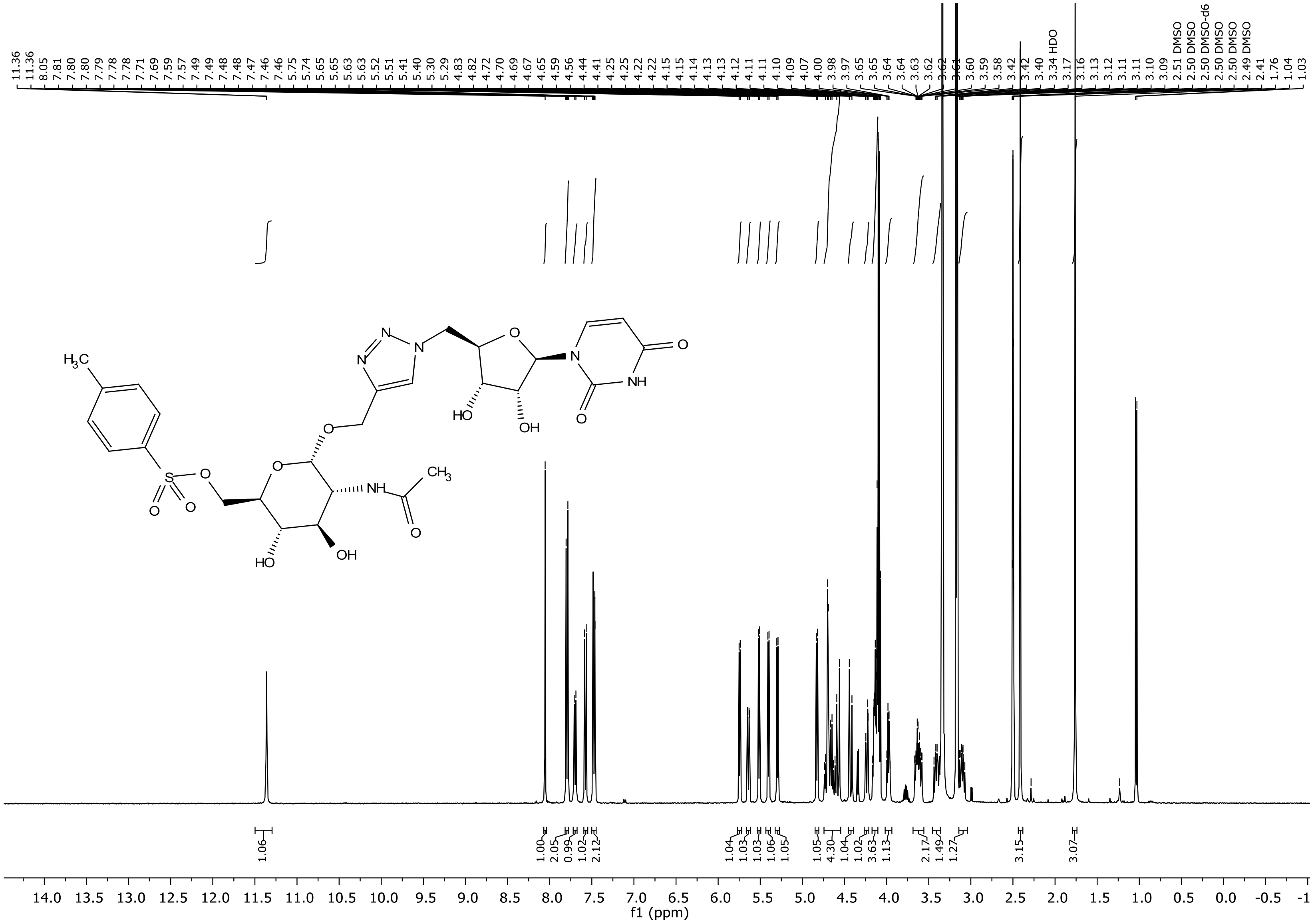
11.36
11.36
8.05
7.81
7.80
7.79
7.78
7.78
7.71
7.69
7.59
7.57
7.49
7.49
7.48
7.47
7.46
7.46
5.75
5.74
5.65
5.65
5.63
5.63
5.52
5.51
5.41
5.40
5.30
5.29
4.83
4.82
4.72
4.70
4.69
4.67
4.65
4.59
4.56
4.44
4.41
4.25
4.25
4.22
4.22
4.15
4.15
4.14
4.13
4.13
4.12
4.11
4.11
4.10
4.09
4.07
4.00
3.98
3.97
3.65
3.65
3.64
3.63
3.62
3.62
3.61
3.60
3.59
3.58
3.42
3.42
3.40
3.34 HDO
3.17
3.16
3.13
3.12
3.11
3.11
3.10
3.09
2.51 DMSO
2.50 DMSO
2.50 DMSO-d6
2.50 DMSO
2.50 DMSO
2.49 DMSO
2.41
1.76
1.04
1.03
H3C
S
O
O
O
O
O
NH
CH3
O
HO
OH
N
N
N
O
HO
OH
N
O
NH
O
1.06
1.00
2.05
0.99
1.02
2.12
1.04
1.03
1.03
1.06
1.05
1.05
4.30
1.04
1.02
3.63
1.13
2.17
1.49
1.27
3.15
3.07
14.0 13.5 13.0 12.5 12.0 11.5 11.0 10.5 10.0 9.5 9.0 8.5 8.0 7.5 7.0 6.5 6.0 5.5 5.0 4.5 4.0 3.5 3.0 2.5 2.0 1.5 1.0 0.5 0.0 -0.5 -1
f1 (ppm)

^{13}C NMR (DMSO-d6) of compound **9**

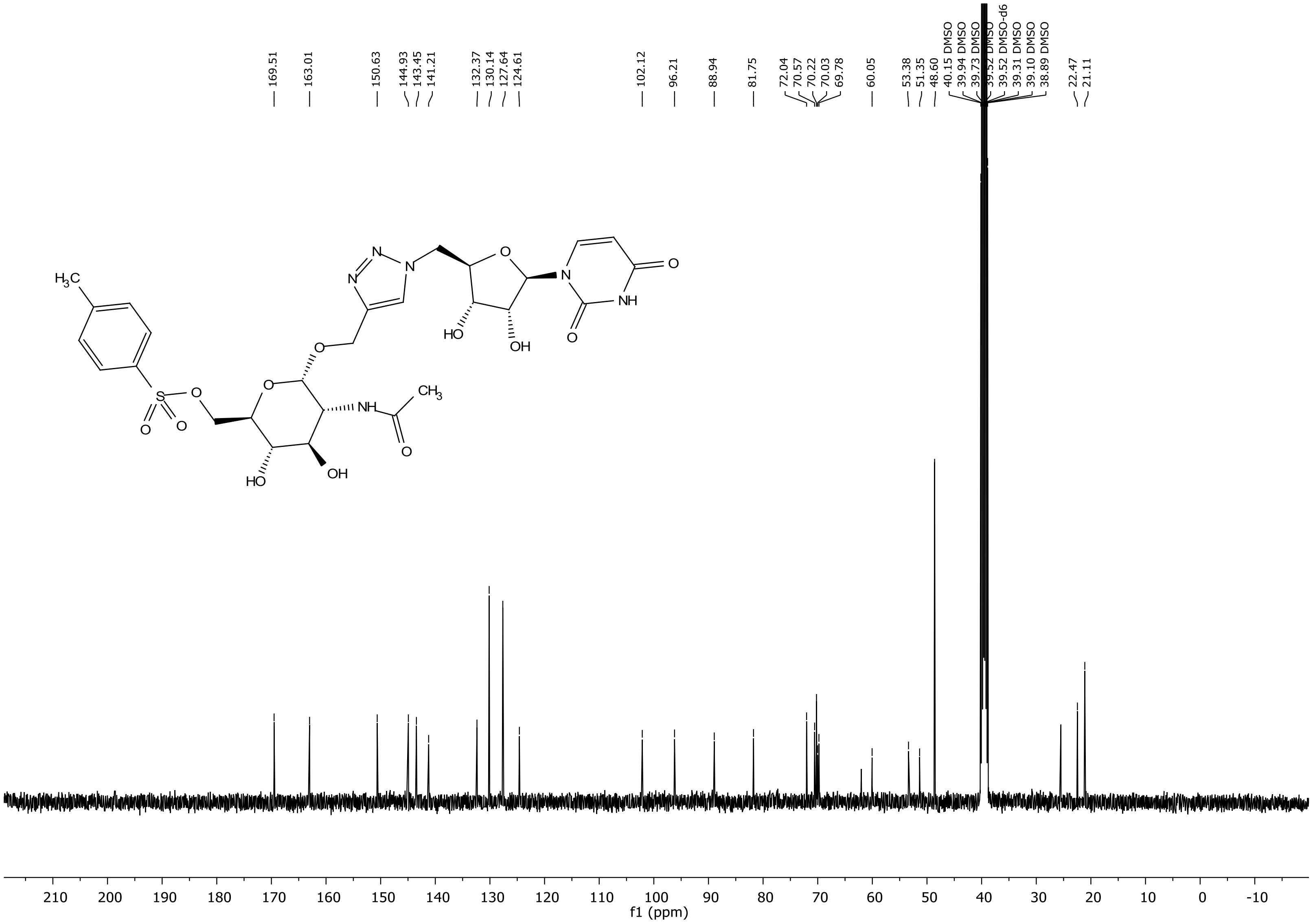

^{1}H-^{13}C HMBC NMR (DMSO-d6) of compound **9**

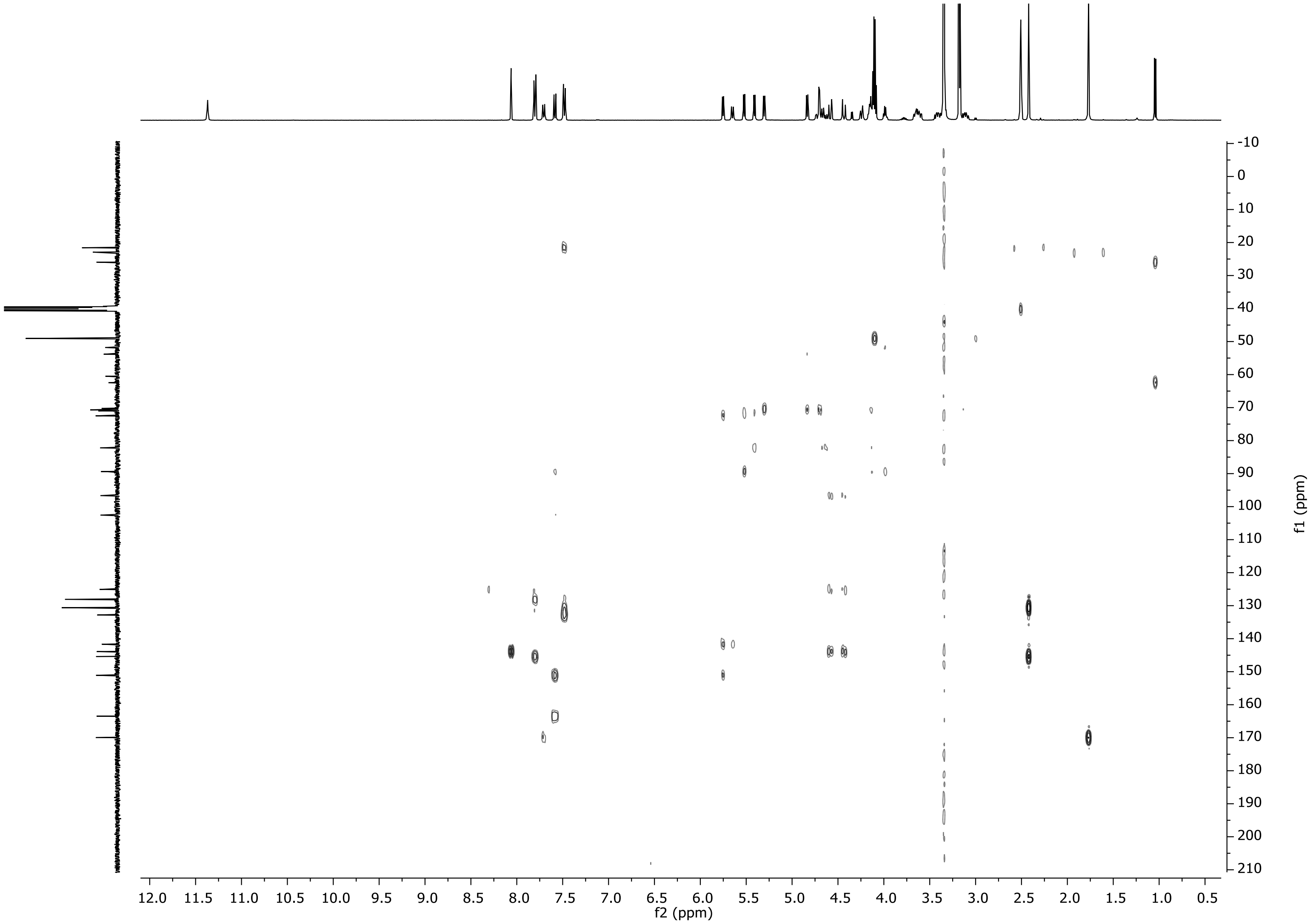

^{1}H NMR (DMSO-d6) of compound **10**

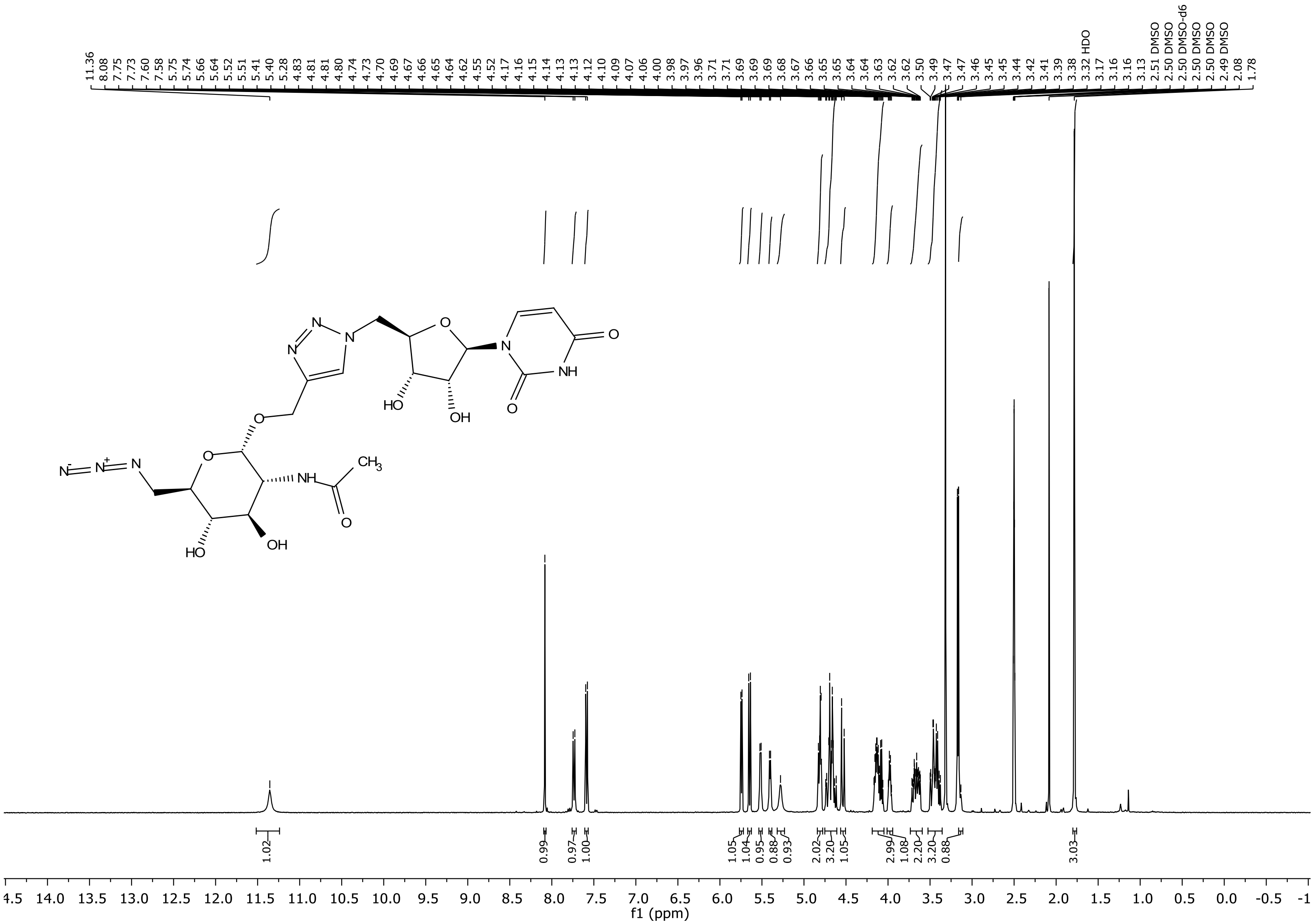

¹³C HMBC NMR (DMSO-d6) of compound **10**

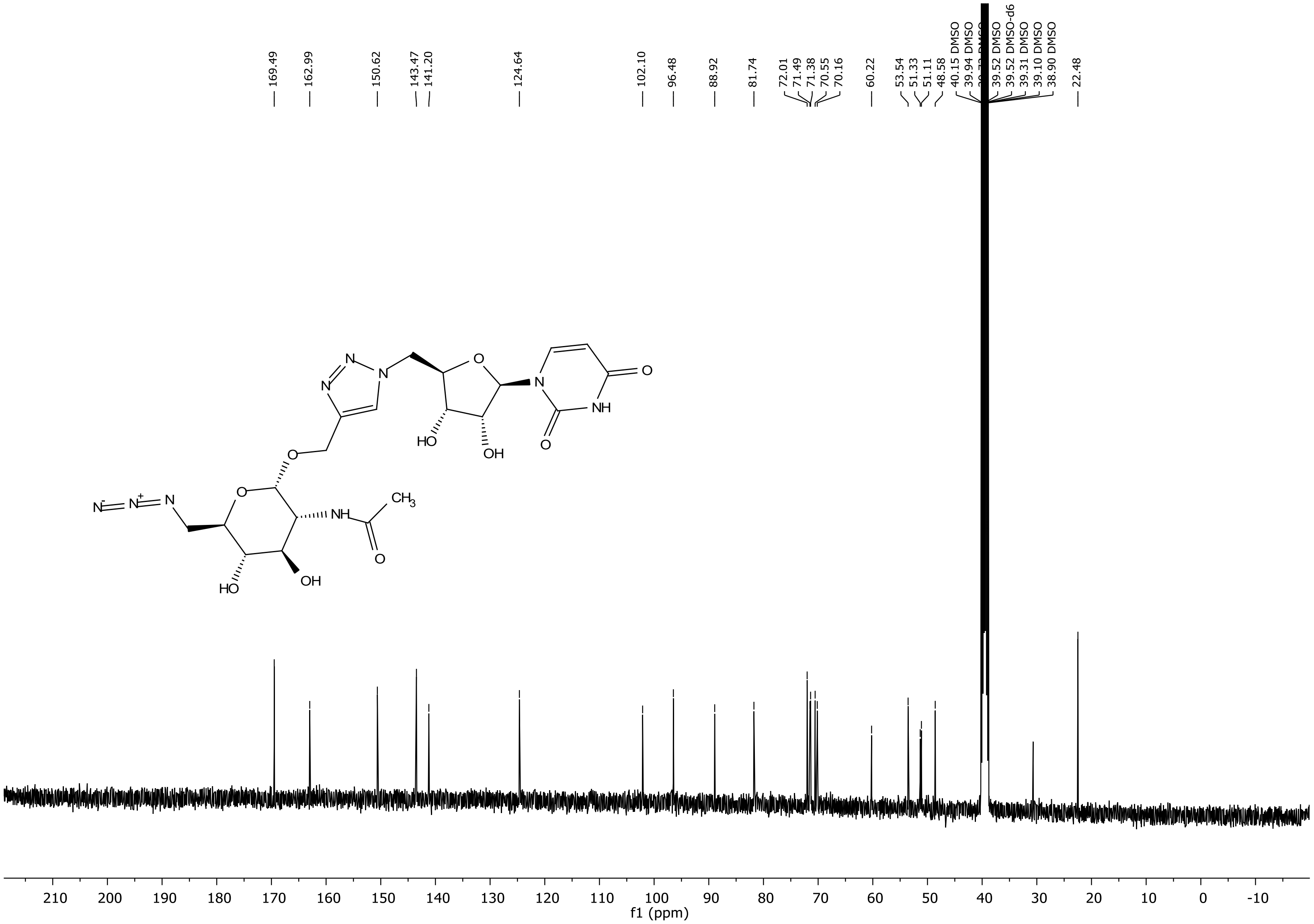

^1H-^{13}C HMBC NMR (DMSO-d6) of compound **10**

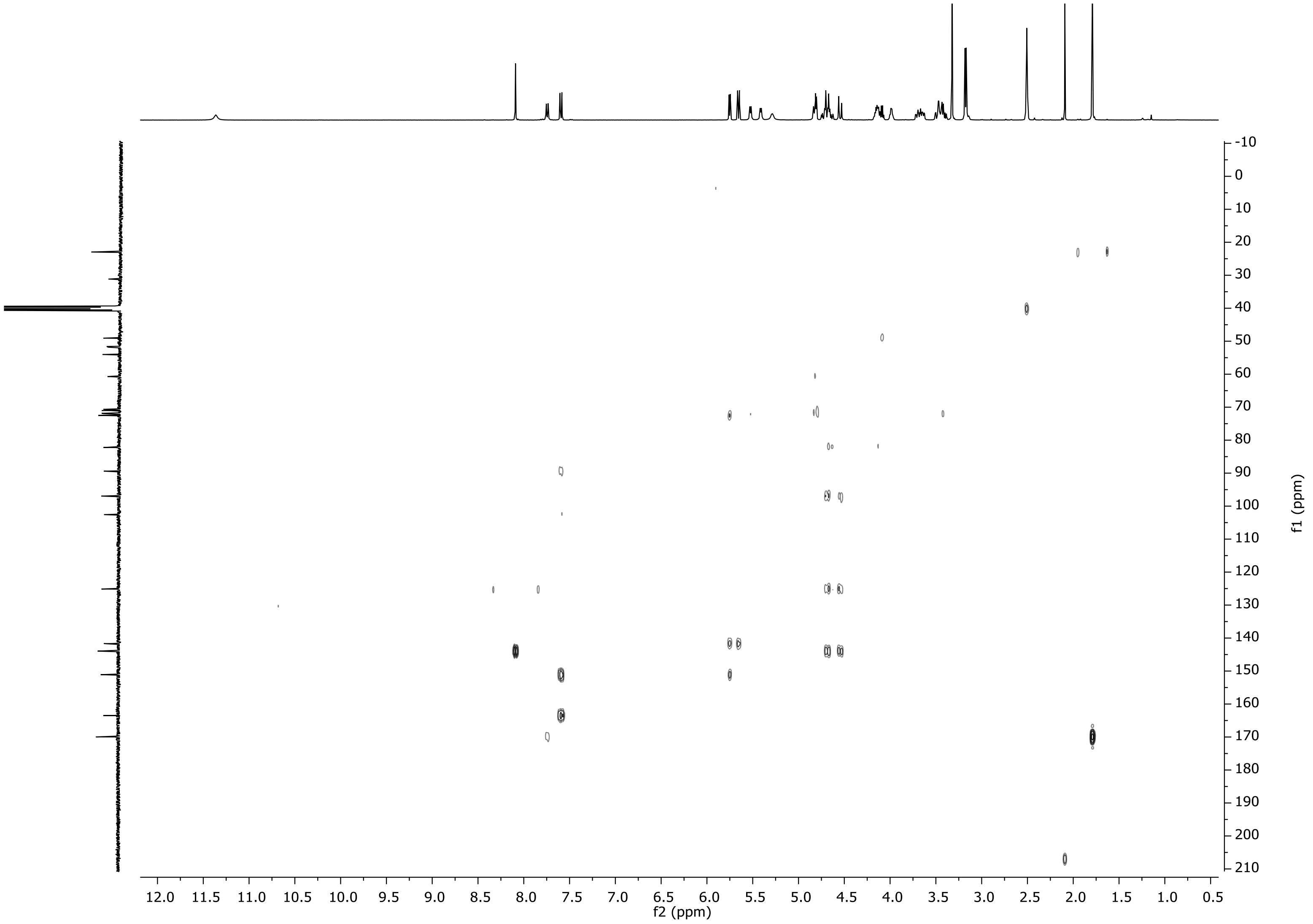

^{1}H NMR (DMSO-d6) of compound **11a**

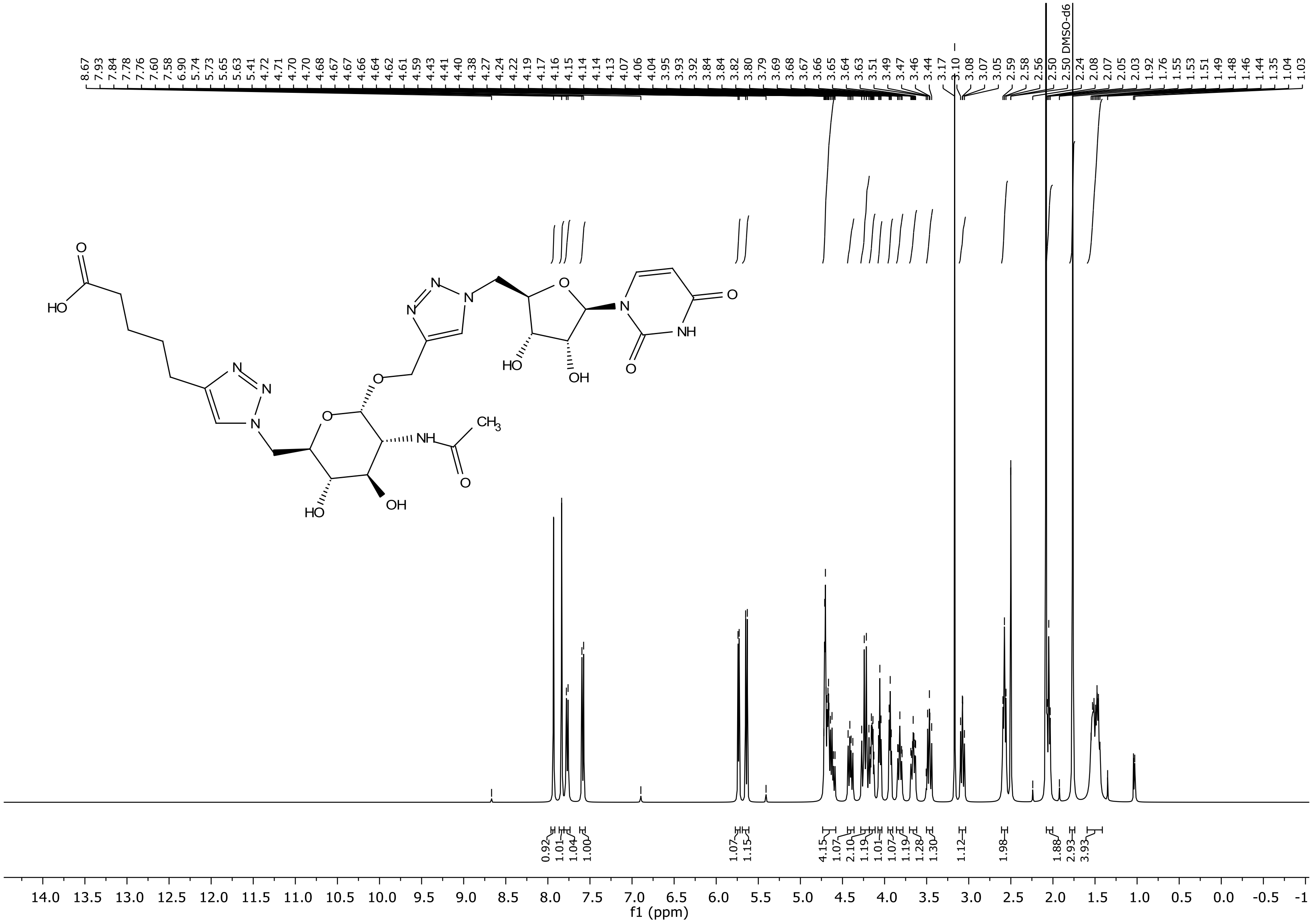

8.67
7.93
7.84
7.78
7.76
7.60
7.58
6.90
5.74
5.73
5.65
5.63
5.41
4.72
4.71
4.70
4.70
4.68
4.67
4.67
4.66
4.64
4.62
4.61
4.59
4.43
4.41
4.40
4.38
4.27
4.24
4.22
4.19
4.17
4.16
4.15
4.14
4.14
4.13
4.07
4.06
4.04
3.95
3.93
3.92
3.84
3.84
3.82
3.80
3.79
3.69
3.68
3.67
3.66
3.65
3.64
3.63
3.51
3.49
3.47
3.46
3.44
3.17
3.10
3.08
3.07
3.05
2.59
2.58
2.56
2.50
2.50 DMSO-d6
2.24
2.08
2.07
2.05
2.03
1.92
1.76
1.55
1.53
1.51
1.49
1.48
1.46
1.44
1.35
1.04
1.03
HO
O
N
N
N
N
N
N
O
O
O
HO
OH
NH
O
CH3
NH
O
HO
OH
0.92
1.01
1.04
1.00
1.07
1.15
4.15
1.07
2.10
1.19
1.01
1.07
1.19
1.28
1.30
1.12
1.98
1.88
2.93
3.93
14.0 13.5 13.0 12.5 12.0 11.5 11.0 10.5 10.0 9.5 9.0 8.5 8.0 7.5 7.0 6.5 6.0 5.5 5.0 4.5 4.0 3.5 3.0 2.5 2.0 1.5 1.0 0.5 0.0 -0.5 -1
f1 (ppm)

^{13}C NMR (DMSO-d6) of compound **11a**

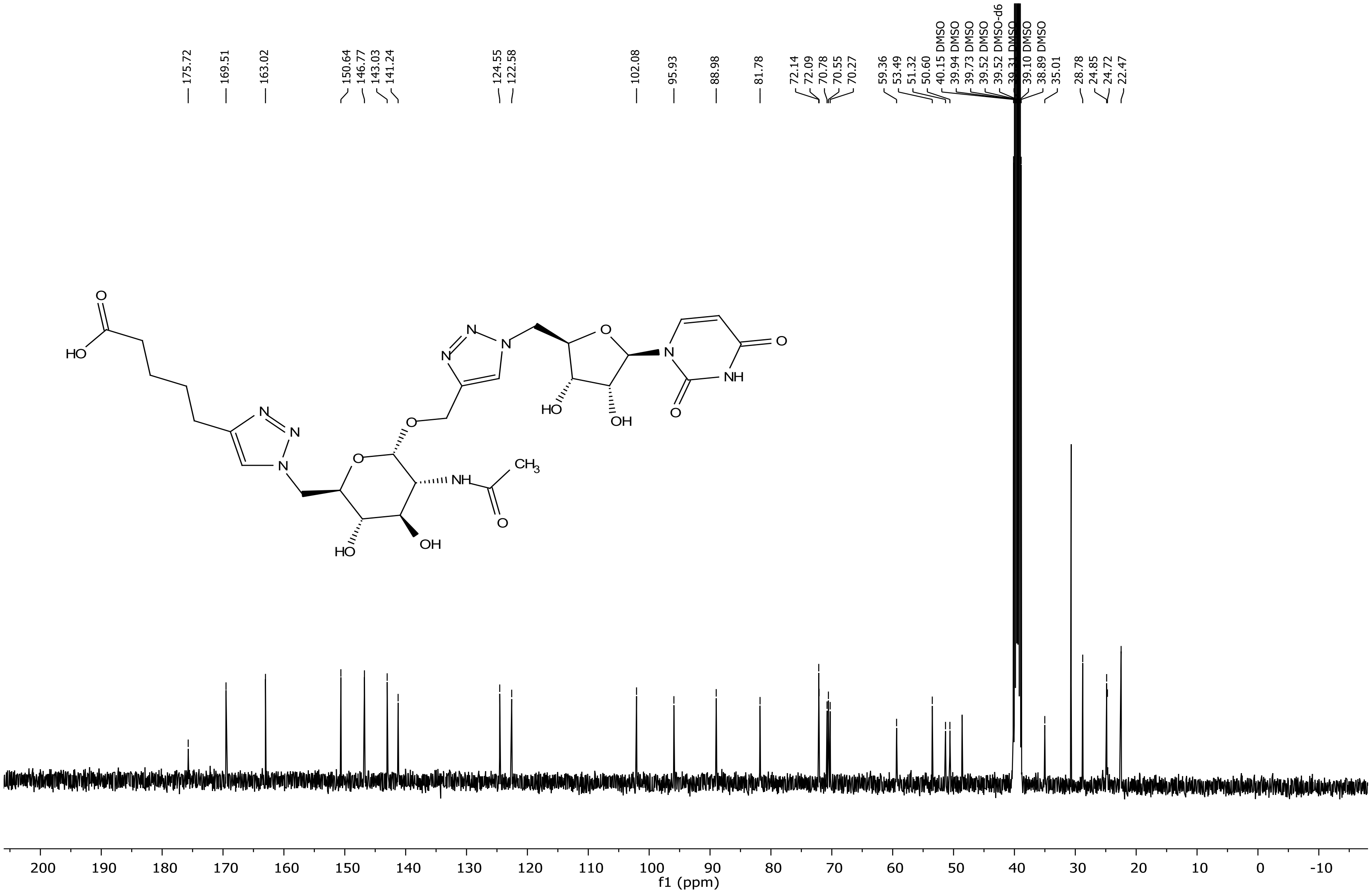

^{1}H-^{13}C HMBC NMR (DMSO-d6) of compound **11a**

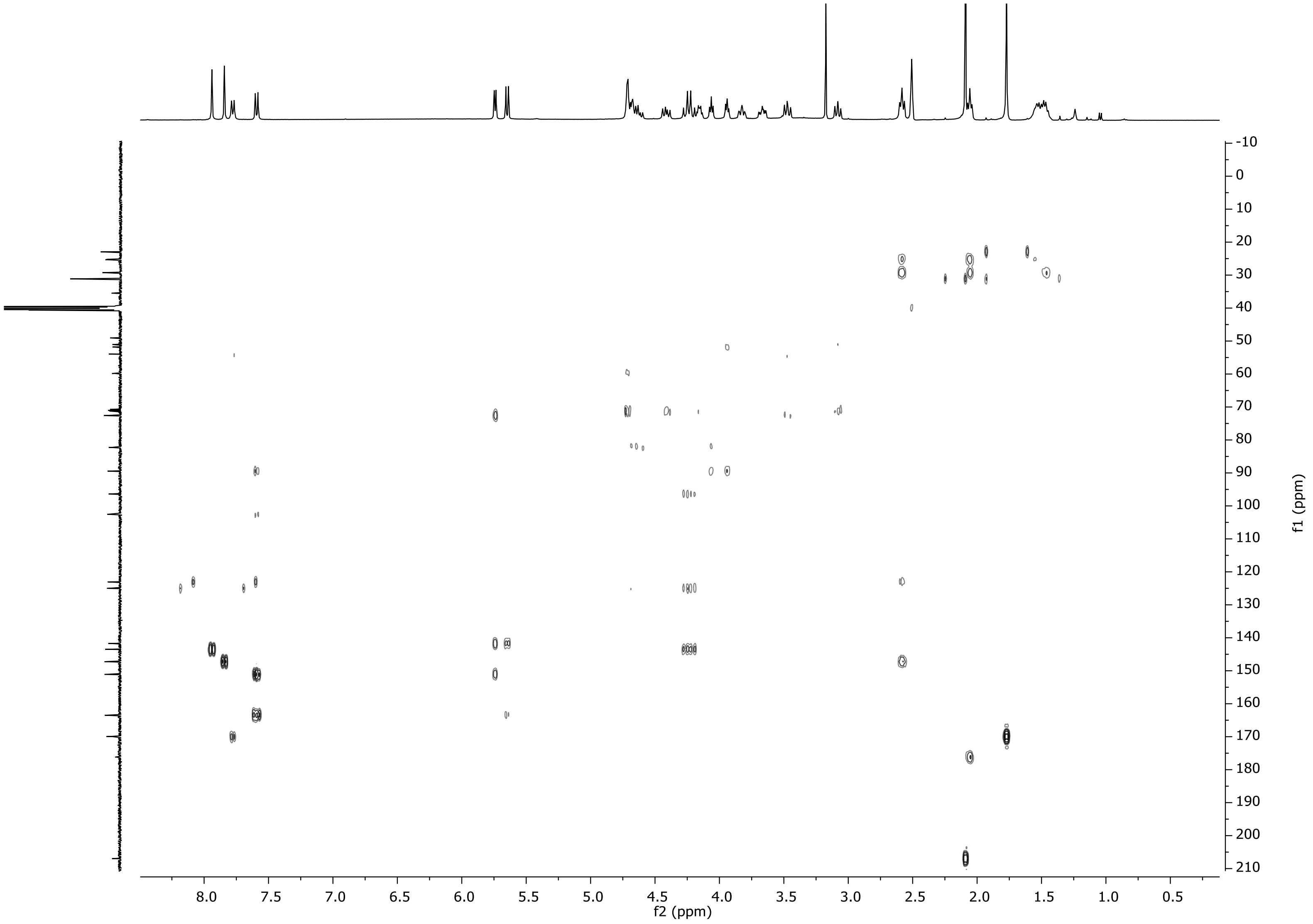

^{1}H NMR (DMSO-d6) of compound **11b**

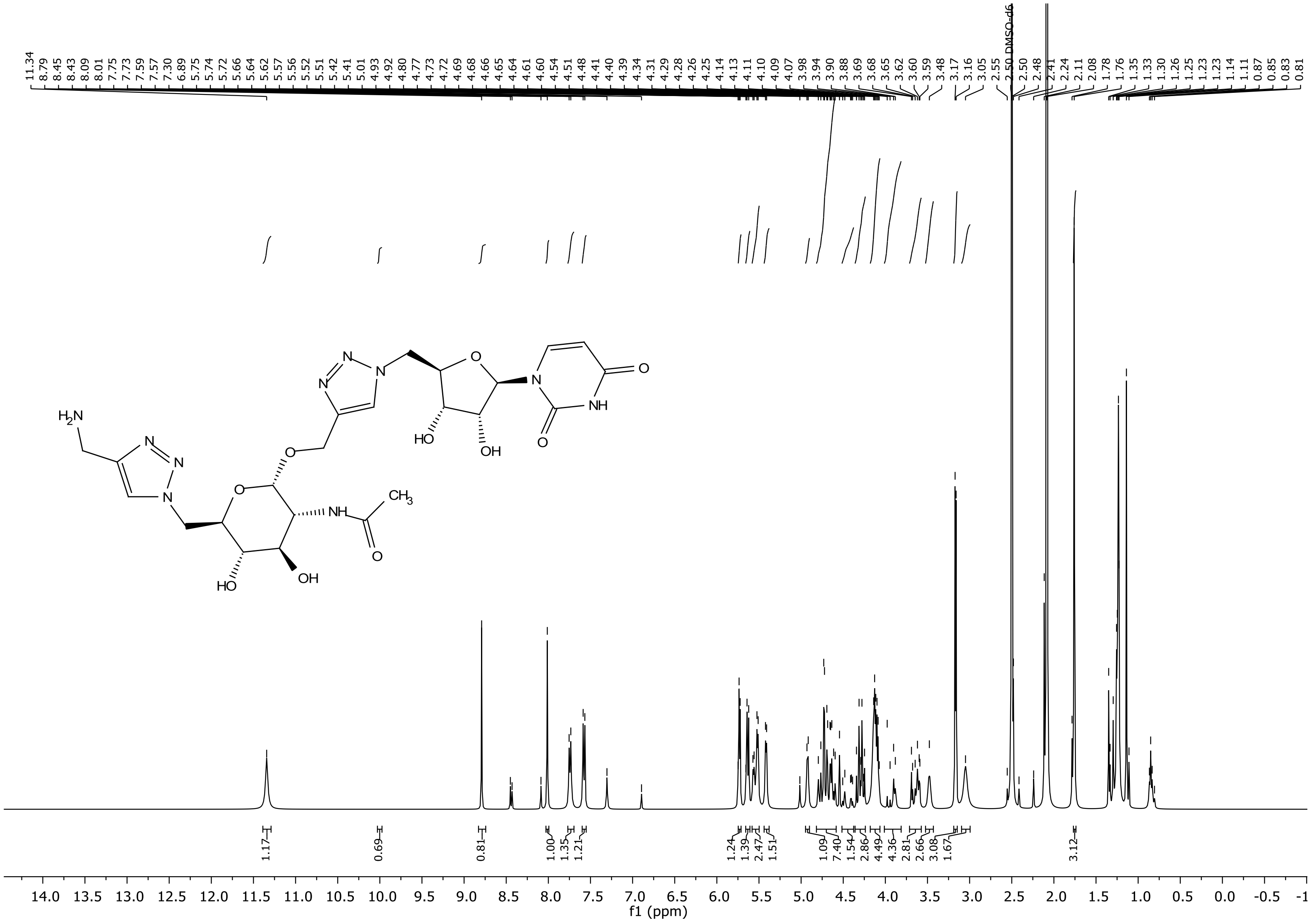

^{13}C NMR (DMSO-d6) of compound **11b**

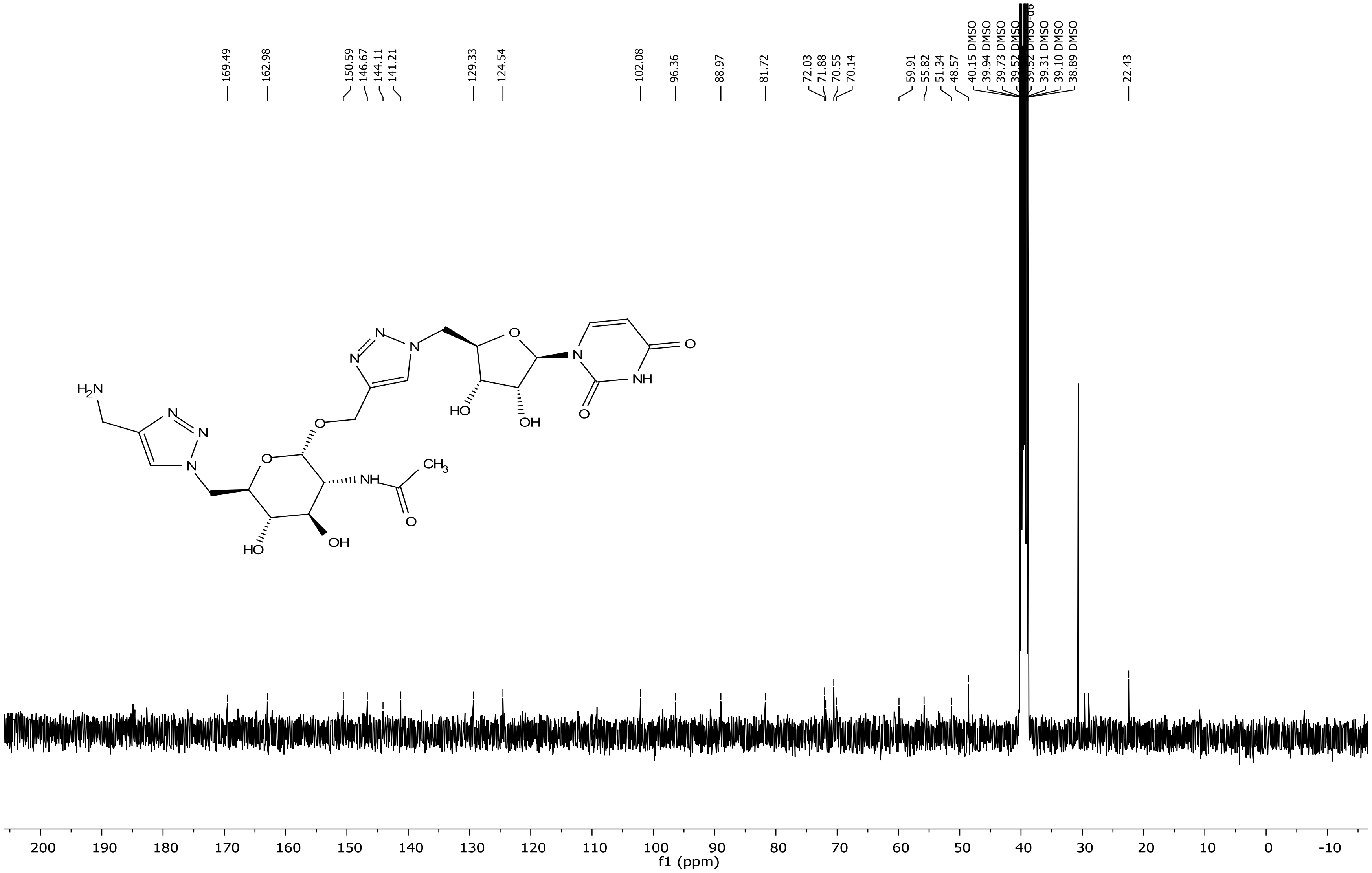

^{1}H-^{13}C HMBC NMR (DMSO-d6) of compound **11b**

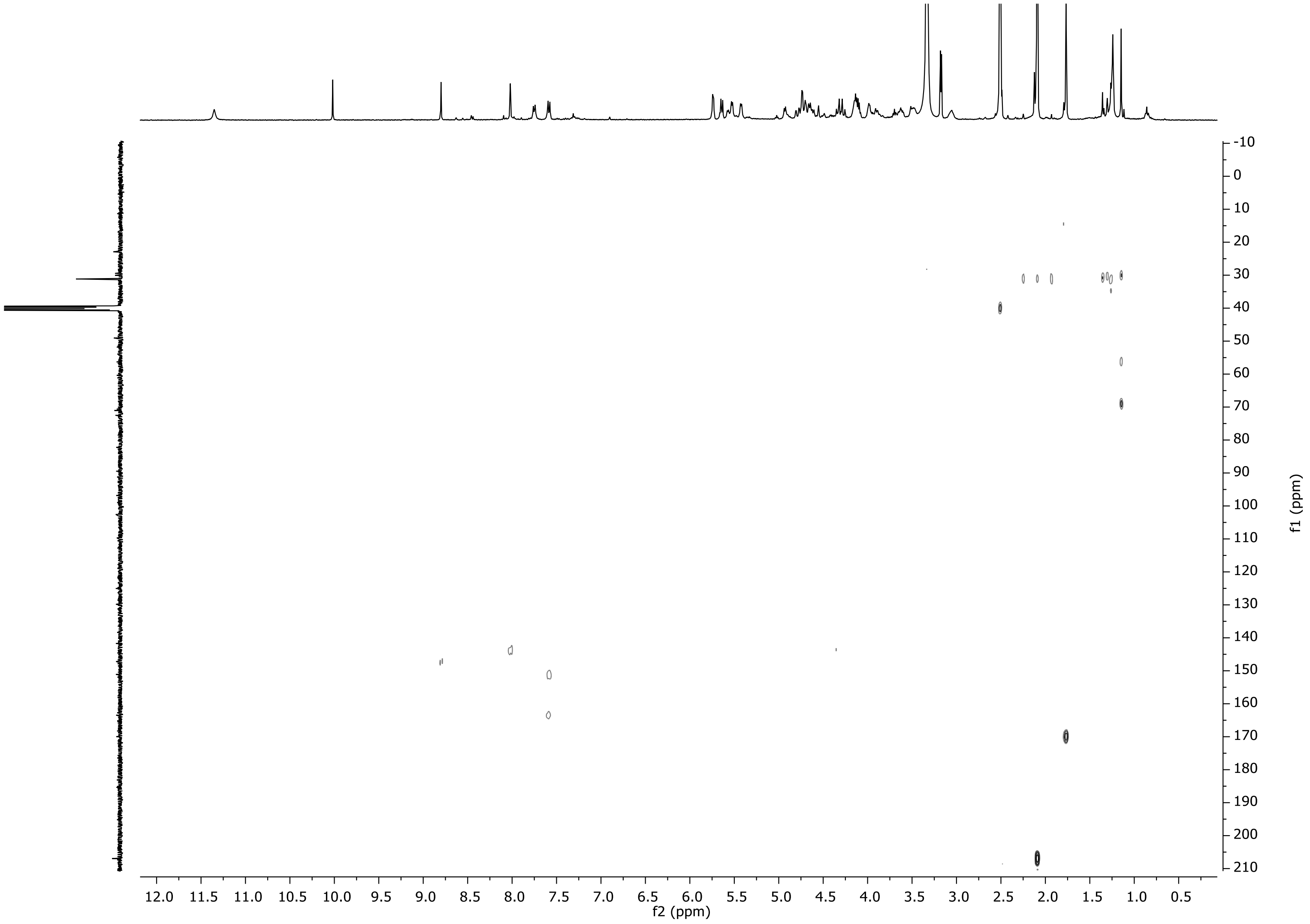

^{1}H NMR (DMSO-d6) of compound **11c**

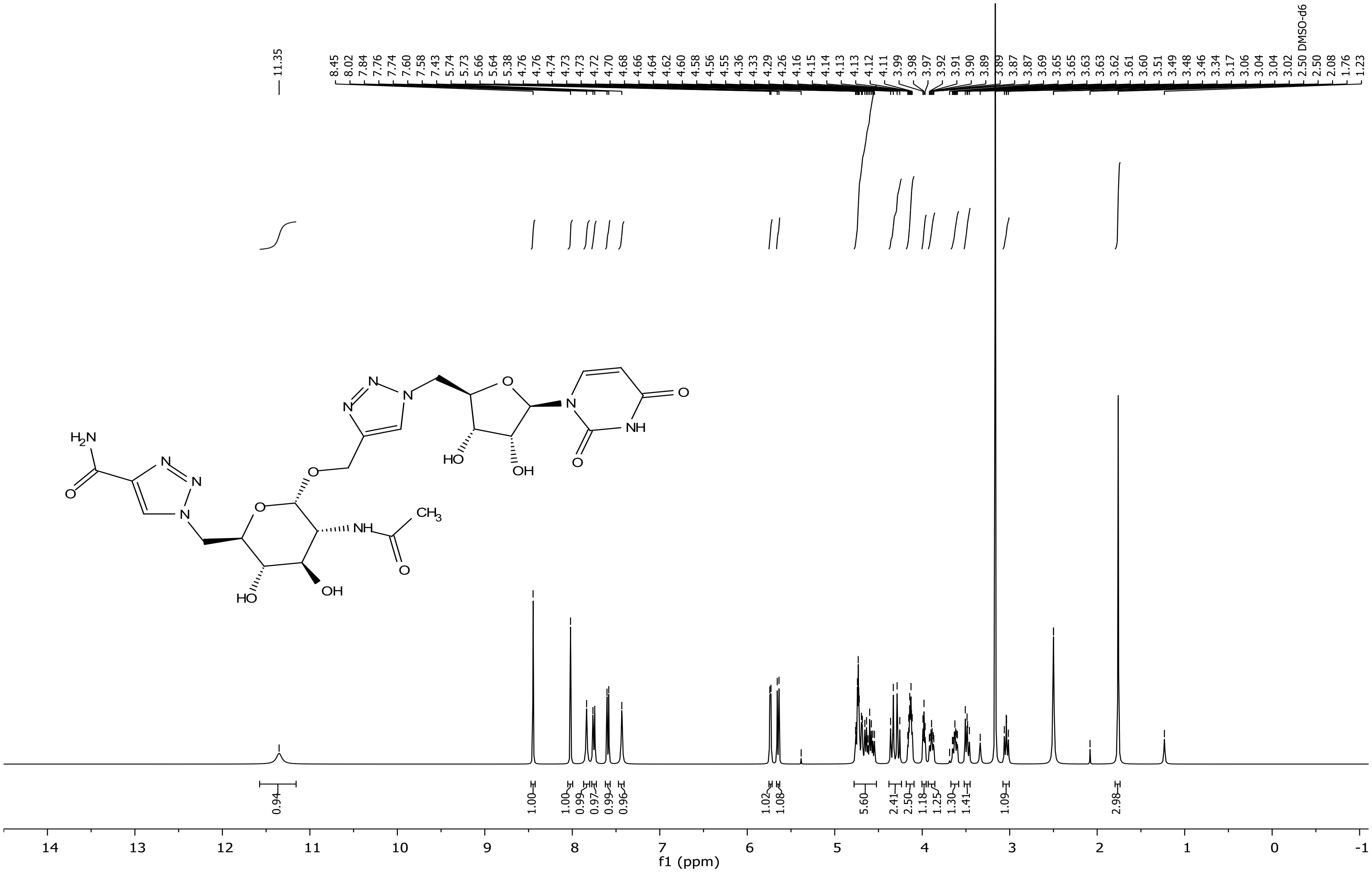

^{13}C NMR (DMSO-d6) of compound **11c**

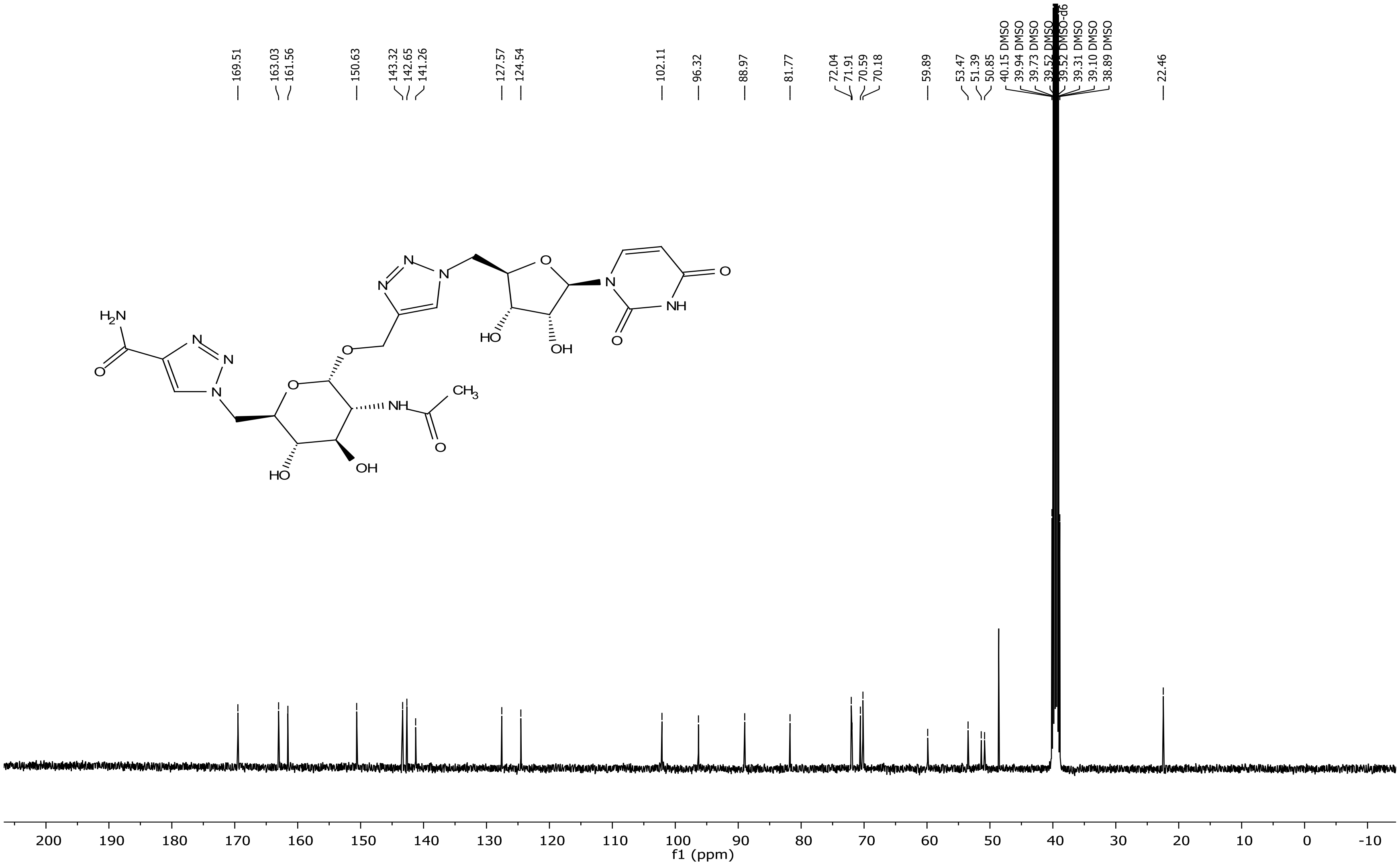

¹H-¹³C HMBC NMR (DMSO-d6) of compound **11c**

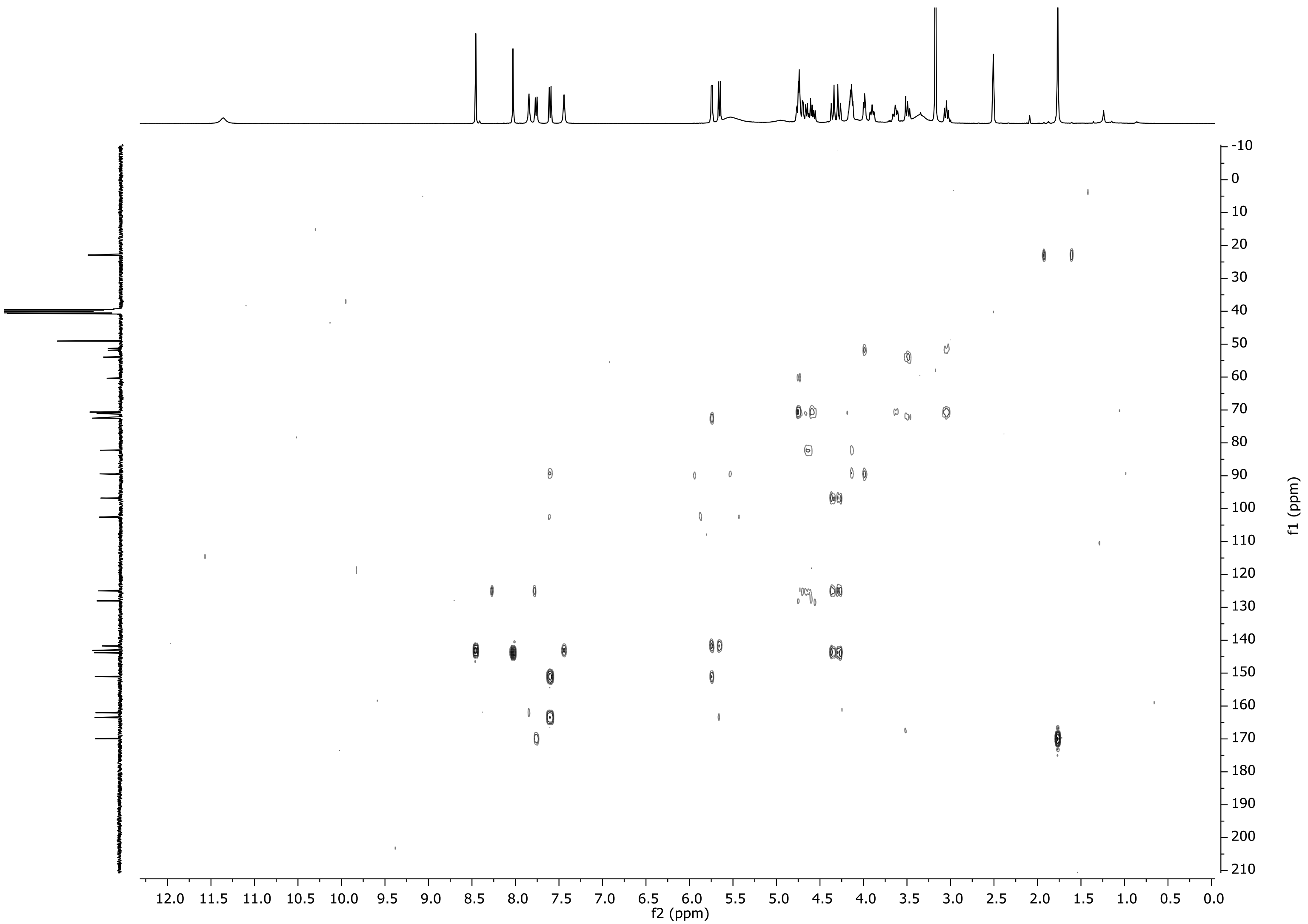

^{1}H NMR (DMSO-d6) of compound **11d**

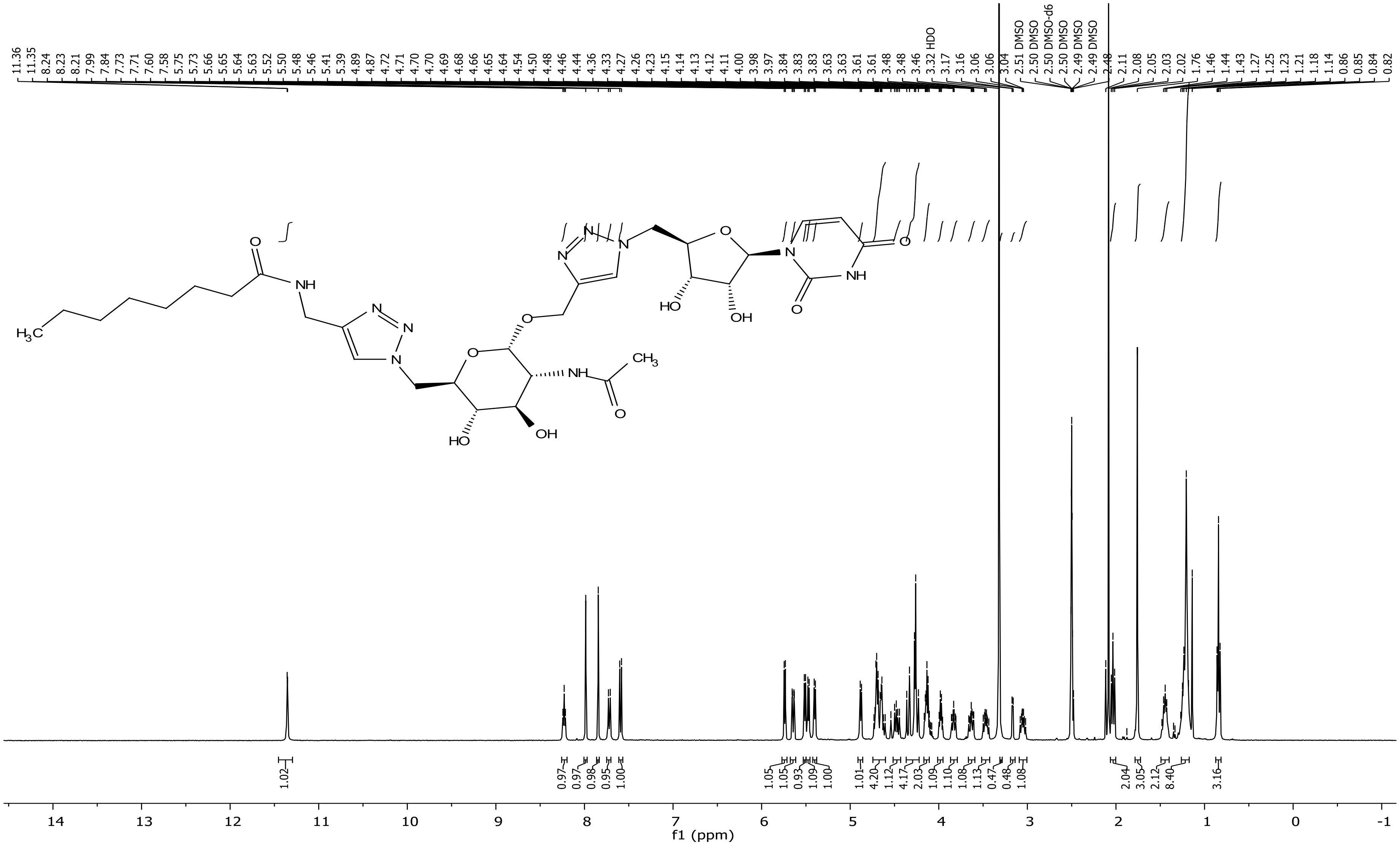

^{13}C NMR (DMSO-d6) of compound **11d**

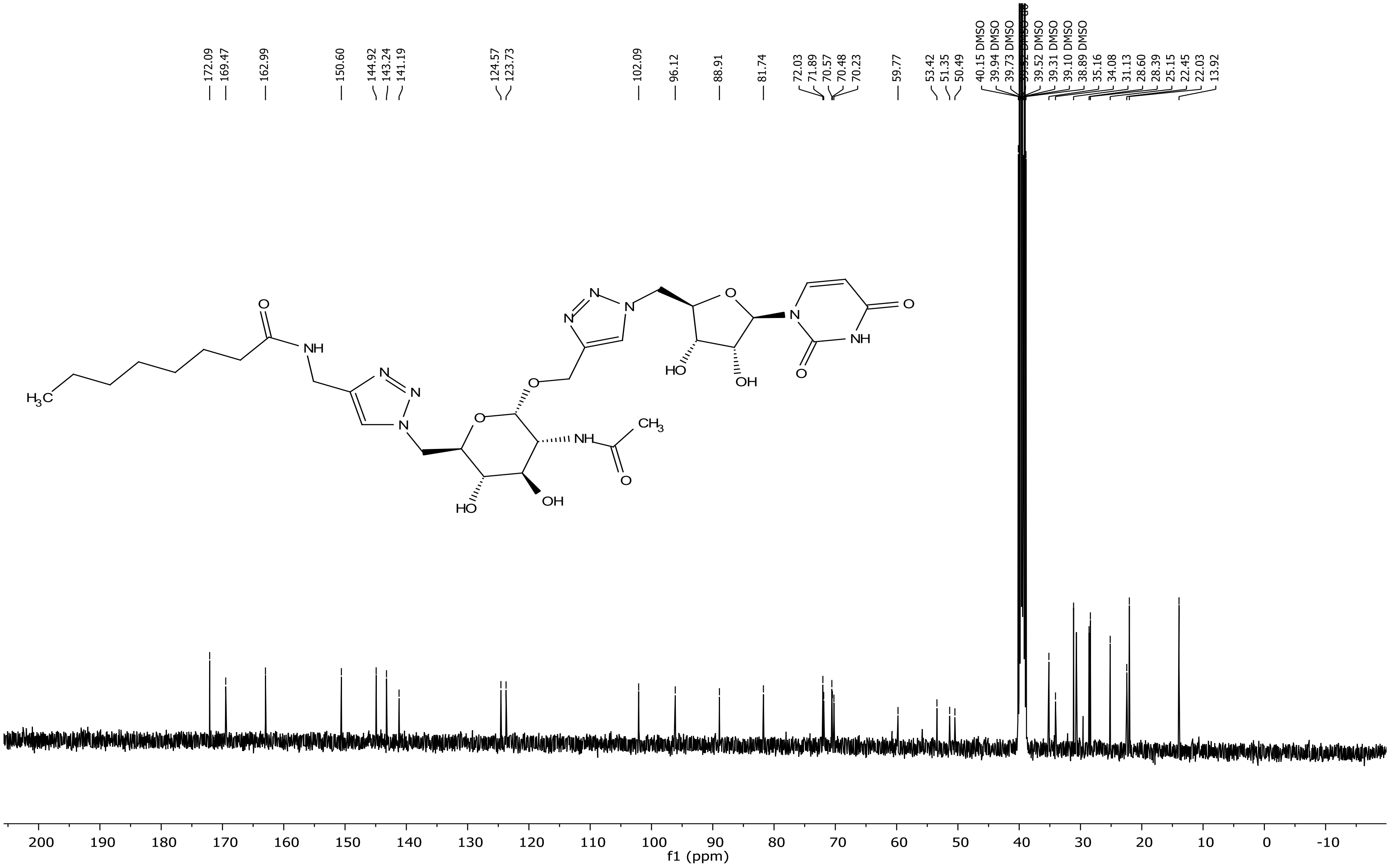

^{1}H-^{13}C HMBC NMR (DMSO-d6) of compound **11d**

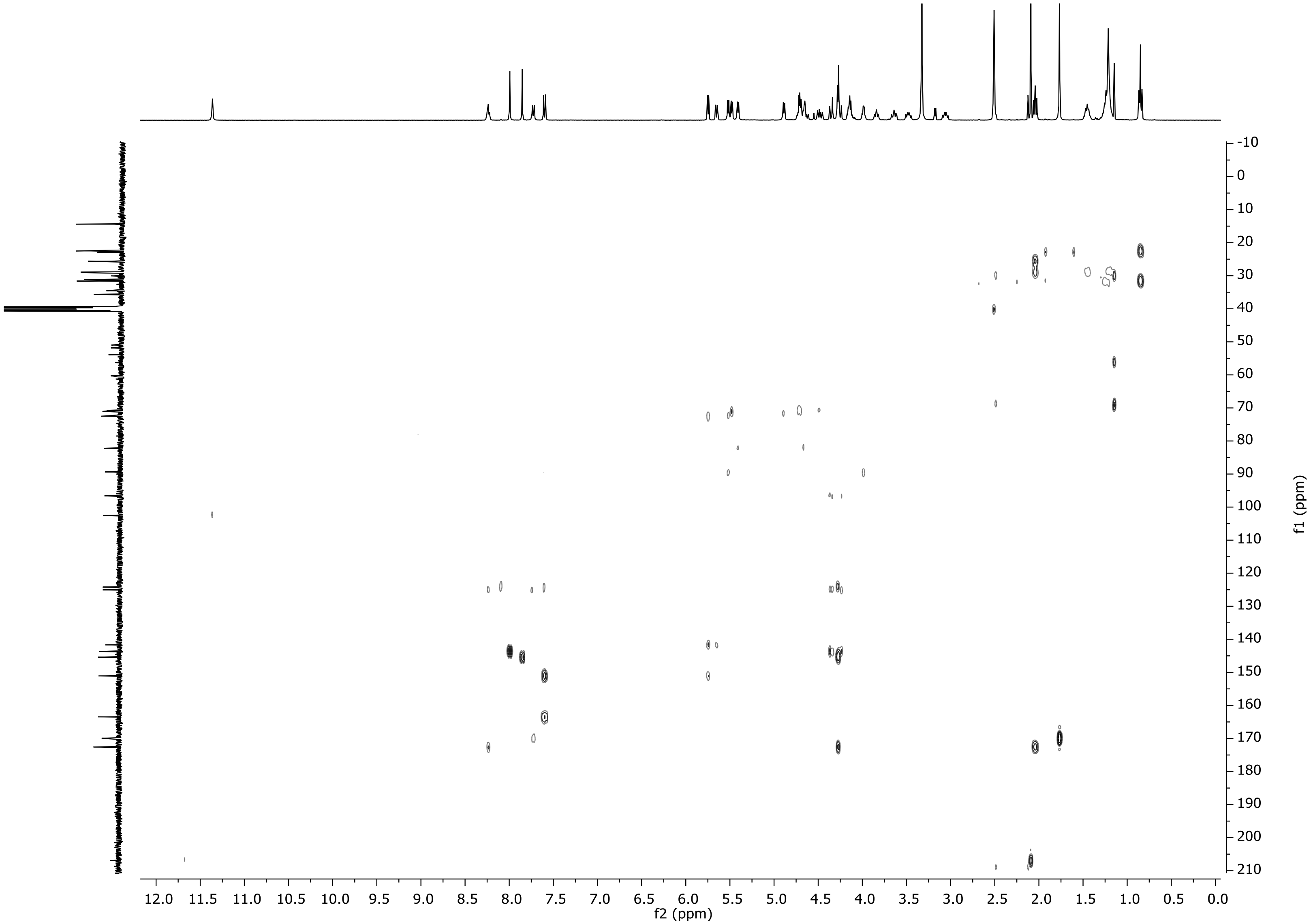

^{1}H NMR (DMSO-d6) of compound **11e**

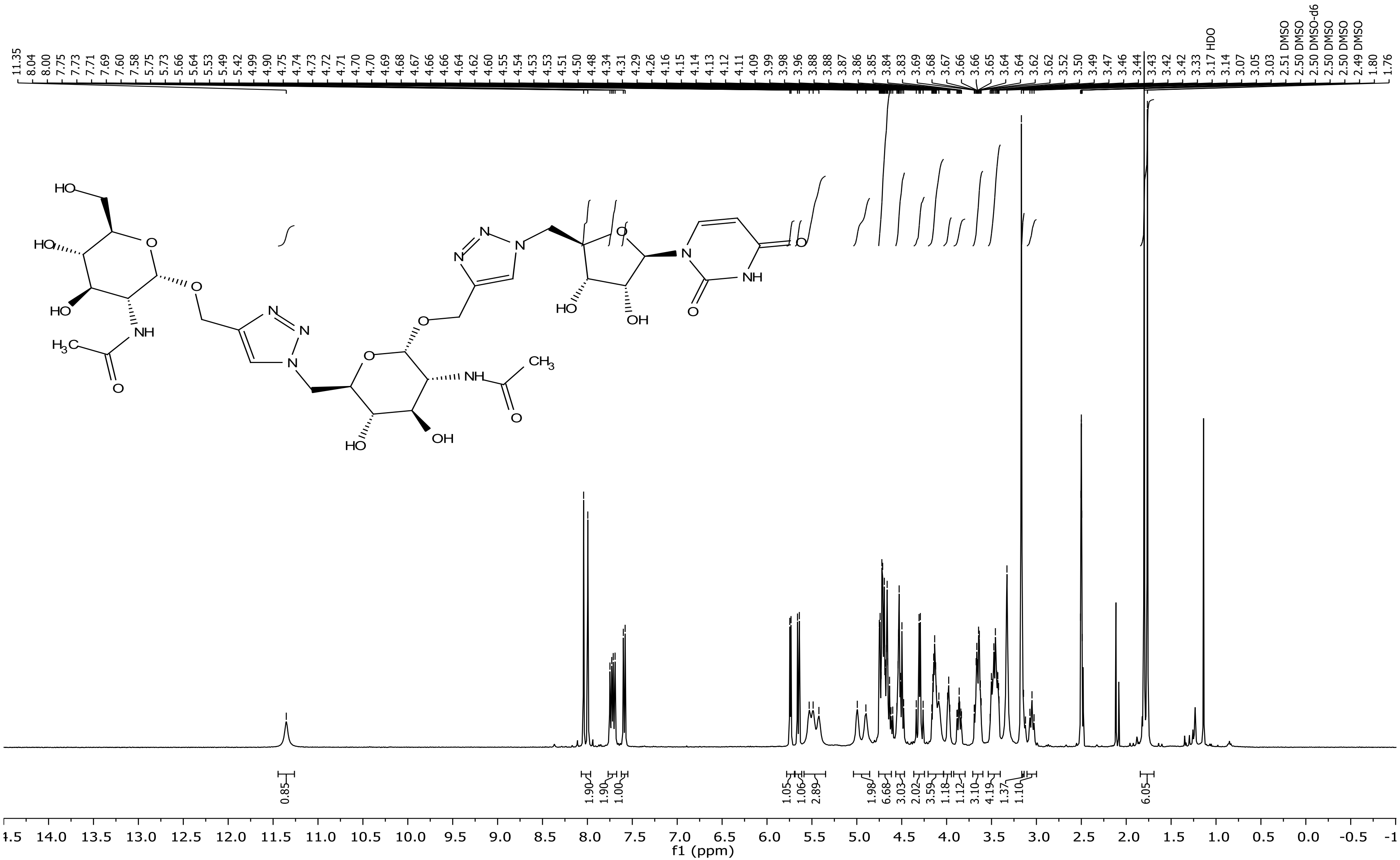

^{13}C NMR (DMSO-d6) of compound 11**e**

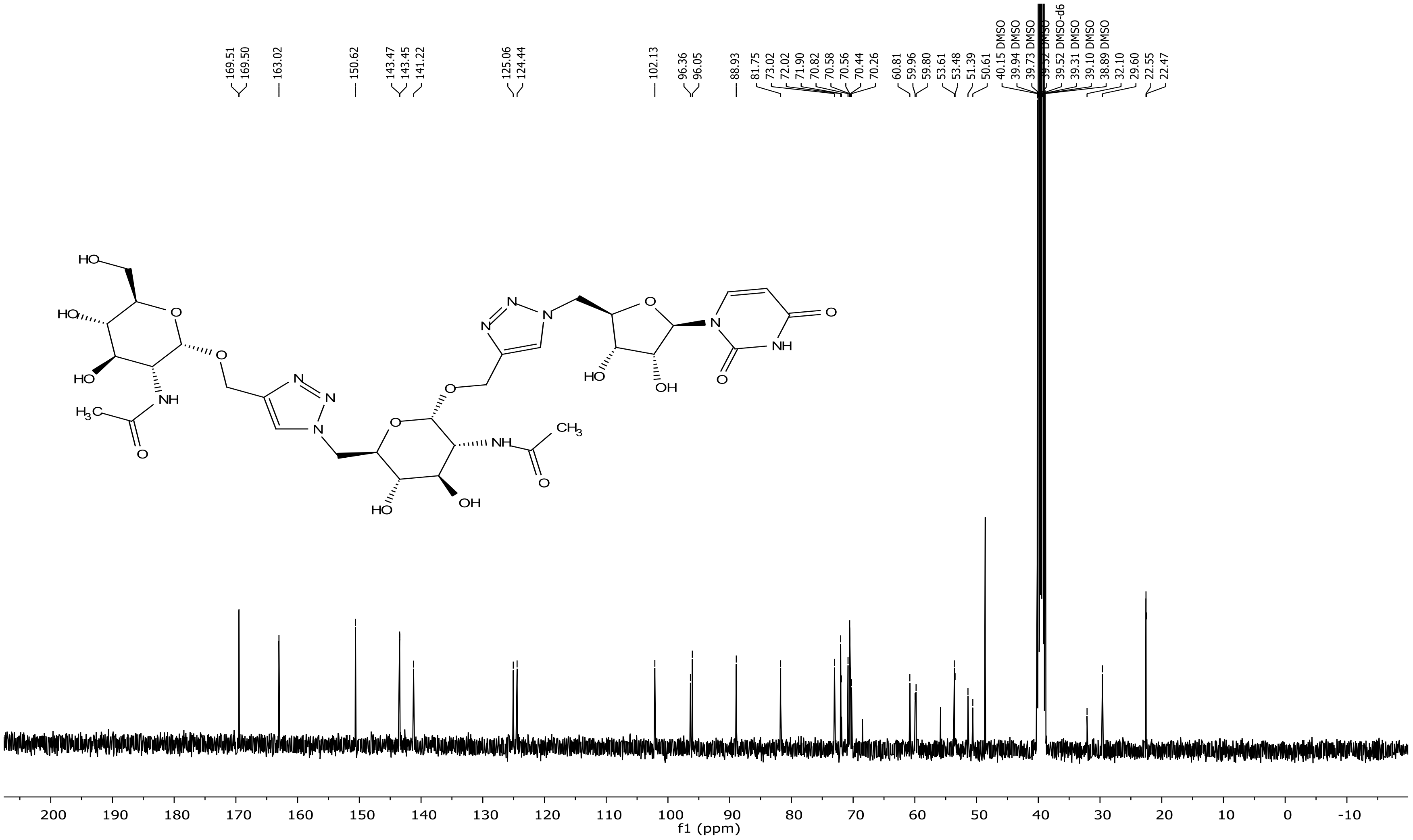

H-^{13}C HMBC NMR (DMSO-d6) of compound **11e**

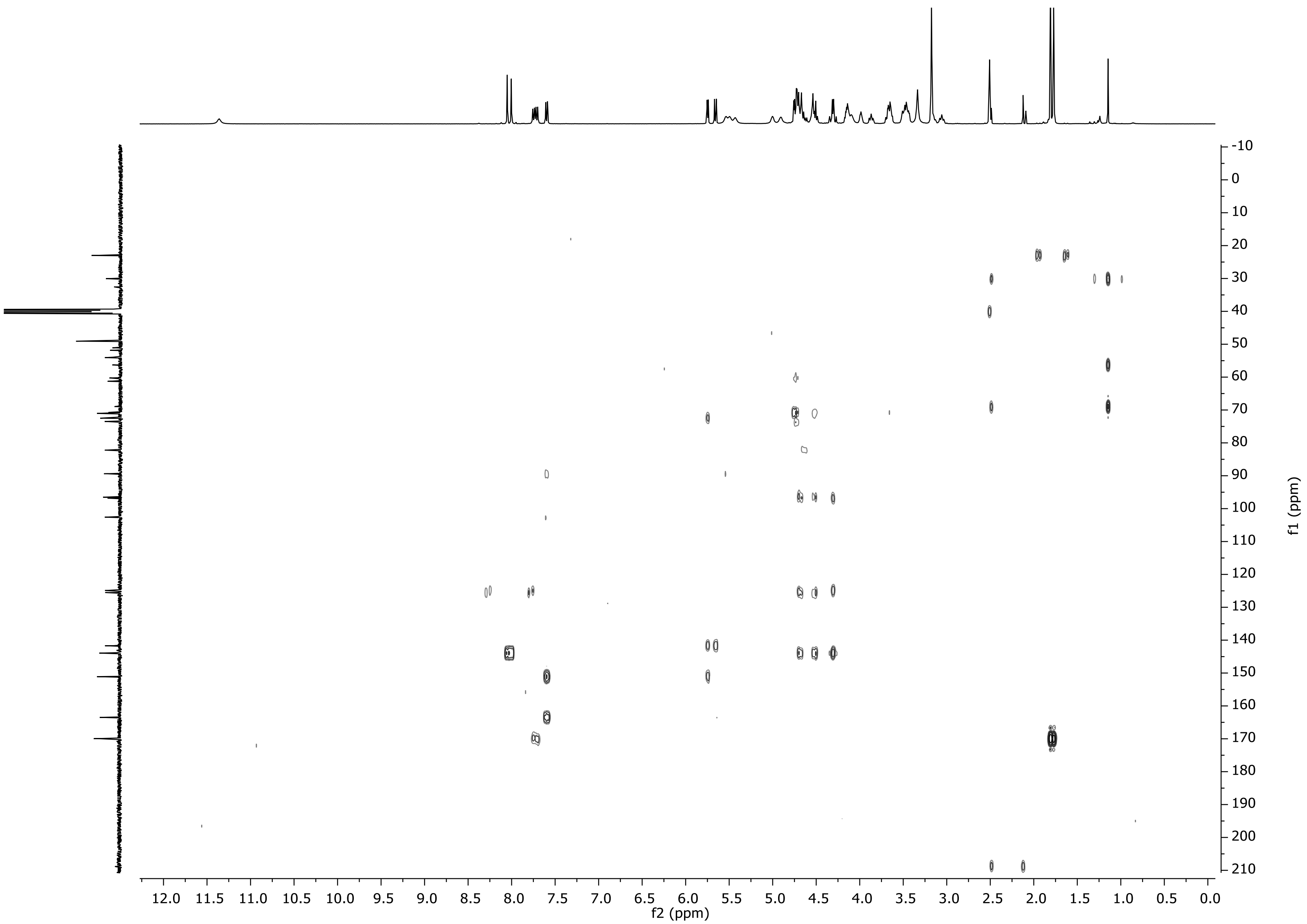
f2 (ppm)
f1 (ppm)
